# Vestiges of the Bacterial Signal Recognition Particle-Based Protein Targeting in Mitochondria

**DOI:** 10.1093/molbev/msab090

**Published:** 2021-04-10

**Authors:** Jan Pyrih, Tomáš Pánek, Ignacio Miguel Durante, Vendula Rašková, Kristýna Cimrhanzlová, Eva Kriegová, Anastasios D Tsaousis, Marek Eliáš, Julius Lukeš

**Affiliations:** 1 Institute of Parasitology, Biology Centre, Czech Academy of Sciences, České Budějovice (Budweis), Czech Republic; 2 Laboratory of Molecular and Evolutionary Parasitology, RAPID Group, School of Biosciences, University of Kent, Canterbury, United Kingdom; 3 Department of Biology and Ecology, Faculty of Science, University of Ostrava, Ostrava, Czech Republic; 4 Department of Zoology, Faculty of Science, Charles University, Prague, Czech Republic; 5 Faculty of Sciences, University of South Bohemia, České Budějovice (Budweis), Czech Republic

**Keywords:** evolution, Ffh, FtsY, LECA, mitochondrion, protein targeting, protists, signal recognition particle

## Abstract

The main bacterial pathway for inserting proteins into the plasma membrane relies on the signal recognition particle (SRP), composed of the Ffh protein and an associated RNA component, and the SRP-docking protein FtsY. Eukaryotes use an equivalent system of archaeal origin to deliver proteins into the endoplasmic reticulum, whereas a bacteria-derived SRP and FtsY function in the plastid. Here we report on the presence of homologs of the bacterial Ffh and FtsY proteins in various unrelated plastid-lacking unicellular eukaryotes, namely Heterolobosea, Alveida, *Goniomonas*, and Hemimastigophora. The monophyly of novel eukaryotic Ffh and FtsY groups, predicted mitochondrial localization experimentally confirmed for *Naegleria gruberi*, and a strong alphaproteobacterial affinity of the Ffh group, collectively suggest that they constitute parts of an ancestral mitochondrial signal peptide-based protein-targeting system inherited from the last eukaryotic common ancestor, but lost from the majority of extant eukaryotes. The ability of putative signal peptides, predicted in a subset of mitochondrial-encoded *N. gruberi* proteins, to target a reporter fluorescent protein into the endoplasmic reticulum of *Trypanosoma brucei*, likely through their interaction with the cytosolic SRP, provided further support for this notion. We also illustrate that known mitochondrial ribosome-interacting proteins implicated in membrane protein targeting in opisthokonts (Mba1, Mdm38, and Mrx15) are broadly conserved in eukaryotes and nonredundant with the mitochondrial SRP system. Finally, we identified a novel mitochondrial protein (MAP67) present in diverse eukaryotes and related to the signal peptide-binding domain of Ffh, which may well be a hitherto unrecognized component of the mitochondrial membrane protein-targeting machinery.

## Introduction

The mitochondrion evolved from an endosymbiont belonging to alphaproteobacteria (Roger et al. 2017; Martijn et al. 2018) and as a cellular component has transitioned into particularly varied forms in different branches of the eukaryotic tree. The key factors underpinning mitochondrial diversity in the extant eukaryotes are lineage-specific innovations and acquisitions, paralleled to a varying degree by losses of ancestral traits. Although mitochondria of conventional model organisms are rather canonical organelles, extremes are found among lesser-known unicellular eukaryotes (Smith and Keeling 2015; Leger et al. 2019; Gray et al. 2020). An example of an especially pronounced lineage-specific elaboration is provided by the kinetoplastid and diplonemid flagellates with baroquely complex structure and functions of their mitochondrial genomes and transcriptomes (Lukeš et al. 2018; Aphasizheva et al. 2020; Kaur et al. 2020). On the other hand, simplifications have dominated the mitochondrial adaptations of obligate anaerobes, which resulted in organelles without a genome and sometimes even without a function in energy metabolism (Leger et al. 2017; Santos et al. 2018). One such lineage, represented by the oxymonad *Monocercomonoides exilis*, has lost the mitochondrion completely (Karnkowska et al. 2016, 2019).

Somewhat less conspicuous are cases of extraordinary mitochondrial primitiveness, namely the retention of ancestral traits lost by the organelles of most other eukaryotes or at least the commonly studied ones. Some protist groups contain mitochondrial genomes (mitogenomes) that have retained genes relocated to the nuclear genome or completely lost in most other taxa (Kamikawa et al. 2016; Janouškovec et al. 2017). Perhaps the most spectacular example are jakobids with their mitogenomes still encoding subunits of the eubacterial-type RNA polymerase (Burger et al. 2013; Yabuki et al. 2018). Other primitive traits became apparent only with analyses of mitochondrial components encoded by the nuclear genome. The bacterial cytokinetic protein FtsZ present in mitochondria of various protists (Beech et al. 2000; Kiefel et al. 2004), some of which have even kept the regulatory Min system (Leger et al. 2015), is an obvious example. Another case is the recent discovery of a mitochondrial system that involves elements of the bacterial type II secretion system (Horváthová et al. 2021), which was most likely present in the last common eukaryotic ancestor (LECA), yet with the exception for a few little studied protist groups, it was lost in most modern lineages.

Altogether, a picture is emerging that the mitochondrion in the LECA was much more “bacterial” than would be inferred from comparing mitochondria of commonly studied eukaryotes. Here, we present evidence for a hitherto unnoticed bacterial piece of the mitochondrial puzzle that we uncovered while analyzing the mitochondrial proteome of the heterolobosean *Naegleria gruberi*, a free-living amoeboflagellate closely related to the “brain-eating” human pathogen *N. fowleri* (Fritz-Laylin et al. 2010). This piece relates to the mechanism of membrane protein targeting, briefly introduced in the following paragraphs to provide a background for the presentation of our findings.

**Fig. 1.**
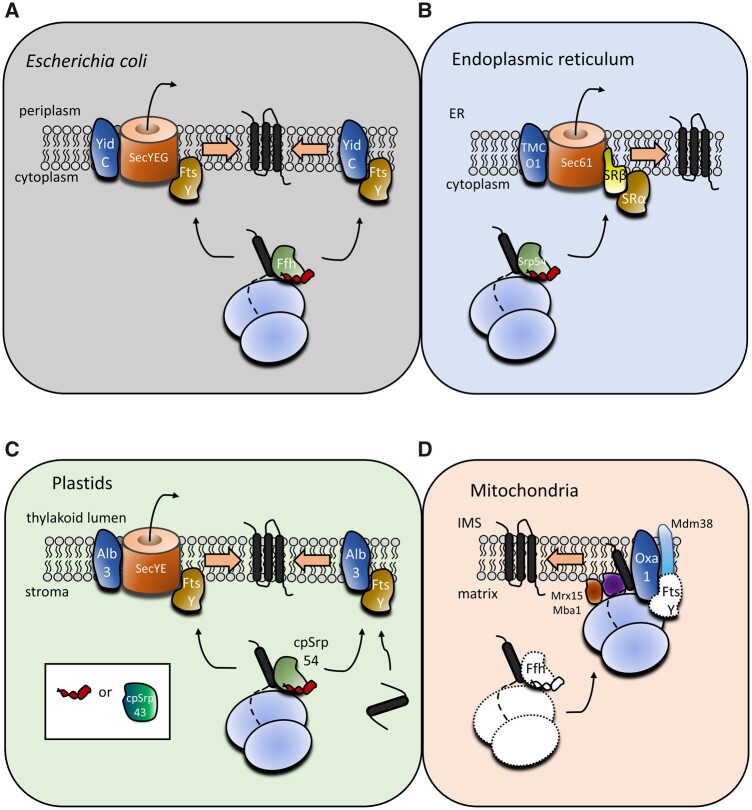
Simplified cartoon representation of evolutionarily related protein targeting systems in bacteria (exemplified by *Escherichia coli*) (*A*), eukaryotic endoplasmic reticulum (*B*), primary plastids (*C*), and mitochondria (exemplified by *Saccharomyces cerevisiae*) (*D*). Homologous components across the systems are rendered in the same color. The small item in red corresponds to the noncoding 4.5S RNA that together with the protein Ffh or its differently named homologs constitutes the SRP. The inset in part *C* indicates that in some plastids, 4.5S RNA is missing and replaced by the novel protein cpSRP43. The model for the mitochondrion (including the names of the proteins) is based primarily on the situation in yeast mitochondria and it is not certain to what extent it is valid for eukaryotes as a whole. White filling and dotted outlines in part *D* indicate ancestral bacterial features, that is, free (membrane-unbound) translating mitoribosomes and components of the SRP pathway presumably present in the proto-mitochondrion yet presently unknown from mitochondria of extant eukaryotes.

We are here primarily concerned with mechanisms mediating protein insertion into the bacterial plasma membrane and its evolutionary equivalents, the mitochondrial inner membrane (MIM) and the thylakoid membrane in plastids. In bacteria, most plasma membrane proteins reach their destination via a cotranslational mechanism dependent on two critical components, the signal recognition particle (SRP) and its receptor protein FtsY (Saraogi and Shan 2014; Steinberg et al. 2018). Being composed of the Ffh protein and an RNA component (called 4.5S RNA or 6S RNA, depending on the taxon), SRP recognizes hydrophobic N-terminal signal peptides of nascent proteins as they emerge from the translating ribosome. Peripherally associated with the plasma membrane, FtsY interacts with the SRP, tethering the ribosome-nascent chain complex to the membrane (fig. 1*A*). This enables docking of this complex to the SecYEG translocation channel, which mediates the integration of the nascent peptide chain into the membrane. An important element of the system is the membrane protein YidC, which functions either in conjunction with the SecYEG channel or as an independent insertase, depending on the substrate (Saraogi and Shan 2014; Steinberg et al. 2018).

It is noteworthy that all eukaryotes share a cytoplasmic signal peptide-driven pathway of cotranslational protein targeting to the endoplasmic reticulum (ER) (fig. 1*B*). This is clearly an evolutionary derivative of the SRP-based system for plasma membrane protein targeting that operated in the archaeal ancestors of eukaryotes (Zwieb and Bhuiyan 2010; Akopian et al. 2013). The eukaryotic SRP consists of SRP54, a homolog of the archaeal Ffh, and an associated RNA component (7SL RNA). The ER-associated SRP receptor is composed of two subunits, one of which (SRα) evolved from the archaeal FtsY. Furthermore, the plastid-bearing eukaryotes also encode Ffh and FtsY homologs closely related to the eubacterial proteins, which are localized to plastids, organelles derived from an endosymbiotic cyanobacterium (Ponce-Toledo et al. 2017), and function as parts of an SRP machinery mediating cotranslational targeting of membrane proteins encoded by the plastid genome (Ziehe et al. 2017). Many algal groups still possess homologs of all the key components of the system, including cpSRP54 (derived from the cyanobacterial Ffh), SRP RNA (specified by the *ffs* gene still residing in the plastid genome), and cpFtsY, whereas some plants and algae have lost the RNA component (Träger et al. 2012; Ševčíková et al. 2019). The plastid SRP system functions in two modes (fig. 1*C*): cotranslationally in cooperation with plastid equivalents of SecYEG and YidC (the plastid homolog of the later protein is called Alb3) or posttranslationally, bringing the nucleus-encoded photosynthetic antenna proteins to the Alb3 insertase for their integration into the thylakoid membrane (Ziehe et al. 2017, 2018).

Mitochondria have their own YidC homologs called Oxa1 and Oxa2 (alternatively termed Cox18), which mediate the insertion of both mitochondrial- and nuclear-encoded proteins into the MIM (Oxa1) or are involved in cytochrome *c* oxidase biogenesis (Oxa2) (Hennon et al. 2015). Furthermore, the core subunits of the IMP protease complex in the MIM, which is needed for proteolytic processing of several subunits of the respiratory chain, are related to the bacterial signal peptidase and thus seem to be a rudiment of the original signal peptide-mediated targeting pathway present in the bacterial ancestor of the mitochondrion (Behrens et al. 1991; Gakh et al. 2002). Interestingly, mitogenomes of certain jakobids encode a homolog of the SecY protein (Lang et al. 1997; Burger et al. 2013). If the whole SecYEG complex is present in the mitochondria of these protists as was suggested previously (Tong et al. 2011), it would represent a case of exceptional retention of another ancestral trait related to the SRP-dependent targeting pathway.

However, no mitochondrial equivalent of the SRP system has been reported to date, and a systematic search for its components by bioinformatic analyses of available eukaryotic genomes failed to identify any mitochondrial homologs of Ffh or FtsY (Glick and Von Heijne 1996; Funes et al. 2013). In fact, there is no place for Ffh or FtsY in the paradigmatic view of mitochondrial translation established primarily by studies on yeast and human (fig. 1*D*), according to which the mitoribosome is stably tethered to the MIM to ensure cotranslational integration of the proteins into the membrane (Ott and Herrmann 2010). In this system, the mitoribosome-membrane association relies on its interaction with a C-terminal extension of Oxa1 and several proteins that seem to be evolutionary innovations of the mitochondrion, including Mba1, Mdm38, and Mrx15 (Ott and Herrmann 2010; Funes et al. 2013; Möller-Hergt et al. 2018). Data available from other eukaryotic models suggest that the mechanism of cotranslational insertion of mitochondrial membrane proteins may be generally similar across distantly related taxa (Christian and Spremulli 2012; Kolli et al. 2018a), although a more detailed comparison is lacking.

Funes et al. (2013) suggested that the SRP system was initially present in mitochondria but became dispensable upon the loss of genes encoding soluble proteins from the mitogenome and was eventually lost due to the emergence of alternative mechanisms for stable association of the mitoribosome with the MIM. They further speculated that some protist lineages with mitogenomes still encoding hydrophilic proteins might represent an intermediate evolutionary stage with the SRP system possibly retained. Here, we demonstrate that this is indeed the case.

## Results

### *Naegleria gruberi* Possesses Mitochondrial Homologs of Ffh and FtsY

While examining a set of putative mitochondrial proteins of the heterolobosean *N. gruberi* defined by Localisation of Organelle Proteins by Isotope Tagging (LOPIT)-based proteomic analysis of cellular fractions (supplementary fig. S1, Supplementary Material online; for details, see Horváthová et al. 2021), we found two proteins, further referred to as *Ng*Ffh and *Ng*FtsY, more similar to the bacterial Ffh and FtsY proteins than to their eukaryotic homologs SRP54 and SRα. Comparison of the existing respective gene models (Fritz-Laylin et al. 2010) with the genome sequence of *N. fowleri* revealed that both are inaccurate, not only due to incorrectly delimited coding sequence (CDS) but in the case of mtFfh also due to a genome assembly issue (supplementary fig. S2, Supplementary Material online). Amendments to both gene models were confirmed by real time-polymerase chain reaction (PCR) amplification of the 5′ end of the respective transcripts and verified by proteomic data (supplementary fig. S1, Supplementary Material online). The corrected protein sequences (supplementary table S1, Supplementary Material online) were evaluated by multiple protein-targeting prediction tools, which suggested the presence of a mitochondrial presequence in both proteins (supplementary table S2, Supplementary Material online), consistent with their identification in the putative mitochondrial proteome.

**Fig. 2.**
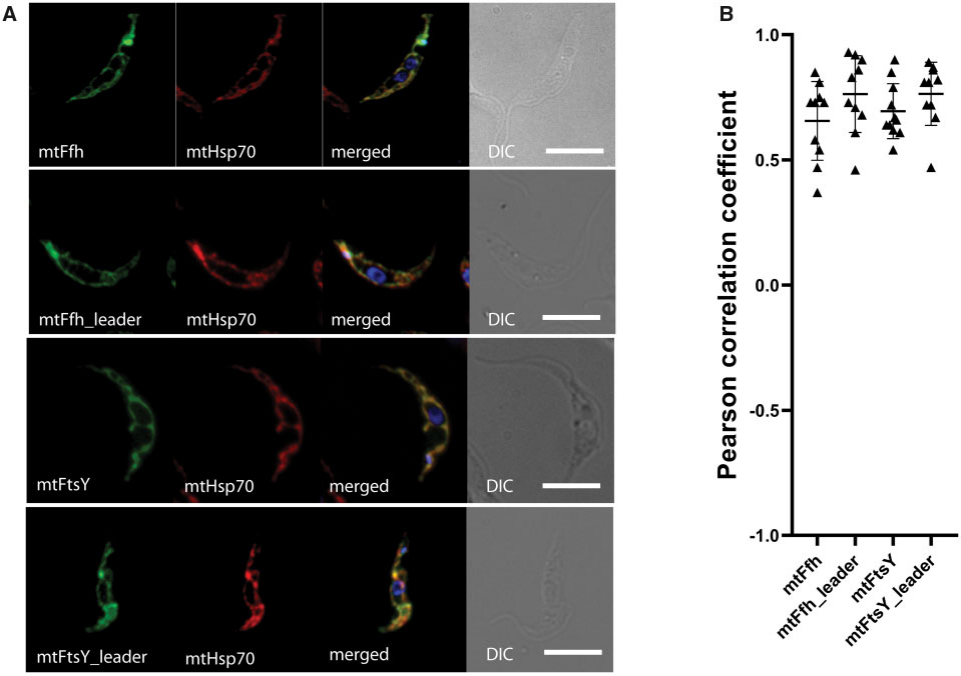
Mitochondrial localization of heterologously expressed mtFfh and mtFtsY from *Naegleria gruberi*. (*A*) Full-length proteins or their N-terminal leader sequences were expressed in *Trypanosoma brucei* as translation fusions with V5-tagged mNeonGreen protein and visualized by immunofluorescence staining using an α-V5 antibody. Monoclonal α-mtHsp70 antibody served as a mitochondrial marker; DAPI (blue channel) was used to stain DNA; DIC, differential interference contrast. (*B*) PCCs of fluorescent signal colocalization for ≥10 randomly selected cells in each individual cell line. The PCC values range from 1 (i.e., 100% correlation) to −1 (i.e., 100% anticorrelation); values close to 0 mean no correlation. PCC means with standard deviations are displayed for each cell line.

Next, the mitochondrial localization of *Ng*Ffh and *Ng*FtsY was tested in the heterologous system of the euglenozoan *Trypanosoma brucei*. Both the N-terminal region (supplementary fig. S1B, Supplementary Material online) and the complete CDSs of *Ng*Ffh and *Ng*FtsY were inserted upstream of the V5-tagged fluorescent mNeonGreen gene and integrated into the rDNA locus of *T. brucei*. Expressed fusion proteins were detected by immunofluorescence with an α-V5 antibody, which in all cases demonstrated colocalization with a mitochondrion-specific marker (α-mtHsp70 antibody) labeling the single reticulated mitochondrion of *T. brucei* (fig. 2). This indicates that the predicted mitochondrial presequences of the *N. gruberi* Ffh and FtsY proteins are recognized by the *T. brucei* mitochondrial protein import machinery, providing further evidence for the presence of homologs of the bacterial Ffh and FtsY proteins in the mitochondrion of *N. gruberi*.

### Mitochondrial Ffh and FtsY Have Been Retained in Several Protist Lineages

**Fig. 3.**
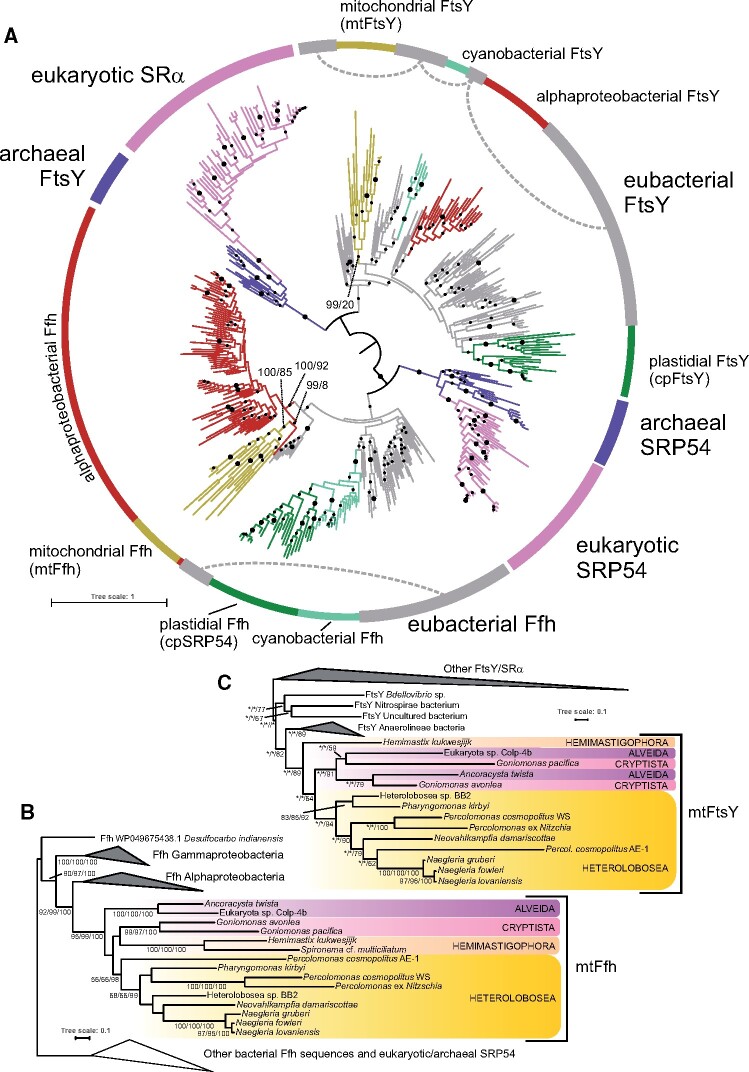
Phylogenetic analysis of the signal recognition–associated GTPase family gene family showing the position of the mitochondrial Ffh and FtsY homologs. The trees were inferred with the ML method in IQ-TREE (using the LG4X substitution model). Branch support was assessed by nonparametric bootstrapping (*N* = 500, IQ-TREE; only the trees in parts *B* and *C*), rapid bootstrapping (*N* = 500, RAxML), and ultrafast bootstrapping (*N* = 1,000, IQ-TREE) using the same substitution model. (*A*) Phylogenetic position of newly identified mitochondrial Ffh and FtsY proteins within the family (452 sequences, 287 amino acid positions). Branch support ≥95% in both methods or only one of them is indicated by a larger or a smaller black dot, respectively. The tree was arbitrarily rooted between the FtsY/SRα and Ffh/SRP groups. Ffh and FtsY homologs from *Paulinella* spp. chromatophore (not specifically highlighted) branch within Cyanobacteria. (*B*) Detailed phylogenetic analysis of Ffh/SRP54 proteins (295 sequences, 422 amino acid positions). (*C*) Detailed phylogenetic analysis of FtsY/SRα proteins (217 sequences, 367 amino acid positions). Full trees are provided (in the Newick format) in supplementary data set S1, Supplementary Material online.

To gain insights into the evolutionary origin of *Ng*Ffh and *Ng*FtsY, we carried out an exhaustive search for homologous genes in other eukaryotes. After excluding Ffh- and FtsY-related sequences most likely representing bacterial contaminants in the eukaryotic genome and transcriptome assemblies, our phylogenetic analysis revealed a broader set of sequences related to *Ng*Ffh and *Ng*FtsY (fig. 3). These sequences are authentic and not bacterial contaminants, as the corresponding genes (when available in genome assemblies) contain spliceosomal introns or are parts of genomic scaffolds containing unambiguous eukaryotic genes (supplementary table S1, Supplementary Material online). In addition, various prediction algorithms suggested mitochondrial localization for these proteins (supplementary table S2, Supplementary Material online). In case of the Ffh homologs, the putative mitochondrial presequences are apparent as an N-terminal extension missing in bacterial proteins (supplementary fig. S3, Supplementary Material online), whereas the FtsY sequences are insufficiently conserved at the N-terminus to allow such a comparison. As the mitochondrial localization seems to be a common feature of the Ffh and FtsY homologs beyond the experimentally investigated ones in *N. gruberi*, we here denote them mtFfh and mtFtsY, respectively.

**Fig. 4.**
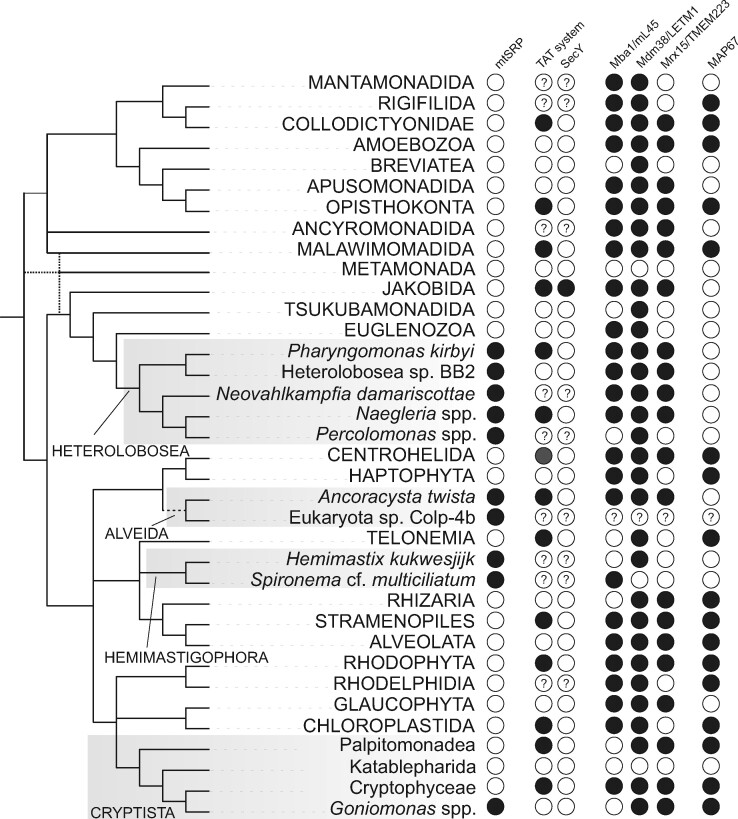
Phylogenetic distribution of proteins and systems involved in membrane protein targeting and translocation in mitochondria. The schematic phylogeny of eukaryotes is plotted as a consensus of recent phylogenomic analyses of the eukaryote phylogeny (reviewed in Burki et al. 2020) The presence of the mitochondrial Ffh and FtsY (mtSRP), Twin-arginine translocation (TAT) complex, SecY, (putative) mitoribosome membrane receptors (Mba1/mL45, Mdm38/LETM1, and Mrx15/TMEM223), and the novel mitochondrial Ffh-related protein MAP67 is indicated for the main eukaryotic lineages if documented from at least one representative (filled circle). Empty circles indicate that the components are absent from the taxon or has not been identified (which may not necessarily mean true absence, given poor conservation of some of the proteins and limited sampling for many of the main eukaryotic lineages). Question marks indicate that relevant data are missing (mitochondrial genome sequences in case of TAT and SecY) or were not available for analysis (transcriptome assembly from Eukaryota sp. Colp-4b). Data on the occurrence of the TAT complex and SecY were adopted from Petrů et al. (2018) and Tong et al. (2011), respectively, with further updates based on Nishimura et al. (2019) and Wideman et al. (2020).

Based on the current sampling of the eukaryotic diversity, mtFfh and mtFtsY are restricted to four distantly related eukaryotic lineages, namely Heterolobosea, Hemimastigophora, Alveida, and the genus *Goniomonas* from the supergroup Cryptista. In Heterolobosea, both mtFfh and mtFtsY were found in all species for which sufficiently complete sequence data are available (supplementary table S1, Supplementary Material online), indicating a widespread occurrence of the mitochondrial SRP system in this group (fig. 4). For Hemimastigophora, a recently recognized deep-branching eukaryote lineage (Lax et al. 2018), single-cell transcriptome assemblies yielded both mtFfh and mtFtsY homologs in *Hemimastix kukwesjijk* but only a mtFfh homolog in *Spironema* cf. *multiciliatum*, which most likely reflects an incomplete representation of the gene repertoire in the latter species. Alveida is another recently identified deep-branching lineage containing *Ancoracysta twista* (Janouškovec et al. 2017; Cavalier-Smith et al. 2018) and the isolate Colp-4b (Tikhonenkov DV, personal communication). Although both mtFfh and mtFtsY were found in the transcriptome assembly of Colp-4b, the assembly of *A. twista* contained only the former gene, yet a careful examination of the unassembled raw RNAseq reads allowed us to assemble a partial sequence that falls into the mtFtsY clade (fig. 3*C* and supplementary table S1, Supplementary Material online). Finally, both mtFfh and mtFtsY sequences were recovered from the genome and/or transcriptome assemblies available for two deeply diverged representatives of the genus *Goniomonas*, *G. avonlea* and *G. pacifica* (supplementary table S1, Supplementary Material online). Interestingly, no mtFfh and mtFtsY candidates were found in other members of Cryptista with genome-scale data available, including diverse algal species of the Cryptophyceae class and the heterotrophic flagellates *Palpitomonas bilix* and *Roombia truncata*.

Phylogenetic analyses resolved mtFfh and mtFtsY sequences as novel clades within the signal recognition–associated GTPase family nested among bacterial sequences but unrelated to the previously known plastid homologs cpSRP54 and cpFtsY (fig. 3). There is strong evidence for the monophyly of mtFfh, with the clade receiving maximal ultrafast bootstrap values in a broad analysis of the whole signal recognition–associated GTPase family (fig. 3*A*) and an analysis restricted to Ffh/SRP54 sequences (fig. 3*B*). For the later data set, we also calculated real nonparametric bootstrap values, providing 95% support for the mtFfh clade. The internal topology of the mtFfh clade is generally congruent with the relationships among and within the four major organismal lineages (fig. 3*B*), which is consistent with vertical inheritance of mtFfh in eukaryotes. Furthermore, the mtFfh clade forms a sister group to sequences from alphaproteobacteria (fig. 3*B*) or is even nested within them (fig. 3*A*). In the later case, it branches as a sister group to an Ffh homolog from the uncultivated alphaproteobacterium “MarineAlpha2.” Importantly, since the relationship of mtFfh and alphaproteobacterial Ffh is strongly supported in both analyses (fig. 3*A* and *B*), mtFfh most likely evolved from Ffh of the alphaproteobacterial ancestor of the mitochondrion.

Presumably due to a more divergent nature of mtFtsY reflected by relatively long branches in the phylogenetic trees (fig. 3*A* and *C*) and a lower number of informative positions (290 vs. 410), its evolutionary history could be reconstructed less robustly than that of mtFfh. Still, the mtFtsY clade is retrieved in both the FtsY/SRα-only (fig. 3*C*) and FtsY-only analyses (supplementary fig. S4, Supplementary Material online) and supported by 87–89% ultrafast bootstrap replicates. When the most divergent and partial mtFtsY sequence of *Percolomonas cosmopolitus* strain AE-1 was removed from the data set, the ultrafast bootstrap support for the mtFtsY clade increased to 99% in the analysis of the whole signal recognition–associated GTPase family and its internal topology became generally congruent with the known relationship among the species (fig. 3*A* and supplementary data set S1, Supplementary Material online). On the other hand, the phylogenetic position of the mtFtsY clade among the bacterial FtsY sequences is poorly resolved in all three analyses and the provenance of the closest relatives differs, in neither case being alphaproteobacterial. However, alternative hypotheses that mtFtsY evolved within or sister to alphaproteobacterial FtsY were not rejected by the approximately unbiased (AU) test (supplementary table S3, Supplementary Material online) when applied to the FtsY-only data set. It is worth noting that the plastidial cpSRP54 and cpFtsY are only distantly related to their respective mitochondrial homologs and that the relation to cyanobacterial equivalents is supported only for the former protein. Similar to mtFtsY, the origin of cpFtsY remains unresolved by our analyses, but the a priori expected cyanobacterial ancestry cannot be rejected by the AU test (supplementary table S3, Supplementary Material online).

### Mitochondrial Signal Recognition Particle Lacks the RNA Component

The existence of mtFfh protein in certain eukaryotes raises an obvious question whether a counterpart of the conserved SRP RNA molecule, which together with Ffh constitutes the bacterial SRP, was also retained in the mitochondrion. SRP RNA is poorly conserved in structure and sequence across distantly related taxa, ranging from the 110-nt-long 4.5S RNA of *E*scherichia *coli* to the 7S RNA of approximately 300 nt found in the archaeal and eukaryotic SRP (Regalia et al. 2002). Hence, we employed a sensitive search strategy using covariance models built based on two different variants of bacterial SRP RNA defined by the Rfam database. As a control, the covariance model representing protistan 7SL RNA (i.e., component of the cytoplasmic SRP) was used. Although the later model identified clear homologs in the nuclear genomes of the mtFfh-carrying species, no candidates for a bacteria-like *ffs* gene were detected.

The failure to detect the RNA component of the putative mitochondrial SRP may be formally explained by its divergence beyond recognition by bacterial covariance models. We reasoned that analogously to the plastid SRP system, the possible mitochondrial SRP RNA—if present at all—would most likely be produced by transcription of a gene residing in the mitogenome. Furthermore, such a gene would possibly be sufficiently conserved between the closely related species to allow its detection by sequence comparison. We thus systematically compared the predicted intergenic regions of the complete mitogenomes of *N. gruberi* and *N. fowleri* (the only pair of closely related mtFfh-carrying species with the mitogenome sequences available) to see if any of them exhibits conservation suggestive of a functional constraint. This allowed the identification of an unannotated homolog of Rpl19 noticed previously (Janouškovec et al. 2017) and a short open reading frame of unknown function conserved in multiple heterolobosean species (supplementary fig. S5A, Supplementary Material online), but no candidate RNA gene was found.

These results suggest that no gene for SRP RNA exists in the mitogenomes of eukaryotes harboring mtFfh, with a theoretical exception of Hemimastigophora, for which genome sequences are not yet available. In this regard, it is instructive to consider the situation with the plastidial (chloroplast) SRP (cpSRP). The *ffs* gene is present in plastid genomes of various algae and plants, but many lineages have independently lost it and there is a direct biochemical evidence for the absence of the RNA component in cpSRP of seed plants (Träger et al. 2012). In addition, the absence of *ffs* perfectly correlates with mutations in two specific motifs of cpSRP54 that are critical for its interaction with 4.5S RNA, suggesting that an alternative nuclear gene does not seem to exist in these taxa and the RNA component has indeed been lost (Träger et al. 2012; Ševčíková et al. 2019). Therefore, we checked the corresponding motifs in the mtFfh proteins and found that they are similarly mutated (supplementary fig. S5*B*, Supplementary Material online). This finding further supports the hypothesis that the mitochondrial SRP is devoid of an RNA component similar to its plastidial counterpart.

### N-Termini of Some *N. gruberi* Mitochondrial Proteins Function as Signal Peptides

**Fig. 5.**
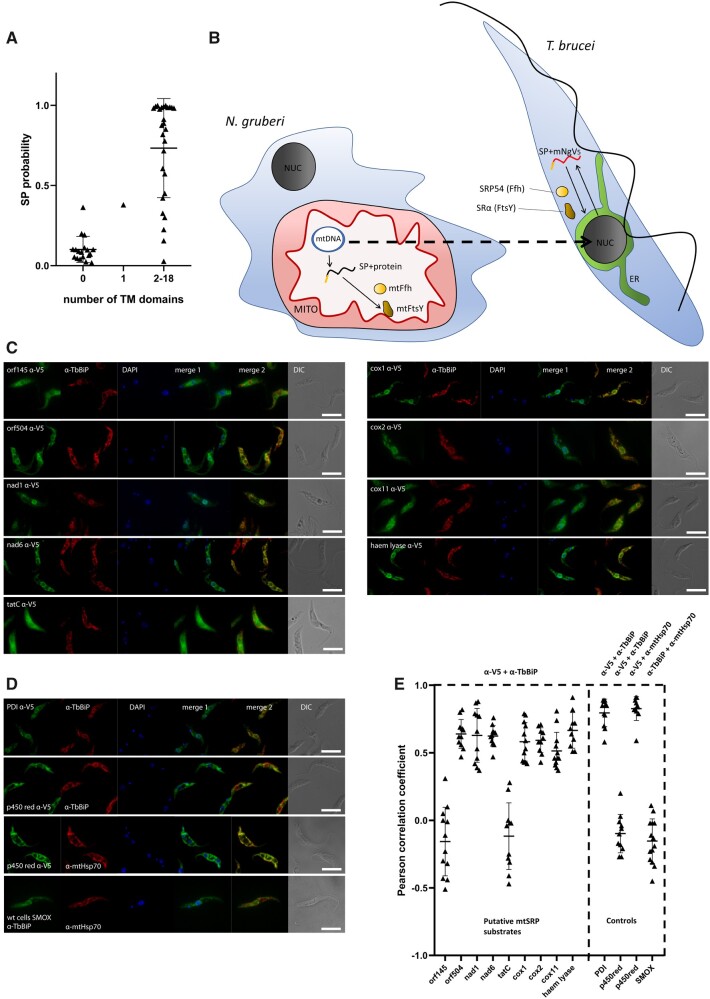
N-terminal regions of selected *Naegleria gruberi* mitochondrial proteins are recognized as signal peptides by the cytosolic SRP. (*A*) Correlation between the probability of an *N. gruberi* mitochondrion-encoded protein to contain a signal peptide (as predicted with TargetP-1.1) and the number of TM domains (detected via TMHMM server). Mann–Whitney test calculated *P* value below 0.0001. The single protein with a single predicted TM is the ribosomal protein S4, representing a likely false-positive prediction. (*B*) Scheme of experiment. NUC, nucleus; MITO, mitochondrion; ER, endoplasmic reticulum. Small arrows indicate gene expression and subcellular targeting, the dashed arrow indicates nuclear transfection of *Trypanosoma brucei* with DNA constructs encoding fusion proteins comprised putative SPs of *N. gruberi* mitochondrion-encoded proteins and V5-tagged mNeonGreen (mNg). (*C*) Codon-optimized 5′ segments of *N. gruberi* mitochondrial genes (encoding N-terminal regions of the respective proteins) were fused with the reporter V5-tagged mNeonGreen gene and integrated into the *T. brucei* nuclear genome. Except for orf145 and tatC cell lines, the fusion proteins (α-V5 antibody signal) colocalized with the signal of an α-TbBiP antibody, which served as an ER marker. (*D*) Control experiments. Top: the N-terminal region of the *T. brucei* Protein disulfide isomerase (PDI) protein targets V5-tagged mNeonGreen into the ER (positive control). Middle: the N-terminal region of the *T. brucei* NADPH-cytochrome p450 reductase targets V5-tagged mNeonGreen to the mitochondrion (specificity control). Bottom: No colocalization between ER and mitochondrial marker was observed in wild-type (SMOX) cell line. The mitochondrion was specifically labeled by the α-mtHsp70 antibody. DAPI (blue channel) represents DNA; merge 1—an overlay of α-V5 and DAPI signals; merge 2—an overlay of α-V5 and α-TbBiP signals; DIC, differential interference contrast. (*E*) PCCs of fluorescence signal colocalization for ≥10 randomly selected cells in each individual cell line (see the legend to fig. 2*B* for further details).

Given the evolutionary derivation of the mtFfh/mtFtsY system from the bacterial Ffh/FtsY system and considering the precedent of the analogous plastidial cpSRP54/cpFtsY system, it is reasonable to assume that it mediates targeting of specific protein substrates into the MIM and that this targeting depends on the interaction of mtFfh with the N-terminal signal peptides of the client proteins. Consistently with this hypothesis, 20 out of 46 proteins encoded by the *N. gruberi* mitogenome carry signal peptides predicted by dedicated bioinformatics tools (supplementary table S4, Supplementary Material online). Furthermore, we have identified a strong correlation (*P* value <0.0001) between the presence of transmembrane (TM) domains and a predicted signal peptide in the mitochondrial-encoded proteins (fig. 5*A*). All proteins where a signal peptide was predicted with the probability exceeding 50% possess two or more TM domains, which is consistent with the assumption that signal peptide targets the protein into the MIM. One protein, the ribosomal protein S4, was predicted to contain a single TM located in the N-terminal region, which is likely a false-positive results due to the function of this protein. The respective region is neither a strong candidate for an SP (fig. 5*A*).

To test the functionality of these putative signal peptides in vivo, we expressed codon-optimized N-terminal regions of nine mitochondrial-encoded candidates as translational fusions with V5-tagged mNeonGreen from the pT7 vector stably integrated into the nuclear genome of *T. brucei* (for the scheme of the experiment see fig. 5*B*). Seven proteins were targeted into the ER, which in *T. brucei* forms a reticulated structure with a central perinuclear ring, whereas two—those with the N-terminal parts derived from *orf145* and *tatC* genes, remained in the cytoplasm, possibly in some granules (fig. 5*C*). Calculation of the hydrophobicity values (free insertion energy; ΔG, kcal/mol; Björkholm et al. 2015) of the putative signal peptides present in the tested N-terminal sequences revealed that the N-terminus of the *tatC*-encoded protein is by far the most hydrophobic. Thus, this protein serves as an additional specificity control excluding the possibility that the protein constructs could be dragged toward the ER simply because of their hydrophobicity. Based on these observations, we conclude that signal peptide-like N-termini of at least seven *N. gruberi* mitochondrial proteins are efficiently recognized by the cytoplasmic (i.e., eukaryotic) SRP-based targeting system in *T. brucei*. As a control, the N-terminus of NADPH-cytochrome p450 reductase from *T. brucei*, which resides in the outer mitochondrial membrane (Niemann et al. 2013) as a predicted signal-anchored protein, targeted the fused V5-tagged mNeonGreen reporter to the mitochondrion (fig. 5*D*). The colocalization of the tested proteins with specific ER or mitochondrial markers (TbBiP and mtHsp70, respectively) was assessed by calculating Pearson correlation coefficients (PCCs) based on ≥10 cells per each individual cell line, with the results consistent with the visual assessment of the fluorescence signals (fig. 5*E*). As a further control, PCC was calculated also for TbBiP and mtHsp70 antibody staining in wild-type (SMOX) cells, which indicated no correlation (fig. 5*E*), consistent with previous studies where those two antibodies were used (Dawoody et al. 2020). This verifies that in our overexpression system the cellular targeting mechanism distinguishes between SRP-dependent signal peptides and similar, yet SRP-independent N-terminal targeting determinants.

### Proteins Implicated in the Mitoribosome–MIM Association in Opisthokonts Are Widespread in Eukaryotes

The most common functional partner of the bacterial SRP system is the SecYEG channel residing in the plasma membrane (fig. 1*A*), which raises the question as to whether a similar partnership also exists in the mtFfh/mtFtsY-containing mitochondria. An SecY homolog is encoded by the mitogenomes of some jakobid flagellates (Burger et al. 2013), but the recently reported draft genome from a member of this group, *Reclinomonas americana* (Horváthová et al. 2021), indicate the absence of mtFfh and mtFtsY from these organisms. On the other hand, our reinvestigation of the mitogenomes, nuclear genomes, and/or transcriptomes of mtFfh/mtFtsY-carrying taxa did not identify any homologs of the SecYEG complex subunits, indicating a genuine absence of the SecYEG complex. Notably, cotranslational integration of a subset of bacterial proteins into a membrane does not depend on the SecYEG complex and is instead mediated by the insertase YidC as an alternative partner of the SRP system (Steinberg et al. 2018). Mitochondria of yeasts, metazoans, and plants contain two or more YidC homologs, typified by the yeast proteins Oxa1 and Cox18, which are involved in membrane integration or biogenesis of the MIM proteins in an SRP-independent fashion (Hennon et al. 2015; Kolli et al. 2018b). In opisthokonts, Oxa1 interacts directly with the mitoribosome via its C-terminal extension containing a coiled-coil motif (Jia et al. 2003). We examined the genomic and/or transcriptomic data of the mtFfh/mtFtsY-carrying species using a profile HMM specific for the YidC/Oxa1 family. One or two homologs were retrieved for each species (supplementary table S5, Supplementary Material online), and according to phylogenetic analysis, “bona fide” Oxa1 is ubiquitous among the species, with some of them additionally containing a putative Cox18 (Oxa2) ortholog (supplementary fig. S6, Supplementary Material online). Furthermore, the Oxa1 proteins from all mtFfh/mtFtsY-carrying species exhibit C-terminal extensions when compared with the bacterial YidC (supplementary fig. S7, Supplementary Material online), suggesting that the conventional mode of mitoribosome-Oxa1 interaction is preserved in these taxa and therefore was likely already present in the LECA.

The membrane association of a translating mitoribosome depends on additional proteins. Studies performed primarily in the yeast *Saccharomyces cerevisiae* identified three proteins involved in tethering the mitoribosome to the MIM. The best characterized is Mba1 (Ott and Herrmann 2010; Pfeffer et al. 2015), an ortholog of a bona fide mitoribosomal protein mL45 (Mrpl45) (Desmond et al. 2011). Recent cryo-EM studies in the mammalian systems revealed that mL45 participates in a characteristic prberance of the large mitoribosomal subunit and like Mba1 mediates the contact of the mitoribosome with the MIM (Greber et al. 2014; Pfeffer et al. 2015; Englmeier et al. 2017), raising the possibility that this function is more broadly conserved, if not ancestral, in eukaryotes as a whole. Mdm38 is another yeast mitoribosomal membrane receptor, with orthologs in other eukaryotes generally called LETM1 (Hashimi et al. 2013; Austin and Nowikovsky 2019). Mdm38/LETM1 are MIM-localized ion transporters, and whether they function as mitoribosome receptors in eukaryotes other than fungi is not clear. Using a specific profile HMM, we have identified orthologs of both Mba1/mL45 and Mdm38/LETM1 in most major eukaryotic lineages, including those with the mitochondrial SRP system (fig. 4 and supplementary table S6, Supplementary Material online).

Finally, Mrx15 is a newly described yeast mitoribosomal receptor organizing, jointly with Mba1, cotranslational membrane protein insertion (Möller-Hergt et al. 2018). Although proposed to be confined to fungi, our PSI-Blast search (Altschul et al. 1997) with the yeast Mrx15 as a query detected significant similarity to proteins in other eukaryotes including humans, where the homolog is called TMEM223 and besides its mitochondrial localization (Mallmann et al. 2019; Sánchez-Caballero et al. 2020), nothing is known about its function. Further analyses using a profile HMM corroborated the existence of a family of Mrx15-/TMEM223-related proteins, which is widely distributed in eukaryotes including most of the mitochondrial SRP-containing protists (supplementary table S6, Supplementary Material online). The unity of the proposed Mrx15/TMEM223 family is supported by the shared presence of two predicted transmembrane domains (supplementary fig. S8, Supplementary Material online), which were experimentally confirmed for the yeast Mrx15 (Möller-Hergt et al. 2018). Our results indicate that an ancestor of the Mrx15/TMEM223 family was likely already present in the LECA (fig. 4). This has been independently proposed in a recent study (Sánchez-Caballero et al. 2020) based on a much more restricted taxon sampling than employed here.

### A Novel Mitochondrial Ffh-Related Protein Occurs in a Broad Range of Eukaryotes

While searching for mtFfh candidates in genome or transcriptome assemblies of diverse eukaryotes, we noticed in some of them weak hits different from the genuine mtFfh or other known proteins. Closer investigation of the corresponding sequences revealed that they constitute a novel protein family related to Ffh/Srp54. These proteins are generally predicted to be targeted to the mitochondrion (supplementary table S7, Supplementary Material online) and the respective representatives were found by mass spectrometry in the mitochondrion of *Toxoplasma gondii* (TGME49_254230; Seidi et al. 2018), *Arabidopsis thaliana* (AT3G04950; Fuchs et al. 2020), and *Chlamydomonas reinhardtii* (v3 annotation ID 184930; Atteia et al. 2009). In the later species, the protein was listed among mitochondrial proteins of unknown function with the label MAP67, which we adopt here for the whole new protein family. The MAP67 family is broadly distributed in eukaryotes, being present in most major lineages, in some taxa even in more than one version (fig. 4 and supplementary table S7, Supplementary Material online). Notable exceptions are Metazoa, Fungi, Discoba, and Metamonada. Furthermore, we found MAP67 in one of the four mitochondrial SRP-bearing lineages, namely in the genus *Goniomonas*.

**Fig. 6.**
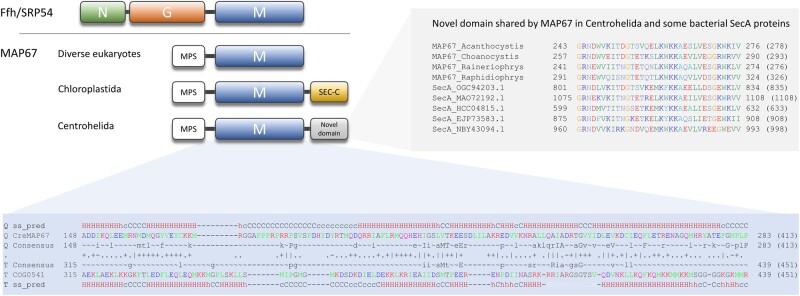
MAP67, a novel mitochondrial Ffh-related protein. Top left: schematic comparison of the domain architecture of Ffh/SRP54 and different variants of MAP67. MPS—mitochondrial presequence. Bottom: alignment of profile HMMs of MAP67 (with the *Chlamydomonas reinhardtii* MAP67 sequence shown as a reference) and the family COG0541 (Ffh represented in the Clusters of Orthologous Groups database) as retrieved by HHpred. The numbers on the left and right indicate coordinates along the length of the profile HMMs (the numbers in brackets correspond to the total length of the profile HMMs). The alignment is sandwiched by predictions of the secondary structure elements provided by HHpred. Top right: multiple sequence alignment of the novel conserve domain found at the C-terminus of MAP67 proteins from Centrohelida (included are all four sequences available) and a subset of bacterial SecA proteins (five sequences selected as a reference). IDs for the centrohelid sequences are provided in supplementary table S7, Supplementary Material online. The bacterial sequences (defined by GenBank accession numbers) come from the following bacterial taxa: OGC94203.1—Candidatus Adlerbacteria bacterium RIFOXYB1_FULL_48_1; MAO72192.1—Flavobacteriales bacterium; HCC04815.1—Patescibacteria group bacterium; EJP73583.1—SAR86 cluster bacterium SAR86B; NBY43094.1—Verrucomicrobia bacterium.

Based on sensitive homology searches, MAP67 proteins are along most of their length homologous to the signal peptide-binding M domain of Ffh/SRP54 proteins (fig. 6). Specifically, HHpred found a match to this domain (Pfam PF02978) with a probability of 99.66% and an *E*-value of 1.4e−15. In addition, the fold prediction server Phyre2 modeled 72% of the length of a reference MAP67 query (from the malawimonad *Gefionella okellyi*) with 100% confidence based on SRP54 from the archaebacterium *Methanocaldococcus jannaschii* as the best template. Interestingly, MAP67 proteins of two different eukaryote groups exhibit short conserved C-terminal extensions. In Chloroplastida, it includes a region matching the SEC-C domain (Pfam PF02810), which is also called the metal-binding domain (MBD) and occurs primarily at the C-terminus of bacterial SecA proteins (Jamshad et al. 2019). The MBD in SecA includes four positions occupied by metal ion-binding cysteine or histidine residues. Its variant in MAP67 proteins from Chloroplastida also includes four cysteine residues, although their positioning is not necessarily the same as in SecA (fig. 6). The second eukaryotic group with C-terminally extended MAP67 is Centrohelida, where the extension consists of a poorly conserved low complexity linker followed by a short highly conserved region of approximately 35 residues homologous to the C-terminus of a subset of bacterial SecA proteins (fig. 6). However, it lacks the characteristic cysteine residues, and HHpred did not detect even a remote similarity to the canonical MBD. Hence, MAP67 independently recruited two different versions of the C-terminus of bacterial SecA proteins in two different eukaryote lineages.

## Discussion

Here we show that at least four distantly related eukaryotic lineages (Heterolobosea, Hemimastigophora, Alveida, and *Goniomonas* spp.) harbor homologs of the bacterial Ffh and FtsY proteins that are unrelated to the previously known cyanobacteria-derived cpSRP54 and cpFtsY functioning in the plastids. Two lines of evidence—proteomic data and expression in a heterologous system—conclusively demonstrate that the respective proteins from *N. gruberi* function within the mitochondrion. Considering additional bioinformatic evidence for the mitochondrial localization of their homologs in other eukaryotes, we labeled these proteins as mtFfh and mtFtsY. Phylogenetic analyses indicate their common origin and are consistent with the vertical inheritance of the gene pair from a common ancestor of the respective eukaryotic lineages. The four mtFfh/mtFtsY-harboring groups represent diverse lineages of the proposed “megagroup” Diphoda (Derelle et al. 2015; Lax et al. 2018), which implies that both proteins appeared no later than in the last common ancestor of this clade. However, the alphaproteobacterial origin evident for mtFfh (and not excluded for mtFtsY) suggests an even more ancient origin, specifically from genes of the proto-mitochondrion. This would by inference mean that mtFfh and mtFtsY were possibly present in the LECA and were lost multiple times in a coordinated manner, supporting their functional interdependence.

Meanwhile, it is noteworthy to compare the evolutionary patterns of the SRP system in the plastids and mitochondria. Except for euglenophytes, the plastidial system is ubiquitous (Záhonová et al. 2018), attesting to its tight integration into the molecular fabric of this cyanobacterium-derived organelle. In contrast, the mitochondrial version has been dispensed with on multiple occasions. Moreover, in some taxa, the plastidial system retains its RNA component (Träger et al. 2012), whereas the available evidence suggests that the corresponding SRP RNA had most likely been present in the alphaproteobacterial ancestor of the mitochondrion, yet was lost prior to the LECA. Another difference rests in the fact that protein targeting mediated by the plastidial SRP system depends on an equivalent of the SecYEG translocation channel (Ziehe et al. 2017), which is missing from eukaryotes bearing the mitochondrial SRP system (at least from those where relevant data are available). Interestingly, the plastidial SRP system has become engaged in posttranslational insertion into the thylakoid membrane of the nucleus-encoded antenna proteins, which (at least in the land plants) depends on a novel protein factor called cpSRP43 interacting with cpSRP (Ziehe et al. 2017, 2018). We wondered whether analogously to cpSRP, mtFfh is accompanied by another novel factor. Following a phylogenetic profiling approach, previously successful in illuminating another patchily distributed mitochondrial system (Horváthová et al. 2021), we looked for proteins with the same or similar phylogenetic profile as mtFfh/mtFtsY but did not find any cooccurring candidates. Nevertheless, the existence of a eukaryote-specific component of the mitochondrial SRP system remains an open possibility that needs to be addressed by more direct approaches.

Since none of the eukaryotes carrying the mitochondrial SRP system is presently amenable to genetic manipulations, it is difficult to address its composition and function by experimental approaches. Assuming functional conservation dating back to bacterial ancestors of the mitochondrion as the most parsimonious alternative, the dissected system is involved in cotranslational membrane protein targeting. Hence, we evaluated the ability of the N-terminal sequences of the mitochondrial-encoded *N. gruberi* proteins that bear characteristics of a signal peptide to navigate a fused reporter fluorescent protein into the ER of genetically tractable *T. brucei*. Previous reports demonstrated that most proteins encoded by the human mitogenome are mistargeted to the ER when expressed from engineered nuclear copies of the respective genes, even when provided with a strong mitochondrial presequence (Björkholm et al. 2015, 2017). This suggested capturing of transmembrane domains in these proteins by the cytosolic SRP analogously to the recognition of signal-anchor sequences in proteins normally targeted to the ER membrane. Our experiments extend these observations by showing that the N-terminal regions of some nonhuman mitochondrial proteins are interpreted by the cytosolic SRP as bona fide signal peptides. Given the fact that the eukaryotic cytosolic SRP is related, however distantly, to the eubacterial SRP, this suggests that the N-termini of these proteins are likewise recognized by mtFfh when they emerge as nascent peptides from a translating mitoribosome. Following the functional paradigm established for both the eubacterial and eukaryotic SRPs, this leads to relocation of the ribosome-nascent chain-mtFfh complex to the MIM mediated by interaction with the membrane-associated mtFtsY receptor. Since the SecYEG complex is absent from eukaryotes known to have the mtFfh/mtFtsY system, the ubiquitous YidC homolog Oxa1 is an obvious candidate for mediating cotranslational membrane insertion of the nascent protein.

Although the cotranslational function of the mitochondrial SRP system is the default hypothesis to test, the ability of the plastidial SRP system to function in a posttranslational mode suggests that such a possibility cannot be dismissed either. Indeed, numerous nucleus-encoded proteins are translocated into the matrix and then inserted into the MIM by Oxa1 working in a posttranslational mode (Stiller et al. 2016; Kolli et al. 2018a). The assistance of the mitochondrial SRP system in such a delivery route would be analogous to the role of the plastidial SRP system in the integration of light-harvesting chlorophyll *a*/*b*-binding proteins into the thylakoid membrane mediated by the YidC homolog Alb3 (Ziehe et al. 2018). We also considered a possibility that the mitochondrial SRP system interacts with another protein translocase of bacterial origin, the TAT complex located in the MIM of some mitochondria, although this would represent a setting that is unprecedented in bacteria. The mitochondrial TAT components are indeed present in heteroloboseans and the alveid *A. twista* (Petrů et al. 2018). The lack of mitochondrial genome sequences from Hemimastigophora precludes determining if the TAT complex is present in this group. In any case, the recently sequenced mitogenome of *G. avonlea* lacks genes for any TAT subunit (Cenci et al. 2018), making the hypothetical functional link between the mitochondrial SRP system and the TAT translocase unlikely.

**Fig. 7.**
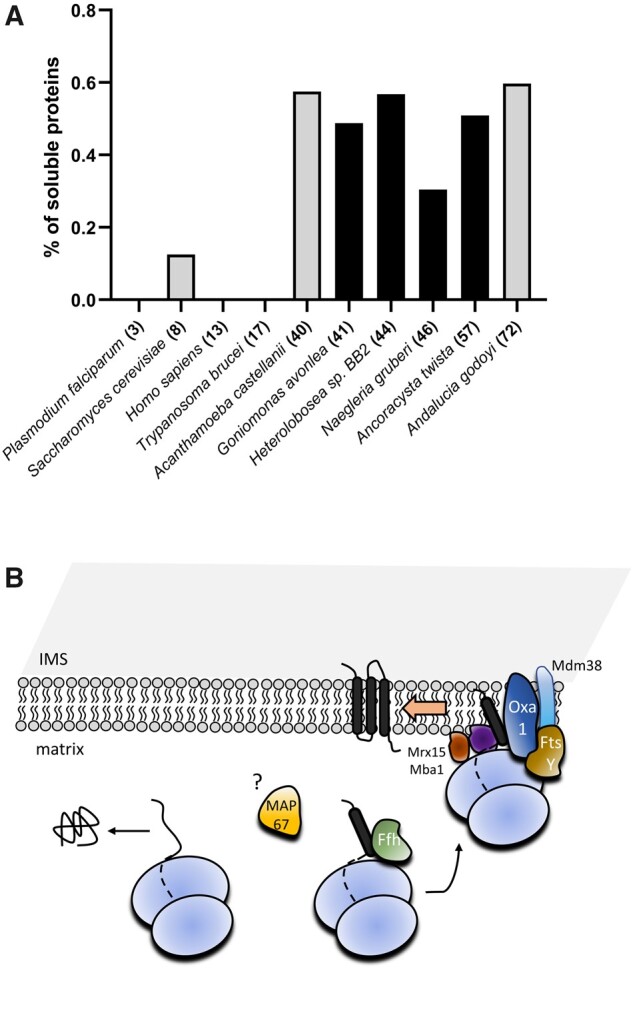
Persistence of the SRP pathway in mitochondria. (*A*) The mtSRP pathway is present only in organisms with a high proportion of soluble proteins encoded in the mitogenome. The *Y*-axis represents the percentage of proteins without any predicted transmembrane domain. Numbers in brackets show the total number of protein-coding genes in the mitogenome. Black color highlights organisms where the mtSRP pathway is present. (*B*) Cartoon representation of the putative mtSRP pathway as deduced from the results presented in this study (compare with fig. 1*D*). Names of the proteins displayed in white represent proteins present in all mtSRP system-possessing taxa, whereas proteins in black are present only in some of them.

Preservation of the mitochondrial SRP system in just a handful of eukaryotic lineages raises the question as to whether they share another feature that predetermined them to keep mtFfh and mtFtsY. Since the morphology and lifestyle of the mtFfh-/mtFtsY-containing eukaryotes vary widely, these provide no clue. It is, however, noticeable that they have gene-rich mitogenomes (yet to be confirmed for Hemimastogophora) with a significant fraction of genes encoding soluble proteins, such as mitoribosomal subunits (fig. 7*A*). It is therefore tempting to speculate that in these organisms, stable tethering of mitoribosomes to the MIM, a situation described in yeast, human, and other eukaryotes with few if any soluble mitochondrial-encoded proteins, would not provide sufficient flexibility to the translation apparatus. Although the proportion of membrane-associated and free mitoribosomes in eukaryotes with gene-rich mitogenomes remains unknown, it is plausible that the ratio will be shifted toward the later state. In such a case, the mitochondrial SRP system would provide a means to flexibly regulate the submitochondrial localization of the translating mitoribosome, depending on the nature of the nascent protein. However, this interpretation does not explain the absence of the mitochondrial SRP system from other protists with gene-rich mitogenomes, including the jakobid *Andalucia godoyi* (Burger et al. 2013), which has retained in its organelle a range of other ancestral bacteria-like traits (Gray et al. 2020).

Interestingly, it has been previously shown that the yeast mitoribosome interacts with a bacterial Ffh when this is expressed in yeast cells and engineered to be targeted to the mitochondrion (Funes et al. 2013), suggesting that the structural prerequisites for the function of the mitochondrial SRP system have been preserved even in lineages that lost it a long time ago. In addition, expression of a mitochondrion-targeted bacterial Ffh in the yeast *Δmba1 oxa1ΔC* strain (i.e., a mutant lacking the *mba1* gene and expressing a truncated version of Oxa1 without the C-terminal ribosome-binding tail) partially rescued the growth defects conferred by the mutations (Funes et al. 2013), which would be compatible with the idea that the ancestral mitochondrial SRP system and the Mba1-driven mitochondrion-specific mechanisms of ribosome membrane association are at least partially functionally redundant. However, the rescue effect of the bacterial Ffh did not depend on the presence of a bacterial FtsY in the yeast mutant (Funes et al. 2013), raising the question about the actual biochemical mechanisms of the Ffh action in the yeast mitochondrion. In this context, it is notable that our comparative genomic and phylogenetic analyses revealed broad conservation of proteins involved in the mitoribosome–MIM association in eukaryotes, including the (putative) mitoribosome receptors Mba1/mL45, Mdm38/LETM1, and Mrx15/TMEM223, as well as the C-terminal extension of Oxa1. Their distribution is consistent with the notion that they could have mediated the mitoribosome–MIM association already in the LECA. Meanwhile, the presence of these proteins in the mtFfh-/mtFtsY-carrying protists suggests that in these eukaryotic lineages, the original SRP-dependent mechanism of protein targeting has coexisted with the newly evolved mechanisms of mitoribosome–MIM association for approximately 1.5 billion years (Betts et al. 2018) and hence is unlikely to be functionally redundant with them.

Furthermore, we identified MAP67, a novel mitochondrial protein that also occurs broadly in eukaryotes and was most likely already present in the LECA. Its obvious evolutionary relationship to Ffh raises the possibility that it is a highly modified ortholog of mtFfh. However, the four mtFfh-bearing lineages are interspersed among taxa with MAP67 and at least one of them, the genus *Goniomonas*, harbors both genes. Therefore, we propose that MAP67 and mtFfh coexisted in early eukaryotes and their current distribution reflects extensive differential loss. Unfortunately, MAP67 is not sufficiently similar to the M domains of Ffh/SRP54 to make a conventional phylogenetic analysis meaningful, but the most parsimonious explanation of its origin is that it emerged from a duplicated copy of mtFfh by an internal deletion that removed its N and G domains. Presently, we can only speculate about the function of MAP67, but it has already been shown to be essential in two model apicomplexans, *T. gondii* (TGGT1_254230; Sidik et al. 2016) and *Plasmodium falciparum* (PF3D7_1004900; https://plasmodb.org/plasmo/, last accessed March 31, 2021). As it represents a divergent version of the Ffh/SRP54 M domain responsible for binding the signal peptide (Janda et al. 2010), it may still bind the N-terminal regions of mitochondrial proteins with characteristics of a signal peptide and mediate their membrane targeting. Such a role of MAP67 is further supported by the accretion, in Chloroplastida and Centrohelida, of two alternative C-terminal domains of SecA, a bacterial protein unknown from mitochondria that is involved in posttranslational membrane protein targeting (Steinberg et al. 2018). One more piece of evidence for our hypothesis was provided by a recent cryo-EM study of the structure of the mitoribosome from the ciliate *Tetrahymena thermophila* that detected a novel protein, denoted mL105, associated with the mitoribosome tunnel (Tobiasson and Amunts 2020). The authors noticed homology of mL105 to the M domain of Ffh and proposed that it may be involved in protein targeting in the mitochondrion. Unsurprisingly, our inspection of the *T. thermophila* mL105 protein (TTHERM_000931898) identified it as an MAP67 ortholog.

In conclusion, with the identification of mtFfh and mtFtsY, we have unveiled a novel mitochondrial attribute that joins the growing list of components present in the proto-mitochondrial endosymbiont but is retained only by marginal extant eukaryotic groups. We predict that with further exploration of the protist diversity, the reconstructed complexity of the mitochondrial cenancestor and its bacterial character will further increase. Somewhat surprisingly, the mitochondrial SRP system seems to be absent from a group where its presence was suspected based on the previous knowledge, namely the mitochondrial SecY-containing jakobids. The apparently nonoverlapping distribution of the mitochondrial SecY (or possibly a full SecYEG translocon) and the mtSRP system is puzzling and cannot be readily explained without functional characterization of both elements. These uncertainties notwithstanding, we hypothesize that protein targeting in certain extant mitochondria relies on a modified SRP-dependent pathway (fig. 7*B*) and may represent an impediment for translocation of the corresponding mitochondrial genes into the nuclear genome in the respective eukaryote lineages. Furthermore, our discovery of the broadly occurring MAP67 family that likely evolved from an mtFfh paralog suggests that vestiges of the SRP pathway in mitochondria may not be restricted to the mtFfh-/mtFtsY-carrying taxa. Direct experimental studies of MAP67 in appropriate model systems are necessary to establish its exact role in mitochondrial biology and to understand why MAP67 was lost from many eukaryotes, including metazoans and fungi.

## Materials and Methods

### Identification of mtFfh and mtFtsY Sequences

In order to identify homologs of mitochondrial SRP pathway in other eukaryotes outside the genus *Naegleria*, we performed a phylogeny-directed search for close homologs of its two protein components (mtFfh and mtFtsY) in publicly available databases. Using *Naegleria* sequences as a query, we collected 500 best tblastn hits from NCBI Transcriptome Shotgun Assembly (TSA) database, 2000 best tblastn hits from The Marine Microbial Eukaryote Transcriptome Sequencing Project (MMETSP), and approximately 400 sequences from the NCBI nonredundant protein database (100 best BlastP hits from each of the following: unclustered Archaea, Eubacteria, Eukaryota, and from clustered database “nr70”). Transcripts obtained from the MMETSP and NCBI TSA databases were translated into proteins using the TransDecoder utility (Haas et al. 2013). We additionally searched with tblastn transcriptome assemblies from various poorly studied protist lineages that were reported in the literature but are not included in the NCBI database; these were downloaded from the specific public repositories or were obtained upon request from the authors. In several cases, the sequences of special interest that were found truncated were extended by iterative manual blastn searches and recruitment of raw unassembled RNAseq reads available in the SRA database at NCBI. A partial FtsY transcript from *A. twista* was assembled similarly, starting from a seed read identified by an iterative tblastn search against the respective database of RNAseq reads. Some current gene models in genome annotations proved inaccurate and were manually corrected using evidence from transcriptome data and/or comparison with conserved regions in homologs. Ffh and FtsY homologs were also identified in our unpublished genome sequence assembly from the heterolobosean *Neovahlkampfia damariscottae* and manually annotated to define the exon–intron structure of the respective genes. All protein sequences were aligned with MAFFT version 7, using the auto mode (Katoh and Standley 2016) and trimmed manually. A preliminary phylogenetic analysis was performed in RAxML version 8.2.11 (Stamatakis 2014) under the simple PROTCATLG model with 100 rapid bootstraps. Sequences branching in the vicinity mtFtsY and mtFfh were retained for further analyses together with representative sequences from the archaeal, eubacterial, eukaryotic, and plastidial SRP54, Ffh, FtsY, and SRα proteins. Subsequently, several rounds of reciprocal Blastp and phylogenetic analyses were performed to remove contaminants and to add homologs from undersampled lineages. All mtFtsY and mtFfh sequences are listed in supplementary table S1, Supplementary Material online.

### Analyses of Protein Sequences

Subcellular targeting of candidate proteins was predicted by using TargetP-1.1 (Emanuelsson et al. 2007; http://www.cbs.dtu.dk/services/TargetP-1.1/index.php, last accessed March 31, 2021), MitoFates (Fukasawa et al. 2015; http://mitf.cbrc.jp/MitoFates/cgi-bin/top.cgi; prediction model: metazoa, last accessed March 31, 2021), MitoProt (Claros and Vincens 1996; https://ihg.gsf.de/ihg/mitoprot.html, last accessed March 31, 2021), and Predotar (Small et al. 2004; https://urgi.versailles.inra.fr/predotar/; animal or fungal sequences, last accessed March 31, 2021). TargetP was also used for the prediction of signal peptides. TMHMM tool (Krogh et al. 2001; http://www.cbs.dtu.dk/services/TMHMM/, last accessed March 31, 2021) served for the detection of transmembrane domains. Sensitive homology detection tools were employed to search for homologs of proteins of interest that evolve too rapidly to be always detectable across distant relationships by using Blastp (Oxa1, Mba1/mL45, Mrx15/TMEM223, MAP67). The HMMER3 package (Eddy 2011) was used to search a locally maintained protein sequence database (combining data protein sequences downloaded from public resources or inferred from nucleotide sequence data) in parallel to the recently reported EukProt database (Richter et al. 2020). The searches employed as queries profile HMMs built based on seed multiple protein sequence alignments downloaded from the Pfam database (El-GebAli et al. 2019) or custom alignments of previously identified reference sequences prepared with MAFFT. Where appropriate or needed, profile HMMs were iteratively updated by expanding the template alignments with new homologs recognized in the previous search. HMMER searches of the NCBI nr database were carried out using a public server (https://toolkit.tuebingen.mpg.de/tools/hmmer, last accessed March 31, 2021). The identity of the hits was assessed by backward Blastp searches against the NCBI nr database, conserved domain (CD) searches against the NCBI Conserved Domain Database (Yang et al. 2020), and by HHpred searches (Zimmermann et al. 2018; https://toolkit.tuebingen.mpg.de/tools/hhpred, last accessed March 31, 2021). The later searches were initiated either with individual reference query sequences with the default maximal three Multiple sequence alignment (MSA) generation steps utilizing HHblits, or multiple prealigned sequences with no extra MSA generation step. Four databases of profile HMMs—PDB_mmCIF30, COG_KOG, Pfam-A, and NCBI_CDs—were searched at once. Homology of MAP67 (using the sequence from the presumably slowly evolving malawimonad *Gefionella okellyi*) was also investigated by using the fold recognition server Phyre2 (Kelley et al. 2015).

### SRP RNA Analysis Using Covariance Models

Alignments of small bacterial SRP RNA (RF00169), large bacterial SRP RNA (RF01854), and protozoan signal recognition particle RNA (RF01856) were downloaded from the Rfam database (Kalvari et al. 2018; http://rfam.xfam.org/clan/CL00003, last accessed March 31, 2021) and processed using tools of the Infernal package version 1.1.2 (Nawrocki and Eddy 2013). Particularly, cmbuild was used to build a covariance model; *E*-value parameters for covariance models were calibrated by cmcalibrate, and cmsearch was used in combination with a particular calibrated model to screen available mtDNAs from Heterolobosea (*Pharyngomonas kirbyi*, Heterolobosea sp. BB2, *Stachyamoeba lipophora*, *Naegleria* spp., *Acrasis kona*, *N. damariscottae*), *A. twista*, and *G. avonlea* as well as nuclear genome assemblies from three *Naegleria* spp., *G. avonlea*, and our unpublished genomic data from *N. damariscottae*.

### Phylogenetic Analyses

In an attempt to evaluate the phylogenetic position and robustness of the phylogenetic placement of identified mitochondrial FtsY and Ffh proteins, we performed a set of phylogenetic analyses using sequences of signal recognition–associated GTPase family identified and collected by phylogeny-directed search (see above). We prepared four taxonomically balanced data sets. Specifically, the broad data set representing the diversity of the whole signal recognition–associated GTPase family (452 taxa) and three more focused data sets: FtsY-only data set containing eubacterial and organellar FtsY sequences (154 operational taxonomic units, or OTUs); FtsY/SRα data set (217 OTUs), and Ffh/SRP54 data set (295 OTUs). Protein sequences were aligned with MAFFT version 7 (Katoh and Standley 2016), using the G-INS-i method with BLOSUM30 scoring matrix and unalignlevel 0.8 (the broad data set) or unalignlevel 0.0 (FtsY-only data set) or the L-INS-i method with BLOSUM30 (Ffh/SRP54 and FtsY/SR data set). Alignments were trimmed manually (FtsY-only data set) or automatically (other data sets) using BMGE version 1.12 (Criscuolo and Gribaldo 2010) with adjusted parameters: BLOSUM30 matrix to estimate entropy-like value for each position; length of selected blocks at least two; maximum gap rate per position 0.6 or 0.8. Divvier 1.0 (Ali et al. 2019) under standard divvying setting was used to remove low confidence homologies from the broad data set before trimming.

Maximum likelihood (ML) phylogenetic analyses were carried out with IQ-TREE multicore version 1.6.10 (Hoang et al. 2018) and RAxML version 8.2.11 (Stamatakis 2014) under the LG4X substitution model suggested by ModelFinder (Kalyaanamoorthy et al. 2017). Branch supports were estimated by using three approaches: ultrafast bootstrapping with an activated “bnni” option to reduce the risk of overestimating branch supports (IQ-TREE, *N* = 1,000), rapid bootstrapping (*N* = 500, RAxML), and in case of Ffh/SRP54 also with nonparametric bootstrapping (*N* = 400, IQ-TREE). All bootstrap replicates were mapped on the best IQ-TREE topology using the “sup” option; final trees were visualized with CorelDRAW Home & Student Suite X8. For the ML phylogenetic analysis of the FtsY-only data set (supplementary fig. S4, Supplementary Material online), the AU test (Shimodaira 2002) was performed as implemented in the IQ-TREE multicore version 1.6.10 to evaluate two hypotheses for the phylogenetic origin of the mitochondrial and plastidial FtsY: mtFtsY branching with sequences from alphaproteobacteria and cpFtsY branching with homologs from cyanobacteria, respectively. The AU tests were conducted with hypothetical groupings (loosely constrained) under the LG4X model. The optimized trees were compared with 10,000 resamplings using the RELL method. Each hypothesis was tested in triplicate to show the consistency of the results. Maximum log-likelihoods (logL) of each constraint and replicate, as well as their differences from the unconstrained ML tree (deltaL) are listed in supplementary table S3, Supplementary Material online. The hypotheses within the 95% confidence interval that could not be rejected are those with *P*-AU ≥0.05.

### Cell Cultivation, Cloning, and Expression

*T. brucei* procyclic cell line SMOX 927 (Poon et al. 2012) was grown at 27 °C in SDM79 medium (Schönenberger 1979), whereas *N. gruberi* strain NEG-M (ATCC 30224) was grown axenically at 27 °C in M7 medium (Fulton 1974). Both media were supplemented with 10% fetal bovine serum. The N-terminal region of the mitochondrial-encoded genes from *N. gruberi* were codon-optimized for the expression in *T. brucei* (https://eu.idtdna.com/CodonOpt, last accessed March 31, 2021) and designed as a partially overlapping opposing long primers, which served both as a template and as primers in one cycle PCR. Analogously, N-terminal regions of PDI and NADPH-cytochrome p450 reductase from *T. brucei* were used as a positive and specificity control, respectively. This led to the synthesis of inserts up to 180 bp in length, which were along with the full-length CDS or N-terminal regions corresponding to the predicted mitochondrial signals of the NgFfh and NgFtsY individually subcloned in pT7 plasmid (Shaner et al. 2013) modified by insertion of the mNg gene in front of the V5 tag. The plasmid was linearized with *Not*I restriction enzyme and nucleofected into the *T. brucei* procyclic stage as described earlier (Kaurov et al. 2018). Expression of the proteins was induced with doxycycline overnight or for just a few hours, as was the case of full-length NgFfh and NgFtsY.

### Immunofluorescence Microscopy

*Trypanosoma brucei* procyclic cells were harvested, washed twice (900 g, 5 min at room temperature [RT]) with Voorheis’s-modified phosphate-buffered saline (vPBS; PBS supplemented with 10 mM glucose and 46 mM sucrose, pH 7.6) and the cell suspension was transferred on a microscopic slide covered with poly-l-lysine. Attached cells were fixed for 15 min with 4% paraformaldehyde at RT. Afterward, the cells were permeabilized with 0.1% Triton X-100 in PBS for 15 min. Blocking was performed for 1 h in 1% bovine serum albumine (BSA) in PBS supplemented with 0.033% Triton-X-100 and the same buffer (but without BSA) was also used for all washing steps. The expressed proteins were visualized using rabbit α-V5 antibody (Sigma–Aldrich), with α-mtHsp70 and α-TbBiP antibodies (Bangs et al. 1993; Panigrahi et al. 2008) used as mitochondrial and ER markers, respectively. Goat α-rabbit Alexa Fluor 488 and goat α-mouse Alexa Fluor 555 (both Life Technologies) were used as secondary antibodies. DNA was stained with ProLong1 Gold antifade reagent with 4′,6-diamidine-2′-phenylindole dihydrochloride (DAPI) (Molecular Probes), and stained cells were observed with Zeiss microscope Axioplan 2 equipped with an Olympus DP73 digital camera. Images were processed using the Fiji software and Pearson correlation coefficient for signals from different channels was calculated using the Coloc 2 plugin with default settings (Schindelin et al. 2012).

## Supplementary Material

Supplementary data are available at *Molecular Biology and Evolution* online.

## Supplementary Material

msab090_Supplementary_Data
