# Supplementary material for: Vestiges of the Bacterial Signal Recognition Particle-Based Protein Targeting in Mitochondria
Source: Mol Biol Evol. 2021 Apr 10;38(8):3170–87. doi: 10.1093/molbev/msab090 (PMC8321541; doi:10.1093/molbev/msab090)
Supplement: msab090_Supplementary_Data [file msab090_supplementary_data.zip › Supplementary_dataset_S1.docx]

**Phylogenetic tree from Fig. 3A (IQ-tree, LG4X model 1000 ultrafast bootstraps with bnni, 452 OTUs)**

(bacFtsY@WP_015110998.1_Nostoc_sp [Cyanobacteria]:0.1025984609,bacFtsY_WP_111893930.1_signal_recognition_particle-docking_protein_FtsY_Arthrospira_sp._O9.13F:0.0527173097,(bacFtsY_WP_081705397.1_signal_recognition_particle-docking_protein_FtsY_Gloeobacter_kilaueensis:0.2568356808,((bacFtsY_RCL55122.1_signal_recognition_particle-docking_protein_FtsY_Synechococcus_sp._MED-G71:0.0448549555,(bacFtsY_WP_038650415.1_signal_recognition_particle-docking_protein_FtsY_Prochlorococcus_sp._MIT_0801:0.1760676884,chrFtsY@AUG32399.1_Paulinella_longichromatophora [Rhizaria]:0.0963701072)100:0.0802887116)100:0.1326013457,(((((((((((((((bacFtsY@RPG16414.1_FtsY_Phycisphaera_sp_TMED9 [Planctomycetes]:0.2864780294,bacFtsY@RMH26650.1_Planctomycetes_bacterium [Planctomycetes]:0.3439239877)100:0.2312223985,bacFtsY@WP_088252003.1_Fimbriiglobus_ruber [Planctomycetes]:0.3219749054)96:0.1386561251,bacFtsY@OGC84905.1_Zixibacteria_bacterium_RBG_16_43_9 [Zixibacteria]:0.2276498895)78:0.0726322688,bacFtsY@EKD41878.1_uncultured_bacterium [unassigned]:0.5993001546)35:0.0412297152,((bacFtsY@WP_011985037.1_Anaeromyxobacter_sp_Fw109-5 [Deltaproteobacteria]:0.3531131526,((bacFtsY@WP_041017695.1_Criblamydia_sequanensis [Chlamydiae]:0.4360265773,bacFtsY@PCI95412.1_Candidatus_Aerophobetes_bacterium [Aerophobetes]:0.3831890118)98:0.1528045470,((ptFtsY@MMETSP0308_Transcript_18196_m19230_Gloeochaete_wittrockiana [Archaeplastida]:0.4168218150,((((ptFtsY@PRW61060.1_Chlorella_sorokiniana_[Archaeplastida]:0.0989835481,(ptFtsY@XP_005651149.1_Coccomyxa_subellipsoidea [Archaeplastida]:0.1357035185,ptFtsY@XP_001697752.1_Chlamydomonas_reinhardtii [Archaeplastida]:0.2882107950)57:0.0916475997)51:0.0167867868,ptFtsY@XP_011399720.1_Auxenochlorella_protothecoides [Archaeplastida]:0.2591420351)56:0.0551313024,ptFtsY@XP_003080532.1_Ostreococcus_tauri [Archaeplastida]:0.3123453027)98:0.0427821822,((((ptFtsY@KHF98418.1_Cell_division_FtsY_chloroplastic-like_protein_Gossypium_arboreum [Archaeplastida]:0.0124224043,ptFtsY@XP_010055217.1_Eucalyptus_grandis [Archaeplastida]:0.0578982624)99:0.0158260357,ptFtsY@XP_023879170.1_Quercus_suber [Archaeplastida]:0.0000026059)100:0.0115382814,ptFtsY@XP_021817084.1_Prunus_avium [Archaeplastida]:0.0411167419)99:0.0082245058,ptFtsY@NP_566056.1_Arabidopsis_thaliana [Archaeplastida]:0.0453747730)100:0.2036819403)100:0.1427343266)100:0.0795041016,((((ptFtsY@CEM32712.1_Vitrella_brassicaformis_CCMP3155 [Alveolata]:0.2652361364,((ptFtsY@EWM29383.1_Nannochloropsis_gaditana [Stramenopiles]:0.2381242447,ptFtsY@CBJ31918.1_Ectocarpus_siliculosus [Stramenopiles]:0.2857588280)85:0.0747701329,ptFtsY@XP_009040860.1_Aureococcus_anophagefferens [Stramenopiles]:0.2110574421)67:0.0229378222)80:0.0441190325,ptFtsY@XP_002296627.1_Thalassiosira_pseudonana_CCMP1335 [Stramenopiles]:0.3782484368)100:0.0696040469,(ptFtsY@XP_005714040.1_Chondrus_crispus [Archaeplastida]:0.0606149504,ptFtsY@PXF44704.1_Gracilariopsis_chorda [Archaeplastida]:0.1035320926)100:0.2529377807)100:0.0827539383,(ptFtsY@XP_005537370.1_Cyanidioschyzon_merolae_strain_10D [Archaeplastida]:0.4726988290,ptFtsY@XP_005705405.1_Galdieria_sulphuraria [Archaeplastida]:0.6033162802)93:0.0640650942)100:0.2140127657)98:0.0983403179)48:0.0497017864)40:0.0000028448,(((bacFtsY@WP_056203513.1_Pelomonas_sp_Root1237 [Betaproteobacteria]:0.0312405088,bacFtsY@PZP35629.1_Roseateles_depolymerans [Betaproteobacteria]:0.0256580532)78:0.0259789877,((bacFtsY@WP_124447410.1_Paucibacter_sp_KBW04 [Betaproteobacteria]:0.0550779484,(bacFtsY@OWQ45088.1_Mitsuaria_noduli [Betaproteobacteria]:0.0283250051,bacFtsY@WP_066336977.1_Azohydromonas_lata [Betaproteobacteria]:0.0721091710)56:0.0134363411)55:0.0213106927,bacFtsY@WP_089417744.1_Vitreoscilla_filiformis [Betaproteobacteria]:0.0778083643)95:0.0189510130)100:0.3992745477,bacFtsY@OYZ20489.1_Bdellovibrio_sp_28-41-41 [Deltaproteobacteria]:0.5310603280)72:0.1085071336)43:0.0286810520)19:0.0261450815,((((((((((bacFtsY@WP_107510123.1_Staphylococcus_fleurettii [Firmicutes]:0.0886370051,bacFtsY@AVK83142.1_Lysinibacillus_sp_B2A1 [Firmicutes]:0.1100561878)100:0.0654563141,(bacFtsY@WP_009554695.1_Lactobacillus_saerimneri [Firmicutes]:0.0983202637,(bacFtsY@WP_103423367.1_Lactobacillus_sanfranciscensis [Firmicutes]:0.0936393745,bacFtsY@WP_056961480.1_Lactobacillus_florum [Firmicutes]:0.1378236570)100:0.2029132721)100:0.1340204560)97:0.0855891884,(bacFtsY@WP_069327418.1_Paenibacillus_sp_TI45-13ar [Firmicutes]:0.1043515089,(bacFtsY@WP_091834879.1_Marininema_halotolerans [Firmicutes]:0.1670002371,bacFtsY@WP_028778316.1_Shimazuella_kribbensis [Firmicutes]:0.0920569648)95:0.0635472870)91:0.0570913585)91:0.0282749925,bacFtsY@PTQ57904.1_Candidatus_Carbobacillus_altaicus [Firmicutes]:0.2653737421)86:0.0503844997,bacFtsY@WP_026974763.1_Alicyclobacillus_contaminans [Firmicutes]:0.3463228752)98:0.0584502201,bacFtsY@WP_073092242.1_Thermosyntropha_lipolytica [Firmicutes]:0.3361513912)79:0.0288119894,(bacFtsY@WP_072972532.1_Tissierella_praeacuta [Firmicutes]:0.2158475803,bacFtsY@WP_054252106.1_Neofamilia_massiliensis [Firmicutes]:0.3670846799)90:0.1105261729)29:0.0053983557,bacFtsY@CDA51269.1_Clostridium_sp_CAG-138 [Firmicutes]:0.3342189844)17:0.0546105037,bacFtsY@EEG77220.1_Dethiobacter_alkaliphilus_AHT_1 [Firmicutes]:0.2413766442)16:0.0482681508,bacFtsY@KUO52399.1_Desulfitibacter_sp_BRH_c19 [Firmicutes]:0.3344039832)86:0.0343839939)68:0.0398801024,((((((bacFtsY@OJX59420.1_Candidatus_Kapabacteria_thiocyanatum [Bacteroidetes]:0.1031630516,bacFtsY@PKL79980.1_Ignavibacteriae_bacterium_HGW-Ignavibacteriae-4 [Ignavibacteriae]:0.1852289410)100:0.0986049867,bacFtsY@KXK57805.1_Chlorobi_bacterium_OLB7 [Chlorobi]:0.1499824753)100:0.0611364655,bacFtsY@PLX30570.1_Ignavibacteria_bacterium [Ignavibacteriae]:0.1651753225)81:0.0142764934,(bacFtsY@WP_092350764.1_Candidatus_Chrysopegis_kryptomonas [Kryptonia]:0.1184523446,((bacFtsY@OGU83728.1_Ignavibacteria_bacterium_RBG_16_35_7 [Ignavibacteriae]:0.4052251937,bacFtsY@PKL82841.1_Ignavibacteriae_bacterium_HGW-Ignavibacteriae-3 [Ignavibacteriae]:0.3663777629)100:0.0864530170,bacFtsY@OQY74580.1_Ignavibacteriales_bacterium_UTCHB3 [Ignavibacteriae]:0.2121315607)100:0.1573995642)96:0.0570816626)90:0.0455197281,bacFtsY@OGU26318.1_Ignavibacteria_bacterium_GWA2_54_16 [Ignavibacteriae]:0.3262523400)96:0.0234272119,(((((bacFtsY@PSQ63899.1_Bacteroidetes_bacterium_QH_1_61_8 [Bacteroidetes]:0.2118029803,bacFtsY@PSR05731.1_Bacteroidetes_bacterium_SW_10_40_5 [Bacteroidetes]:0.2449919350)98:0.0711412944,(bacFtsY@WP_103327589.1_Bacteroidetes_endosymbiont_of_Geopemphigus_sp [Bacteroidetes]:0.1601222108,bacFtsY@WP_114910387.1_Cardinium_endosymbiont_of_Sogatella_furcifera [Bacteroidetes]:0.3089126086)98:0.1220302185)96:0.0266935792,bacFtsY@OUV32974.1_Rhodothermaceae_bacterium_TMED105 [Bacteroidetes]:0.3045918403)47:0.0238173460,(bacFtsY@WP_100314957.1_Thermoflavifilum_aggregans [Bacteroidetes]:0.1608759628,bacFtsY@OUU18192.1_Crocinitomicaceae_bacterium_TMED45 [Bacteroidetes]:0.2654664305)95:0.0736095700)100:0.0845296127,((((bacFtsY@WP_031499580.1_Bryobacter_aggregatus [Acidobacteria]:0.1590684517,bacFtsY@ANM28936.1_Acidobacteria_bacterium_Mor1 [Acidobacteria]:0.4934924954)19:0.0422922272,(bacFtsY@WP_121469822.1_Edaphobacter_dinghuensis [Acidobacteria]:0.2079413497,bacFtsY@WP_081490725.1_Terriglobus_roseus [Acidobacteria]:0.0814469649)100:0.3701083276)78:0.0473594308,bacFtsY@PYS67814.1_Acidobacteria_bacterium [Acidobacteria]:0.2990114722)94:0.0935975770,bacFtsY@PIE91415.1_Acidobacteria_bacterium [Acidobacteria]:0.3161560915)100:0.1121591079)68:0.0320589610)84:0.0787352217)80:0.0315029102,((bacFtsY@OGP30931.1_Deltaproteobacteria_bacterium_GWC2_42_11 [Deltaproteobacteria]:0.2855648834,((bacFtsY@GBD43248.1_Bacterium_HR40 [unassigned]:0.3217984911,((bacFtsY@KRS17267.1_Roseovarius_indicus [Alphaproteobacteria]:0.0503175650,bacFtsY@WP_025048799.1_Sulfitobacter_mediterraneus [Alphaproteobacteria]:0.0355861916)100:0.1705220739,((((bacFtsY@WP_108880431.1_Anderseniella_sp_Alg231-50 [Alphaproteobacteria]:0.2312906069,bacFtsY@WP_109793879.1_Rhizobiales_bacterium [Alphaproteobacteria]:0.1813153349)98:0.0560252691,((((bacFtsY@SKC16052.1_Bosea_thiooxidans [Alphaproteobacteria]:0.1342273530,(bacFtsY@WP_024277252.1_Xanthobacter_sp_126 [Alphaproteobacteria]:0.1031758743,bacFtsY@WP_013168132.1_Starkeya_novella [Alphaproteobacteria]:0.1018687678)100:0.0590256664)100:0.0554728420,(bacFtsY@WP_088520031.1_Rhodoblastus_acidophilus [Alphaproteobacteria]:0.1521217858,bacFtsY@WP_115516251.1_Pseudolabrys_sp_GY_H [Alphaproteobacteria]:0.2022607218)98:0.0485579046)69:0.0256323554,bacFtsY@WP_099557887.1_Hartmannibacter_diazotrophicus [Alphaproteobacteria]:0.0922687307)24:0.0000022382,(((bacFtsY@WP_113333951.1_Rhizobiales_bacterium [Alphaproteobacteria]:0.1160154268,bacFtsY@RCL01761.1_Candidatus_Tokpelaia_sp_JSC085 [Alphaproteobacteria]:0.2508403670)100:0.0960727988,bacFtsY@PPD07903.1_Hyphomicrobium_sp [Alphaproteobacteria]:0.2000958593)54:0.0414273035,bacFtsY@WP_029041189.1_Cucumibacter_marinus [Alphaproteobacteria]:0.1274795464)27:0.0300748915)24:0.0507028742)97:0.1012515846,(bacFtsY@ACT57804.1_Hirschia_baltica_ATCC_49814 [Alphaproteobacteria]:0.0000028572,bacFtsY@WP_083773102.1_Hirschia_baltica [Alphaproteobacteria]:0.0000021088)100:0.3385256967)55:0.0472961632,(((bacFtsY@EPY01636.1_Phaeospirillum_fulvum_MGU-K5 [Alphaproteobacteria]:0.1259588989,bacFtsY@OFX10022.1_Alphaproteobacteria_bacterium_RIFOXYD12_FULL_60_8 [Alphaproteobacteria]:0.1298757310)99:0.0533401045,bacFtsY@WP_092615803.1_Roseospirillum_parvum [Alphaproteobacteria]:0.1949219340)98:0.0287316367,(bacFtsY@OUU28495.1_Candidatus_Endolissoclinum_sp_TMED37 [Alphaproteobacteria]:0.2122478022,bacFtsY@AIL12880.1_Candidatus_Paracaedimonas_acanthamoebae [Alphaproteobacteria]:0.3941037615)92:0.0841448883)92:0.0594867055)73:0.0448674645)79:0.0472218559)100:0.1278152029,bacFtsY@SME87908.1_Pseudobacteriovorax_antillogorgiicola [Deltaproteobacteria]:0.4084845966)100:0.1231814066)96:0.0841433606,(bacFtsY@WP_012175138.1_Desulfococcus_oleovorans [Deltaproteobacteria]:0.2894452082,bacFtsY@OLB22504.1_Nitrospirae_bacterium_13_2_20CM_2_63_8 [Nitrospirae]:0.5352526313)83:0.0527564728)71:0.0256637972)76:0.0235224622,(((bacFtsY@AEG15826.1_Desulfofundulus_kuznetsovii_DSM_6115 [Firmicutes]:0.1513786944,(bacFtsY@WP_075860076.1_Carboxydothermus_pertinax [Firmicutes]:0.2847319448,bacFtsY@WP_009108227.1_Desulfovibrio_sp_U5L [Deltaproteobacteria]:0.4075900087)99:0.0634841534)98:0.0554320894,((bacFtsY@PIP06279.1_Syntrophobacteraceae_bacterium_CG23_combo_of_CG06-09_8_20_14_all_50_8 [Deltaproteobacteria]:0.1474561858,bacFtsY@ABC77884.1_Syntrophus_aciditrophicus_SB [Deltaproteobacteria]:0.1524975929)100:0.1319223200,(bacFtsY@PIE60181.1_Desulfobulbus_propionicus [Deltaproteobacteria]:0.2553070336,bacFtsY@WP_083764053.1_Syntrophobacter_fumaroxidans [Deltaproteobacteria]:0.3025330107)95:0.0584216780)93:0.0394823322)89:0.0592770893,((bacFtsY@WP_038038136.1_Thermorudis_peleae [Chloroflexi]:0.3463364257,(bacFtsY@OQY47814.1_Anaerolineaceae_bacterium_4572_78 [Chloroflexi]:0.3500109677,bacFtsY@KUK71287.1_Anaerolineae_bacterium_49_20 [Chloroflexi]:0.4816277022)88:0.1020719652)97:0.0362742274,(((((((((((((eukSRa@MMETSP0308_Transcript_27849_m29213_Gloeochaete_wittrockiana [Archaeplastida]:0.1436011459,eukSRa@ANM86232.1_Stygiella_incarcerata [Jakobida]:0.2429392750)88:0.0557236582,(((((eukSRa@NA_Hemimastix_kukwesjijk [Hemimastigophora]:0.2065952100,eukSRa@NP_001171313.1_Homo_sapiens [Opisthokonta]:0.2327632440)88:0.0192038177,(eukSRa@XP_010703301.1_Leishmania_panamensis [Euglenozoa]:0.0451203710,(eukSRa@EKF29134.1_Trypanosoma_cruzi_marinkellei [Euglenozoa]:0.0449299803,eukSRa@EPY25999.1_Angomonas_deanei [Euglenozoa]:0.0421748536)92:0.0271434014)100:0.3348917730)92:0.0504848864,(((((eukSRa@XP_005848975.1_Chlorella_variabilis [Archaeplastida]:0.0547499571,((eukSRa@XP_002949444.1_Volvox_carteri_nagariensis [Archaeplastida]:0.0160582931,eukSRa@KXZ55716.1_Gonium_pectorale [Archaeplastida]:0.0165739712)100:0.0178847623,eukSRa@XP_001692081.1_Chlamydomonas_reinhardtii [Archaeplastida]:0.0151959151)100:0.0955978018)100:0.0584409011,eukSRa@OUS44851.1_Ostreococcus_tauri [Archaeplastida]:0.3671548463)93:0.0246933921,eukSRa@CAMPEP_0191492328_Pyramimonas-parkeae-CCMP726 [Archaeplastida]:0.1641193531)93:0.0107206966,(((eukSRa@ONL92607.1_Zea_mays [Archaeplastida]:0.0237691834,(((eukSRa@XP_021634617.1_Manihot_esculenta [Archaeplastida]:0.0000028122,eukSRa@XP_003527179.1_Glycine_max [Archaeplastida]:0.0000028122)96:0.0000025681,eukSRa@XP_022750268.1_Durio_zibethinus [Archaeplastida]:0.0113129978)100:0.0062985799,eukSRa@XP_020873914.1_Arabidopsis_lyrata_subsp_lyrata [Archaeplastida]:0.0282073842)99:0.0109695792)99:0.0221110907,eukSRa@XP_024403926.1_Physcomitrella_patens [Archaeplastida]:0.1015919987)99:0.0335318293,eukSRa@GBG74478.1_Chara_braunii [Archaeplastida]:0.0492441081)99:0.0307459820)100:0.1109291285,(eukSRa@GECH01003431.1_Pharyngomonas_kirbyi [Heterolobosea]:0.1869766168,(((eukSRa@CEL66130.1_Neospora_caninum [Alveolata]:0.0056935469,eukSRa@ESS33331.1_Toxoplasma_gondii [Alveolata]:0.0119827517)100:0.1843937279,eukSRa@SBT01585.1_Plasmodium_malariae [Alveolata]:0.1984072250)99:0.0464835029,(eukSRa@XP_004029895.1_Ichthyophthirius_multifiliis [Alveolata]:0.2568792593,eukSRa@CAMPEP_0170537274_Litonotus_pictus_Strain_P1 [Alveolata]:0.2411399635)100:0.0274040211)99:0.0864518144)93:0.0311181041)79:0.0614280516)25:0.0133491588,(eukSRa@GEZU01029986.1_GEZU01002276.1_Heterolobosea_BB2 [Heterolobosea]:0.1025790813,((eukSRa@XP_002670075.1_Naegleria_gruberi [Heterolobosea]:0.0000026045,eukSRa@NF0122080_p1_Naegleria_fowleri [Heterolobosea]:0.0403735378)100:0.1785479521,(eukSRa@CAMNT_0005228041_Percolomonas_cosmopolitus_strain_WS [Heterolobosea]:0.1519249591,eukSRa@CAMNT_0005204115_Percolomonas_cosmopolitus_AE [Heterolobosea]:0.3491696836)100:0.1337679026)98:0.0790208999)71:0.0765367606)6:0.0180031746,((eukSRa@GGUN01044729.1_Goniomonas_avonlea [Cryptophyta]:0.0678297839,eukSRa@CAMNT_0031806435_Goniomonas_pacifica [Cryptophyta]:0.0351240073)100:0.1841129723,eukSRa@GFYU01005410.1_Ancoracysta_twista [Ancoracysta]:0.1264963016)80:0.0535632017)6:0.0114831031)17:0.0299622706,(eukSRa@NA_Neovahlkampfia_damariscottae [Heterolobosea]:0.1826048489,(eukSRa@KYQ94416.1_Tieghemostelium_lacteum [Amoebozoa]:0.1013791494,eukSRa@XP_012757500.1_Acytostelium_subglobosum_LB1 [Amoebozoa]:0.0992353661)100:0.1596751589)94:0.0633106411)90:0.0461393122,eukSRa@KOO32508.1_Chrysochromulina_sp_CCMP291 [Haptophyta]:0.2603812634)19:0.0426507971,(eukSRa@POM83525.1_Cryptosporidium_meleagridis [Alveolata]:0.2308413474,eukSRa@GBE59094.1_Babesia_ovata [Alveolata]:0.2249541625)99:0.0835832420)14:0.0155027034,((((eukSRa@RLN51388.1_Phytophthora_kernoviae [Stramenopiles]:0.0128016245,eukSRa@XP_002906671.1_Phytophthora_infestans_T30-4 [Stramenopiles]:0.0151147999)100:0.0539432802,eukSRa@GAX97406.1_Pythium_insidiosum [Stramenopiles]:0.0645669090)100:0.1251013252,eukSRa@XP_005853460.1_Nannochloropsis_gaditana_CCMP526 [Stramenopiles]:0.1629716670)74:0.0439991431,eukSRa@CBJ30645.1_Ectocarpus_siliculosus [Stramenopiles]:0.1340370627)100:0.1297428618)97:0.1109143308,eukSRa@XP_012894318.1_Blastocystis_hominis [Stramenopiles]:0.1883957903)99:0.1640721226,eukSRa@AAD11975.1_Giardia_intestinalis [Metamonada]:0.5360540237)100:0.5300567342,(archFtsY@OIO41360.1_Candidatus_Pacearchaeota_archaeon_CG1_02_31_27 [DPANN]:0.3337497696,archFtsY@PIZ51778.1_Candidatus_Woesearchaeota_archaeon_CG_4_10_14_0_2_um_filter_33_13 [DPANN]:0.2588238671)100:0.2078710112)93:0.0783009139,(archFtsY@WP_048122758.1_Methanosarcina_vacuolata [Euryarchaeota]:0.2039499697,((archFtsY@WP_004045232.1_Haloferax_volcanii [Euryarchaeota]:0.0583386851,((archFtsY@WP_021073086.1_Haloarchaeon_3A1_DGR [Euryarchaeota]:0.0527255162,archFtsY@WP_050034148.1_Halorubrum_halophilum [Euryarchaeota]:0.0814083373)100:0.0672965566,archFtsY@WP_114604860.1_Haloplanus_sp._CBA1112 [Euryarchaeota]:0.0990098246)100:0.0585720381)99:0.0265076407,archFtsY@WP_006182963.1_Natrinema_pellirubrum [Euryarchaeota]:0.1410897231)100:0.2838565180)100:0.1172996381)93:0.0273334179,archFtsY@RLI30149.1_Candidatus_Bathyarchaeota_archaeon [TACK]:0.3261141927)89:0.0373397204,(archFtsY@RLI57948.1_Candidatus_Thorarchaeota_archaeon [Asgard]:0.3353831606,((archFtsY@WP_014122151.1_Thermococcus_sp._AM4 [Euryarchaeota]:0.0998650837,archFtsY@WP_011012907.1_Pyrococcus_furiosus [Euryarchaeota]:0.0804651321)100:0.24

3910077,(archFtsY@RMF91339.1_Euryarchaeota_archaeon [Euryarchaeota]:0.3516141671,archFtsY@OYT54953.1_Candidatus_Altiarchaeales_archaeon_ex4484_2 [DPANN]:0.2707848378)99:0.1381516516)97:0.0455205456)95:0.0498099165)100:0.4263682240,(((((((((((((ptFfh@MMETSP0308_Transcript_33397_m34971_Gloeochaete_wittrockiana [Archaeplastida]:0.4236869707,(((((((ptFfh@XP_016738451.1_Gossypium_hirsutum [Archaeplastida]:0.0146289372,(ptFfh@XP_024442952.1_Populus_trichocarpa [Archaeplastida]:0.0512892952,ptFfh@NP_196014.1_Arabidopsis_thaliana [Archaeplastida]:0.0303318869)96:0.0148553949)96:0.0033479142,(ptFfh@XP_007204322.1_Prunus_persica [Archaeplastida]:0.0267997201,ptFfh@XP_023898971.1_Quercus_suber [Archaeplastida]:0.0243099074)86:0.0173465329)100:0.1158813334,ptFfh@ABR16458.1_Picea_sitchensis [Archaeplastida]:0.0496095179)100:0.0800911865,ptFfh@XP_002964409.2_Selaginella_moellendorffii [Archaeplastida]:0.1492101886)98:0.0600374425,(ptFfh@A9RGM4_Physcomitrella_patens_subsp_patens [Archaeplastida]:0.0000020886,ptFfh@XP_024383502.1_Physcomitrella_patens [Archaeplastida]:0.0000022214)100:0.0650450156)98:0.0683848297,ptFfh@GAQ87100.1_Klebsormidium_nitens [Archaeplastida]:0.1757713995)98:0.0652309086,(((ptFfh@A0A2P6TNJ4_Chlorella_sorokiniana [Archaeplastida]:0.0736878135,ptFfh@XP_011401759.1_Auxenochlorella_protothecoides [Archaeplastida]:0.2430473300)100:0.0919873662,ptFfh@I0YJE8_Coccomyxa_subellipsoidea_strain_C-169 [Archaeplastida]:0.1307309361)85:0.0216966976,(ptFfh@A0A061R4M7_Tetraselmis_sp_GSL018 [Archaeplastida]:0.2385275436,ptFfh@GAX85777.1_Chlamydomonas_eustigma [Archaeplastida]:0.1912316013)85:0.0395018870)87:0.0518454226)90:0.1032461382)98:0.0585189060,(((ptFfh@GFZU01021206.1_Laurencia_pacifica [Archaeplastida]:0.1118898630,ptFfh@XP_005717012.1_Chondrus_crispus [Archaeplastida]:0.0797530287)100:0.0215365399,ptFfh@PXF44869.1_Gracilariopsis_chorda [Archaeplastida]:0.1186481012)100:0.1446126878,((ptFfh@OEU13328.1_Fragilariopsis_cylindrus_CCMP1102 [Stramenopiles]:0.1869622424,((ptFfh@GAX28389.1_Fistulifera_solaris [Stramenopiles]:0.1163489060,ptFfh@XP_002179613.1_Phaeodactylum_tricornutum_CCAP_1055-1 [Stramenopiles]:0.1105110507)100:0.0835931260,ptFfh@XP_002287312.1_Thalassiosira_pseudonana_CCMP1335 [Stramenopiles]:0.1595216252)100:0.0645365197)100:0.1336213089,ptFfh@CBN76263.1_Ectocarpus_siliculosus [Stramenopiles]:0.3064345950)99:0.0730396768)100:0.1144113306)97:0.0998596498,((((chrFfh@AQX44624.1_Paulinella_micropora [Rhizaria]:0.0110622517,chrFfh@AXY63015.1_Paulinella_micropora [Rhizaria]:0.0000025323)100:0.0285835520,chrFfh@AUG32274.1_Paulinella_longichromatophora [Rhizaria]:0.0325410418)100:0.0476265563,chrFfh@YP_002048787.1_Paulinella_chromatophora [Rhizaria]:0.0590809029)100:0.0517334205,((bacFfh@WP_011618707.1_Synechococcus_sp._CC9311 [Cyanobacteria]:0.0279621821,(bacFfh@WP_087068640.1_Cyanobium_sp_NIES-981 [Cyanobacteria]:0.0268580182,bacFfh@WP_106220726.1_Aphanothece_minutissima [Cyanobacteria]:0.0643584198)100:0.0470053094)81:0.0214034261,bacFfh@WP_063414566.1_Prochlorococcus_marinus [Cyanobacteria]:0.0443576394)98:0.0469506796)100:0.1900687350)91:0.0465849460,((((bacFfh@WP_011612100.1_Trichodesmium_erythraeum [Cyanobacteria]:0.0895351360,bacFfh@0A2P8WK82_Filamentous_cyanobacterium_CCP5 [Cyanobacteria]:0.0758635637)56:0.0275808412,((bacFfh@WP_009785362.1_Lyngbya_sp_PCC_8106 [Cyanobacteria]:0.0851583778,(bacFfh@A8YEA3_Microcystis_aeruginosa_PCC_7806 [Cyanobacteria]:0.0654954862,bacFfh@KFF41240.1_Candidatus_Atelocyanobacterium_thalassa_isolate_SIO64986 [Cyanobacteria]:0.1770505229)100:0.0687195620)87:0.0237037111,bacFfh@PSP17129.1_Cyanobacteria_bacterium_QS_8_64_29 [Cyanobacteria]:0.2033778491)30:0.0267293738)13:0.0050794440,bacFfh@WP_081914739.1_Leptolyngbya_sp [Cyanobacteria]:0.0715818126)33:0.0280483473,(bacFfh@WP_015124913.1_Synechococcus_sp_PCC_6312 [Cyanobacteria]:0.0923393422,bacFfh@RMH67925.1_Cyanobacteria_bacterium_J003 [Cyanobacteria]:0.0551635237)94:0.0288535713)98:0.0570236934)88:0.0383817888,bacFfh@WP_023172070.1_Gloeobacter_kilaueensis [Cyanobacteria]:0.1716661477)99:0.0774197992,bacFfh@WP_011429947.1_Synechococcus_sp [Cyanobacteria]:0.0678769712)100:0.2011265362,bacFfh@OJX56912.1_Candidatus_Kapabacteria_thiocyanatum [Bacteroidetes]:0.2358353409)100:0.0691774680,(bacFfh@PIQ24328.1_Candidatus_Blackallbacteria [unassigned]:0.2059122374,bacFfh@PCJ61345.1_Planctomycetes_bacterium [Planctomycetes]:0.3119859157)100:0.0931958374)99:0.0597012688,(((bacFfh@OPX24629.1_Candidatus_Latescibacteria_bacterium_4484_107 [Bacteroidetes]:0.2650265233,bacFfh@WP_006928829.1_Caldithrix_abyssi [Calditrichaeota]:0.1893840446)100:0.0664371325,bacFfh@EFH87465.1_Ktedonobacter_racemifer_DSM_44963 [Chloroflexi]:0.3726692964)93:0.0876470318,(bacFfh@WP_049675438.1_Desulfocarbo_indianensis [Deltaproteobacteria]:0.2389121237,(((bacFfh@WP_102742496.1_Akkermansia_muciniphila [Verrucomicrobia]:0.0051673575,bacFfh@CDD93459.1_Akkermansia_sp_CAG_344 [Verrucomicrobia]:0.0186752443)100:0.1584612398,(bacFfh@WP_035603039.1_Haloferula_sp_BvORR071 [Verrucomicrobia]:0.1997167562,bacFfh@WP_105044656.1_Rubritalea_profundi [Verrucomicrobia]:0.1513866978)100:0.0420935462)100:0.1375510678,bacFfh@WP_006980240.1_Chthoniobacter_flavus [Verrucomicrobia]:0.1992059422)100:0.2477440089)98:0.0590083099)88:0.0332920511)94:0.0706773807,(((((((((((((bacFfh@WP_086120482.1_Lactobacillus_reuteri [Firmicutes]:0.1884664187,bacFfh@WP_050441216.1_Streptococcus_pneumoniae [Firmicutes]:0.2454715662)100:0.0550261346,bacFfh@WP_015076211.1_Carnobacterium_maltaromaticum [Firmicutes]:0.1319189656)100:0.0622369720,bacFfh@PKL00150.1_Tenericutes_bacterium_HGW-Tenericutes-1 [Tenericutes]:0.3197073246)100:0.0713758947,(bacFfh@WP_117520369.1_Ruminococcus_sp_AF43-11 [Firmicutes]:0.1704486085,bacFfh@WP_022786100.1_Clostridiales_bacterium_NK3B98 [Firmicutes]:0.2835839623)100:0.0638664942)100:0.0466837837,bacFfh@WP_058486324.1_Defluviitalea_phaphyphila [Firmicutes]:0.1527045697)75:0.0247749301,((bacFfh@WP_008908195.1_Caloramator_australicus [Firmicutes]:0.1242000599,bacFfh@WP_074910001.1_Proteiniclasticum_ruminis [Firmicutes]:0.2079498398)100:0.0648507078,bacFfh@WP_054875147.1_Oxobacter_pfennigii [Firmicutes]:0.1568205679)100:0.0626580740)65:0.0177513093,(bacFfh@CDC00866.1_Firmicutes_bacterium_CAG_41 [Firmicutes]:0.2356886285,bacFfh@WP_072469189.1_Urinacoccus_massiliensis [Firmicutes]:0.2329311965)75:0.0453173062)99:0.0367534233,(bacFfh@KJS18981.1_Clostridiaceae_bacterium_BRH_c20a [Firmicutes]:0.1570690200,bacFfh@PWM50529.1_Clostridiales_bacterium [Firmicutes]:0.1643874776)100:0.0648111954)97:0.0258332998,(((bacFfh@WP_015891890.1_Brevibacillus_brevis [Firmicutes]:0.1004375434,bacFfh@WP_120461224.1_Paenibacillus_aceti [Firmicutes]:0.1364783779)98:0.0352856867,(((bacFfh@WP_109984449.1_Gracilibacillus_dipsosauri [Firmicutes]:0.1469420693,bacFfh@WP_116552983.1_Pueribacillus_theae [Firmicutes]:0.0882488249)100:0.1045910135,bacFfh@WP_007505027.1_Caldalkalibacillus_thermarum [Firmicutes]:0.0781187646)100:0.0373727998,bacFfh@WP_089967246.1_Lihuaxuella_thermophila [Firmicutes]:0.1469110086)96:0.0286173319)95:0.0352372659,bacFfh@WP_038091674.1_Tumebacillus_flagellatus [Firmicutes]:0.0972464100)100:0.0501768282)97:0.0286618692,bacFfh@WP_018702437.1_Anaeromusa_acidaminophila [Firmicutes]:0.1140851774)94:0.0338050030,(bacFfh@WP_046498292.1_Syntrophomonas_zehnderi [Firmicutes]:0.1541763570,bacFfh@WP_106004935.1_Moorella_humiferrea [Firmicutes]:0.1524340455)100:0.0430187806)65:0.0380064751,(((bacFfh@WP_072905435.1_Anaerobranca_californiensis [Firmicutes]:0.1659615886,bacFfh@OPL10543.1_Firmicutes_bacterium_ML8_F2 [Firmicutes]:0.2841670430)100:0.0429466028,bacFfh@WP_109430892.1_Acidibacillus_sulfuroxidans [Firmicutes]:0.2516774973)98:0.0461931715,bacFfh@WP_013275867.1_Thermosediminibacter_oceani [Firmicutes]:0.1521948513)53:0.0225933889)93:0.0430969579,(bacFfh@ACX52518.1_Ammonifex_degensii_KC4 [Firmicutes]:0.1400820621,bacFfh@WP_054491650.1_Ardenticatena_maritima [Chloroflexi]:0.2379964503)67:0.0541068813)61:0.0216271176)66:0.0385379934,((((((bacFfh@WP_033187470.1_Pseudoalteromonas_sp_PLSV [Gammaproteobacteria]:0.1084017411,(bacFfh@WP_078744295.1_Oceanospirillum_multiglobuliferum [Gammaproteobacteria]:0.0782373225,bacFfh@WP_039914025.1_Cellvibrio_mixtus [Gammaproteobacteria]:0.1335427754)100:0.0345980392)100:0.0703286321,bacFfh@WP_024496304.1_Candidatus_Schmidhempelia_bombi [Gammaproteobacteria]:0.1246428134)96:0.0209272763,(bacFfh@WP_114137085.1_Klebsiella_pneumoniae [Gammaproteobacteria]:0.0502566358,bacFfh@RJL31521.1_Pectobacterium_polaris [Gammaproteobacteria]:0.0552333683)100:0.0594288248)96:0.0428759712,bacFfh@WP_091713482.1_Methylophaga_sulfidovorans [Gammaproteobacteria]:0.1416848959)97:0.0448912081,bacFfh@WP_025769383.1_Thioalkalivibrio_sp_HK1 [Gammaproteobacteria]:0.2084042074)100:0.1800633423,(((((((((((bacFfh@WP_109920731.1_Zavarzinia_compransoris [Alphaproteobacteria]:0.2021373005,(bacFfh@PCI43326.1_Alphaproteobacteria_bacterium [Alphaproteobacteria]:0.2156284164,((bacFfh@WP_099557780.1_Hartmannibacter_diazotrophicus [Alphaproteobacteria]:0.1624925998,bacFfh@WP_018634118.1_Neomegalonema_perideroedes [Alphaproteobacteria]:0.1688837118)94:0.0444855290,((bacFfh@OJT95039.1_Alphaproteobacteria_bacterium_65-7 [Alphaproteobacteria]:0.1472084041,bacFfh@OQW59094.1_Proteobacteria_bacterium_HN_bin10 [Proterobacteria]:0.3308007785)94:0.0775172699,(bacFfh@WP_116392064.1_Parvularcula_sp_SM1705 [Alphaproteobacteria]:0.2578358552,bacFfh@RCL81099.1_SAR116_cluster_bacterium [Alphaproteobacteria]:0.2695424187)59:0.0448998091)91:0.0417821513)81:0.0162830189)82:0.0157626862)84:0.0334363937,(((((bacFfh@GBE42717.1_Bacterium_BMS3Bbin10 [unassigned]:0.1654748845,bacFfh@WP_038035438.1_Thermopetrobacter_sp_TC1 [Alphaproteobacteria]:0.2161740395)6:0.0247425717,(bacFfh@WP_111197444.1_Rhizobiales_bacterium_KCTC_52945 [Alphaproteobacteria]:0.0987834711,bacFfh@WP_085770433.1_Methylocystis_bryophila [Alphaproteobacteria]:0.2889858099)43:0.0361962123)23:0.0206360885,bacFfh@WP_013420482.1_Rhodomicrobium_vannielii [Alphaproteobacteria]:0.1266991934)53:0.0118728594,(bacFfh@PCI85686.1_Rhizobiales_bacterium [Alphaproteobacteria]:0.0000023276,bacFfh@PCJ00993.1_OCS116_cluster_bacterium [Alphaproteobacteria]:0.0038417285)100:0.2209766304)90:0.0263674930,(bacFfh@OUU83527.1_Hyphomicrobiaceae_bacterium_TMED74 [Alphaproteobacteria]:0.1496680387,(bacFfh@PKQ09217.1_Alphaproteobacteria_bacterium_HGW-Alphaproteobacteria-12 [Alphaproteobacteria]:0.1054791362,bacFfh@RCL83452.1_PS1_clade_bacterium [Alphaproteobacteria]:0.2936841811)96:0.0551262635)91:0.0381067286)92:0.0504217132)93:0.0208783907,(((((((((((bacFfh@OJX70273.1_Magnetospirillum_sp_64-120 [Alphaproteobacteria]:0.0387358380,bacFfh@WP_024081694.1_Magnetospirillum_gryphiswaldense [Alphaproteobacteria]:0.0228796481)100:0.0304798305,bacFfh@WP_068497751.1_Magnetospirillum_moscoviense [Alphaproteobacteria]:0.0123984208)100:0.0715182669,(bacFfh@WP_002727862.1_Phaeospirillum_molischianum [Alphaproteobacteria]:0.0569402550,bacFfh@WP_068493492.1_Magnetospirillum_marisnigri [Alphaproteobacteria]:0.0469393363)55:0.0229310012)100:0.1024110830,((bacFfh@WP_073953247.1_Thalassospira_sp_TSL5-1 [Alphaproteobacteria]:0.1748987559,bacFfh@WP_028877797.1_Terasakiella_pusilla [Alphaproteobacteria]:0.1368266771)97:0.0424343504,(bacFfh@WP_043360684.1_Belnapia_sp_F-4-1 [Alphaproteobacteria]:0.1213527017,bacFfh@WP_034336672.1_Commensalibacter_sp_MX01 [Alphaproteobacteria]:0.2500029720)100:0.0912076896)93:0.0153566464)76:0.0309054696,(bacFfh@WP_019645789.1_Novispirillum_itersonii [Alphaproteobacteria]:0.1033475822,(bacFfh@WP_092613925.1_Roseospirillum_parvum [Alphaproteobacteria]:0.1893426111,((bacFfh@CDB39986.1_Azospirillum_sp_CAG_260 [Alphaproteobacteria]:0.0393066130,bacFfh@CDB53938.1_Azospirillum_sp_CAG_239 [Alphaproteobacteria]:0.0393233239)100:0.2160181674,bacFfh@OFX07559.1_Alphaproteobacteria_bacterium_RIFOXYD12_FULL_60_8 [Alphaproteobacteria]:0.1337495008)98:0.0572102436)70:0.0344721630)62:0.0313607025)92:0.0320797032,bacFfh@WP_041795188.1_Pararhodospirillum_photometricum [Alphaproteobacteria]:0.1711881203)83:0.0292780880,bacFfh@CCZ21287.1_Acetobacter_sp_CAG_977 [Alphaproteobacteria]:0.1702199914)84:0.0402723788,(bacFfh@WP_069956818.1_Magnetovibrio_blakemorei [Alphaproteobacteria]:0.1472851052,bacFfh@OUT52366.1_Rhodospirillaceae_bacterium_TMED8 [Alphaproteobacteria]:0.2191824853)76:0.0776201169)47:0.0127537994,bacFfh@PHY00942.1_Rhodospirillaceae_bacterium [Alphaproteobacteria]:0.2736118044)78:0.0388848346,(((bacFfh@OIN86659.1_Alphaproteobacteria_bacterium_CG1_02_46_17 [Alphaproteobacteria]:0.1508487312,bacFfh@PZQ45682.1_Micavibrio_aeruginosavorus [Alphaproteobacteria]:0.1075980834)100:0.0663148676,(bacFfh@PCJ00252.1_Alphaproteobacteria_bacterium [Alphaproteobacteria]:0.2094209011,bacFfh@WP_015467793.1_Micavibrio_aeruginosavorus [Alphaproteobacteria]:0.1499717187)100:0.0303971399)100:0.0517875856,bacFfh@OUX71121.1_Rhodospirillaceae_bacterium_TMED140 [Alphaproteobacteria]:0.1967852754)93:0.0441099451)43:0.0051762913,bacFfh@WP_092823393.1_Rhodospirillales_bacterium_URHD0017 [Alphaproteobacteria]:0.2181525797)48:0.0193904205)45:0.0139952425,((bacFfh@WP_012973141.1_Azospirillum_lipoferum [Alphaproteobacteria]:0.0426301272,(bacFfh@WP_094454746.1_Niveispirillum_lacus [Alphaproteobacteria]:0.1699786772,bacFfh@WP_028466142.1_Nisaea_denitrificans [Alphaproteobacteria]:0.1673505140)48:0.0340745157)51:0.0238725453,bacFfh@WP_027287498.1_Rhodovibrio_salinarum [Alphaproteobacteria]:0.2129010448)90:0.0657452863)50:0.0052381118,(bacFfh@WP_046506348.1_Kiloniella_litopenaei [Alphaproteobacteria]:0.1213763874,bacFfh@WP_119283627.1_Rhodospirillaceae_bacterium_SYSU_D60006 [Alphaproteobacteria]:0.1324157591)91:0.0531429741)58:0.0229072459,(((bacFfh@OUR76855.1_Alphaproteobacteria_bacterium_46_93_T64 [Alphaproteobacteria]:0.0703317678,bacFfh@WP_025896931.1_Sneathiella_glossodoripedis [Alphaproteobacteria]:0.0258605458)100:0.1178110213,(((((((bacFfh@SCW52489.1_Sphingobium_faniae [Alphaproteobacteria]:0.0409198313,bacFfh@WP_010335520.1_Sphingobium_yanoikuyae [Alphaproteobacteria]:0.0288542390)99:0.0279108773,bacFfh@WP_014076705.1_Sphingobium_sp_SYK-6 [Alphaproteobacteria]:0.0771452654)97:0.0324971502,bacFfh@WP_089215287.1_Sphingopyxis_indica [Alphaproteobacteria]:0.1040507703)43:0.0348010366,bacFfh@WP_022691338.1_Sphingomonas-like_bacterium_B12 [Alphaproteobacteria]:0.1020050335)43:0.0276479665,bacFfh@WP_116091360.1_Sphingomonas_crusticola [Alphaproteobacteria]:0.0860273339)77:0.0383678272,bacFfh@WP_011240910.1_Zymomonas_mobilis [Alphaproteobacteria]:0.1617219488)89:0.0550787625,bacFfh@WP_072596811.1_Sphingomonas_sp_JJ-A5 [Alphaproteobacteria]:0.1018437942)100:0.1226601732)72:0.0516078894,bacFfh@WP_115937331.1_Aestuariispira_insulae [Alphaproteobacteria]:0.1334550233)64:0.0214606251)16:0.0037915925,bacFfh@PCJ58386.1_Rhodospirillaceae_bacterium [Alphaproteobacteria]:0.2762243242)83:0.0446822999,bacFfh@PPR12333.1_Alphaproteobacteria_bacterium_MarineAlpha11_Bin1 [Alphaproteobacteria]:0.1802437299)81:0.0246876092,(bacFfh@PPR20700.1_Alphaproteobacteria_bacterium_MarineAlpha10_Bin2 [Alphaproteobacteria]:0.0836436521,bacFfh@PPR14985.1_Alphaproteobacteria_bacterium_MarineAlpha9_Bin3 [Alphaproteobacteria]:0.3710316682)97:0.0941163127)91:0.0302543215,((bacFfh@WP_014746762.1_Tistrella_mobilis [Alphaproteobacteria]:0.1635316115,(bacFfh@OHC73580.1_Rhodospirillales_bacterium_RIFCSPLOWO2_02_FULL_58_16 [Alphaproteobacteria]:0.1712935772,bacFfh@PPR36214.1_Alphaproteobacteria_bacterium_MarineAlpha9_Bin6 [Alphaproteobacteria]:0.2443616780)100:0.0377808954)95:0.0434882468,(((((bacFfh@OJX13986.1_Caedibacter_sp_37-49 [Alphaproteobacteria]:0.1860034235,(bacFfh@WP_085783826.1_Candidatus_Nucleicultrix_amoebiphila [Alphaproteobacteria]:0.1222522339,bacFfh@OJV16027.1_Alphaproteobacteria_bacterium_33-17 [Alphaproteobacteria]:0.3791040130)100:0.0665643444)98:0.0491536772,bacFfh@OYZ36327.1_Alphaproteobacteria_bacterium_16-39-46 [Alphaproteobacteria]:0.2216077172)94:0.0321257608,(bacFfh@WP_032113236.1_Candidatus_Paracaedibacter_symbiosus [Alphaproteobacteria]:0.1962513492,((bacFfh@OUT75042.1_Rhizobiales_bacterium_TMED25 [Alphaproteobacteria]:0.1239525367,bacFfh@OUX67412.1_Rhizobiales_bacterium_TMED227 [Alphaproteobacteria]:0.0457330389)100:0.1894693788,(bacFfh@WP_025264366.1_Wolbachia_endosymbiont_of_Onchocerca_volvulus [Alphaproteobacteria]:0.1362008401,bacFfh@WP_065432712.1_Ehrlichia_ruminantium [

lphaproteobacteria]:0.2076753339)100:0.2343567933)100:0.0789069440)88:0.0357750438)91:0.0230052271,(bacFfh@PIR38902.1_Alphaproteobacteria_bacterium_CG11_big_fil_rev_8_21_14_0_20_39_49 [Alphaproteobacteria]:0.1628513648,(bacFfh@PLX30515.1_Alphaproteobacteria_bacterium [Alphaproteobacteria]:0.2508092806,(bacFfh@PDH20339.1_Pelagibacterales_bacterium_MED-G40 [Alphaproteobacteria]:0.4112351198,bacFfh@OUW71296.1_Rickettsiales_bacterium_TMED211 [Alphaproteobacteria]:0.3256927268)100:0.0857741038)100:0.0578797656)92:0.0716588458)86:0.0457477956,(bacFfh@KKB96089.1_Arcanobacter_lacustris [Alphaproteobacteria]:0.2448275500,((bacFfh@WP_027134478.1_Geminicoccus_roseus [Alphaproteobacteria]:0.2231879978,bacFfh@WP_088559973.1_Arboriscoccus_pini [Alphaproteobacteria]:0.1612733047)99:0.0773401070,bacFfh@PZP86081.1_Azospirillum_brasilense [Alphaproteobacteria]:0.2435483918)97:0.0491592339)81:0.0167528372)85:0.0390354067)84:0.0247213824)93:0.0617061554,(bacFfh@PPR79452.1_Alphaproteobacteria_bacterium_MarineAlpha2_Bin1 [Alphaproteobacteria]:0.3064535037,((((mtFfh@NA_Ancoracysta_twista [Ancoracysta]:0.2120931008,mtFfh@NA_Ancoracysta-related_Colp-4b [Ancoracysta]:0.2742728989)100:0.2523430643,(mtFfh@NA_Hemimastix_kukwesjijk [Hemimastigophora]:0.2739907367,mtFfh@NA_Spironema_sp [Hemimastigophora]:0.4675398927)100:0.4340449399)71:0.0628022979,((mtFfh@NA_Goniomonas_avonlea [Cryptophyta]:0.2312853963,mtFfh@NA_Goniomonas_pacifica [Cryptophyta]:0.5913046805)100:0.2375575113,mtFfh@NA_Percolomonas_cosmopolitus_strain_AE [Heterolobosea]:0.7347788359)99:0.0861153588)98:0.0642263586,(((mtFfh@NA_Neovahlkampfia_damariscottae [Heterolobosea]:0.3395979923,(mtFfh@NA_Naegleri_gruberi [Heterolobosea]:0.0971116724,(mtFfh@NA_Naegleria_fowleri [Heterolobosea]:0.0144322284,mtFfh@NA_Naegleria_lovaniensis [Heterolobosea]:0.0130392733)100:0.0374083488)100:0.3062422440)100:0.0945082900,mtFfh@NA_Heterolobosea_BB2 [Heterolobosea]:0.2026328470)100:0.0705992681,(mtFfh@NA_Pharyngomonas_kirbyi [Heterolobosea]:0.3563691974,(mtFfh@NA_Percolomonas_cosmopolitus_strain_WS [Heterolobosea]:0.2439086365,mtFfh@NA_Percolomonas_ex_Nitzchia_Cheng_2013 [Heterolobosea]:0.3887409726)100:0.4461774259)94:0.0838797135)99:0.1354998241)100:0.1376227864)99:0.0403180290)100:0.1038572917)100:0.1035202346)63:0.0207132480,bacFfh@OIP71264.1_Candidatus_Atribacteria_bacterium_CG2_30_33_13 [Atribacteria]:0.5595090024)100:0.2235916954,(((((((((((eukSRP54@MMETSP0308_Transcript_29267_m30521_Gloeochaete_wittrockiana [Archaeplastida]:0.2210528515,(eukSRP54@GGUN01039927.1_Goniomonas_avonlea [Cryptophyta]:0.0536238835,eukSRP54@CAMNT_0031843689_Goniomonas_pacifica [Cryptophyta]:0.0555038804)100:0.0565042300)89:0.0490712564,((eukSRP54@XP_004367866.1_Acanthamoeba_castellanii_strain_Neff [Amoebozoa]:0.1906478224,(((eukSRP54@XP_016608410.1_Spizellomyces_punctatus_DAOM_BR117 [Opisthokonta]:0.1100790112,(((eukSRP54@KNZ71807.1_Termitomyces_sp_J132 [Opisthokonta]:0.0243454187,eukSRP54@PBK96926.1_Armillaria_gallica [Opisthokonta]:0.0038127853)100:0.0131401431,eukSRP54@KXN86887.1_Leucoagaricus_sp_SymC_cos [Opisthokonta]:0.0198026280)100:0.0272528800,eukSRP54@RDX56745.1_Polyporus_brumalis [Opisthokonta]:0.0251725963)100:0.2954504890)99:0.0575524188,eukSRP54@EPZ32142.1_Rozella_allomycis_CSF55 [Opisthokonta]:0.1864678223)100:0.0526053765,((eukSRP54@XP_023304254.1_Lucilia_cuprina [Opisthokonta]:0.0111016350,((eukSRP54@SPP87160.1_Drosophila_guanche [Opisthokonta]:0.0000028804,eukSRP54@XP_017138354.1_Drosophila_miranda [Opisthokonta]:0.0000026200)100:0.0073741033,eukSRP54@XP_002068627.1_Drosophila_willistoni [Opisthokonta]:0.0073668870)96:0.0150022861)96:0.0150567708,(eukSRP54@ETN65735.1_Anopheles_darlingi [Opisthokonta]:0.0086558255,eukSRP54@XP_021695011.1_Aedes_aegypti [Opisthokonta]:0.0091226318)100:0.0316946871)100:0.1917723973)99:0.0436721973)64:0.0282810792,eukSRP54@NA_Spironema_sp [Hemimastigophora]:0.2975628732)54:0.0221928370)45:0.0146065044,eukSRP54@GFYU01001334.1_Ancoracysta_twista [Ancoracysta]:0.2043740084)18:0.0246117050,(eukSRP54@XP_024367976.1_Physcomitrella_patens [Archaeplastida]:0.0647589087,(eukSRP54@PON48078.1_Parasponia_andersonii [Archaeplastida]:0.0068370754,((eukSRP54@XP_022724150.1_Durio_zibethinus [Archaeplastida]:0.0038770339,eukSRP54@XP_008454178.1_Cucumis_melo [Archaeplastida]:0.0303082624)100:0.0074379509,eukSRP54@XP_006427317.1_Citrus_clementina [Archaeplastida]:0.0038874250)100:0.0157575235)100:0.0600853167)100:0.1317250341)78:0.0470789831,eukSRP54@NA_Neovahlkampfia_damariscottae [Heterolobosea]:0.2163972624)81:0.0486502623,((eukSRP54@ANM86233.1_Stygiella_incarcerata [Jakobida]:0.3088118423,((eukSRP54@XP_002671296.1-corrected_Naegleria_gruberi [Heterolobosea]:0.0604522423,eukSRP54@NF0102970_p1_Naegleria_fowleri [Heterolobosea]:0.0329418395)100:0.0956813944,eukSRP54@ACER01000537.1_Naegleri_gruberi [Heterolobosea]:0.4630053823)100:0.0998979020)99:0.0420019680,(eukSRP54@GEZU01011656.1_Heterolobosea_BB2 [Heterolobosea]:0.1619750460,eukSRP54@GECH01013817.1_Pharyngomonas_kirbyi [Heterolobosea]:0.2227353726)98:0.0477305999)98:0.0383525269)93:0.0501756585,(eukSRP54@XP_001018396.1_Tetrahymena_thermophila_SB210 [Alveolata]:0.0794846203,eukSRP54@XP_004039207.1_Ichthyophthirius_multifiliis [Alveolata]:0.1282018196)100:0.1667775611)95:0.0484904573,((eukSRP54@RAW42240.1_Phytophthora_cactorum [Stramenopiles]:0.0037292679,eukSRP54@XP_008893053.1_Phytophthora_parasitica_INRA-310 [Stramenopiles]:0.0000027192)100:0.1686318027,(eukSRP54@CBN75372.1_Ectocarpus_siliculosus [Stramenopiles]:0.1674880966,(eukSRP54@OEU17714.1_Fragilariopsis_cylindrus_CCMP1102 [Stramenopiles]:0.0976988536,eukSRP54@XP_002185775.1_Phaeodactylum_tricornutum_CCAP1055-1 [Stramenopiles]:0.1362292713)100:0.0628093352)100:0.0663978827)100:0.1139352088)97:0.0637981686,eukSRP54@CAMNT_0031816087_Goniomonas_pacifica [Cryptophyta]:0.4164249176)100:0.1760658708,(((((archSRP54@Q977V2_Haloferax_volcanii [Euryarchaeota]:0.0711933797,((archSRP54@WP_066299325.1_Haloterrigena_mahii [Euryarchaeota]:0.0128964461,archSRP54@WP_005578715.1_Natronobacterium_gregoryi [Euryarchaeota]:0.0563596201)80:0.0186211450,archSRP54@WP_049990021.1_Halopiger_salifodinae [Euryarchaeota]:0.0000024603)100:0.0998614517)69:0.0198067483,archSRP54@WP_092813433.1_Halopenitus_malekzadehii [Euryarchaeota]:0.0816621883)70:0.0175947462,archSRP54@WP_079234317.1_Halolamina_sp_CBA1230 [Euryarchaeota]:0.0827101816)100:0.3397124018,(((archSRP54@WP_095645771.1_Methanosarcina_spelaei [Euryarchaeota]:0.1165729830,archSRP54@KXS43658.1_Methanolobus_sp_T82-4 [Euryarchaeota]:0.1267870795)99:0.0254520811,archSRP54@WP_011449961.1_Methanospirillum_hungatei [Euryarchaeota]:0.3412095658)89:0.0294214644,archSRP54@WP_014405995.1_Methanocella_conradii [Euryarchaeota]:0.1389665047)89:0.0329386317)100:0.1463984672,archSRP54@RLF74017.1_Thermoplasmata_archaeon [Euryarchaeota]:0.3046173507)100:0.1250079598)69:0.0580354301,(((archSRP54@RLE51417.1_Candidatus_Verstraetearchaeota_archaeon [TACK]:0.1636159491,(archSRP54@RLF14065.1_Thermoprotei_archaeon [TACK]:0.2433861533,archSRP54@WP_110270916.1_Acidianus_brierleyi [TACK]:0.3698919172)100:0.0759340188)99:0.0692505348,archSRP54@OLS17826.1_Candidatus_Odinarchaeota_archaeon LCB4 [Asgard]:0.3111973709)99:0.0752128225,((archSRP54@WP_087037458.1_Thermococcus_litoralis [Euryarchaeota]:0.0474374276,(archSRP54@WP_062370762.1_Thermococcus_guaymasensis [Euryarchaeota]:0.0590421354,archSRP54@WP_088864111.1_Thermococcus_barossii [Euryarchaeota]:0.0488903580)100:0.0457057704)100:0.0280056213,archSRP54@WP_014734678.1_Pyrococcus_sp_ST04 [Euryarchaeota]:0.0523033859)100:0.1337012327)98:0.0352493703)100:0.3638609658)100:0.3580981479)100:0.2340158647)95:0.0423131955)96:0.0575174803)87:0.0847084207,((((((mtFtsY@NA_Naegleria_gruberi [Heterolobosea]:0.0000027799,(mtFtsY@NA_Naegleria_fowleri [Heterolobosea]:0.0000023565,mtFtsY@NA_Naegleria_lovaniensis [Heterolobosea]:0.0116783156)100:0.0623458047)100:0.2576153046,mtFtsY@NA_Neovahlkampfia_damariscottae [Heterolobosea]:0.5127439533)100:0.1488362420,(mtFtsY@CAMNT_0005246107_Percolomonas_cosmopolitus_WS [Heterolobosea]:0.2623043247,mtFtsY@NA_partial_Percolomonas_ex_Nitzchia_Cheng_2013 [Heterolobosea]:0.4508356941)100:0.2666347064)73:0.0878043285,(mtFtsY@NA_Heterolobosea_BB2 [Heterolobosea]:0.1390463611,mtFtsY@NA_Pharyngomonas_kirbyi [Heterolobosea]:0.3021223479)100:0.1670737950)76:0.1574497116,mtFtsY@NA_1_Hemimastix_kukwesjijk [Hemimastigophora]:0.3367216719)66:0.0884258613,(((mtFtsY@QUTJ01025227.1_Goniomonas_avonlea [Cryptophyta]:0.4452463709,mtFtsY@NA_partial_Goniomonas_pacifica [Cryptophyta]:0.8173922742)98:0.1692876250,mtFtsY@NA_N-terminus_Ancoracysta_twista [Ancoracysta]:0.3472174085)32:0.0000023154,mtFtsY@NA_Ancoracysta-related_Colp-4b [Ancoracysta]:0.5774698992)99:0.2134761026)99:0.1261469654)49:0.0000020087,(bacFtsY@PID38167.1_Proteobacteria_bacterium [Proterobacteria]:0.2915430885,bacFtsY@KPK53060.1_Myxococcales_bacterium_SG8_38_1 [Deltaproteobacteria]:0.4449080096)95:0.0859916245)96:0.0640578894,bacFtsY@WP_095208401.1_Luteimonas_sp_JM171 [Gammaproteobacteria]:0.2679230478)88:0.0559618104,bacFtsY@OUX68934.1_Oceanospirillales_bacterium_TMED91 [Gammaproteobacteria]:0.3636314980)96:0.0661322899,(((bacFtsY@WP_097790462.1_Halomonas_beimenensis [Verrucomicrobia]:0.1392603475,(((bacFtsY@WP_094041167.1_Zobellella_denitrificans [Gammaproteobacteria]:0.0598406532,bacFtsY@WP_091986549.1_Pseudoalteromonas_denitrificans [Gammaproteobacteria]:0.1301882136)100:0.0238144054,bacFtsY@WP_113743312.1_Anaerobiospirillum_thomasii [Gammaproteobacteria]:0.3004114568)100:0.0483112809,bacFtsY@PPI88525.1_Pantoea_sp_SoEO [Gammaproteobacteria]:0.4202835133)100:0.0926314051)66:0.0412600387,bacFtsY@ARM82543.1_Marinobacter_salarius [Gammaproteobacteria]:0.1370050663)72:0.0395822268,bacFtsY@WP_091826228.1_Marinobacterium_georgiense [Gammaproteobacteria]:0.0781038487)90:0.1095108334)98:0.0751077428,bacFtsY@WP_086487265.1_Thioflexothrix_psekupsii [Gammaproteobacteria]:0.0781158282)100:0.3822368027)100:0.0833218410)100:0.1144816081);

**Phylogenetic tree from Fig. 3B (IQ-tree, LG4X model 1000 ultrafast bootstraps with bnni, 295 OTUs)**

(mtFfh@NA_Ancoracysta_twista [Ancoracysta]:0.3240040741,mtFfh@NA_Ancoracysta-related_Colp-4b [Ancoracysta]:0.4322143688,(((((((((((((((chrFfh@AQX44624.1_Paulinella_micropora [Rhizaria]:0.0131911550,chrFfh@AXY63015.1_Paulinella_micropora [Rhizaria]:0.0036699011)100:0.036439,chrFfh@AUG32274.1_Paulinella_longichromatophora [Rhizaria]:0.0494518529)100:0.050312,chrFfh@YP_002048787.1_Paulinella_chromatophora [Rhizaria]:0.1160589258)100:0.099609,((bacFfh@WP_011618707.1_Synechococcus_sp._CC9311 [Cyanobacteria]:0.0427082268,bacFfh@WP_063414566.1_Prochlorococcus_marinus [Cyanobacteria]:0.0678173938)94:0.018354,(bacFfh@WP_087068640.1_Cyanobium_sp_NIES-981 [Cyanobacteria]:0.0568410700,bacFfh@WP_106220726.1_Aphanothece_minutissima [Cyanobacteria]:0.0676397628)97:0.034069)89:0.029141)100:0.238965,(((((((bacFfh@WP_011612100.1_Trichodesmium_erythraeum [Cyanobacteria]:0.1073360865,bacFfh@WP_009785362.1_Lyngbya_sp_PCC_8106 [Cyanobacteria]:0.1144374074)61:0.041491,bacFfh@WP_081914739.1_Leptolyngbya_sp [Cyanobacteria]:0.0879415777)59:0.019403,(bacFfh@0A2P8WK82_Filamentous_cyanobacterium_CCP5 [Cyanobacteria]:0.1189108173,bacFfh@PSP17129.1_Cyanobacteria_bacterium_QS_8_64_29 [Cyanobacteria]:0.2484135224)19:0.020361)18:0.022031,(bacFfh@A8YEA3_Microcystis_aeruginosa_PCC_7806 [Cyanobacteria]:0.0990813550,bacFfh@KFF41240.1_Candidatus_Atelocyanobacterium_thalassa_isolate_SIO64986 [Cyanobacteria]:0.2173374164)100:0.077455)95:0.026578,(bacFfh@WP_015124913.1_Synechococcus_sp_PCC_6312 [Cyanobacteria]:0.0949043301,bacFfh@RMH67925.1_Cyanobacteria_bacterium_J003 [Cyanobacteria]:0.0789530214)100:0.050463)100:0.082204,bacFfh@WP_023172070.1_Gloeobacter_kilaueensis [Cyanobacteria]:0.2173079209)90:0.035101,bacFfh@WP_011429947.1_Synechococcus_sp [Cyanobacteria]:0.2030727622)87:0.024112)97:0.074256,((((ptFfh@GFZU01021206.1_Laurencia_pacifica [Archaeplastida]:0.1904208104,ptFfh@XP_005717012.1_Chondrus_crispus [Archaeplastida]:0.1199700524)95:0.030951,ptFfh@PXF44869.1_Gracilariopsis_chorda [Archaeplastida]:0.1317768801)100:0.260840,((ptFfh@OEU13328.1_Fragilariopsis_cylindrus_CCMP1102 [Stramenopiles]:0.2051212950,((ptFfh@GAX28389.1_Fistulifera_solaris [Stramenopiles]:0.1017481967,ptFfh@XP_002179613.1_Phaeodactylum_tricornutum_CCAP_1055-1 [Stramenopiles]:0.1387927767)100:0.105040,ptFfh@XP_002287312.1_Thalassiosira_pseudonana_CCMP1335 [Stramenopiles]:0.1605004586)100:0.083827)100:0.171797,ptFfh@CBN76263.1_Ectocarpus_siliculosus [Stramenopiles]:0.4281104438)99:0.090004)100:0.114585,(((((((((((ptFfh@XP_016738451.1_Gossypium_hirsutum [Archaeplastida]:0.0296418884,ptFfh@NP_196014.1_Arabidopsis_thaliana [Archaeplastida]:0.0560688628)73:0.013908,((((((ptFfh@XP_023898971.1_Quercus_suber [Archaeplastida]:0.0217389420,(ptFfh@XP_007204322.1_Prunus_persica [Archaeplastida]:0.0208922101,ptFfh@XP_008391835.1_Malus_domestica [Archaeplastida]:0.0251432498)100:0.026105)55:0.008419,((ptFfh@XP_006341101.1_Solanum_tuberosum [Archaeplastida]:0.0156515273,ptFfh@XP_016448785.1_Nicotiana_tabacum [Archaeplastida]:0.0354023919)100:0.016195,ptFfh@XP_021977940.1_Helianthus_annuus [Archaeplastida]:0.0289471085)100:0.034355)91:0.013504,ptFfh@XP_024038087.1_Citrus_clementina [Archaeplastida]:0.0318194592)67:0.010666,ptFfh@PON78013.1_Parasponia_andersonii [Archaeplastida]:0.0202136371)42:0.002220,(((ptFfh@XP_003521470.1_Glycine_max [Archaeplastida]:0.0085298997,ptFfh@XP_017418457.1_Vigna_angularis [Archaeplastida]:0.0178178157)100:0.017419,(ptFfh@CCH47177.1_Lupinus_angustifolius [Archaeplastida]:0.0597351205,ptFfh@XP_020980415.1_Arachis_duranensis [Archaeplastida]:0.0137509684)96:0.017330)67:0.012874,ptFfh@ABN06080.1_Medicago_truncatula [Archaeplastida]:0.0487334956)90:0.014457)59:0.007735,ptFfh@XP_024442952.1_Populus_trichocarpa [Archaeplastida]:0.0417068096)58:0.006389)66:0.010844,ptFfh@XP_002530328.1_Ricinus_communis [Archaeplastida]:0.0187960593)38:0.005571,ptFfh@XP_021621164.1_Manihot_esculenta [Archaeplastida]:0.0192068298)99:0.048207,ptFfh@OAY71139.1_Ananas_comosus [Archaeplastida]:0.0365732185)82:0.023363,(ptFfh@XP_021316972.1_Sorghum_bicolor [Archaeplastida]:0.0308850736,ptFfh@XP_003576310.1_Brachypodium_distachyon [Archaeplastida]:0.0481639723)100:0.113750)100:0.085591,ptFfh@ABR16458.1_Picea_sitchensis [Archaeplastida]:0.1159776561)100:0.092925,ptFfh@XP_002964409.2_Selaginella_moellendorffii [Archaeplastida]:0.2064512956)97:0.052329,(ptFfh@A9RGM4_Physcomitrella_patens_subsp_patens [Archaeplastida]:0.0000029386,ptFfh@XP_024383502.1_Physcomitrella_patens [Archaeplastida]:0.0000020523)100:0.081551)100:0.144739,ptFfh@GAQ87100.1_Klebsormidium_nitens [Archaeplastida]:0.2422009607)100:0.076287,(((ptFfh@A0A2P6TNJ4_Chlorella_sorokiniana [Archaeplastida]:0.1441540374,ptFfh@XP_011401759.1_Auxenochlorella_protothecoides [Archaeplastida]:0.3120269863)98:0.090510,ptFfh@I0YJE8_Coccomyxa_subellipsoidea_strain_C-169 [Archaeplastida]:0.1686530652)98:0.049590,(ptFfh@A0A061R4M7_Tetraselmis_sp_GSL018 [Archaeplastida]:0.2878516310,ptFfh@GAX85777.1_Chlamydomonas_eustigma [Archaeplastida]:0.3153123145)98:0.048037)100:0.077544)100:0.159505)99:0.053576)100:0.204142,bacFfh@OJX56912.1_Candidatus_Kapabacteria_thiocyanatum [Bacteroidetes]:0.3725870565)97:0.052153,(bacFfh@PIQ24328.1_Candidatus_Blackallbacteria [unassigned]:0.2666285421,bacFfh@PCJ61345.1_Planctomycetes_bacterium [Planctomycetes]:0.4888025678)99:0.079905)98:0.052849,((((bacFfh@WP_102742496.1_Akkermansia_muciniphila [Verrucomicrobia]:0.0108944738,bacFfh@CDD93459.1_Akkermansia_sp_CAG_344 [Verrucomicrobia]:0.0135097465)100:0.188890,((bacFfh@WP_018970803.1_Rubritalea_marina [Verrucomicrobia]:0.0706131681,bacFfh@WP_105044656.1_Rubritalea_profundi [Verrucomicrobia]:0.0524410640)100:0.137123,(bacFfh@WP_035603039.1_Haloferula_sp_BvORR071 [Verrucomicrobia]:0.1707929879,bacFfh@WP_038136700.1_Verrucomicrobia_bacterium_SCGC_AAA168-F10 [Verrucomicrobia]:0.2280097053)99:0.033449)100:0.074610)100:0.120492,bacFfh@WP_006980240.1_Chthoniobacter_flavus [Verrucomicrobia]:0.2812190468)100:0.260028,((((((((((((eukSRP54@GFYU01001334.1_Ancoracysta_twista [Ancoracysta]:0.2124653486,(eukSRP54@XP_001018396.1_Tetrahymena_thermophila_SB210 [Alveolata]:0.1389680418,eukSRP54@XP_004039207.1_Ichthyophthirius_multifiliis [Alveolata]:0.1348102060)100:0.229770)97:0.049189,((eukSRP54@CBN75372.1_Ectocarpus_siliculosus [Stramenopiles]:0.2676032701,(eukSRP54@OEU17714.1_Fragilariopsis_cylindrus_CCMP1102 [Stramenopiles]:0.1345155328,eukSRP54@XP_002185775.1_Phaeodactylum_tricornutum_CCAP1055-1 [Stramenopiles]:0.1585511419)100:0.120135)100:0.094910,(eukSRP54@RAW42240.1_Phytophthora_cactorum [Stramenopiles]:0.0093548209,eukSRP54@XP_008893053.1_Phytophthora_parasitica_INRA-310 [Stramenopiles]:0.0000023741)100:0.202045)100:0.190548)98:0.054846,eukSRP54@NA_Spironema_sp [Hemimastigophora]:0.3988684899)52:0.028457,((eukSRP54@GGUN01039927.1_Goniomonas_avonlea [Cryptophyta]:0.0592952437,eukSRP54@CAMNT_0031843689_Goniomonas_pacifica [Cryptophyta]:0.0570135382)100:0.127905,(eukSRP54@XP_004367866.1_Acanthamoeba_castellanii_strain_Neff [Amoebozoa]:0.2096673015,(((eukSRP54@XP_023304254.1_Lucilia_cuprina [Opisthokonta]:0.0071706299,((eukSRP54@SPP87160.1_Drosophila_guanche [Opisthokonta]:0.0023155166,eukSRP54@XP_017138354.1_Drosophila_miranda [Opisthokonta]:0.0000020493)100:0.010804,eukSRP54@XP_002068627.1_Drosophila_willistoni [Opisthokonta]:0.0080986093)100:0.021229)100:0.030288,(eukSRP54@ETN65735.1_Anopheles_darlingi [Opisthokonta]:0.0129829643,eukSRP54@XP_021695011.1_Aedes_aegypti [Opisthokonta]:0.0081718924)100:0.037495)100:0.220624,(eukSRP54@EPZ32142.1_Rozella_allomycis_CSF55 [Opisthokonta]:0.2633992014,(eukSRP54@XP_016608410.1_Spizellomyces_punctatus_DAOM_BR117 [Opisthokonta]:0.1254962132,((((eukSRP54@KNZ71807.1_Termitomyces_sp_J132 [Opisthokonta]:0.0311474368,(eukSRP54@KIK08302.1_Laccaria_amethystina_LaAM-08-1 [Opisthokonta]:0.0235702221,(eukSRP54@KXN86887.1_Leucoagaricus_sp_SymC_cos [Opisthokonta]:0.0302955132,eukSRP54@XP_001833227.1_Coprinopsis_cinerea_okayama [Opisthokonta]:0.0650786348)57:0.009030)55:0.006461)55:0.003554,eukSRP54@PBK96926.1_Armillaria_gallica [Opisthokonta]:0.0195482281)58:0.011397,eukSRP54@KDQ33155.1_Pleurotus_ostreatus_PC15 [Opisthokonta]:0.0275025315)91:0.044910,eukSRP54@RDX56745.1_Polyporus_brumalis [Opisthokonta]:0.0252372797)100:0.381442)70:0.051188)100:0.079911)100:0.107015)65:0.054720)61:0.045015)58:0.028405,(eukSRP54@XP_024367976.1_Physcomitrella_patens [Archaeplastida]:0.0650606114,(eukSRP54@PON48078.1_Parasponia_andersonii [Archaeplastida]:0.0093052137,((eukSRP54@XP_006465307.1_Citrus_sinensis [Archaeplastida]:0.0046581489,eukSRP54@XP_006427317.1_Citrus_clementina [Archaeplastida]:0.0000029267)100:0.018337,(((eukSRP54@XP_012455795.1_Gossypium_raimondii [Archaeplastida]:0.0023181376,eukSRP54@XP_022724150.1_Durio_zibethinus [Archaeplastida]:0.0046453862)98:0.000002,eukSRP54@XP_008454178.1_Cucumis_melo [Archaeplastida]:0.0259152480)91:0.005088,eukSRP54@XP_021636295.1_Hevea_brasiliensis [Archaeplastida]:0.0065758187)90:0.007241)97:0.012865)100:0.074160)100:0.188786)98:0.061697,eukSRP54@ANM86233.1_Stygiella_incarcerata [Jakobida]:0.3582642289)50:0.014929,(((eukSRP54@XP_002671296.1-corrected_Naegleria_gruberi [Heterolobosea]:0.0617716163,eukSRP54@NF0102970_p1_Naegleria_fowleri [Heterolobosea]:0.0557975500)100:0.104813,eukSRP54@ACER01000537.1_Naegleri_gruberi [Heterolobosea]:0.5089396136)100:0.155543,eukSRP54@NA_Neovahlkampfia_damariscottae [Heterolobosea]:0.2062322555)100:0.089910)32:0.030085,(eukSRP54@GEZU01011656.1_Heterolobosea_BB2 [Heterolobosea]:0.2055976692,eukSRP54@CAMNT_0005238523_Percolomonas_cosmopolitus_WS [Heterolobosea]:0.4140734138)96:0.058855)82:0.065397,eukSRP54@GECH01013817.1_Pharyngomonas_kirbyi [Heterolobosea]:0.3298397325)99:0.069214,eukSRP54@CAMNT_0031816087_Goniomonas_pacifica [Cryptophyta]:0.6215533851)100:0.311261,((((archSRP54@RLE51417.1_Candidatus_Verstraetearchaeota_archaeon [TACK]:0.1037773813,archSRP54@RLE50907.1_Candidatus_Verstraetearchaeota_archaeon [TACK]:0.1062993623)100:0.155426,(archSRP54@RLF14065.1_Thermoprotei_archaeon [TACK]:0.4011882177,archSRP54@OLS17826.1_Candidatus_Odinarchaeota_archaeon LCB4 [Asgard]:0.3565473710)47:0.040828)73:0.072123,archSRP54@WP_110270916.1_Acidianus_brierleyi [TACK]:0.5862545987)95:0.084119,((archSRP54@WP_087037458.1_Thermococcus_litoralis [Euryarchaeota]:0.0752629314,(archSRP54@WP_062370762.1_Thermococcus_guaymasensis [Euryarchaeota]:0.0706191454,(archSRP54@WP_088864111.1_Thermococcus_barossii [Euryarchaeota]:0.0687306736,archSRP54@WP_050002783.1_Thermococcus_eurythermalis [Euryarchaeota]:0.0500860573)97:0.015108)97:0.039768)100:0.053881,archSRP54@WP_014734678.1_Pyrococcus_sp_ST04 [Euryarchaeota]:0.0569589780)100:0.249609)99:0.091484)97:0.058252,((archSRP54@PIV68086.1_Euryarchaeota_archaeon_CG01_land_8_20_14_3_00_38_12 [Euryarchaeota]:0.2848243628,archSRP54@RLF74017.1_Thermoplasmata_archaeon [Euryarchaeota]:0.1767545999)100:0.159180,((((archSRP54@Q977V2_Haloferax_volcanii [Euryarchaeota]:0.0796196892,(archSRP54@WP_092813433.1_Halopenitus_malekzadehii [Euryarchaeota]:0.0925853650,archSRP54@WP_079234317.1_Halolamina_sp_CBA1230 [Euryarchaeota]:0.1155443883)82:0.023236)83:0.022995,(archSRP54@WP_066299325.1_Haloterrigena_mahii [Euryarchaeota]:0.0000027199,((archSRP54@WP_049990021.1_Halopiger_salifodinae [Euryarchaeota]:0.0166664659,archSRP54@WP_006826848.1_Natrialba_taiwanensis [Euryarchaeota]:0.0294760672)100:0.010226,archSRP54@WP_005578715.1_Natronobacterium_gregoryi [Euryarchaeota]:0.0480330970)100:0.023231)100:0.111493)100:0.352796,archSRP54@WP_011449961.1_Methanospirillum_hungatei [Euryarchaeota]:0.3437339084)97:0.095914,((archSRP54@WP_095645771.1_Methanosarcina_spelaei [Euryarchaeota]:0.1333553776,archSRP54@KXS43658.1_Methanolobus_sp_T82-4 [Euryarchaeota]:0.1361978246)100:0.081367,archSRP54@WP_014405995.1_Methanocella_conradii [Euryarchaeota]:0.2087357361)59:0.042946)100:0.143475)100:0.133400)100:0.616571)94:0.035545)82:0.030107,(bacFfh@OPX24629.1_Candidatus_Latescibacteria_bacterium_4484_107 [Bacteroidetes]:0.3161005924,bacFfh@WP_006928829.1_Caldithrix_abyssi [Calditrichaeota]:0.2967271421)100:0.131317)94:0.037799,(((((((((((((bacFfh@WP_086120482.1_Lactobacillus_reuteri [Firmicutes]:0.2427623599,bacFfh@WP_050441216.1_Streptococcus_pneumoniae [Firmicutes]:0.3019813068)100:0.097434,bacFfh@WP_015076211.1_Carnobacterium_maltaromaticum [Firmicutes]:0.1306474986)100:0.115747,bacFfh@PKL00150.1_Tenericutes_bacterium_HGW-Tenericutes-1 [Tenericutes]:0.4303103328)100:0.084227,bacFfh@WP_109984449.1_Gracilibacillus_dipsosauri [Firmicutes]:0.1231451407)100:0.062421,bacFfh@WP_116552983.1_Pueribacillus_theae [Firmicutes]:0.0992968921)100:0.088784,bacFfh@WP_007505027.1_Caldalkalibacillus_thermarum [Firmicutes]:0.0898689209)99:0.048545,bacFfh@WP_089967246.1_Lihuaxuella_thermophila [Firmicutes]:0.1772455213)88:0.029838,((bacFfh@WP_015891890.1_Brevibacillus_brevis [Firmicutes]:0.1261465959,bacFfh@WP_120461224.1_Paenibacillus_aceti [Firmicutes]:0.1944352969)99:0.044691,bacFfh@WP_038091674.1_Tumebacillus_flagellatus [Firmicutes]:0.1438118938)96:0.040143)99:0.055496,bacFfh@WP_109430892.1_Acidibacillus_sulfuroxidans [Firmicutes]:0.3592890594)51:0.031322,(bacFfh@WP_018702437.1_Anaeromusa_acidaminophila [Firmicutes]:0.2175006437,((bacFfh@WP_062283840.1_Moorella_mulderi [Firmicutes]:0.0452513653,bacFfh@WP_106004935.1_Moorella_humiferrea [Firmicutes]:0.0470609125)100:0.158251,bacFfh@WP_046498292.1_Syntrophomonas_zehnderi [Firmicutes]:0.2149364486)100:0.032307)97:0.028685)49:0.030639,(((((bacFfh@KJS18981.1_Clostridiaceae_bacterium_BRH_c20a [Firmicutes]:0.1559526228,bacFfh@PWM50529.1_Clostridiales_bacterium [Firmicutes]:0.2345701946)100:0.088419,bacFfh@CDC00866.1_Firmicutes_bacterium_CAG_41 [Firmicutes]:0.2374448357)79:0.039783,(((((bacFfh@WP_008908195.1_Caloramator_australicus [Firmicutes]:0.1587098318,bacFfh@WP_054875147.1_Oxobacter_pfennigii [Firmicutes]:0.2013532236)96:0.035365,bacFfh@WP_074910001.1_Proteiniclasticum_ruminis [Firmicutes]:0.3260433093)97:0.048484,bacFfh@WP_058486324.1_Defluviitalea_phaphyphila [Firmicutes]:0.2197676029)63:0.011698,bacFfh@WP_072469189.1_Urinacoccus_massiliensis [Firmicutes]:0.2774731048)69:0.018304,(bacFfh@WP_117520369.1_Ruminococcus_sp_AF43-11 [Firmicutes]:0.2530205695,bacFfh@WP_022786100.1_Clostridiales_bacterium_NK3B98 [Firmicutes]:0.3101341591)94:0.113366)68:0.022324)93:0.036757,bacFfh@WP_013275867.1_Thermosediminibacter_oceani [Firmicutes]:0.2376994158)83:0.038566,(bacFfh@WP_072905435.1_Anaerobranca_californiensis [Firmicutes]:0.2066108889,bacFfh@OPL10543.1_Firmicutes_bacterium_ML8_F2 [Firmicutes]:0.4383016514)78:0.068413)6:0.021425)82:0.071452,(bacFfh@ACX52518.1_Ammonifex_degensii_KC4 [Firmicutes]:0.2301071553,(bacFfh@WP_054491650.1_Ardenticatena_maritima [Chloroflexi]:0.2124797067,bacFfh@EFH87465.1_Ktedonobacter_racemifer_DSM_44963 [Chloroflexi]:0.4736777768)99:0.096792)95:0.064583)77:0.021847,bacFfh@OIP71264.1_Candidatus_Atribacteria_bacterium_CG2_30_33_13 [Atribacteria]:0.7887511599)95:0.018799)90:0.029932,bacFfh@WP_049675438.1_Desulfocarbo_indianensis [Deltaproteobacteria]:0.3818395171)100:0.105624,((((bacFfh@WP_033187470.1_Pseudoalteromonas_sp_PLSV [Gammaproteobacteria]:0.1365006851,(bacFfh@WP_078744295.1_Oceanospirillum_multiglobuliferum [Gammaproteobacteria]:0.1327375183,bacFfh@WP_039914025.1_Cellvibrio_mixtus [Gammaproteobacteria]:0.1734175327)100:0.054381)100:0.082779,((bacFfh@WP_114137085.1_Klebsiella_pneumoniae [Gammaproteobacteria]:0.0462690568,bacFfh@RJL31521.1_Pectobacterium_polaris [Gammaproteobacteria]:0.0609334135)100:0.052500,bacFfh@WP_024496304.1_Candidatus_Schmidhempelia_bombi [Gammaproteobacteria]:0.1413602908)100:0.058029)100:0.083361,bacFfh@WP_091713482.1_Methylophaga_sulfidovorans [Gammaproteobacteria]:0.1824634190)100:0.059701,bacFfh@WP_025769383.1_Thioalkalivibrio_sp_HK1 [Gammaproteobacteria]:0.3098855048)100:0.206970)100:0.120055,(((((((((bacFfh@ANK81659.1_Rhizobiales_bacterium_NRL2 [Alphaproteobacteria]:0.1983664187,bacFfh@WP_109920731.1_Zavarzinia_compransoris [Alphaproteobacteria]:0.1897777855)95:0.028032,(bacFfh@OUR76855.1_Alphaproteobacteria_bacterium_46_93_T64 [Alphaproteobacteria]:0.0639843435,bacFfh@WP_025896931.1_Sneathiella_glossodoripedis [Alphaproteobacteria]:0.0378971061)100:0.202562)100:0.040145,((bacFfh@PCI43326.1_Alphaproteobacteria_bacterium [Alphaproteobacteria]:0.1887366148,(bacFfh@WP_028466142.1_Nisaea_denitrificans [Alphaproteobacteria]:0.1078858158,bacFfh@OUU28491.1_Candidatus_End

lissoclinum_sp_TMED37 [Alphaproteobacteria]:0.2272547666)100:0.108772)80:0.062188,(((bacFfh@WP_018634118.1_Neomegalonema_perideroedes [Alphaproteobacteria]:0.2387877582,(bacFfh@OJT95039.1_Alphaproteobacteria_bacterium_65-7 [Alphaproteobacteria]:0.1966312081,bacFfh@OQW59094.1_Proteobacteria_bacterium_HN_bin10 [Proterobacteria]:0.3349031293)99:0.077290)79:0.041796,(bacFfh@WP_116392064.1_Parvularcula_sp_SM1705 [Alphaproteobacteria]:0.2546881022,bacFfh@RCL81099.1_SAR116_cluster_bacterium [Alphaproteobacteria]:0.3513542294)80:0.043037)91:0.036583,(((bacFfh@GBE42717.1_Bacterium_BMS3Bbin10 [unassigned]:0.2258459730,((bacFfh@OUU83527.1_Hyphomicrobiaceae_bacterium_TMED74 [Alphaproteobacteria]:0.1451975235,bacFfh@WP_099557780.1_Hartmannibacter_diazotrophicus [Alphaproteobacteria]:0.1723856161)99:0.058353,(bacFfh@WP_013420482.1_Rhodomicrobium_vannielii [Alphaproteobacteria]:0.1482098490,((bacFfh@WP_111197444.1_Rhizobiales_bacterium_KCTC_52945 [Alphaproteobacteria]:0.1784964690,bacFfh@WP_038035438.1_Thermopetrobacter_sp_TC1 [Alphaproteobacteria]:0.2557508036)100:0.044261,bacFfh@WP_085770433.1_Methylocystis_bryophila [Alphaproteobacteria]:0.3082592684)95:0.029975)64:0.024660)65:0.014849)93:0.024776,(bacFfh@PCI85686.1_Rhizobiales_bacterium [Alphaproteobacteria]:0.0073892491,bacFfh@PCJ00993.1_OCS116_cluster_bacterium [Alphaproteobacteria]:0.0099651321)100:0.239814)95:0.015602,(bacFfh@PKQ09217.1_Alphaproteobacteria_bacterium_HGW-Alphaproteobacteria-12 [Alphaproteobacteria]:0.1637372446,bacFfh@RCL83452.1_PS1_clade_bacterium [Alphaproteobacteria]:0.3084356308)96:0.064030)97:0.038778)93:0.034845)71:0.031375)70:0.024424,((((((((bacFfh@WP_014746762.1_Tistrella_mobilis [Alphaproteobacteria]:0.2436741076,(bacFfh@WP_028877797.1_Terasakiella_pusilla [Alphaproteobacteria]:0.0365537820,bacFfh@WP_069189220.1_Terasakiella_sp_PR1 [Alphaproteobacteria]:0.0720941117)100:0.165834)60:0.036111,(((bacFfh@OJX70273.1_Magnetospirillum_sp_64-120 [Alphaproteobacteria]:0.0377932161,bacFfh@WP_024081694.1_Magnetospirillum_gryphiswaldense [Alphaproteobacteria]:0.0308300137)100:0.017513,bacFfh@WP_068497751.1_Magnetospirillum_moscoviense [Alphaproteobacteria]:0.0453069793)100:0.053948,((bacFfh@WP_002727862.1_Phaeospirillum_molischianum [Alphaproteobacteria]:0.0049441075,bacFfh@WP_074764996.1_Phaeospirillum_fulvum [Alphaproteobacteria]:0.0074407419)100:0.072335,(bacFfh@WP_011386413.1_Magnetospirillum_magneticum [Alphaproteobacteria]:0.0426191518,bacFfh@WP_068493492.1_Magnetospirillum_marisnigri [Alphaproteobacteria]:0.0332132909)100:0.022033)99:0.033723)100:0.121805)59:0.030344,(((bacFfh@WP_008853607.1_Commensalibacter_intestini [Alphaproteobacteria]:0.0000021325,bacFfh@WP_086632043.1_Commensalibacter_intestini [Alphaproteobacteria]:0.0000021325)100:0.010076,bacFfh@WP_034336672.1_Commensalibacter_sp_MX01 [Alphaproteobacteria]:0.0089952872)100:0.281812,bacFfh@WP_043360684.1_Belnapia_sp_F-4-1 [Alphaproteobacteria]:0.1515031629)100:0.131468)59:0.014696,(((bacFfh@WP_019645789.1_Novispirillum_itersonii [Alphaproteobacteria]:0.1360451467,bacFfh@WP_092613925.1_Roseospirillum_parvum [Alphaproteobacteria]:0.2052304169)64:0.031195,(((bacFfh@CDB39986.1_Azospirillum_sp_CAG_260 [Alphaproteobacteria]:0.0049987174,bacFfh@OLA79528.1_Azospirillum_sp_47_25 [Alphaproteobacteria]:0.0000021251)100:0.080287,bacFfh@CDB53938.1_Azospirillum_sp_CAG_239 [Alphaproteobacteria]:0.0555207953)100:0.255845,bacFfh@PHY00942.1_Rhodospirillaceae_bacterium [Alphaproteobacteria]:0.2920654517)84:0.067938)51:0.012830,(bacFfh@CCZ21287.1_Acetobacter_sp_CAG_977 [Alphaproteobacteria]:0.2335846755,bacFfh@WP_041795188.1_Pararhodospirillum_photometricum [Alphaproteobacteria]:0.2193269308)56:0.014682)85:0.038546)58:0.024666,(bacFfh@WP_073953247.1_Thalassospira_sp_TSL5-1 [Alphaproteobacteria]:0.1942508650,bacFfh@OFX07559.1_Alphaproteobacteria_bacterium_RIFOXYD12_FULL_60_8 [Alphaproteobacteria]:0.2199955561)86:0.064769)82:0.035877,((bacFfh@OHC73580.1_Rhodospirillales_bacterium_RIFCSPLOWO2_02_FULL_58_16 [Alphaproteobacteria]:0.1975630523,bacFfh@OUT52366.1_Rhodospirillaceae_bacterium_TMED8 [Alphaproteobacteria]:0.3161916809)87:0.053489,bacFfh@WP_069956818.1_Magnetovibrio_blakemorei [Alphaproteobacteria]:0.1988062453)100:0.061456)71:0.032489,(((bacFfh@WP_012973141.1_Azospirillum_lipoferum [Alphaproteobacteria]:0.1093245800,bacFfh@WP_094454746.1_Niveispirillum_lacus [Alphaproteobacteria]:0.1853987910)100:0.069121,(bacFfh@WP_108794692.1_Rhodospirillaceae_bacterium_Spongia-Bin9 [Alphaproteobacteria]:0.2103533478,bacFfh@WP_092823393.1_Rhodospirillales_bacterium_URHD0017 [Alphaproteobacteria]:0.2492483708)97:0.052737)94:0.025451,(((bacFfh@OIN86659.1_Alphaproteobacteria_bacterium_CG1_02_46_17 [Alphaproteobacteria]:0.2117622193,bacFfh@PZQ45682.1_Micavibrio_aeruginosavorus [Alphaproteobacteria]:0.1445347834)99:0.052698,(bacFfh@PCJ00252.1_Alphaproteobacteria_bacterium [Alphaproteobacteria]:0.2347078287,bacFfh@WP_015467793.1_Micavibrio_aeruginosavorus [Alphaproteobacteria]:0.1422153809)100:0.047038)100:0.105865,bacFfh@OUX71121.1_Rhodospirillaceae_bacterium_TMED140 [Alphaproteobacteria]:0.2397692586)95:0.052313)73:0.019855)63:0.019992,((((bacFfh@WP_068791160.1_unknown alphaproteobacterium [Alphaproteobacteria]:0.1473128942,bacFfh@WP_027287498.1_Rhodovibrio_salinarum [Alphaproteobacteria]:0.2502545798)99:0.057232,bacFfh@WP_046506348.1_Kiloniella_litopenaei [Alphaproteobacteria]:0.1915589632)91:0.016870,bacFfh@WP_119283627.1_Rhodospirillaceae_bacterium_SYSU_D60006 [Alphaproteobacteria]:0.2008317899)99:0.042379,bacFfh@PPR12333.1_Alphaproteobacteria_bacterium_MarineAlpha11_Bin1 [Alphaproteobacteria]:0.3029723547)89:0.025291)65:0.025550)76:0.023438,(bacFfh@WP_115937331.1_Aestuariispira_insulae [Alphaproteobacteria]:0.1767585965,bacFfh@PCJ58386.1_Rhodospirillaceae_bacterium [Alphaproteobacteria]:0.3673954585)82:0.028452)79:0.029954,((((((bacFfh@SCW52489.1_Sphingobium_faniae [Alphaproteobacteria]:0.0354312934,(bacFfh@WP_010335520.1_Sphingobium_yanoikuyae [Alphaproteobacteria]:0.0211538959,bacFfh@WP_014076705.1_Sphingobium_sp_SYK-6 [Alphaproteobacteria]:0.0978608421)98:0.034737)100:0.058020,(bacFfh@WP_076073722.1_Sphingopyxis_granuli [Alphaproteobacteria]:0.0366445584,bacFfh@WP_089215287.1_Sphingopyxis_indica [Alphaproteobacteria]:0.0325315298)100:0.088096)100:0.048383,bacFfh@WP_022691338.1_Sphingomonas-like_bacterium_B12 [Alphaproteobacteria]:0.0979899963)58:0.019588,(bacFfh@WP_116091360.1_Sphingomonas_crusticola [Alphaproteobacteria]:0.1151357182,bacFfh@WP_119532804.1_Sphingomonas_sp_DAC4 [Alphaproteobacteria]:0.1963407800)97:0.042431)99:0.036104,(bacFfh@WP_011240910.1_Zymomonas_mobilis [Alphaproteobacteria]:0.0267933866,bacFfh@WP_013933516.1_Zymomonas_mobilis [Alphaproteobacteria]:0.0405317341)100:0.147388)94:0.034307,bacFfh@WP_072596811.1_Sphingomonas_sp_JJ-A5 [Alphaproteobacteria]:0.1349620851)100:0.157902)86:0.042117,(((((bacFfh@OJX13986.1_Caedibacter_sp_37-49 [Alphaproteobacteria]:0.2028665488,bacFfh@WP_085783826.1_Candidatus_Nucleicultrix_amoebiphila [Alphaproteobacteria]:0.1947228917)100:0.052336,bacFfh@OYZ36327.1_Alphaproteobacteria_bacterium_16-39-46 [Alphaproteobacteria]:0.2639991913)100:0.045917,bacFfh@WP_032113236.1_Candidatus_Paracaedibacter_symbiosus [Alphaproteobacteria]:0.2536622794)99:0.036513,(((bacFfh@KKB96089.1_Arcanobacter_lacustris [Alphaproteobacteria]:0.2689492159,bacFfh@PIR38902.1_Alphaproteobacteria_bacterium_CG11_big_fil_rev_8_21_14_0_20_39_49 [Alphaproteobacteria]:0.2120285663)99:0.048403,bacFfh@PLX30515.1_Alphaproteobacteria_bacterium [Alphaproteobacteria]:0.3791216837)69:0.036887,(((bacFfh@OUT75042.1_Rhizobiales_bacterium_TMED25 [Alphaproteobacteria]:0.0898239531,(bacFfh@OUX67412.1_Rhizobiales_bacterium_TMED227 [Alphaproteobacteria]:0.0812417028,bacFfh@OUT82378.1_Rhizobiales_bacterium_TMED28 [Alphaproteobacteria]:0.0888256857)99:0.021667)100:0.242791,(bacFfh@OUW71296.1_Rickettsiales_bacterium_TMED211 [Alphaproteobacteria]:0.4312191656,bacFfh@PDH20339.1_Pelagibacterales_bacterium_MED-G40 [Alphaproteobacteria]:0.4977861457)85:0.070528)82:0.064873,(bacFfh@OJV16027.1_Alphaproteobacteria_bacterium_33-17 [Alphaproteobacteria]:0.4433333322,(bacFfh@WP_025264366.1_Wolbachia_endosymbiont_of_Onchocerca_volvulus [Alphaproteobacteria]:0.1935306994,bacFfh@WP_065432712.1_Ehrlichia_ruminantium [Alphaproteobacteria]:0.3039414174)100:0.250057)87:0.063344)61:0.035164)96:0.040996)98:0.074303,((bacFfh@WP_027134478.1_Geminicoccus_roseus [Alphaproteobacteria]:0.2303561920,(bacFfh@WP_088559973.1_Arboricoccus_pini [Alphaproteobacteria]:0.0000021325,bacFfh@WP_088559973.1_Arboriscoccus_pini [Alphaproteobacteria]:0.0000025135)100:0.197131)100:0.070234,bacFfh@PZP86081.1_Azospirillum_brasilense [Alphaproteobacteria]:0.3559079054)100:0.086455)80:0.041969)82:0.023695,(bacFfh@PPR36214.1_Alphaproteobacteria_bacterium_MarineAlpha9_Bin6 [Alphaproteobacteria]:0.3028982363,bacFfh@PPR79452.1_Alphaproteobacteria_bacterium_MarineAlpha2_Bin1 [Alphaproteobacteria]:0.3651273236)97:0.061760)68:0.019590,((bacFfh@PPR20700.1_Alphaproteobacteria_bacterium_MarineAlpha10_Bin2 [Alphaproteobacteria]:0.0408869934,bacFfh@PPR25390.1_Alphaproteobacteria_bacterium_MarineAlpha10_Bin1 [Alphaproteobacteria]:0.0538717990)100:0.171475,bacFfh@PPR14985.1_Alphaproteobacteria_bacterium_MarineAlpha9_Bin3 [Alphaproteobacteria]:0.3795388110)90:0.067231)99:0.072513)100:0.231356,(((mtFfh@NA_Goniomonas_avonlea [Cryptophyta]:0.3978877892,mtFfh@NA_Goniomonas_pacifica [Cryptophyta]:0.7145744866)100:0.286375,(mtFfh@NA_Hemimastix_kukwesjijk [Hemimastigophora]:0.4481980623,mtFfh@NA_Spironema_sp [Hemimastigophora]:0.6862408434)100:0.608949)59:0.051240,(((mtFfh@NA_Pharyngomonas_kirbyi [Heterolobosea]:0.4667191415,(mtFfh@NA_Percolomonas_cosmopolitus_strain_WS [Heterolobosea]:0.4112861489,mtFfh@NA_Percolomonas_ex_Nitzchia_Cheng_2013 [Heterolobosea]:0.6420002045)100:0.674242)95:0.082879,(mtFfh@NA_Heterolobosea_BB2 [Heterolobosea]:0.3814217369,(mtFfh@NA_Neovahlkampfia_damariscottae [Heterolobosea]:0.5152040153,(mtFfh@NA_Naegleri_gruberi [Heterolobosea]:0.1061593776,(mtFfh@NA_Naegleria_fowleri [Heterolobosea]:0.0157582854,mtFfh@NA_Naegleria_lovaniensis [Heterolobosea]:0.0169880949)100:0.045594)100:0.447296)96:0.078025)96:0.091756)99:0.136664,mtFfh@NA_Percolomonas_cosmopolitus_strain_AE [Heterolobosea]:1.1882013811)98:0.059524)61:0.059495)100:0.385527);

**Phylogenetic tree from Fig. 3C (IQ-tree, LG4X model 1000 ultrafast bootstraps with bnni, 217 OTUs)**

(ptFtsY@MMETSP0308_Transcript_18196_m19230_Gloeochaete_wittrockiana [Archaeplastida]:0.4741481496,((((ptFtsY@PRW61060.1_Chlorella_sorokiniana_[Archaeplastida]:0.1431118441,(ptFtsY@XP_005651149.1_Coccomyxa_subellipsoidea [Archaeplastida]:0.2080183879,ptFtsY@XP_001697752.1_Chlamydomonas_reinhardtii [Archaeplastida]:0.3980548973)84:0.0725595462)88:0.0673326822,ptFtsY@XP_011399720.1_Auxenochlorella_protothecoides [Archaeplastida]:0.3499315296)87:0.0824067631,ptFtsY@XP_003080532.1_Ostreococcus_tauri [Archaeplastida]:0.4179242681)66:0.0646582996,(((((ptFtsY@KHF98418.1_Cell_division_FtsY_chloroplastic-like_protein_Gossypium_arboreum [Archaeplastida]:0.0302496280,ptFtsY@XP_022757233.1_Durio_zibethinus [Archaeplastida]:0.0331089367)93:0.0170762412,ptFtsY@XP_023879170.1_Quercus_suber [Archaeplastida]:0.0268780518)51:0.0045173083,ptFtsY@XP_010055217.1_Eucalyptus_grandis [Archaeplastida]:0.0689581527)39:0.0136627823,ptFtsY@XP_021817084.1_Prunus_avium [Archaeplastida]:0.0427968675)75:0.0331341207,ptFtsY@NP_566056.1_Arabidopsis_thaliana [Archaeplastida]:0.0599392129)100:0.2824262484)100:0.2049322413,(((((((bacFtsY@RPG16414.1_FtsY_Phycisphaera_sp_TMED9 [Planctomycetes]:0.4076011042,bacFtsY@RMH26650.1_Planctomycetes_bacterium [Planctomycetes]:0.3487664781)99:0.2175794343,bacFtsY@WP_088252003.1_Fimbriiglobus_ruber [Planctomycetes]:0.3933216697)83:0.1002532711,(bacFtsY@WP_056203513.1_Pelomonas_sp_Root1237 [Betaproteobacteria]:0.0404582047,(bacFtsY@PZP35629.1_Roseateles_depolymerans [Betaproteobacteria]:0.0329138886,((bacFtsY@WP_124447410.1_Paucibacter_sp_KBW04 [Betaproteobacteria]:0.0612265762,bacFtsY@OWQ45088.1_Mitsuaria_noduli [Betaproteobacteria]:0.0504875375)75:0.0417233444,(bacFtsY@WP_066336977.1_Azohydromonas_lata [Betaproteobacteria]:0.0898427912,bacFtsY@WP_089417744.1_Vitreoscilla_filiformis [Betaproteobacteria]:0.1206533334)63:0.0340523368)68:0.0351480991)59:0.0238686861)100:0.5518386518)54:0.0443801671,(((((((((bacFtsY@WP_107510123.1_Staphylococcus_fleurettii [Firmicutes]:0.1425841335,bacFtsY@AVK83142.1_Lysinibacillus_sp_B2A1 [Firmicutes]:0.1510831024)96:0.0865924686,(bacFtsY@WP_009554695.1_Lactobacillus_saerimneri [Firmicutes]:0.2073368656,(bacFtsY@WP_009491663.1_Catellicoccus_marimammalium [Firmicutes]:0.1762346737,((bacFtsY@WP_054646204.1_Lactobacillus_lindneri [Firmicutes]:0.0587951058,bacFtsY@WP_056961480.1_Lactobacillus_florum [Firmicutes]:0.1374198768)94:0.0723664669,bacFtsY@WP_103423367.1_Lactobacillus_sanfranciscensis [Firmicutes]:0.0912257955)100:0.1941674131)49:0.0329566204)100:0.1634242595)77:0.0799228234,(bacFtsY@WP_069327418.1_Paenibacillus_sp_TI45-13ar [Firmicutes]:0.1658578394,(bacFtsY@WP_091834879.1_Marininema_halotolerans [Firmicutes]:0.1687819176,bacFtsY@WP_028778316.1_Shimazuella_kribbensis [Firmicutes]:0.1476236579)98:0.1058211870)78:0.0629570342)91:0.0684312828,(bacFtsY@WP_026974763.1_Alicyclobacillus_contaminans [Firmicutes]:0.3870738839,bacFtsY@PTQ57904.1_Candidatus_Carbobacillus_altaicus [Firmicutes]:0.3475656909)90:0.1014400560)86:0.0585087284,bacFtsY@WP_073092242.1_Thermosyntropha_lipolytica [Firmicutes]:0.3973494377)50:0.0343941175,((bacFtsY@WP_072972532.1_Tissierella_praeacuta [Firmicutes]:0.2397413647,bacFtsY@WP_054252106.1_Neofamilia_massiliensis [Firmicutes]:0.3415220791)98:0.1469002905,bacFtsY@CDA51269.1_Clostridium_sp_CAG-138 [Firmicutes]:0.4949567390)56:0.0246442606)78:0.0718075537,bacFtsY@KUO52399.1_Desulfitibacter_sp_BRH_c19 [Firmicutes]:0.3544666922)56:0.0520561561,bacFtsY@EEG77220.1_Dethiobacter_alkaliphilus_AHT_1 [Firmicutes]:0.2748314437)78:0.0470423742,((((((bacFtsY@AEG15826.1_Desulfofundulus_kuznetsovii_DSM_6115 [Firmicutes]:0.2503701090,bacFtsY@WP_075860076.1_Carboxydothermus_pertinax [Firmicutes]:0.3082928524)67:0.0939063156,(bacFtsY@WP_012175138.1_Desulfococcus_oleovorans [Deltaproteobacteria]:0.3912463387,bacFtsY@WP_009108227.1_Desulfovibrio_sp_U5L [Deltaproteobacteria]:0.5066545021)47:0.0354927891)53:0.0429035513,(bacFtsY@OGP30931.1_Deltaproteobacteria_bacterium_GWC2_42_11 [Deltaproteobacteria]:0.3742171937,((((bacFtsY@GBD43248.1_Bacterium_HR40 [unassigned]:0.3939766113,((bacFtsY@EPY01636.1_Phaeospirillum_fulvum_MGU-K5 [Alphaproteobacteria]:0.1680021656,(bacFtsY@OFX10022.1_Alphaproteobacteria_bacterium_RIFOXYD12_FULL_60_8 [Alphaproteobacteria]:0.1542980049,bacFtsY@WP_092615803.1_Roseospirillum_parvum [Alphaproteobacteria]:0.2378382591)75:0.0578758745)85:0.0688814759,bacFtsY@OUU28495.1_Candidatus_Endolissoclinum_sp_TMED37 [Alphaproteobacteria]:0.3539485913)57:0.0626622840)51:0.0242699964,((bacFtsY@KRS17267.1_Roseovarius_indicus [Alphaproteobacteria]:0.0705193701,bacFtsY@WP_025048799.1_Sulfitobacter_mediterraneus [Alphaproteobacteria]:0.0546387052)100:0.2223156112,bacFtsY@AIL12880.1_Candidatus_Paracaedimonas_acanthamoebae [Alphaproteobacteria]:0.4495507061)55:0.0723859842)82:0.0344676044,(((bacFtsY@WP_108880431.1_Anderseniella_sp_Alg231-50 [Alphaproteobacteria]:0.2268806565,bacFtsY@WP_109793879.1_Rhizobiales_bacterium [Alphaproteobacteria]:0.2521272980)61:0.0538511278,((((((bacFtsY@SKC16052.1_Bosea_thiooxidans [Alphaproteobacteria]:0.1583367038,((bacFtsY@WP_011996071.1_Xanthobacter_autotrophicus [Alphaproteobacteria]:0.0728790441,bacFtsY@WP_024277252.1_Xanthobacter_sp_126 [Alphaproteobacteria]:0.0415955985)100:0.1256196708,bacFtsY@WP_013168132.1_Starkeya_novella [Alphaproteobacteria]:0.1227328792)97:0.0867237223)89:0.0504017670,bacFtsY@WP_115516251.1_Pseudolabrys_sp_GY_H [Alphaproteobacteria]:0.2580800199)58:0.0297282558,bacFtsY@WP_088520031.1_Rhodoblastus_acidophilus [Alphaproteobacteria]:0.1789070784)84:0.0509470472,bacFtsY@WP_099557887.1_Hartmannibacter_diazotrophicus [Alphaproteobacteria]:0.1406975981)39:0.0158760140,(bacFtsY@WP_029041189.1_Cucumibacter_marinus [Alphaproteobacteria]:0.1708354162,bacFtsY@PPD07903.1_Hyphomicrobium_sp [Alphaproteobacteria]:0.2521917766)32:0.0265029206)59:0.0493722700,(bacFtsY@WP_113333951.1_Rhizobiales_bacterium [Alphaproteobacteria]:0.1446479649,bacFtsY@RCL01761.1_Candidatus_Tokpelaia_sp_JSC085 [Alphaproteobacteria]:0.3226327781)95:0.1146384615)39:0.0233482116)90:0.1089203892,(bacFtsY@ACT57804.1_Hirschia_baltica_ATCC_49814 [Alphaproteobacteria]:0.0000023463,bacFtsY@WP_083773102.1_Hirschia_baltica [Alphaproteobacteria]:0.0000023463)100:0.3919159733)73:0.0400188986)100:0.2367122635,bacFtsY@SME87908.1_Pseudobacteriovorax_antillogorgiicola [Deltaproteobacteria]:0.4833556350)93:0.1322458761)61:0.0638804381)28:0.0185772945,(((bacFtsY@WP_083764053.1_Syntrophobacter_fumaroxidans [Deltaproteobacteria]:0.3744656081,((bacFtsY@PIP45503.1_Deltaproteobacteria_bacterium_CG23_combo_of_CG06-09_8_20_14_all_51_20 [Deltaproteobacteria]:0.3020546627,bacFtsY@PIE60181.1_Desulfobulbus_propionicus [Deltaproteobacteria]:0.3248167183)52:0.0411578969,(bacFtsY@PIP06279.1_Syntrophobacteraceae_bacterium_CG23_combo_of_CG06-09_8_20_14_all_50_8 [Deltaproteobacteria]:0.1474139609,bacFtsY@ABC77884.1_Syntrophus_aciditrophicus_SB [Deltaproteobacteria]:0.2613582251)100:0.2855605307)31:0.0254914524)80:0.0701028566,(bacFtsY@WP_011985037.1_Anaeromyxobacter_sp_Fw109-5 [Deltaproteobacteria]:0.3450361569,(bacFtsY@PID38167.1_Proteobacteria_bacterium [Proterobacteria]:0.3640104258,bacFtsY@KPK53060.1_Myxococcales_bacterium_SG8_38_1 [Deltaproteobacteria]:0.5453455419)68:0.1094218519)78:0.1129214862)49:0.0635594730,((((((bacFtsY@WP_121469822.1_Edaphobacter_dinghuensis [Acidobacteria]:0.1906696861,(bacFtsY@WP_074656074.1_Terriglobus_roseus [Acidobacteria]:0.0123503223,bacFtsY@WP_081490725.1_Terriglobus_roseus [Acidobacteria]:0.0167925078)100:0.1571458917)100:0.3276678876,(bacFtsY@WP_031499580.1_Bryobacter_aggregatus [Acidobacteria]:0.2263868171,bacFtsY@WP_020721417.1_Acidobacteriaceae_bacterium_KBS_96 [Acidobacteria]:0.1037704547)98:0.1230239597)65:0.0577533923,bacFtsY@ANM28936.1_Acidobacteria_bacterium_Mor1 [Acidobacteria]:0.5530596022)74:0.0703930819,(bacFtsY@PYS67814.1_Acidobacteria_bacterium [Acidobacteria]:0.2727291154,bacFtsY@PIE91415.1_Acidobacteria_bacterium [Acidobacteria]:0.4973952754)70:0.0765548111)92:0.1315151200,(bacFtsY@WP_038038136.1_Thermorudis_peleae [Chloroflexi]:0.4006763351,((bacFtsY_RCL55122.1_signal_recognition_particle-docking_protein_FtsY_Synechococcus_sp._MED-G71:0.1189745528,(bacFtsY_WP_038650415.1_signal_recognition_particle-docking_protein_FtsY_Prochlorococcus_sp._MIT_0801:0.2150018032,chrFtsY@AUG32399.1_Paulinella_longichromatophora [Rhizaria]:0.1416072881)99:0.0992253563)100:0.1811605616,((bacFtsY_WP_111893930.1_signal_recognition_particle-docking_protein_FtsY_Arthrospira_sp._O9.13F:0.0734980389,bacFtsY@WP_015110998.1_Nostoc_sp [Cyanobacteria]:0.1107298844)99:0.1627038686,bacFtsY_WP_081705397.1_signal_recognition_particle-docking_protein_FtsY_Gloeobacter_kilaueensis:0.2458791994)67:0.0890152829)100:0.4264376879)81:0.0868538488)59:0.0440649670,(((((((bacFtsY@KGA01103.1_Cobetia_amphilecti [Gammaproteobacteria]:0.0000023463,bacFtsY@WP_107335894.1_Halomonas_sp_SF2003 [Verrucomicrobia]:0.0000023463)100:0.1112272801,bacFtsY@WP_097790462.1_Halomonas_beimenensis [Verrucomicrobia]:0.0874016097)100:0.0763715407,(bacFtsY@WP_091826228.1_Marinobacterium_georgiense [Gammaproteobacteria]:0.1095216860,(bacFtsY@ARM82543.1_Marinobacter_salarius [Gammaproteobacteria]:0.0441247930,bacFtsY@KXS51830.1_Marinobacter_sp_T13-3 [Gammaproteobacteria]:0.0261513192)100:0.1264693669)96:0.0945215687)69:0.0483128056,(bacFtsY@WP_116686709.1_contaminant of Flavobacteriaceae_bacterium Hp12 genome [Gammaproteobacteria]:0.2158555023,bacFtsY@WP_086487265.1_Thioflexothrix_psekupsii [Gammaproteobacteria]:0.2188552271)71:0.0584119069)56:0.0476176766,(((bacFtsY@WP_094041167.1_Zobellella_denitrificans [Gammaproteobacteria]:0.0927171871,bacFtsY@WP_091986549.1_Pseudoalteromonas_denitrificans [Gammaproteobacteria]:0.1691412431)95:0.0540378746,bacFtsY@WP_113743312.1_Anaerobiospirillum_thomasii [Gammaproteobacteria]:0.3871739723)54:0.0229921946,bacFtsY@PPI88525.1_Pantoea_sp_SoEO [Gammaproteobacteria]:0.4234814686)64:0.0475089047)81:0.0735672973,bacFtsY@OUX68934.1_Oceanospirillales_bacterium_TMED91 [Gammaproteobacteria]:0.4582766006)60:0.0640786435,bacFtsY@WP_095208401.1_Luteimonas_sp_JM171 [Gammaproteobacteria]:0.3843510035)95:0.0969149614)32:0.0271417560)55:0.0468487934)62:0.0567629246,((((((bacFtsY@OJX59420.1_Candidatus_Kapabacteria_thiocyanatum [Bacteroidetes]:0.1557963972,bacFtsY@PKL79980.1_Ignavibacteriae_bacterium_HGW-Ignavibacteriae-4 [Ignavibacteriae]:0.2668194365)96:0.1089476435,bacFtsY@KXK57805.1_Chlorobi_bacterium_OLB7 [Chlorobi]:0.2151796161)96:0.1268957891,(bacFtsY@PLX30570.1_Ignavibacteria_bacterium [Ignavibacteriae]:0.1650563519,bacFtsY@OGU26318.1_Ignavibacteria_bacterium_GWA2_54_16 [Ignavibacteriae]:0.3586965303)68:0.0589641274)59:0.0267271383,(bacFtsY@WP_092350764.1_Candidatus_Chrysopegis_kryptomonas [Kryptonia]:0.1967031616,((bacFtsY@OGU83728.1_Ignavibacteria_bacterium_RBG_16_35_7 [Ignavibacteriae]:0.4199146946,bacFtsY@PKL82841.1_Ignavibacteriae_bacterium_HGW-Ignavibacteriae-3 [Ignavibacteriae]:0.4282771041)95:0.1113401172,bacFtsY@OQY74580.1_Ignavibacteriales_bacterium_UTCHB3 [Ignavibacteriae]:0.2951676178)100:0.2079820155)91:0.0814101671)70:0.0400941888,(bacFtsY@PSQ63899.1_Bacteroidetes_bacterium_QH_1_61_8 [Bacteroidetes]:0.3204436291,((bacFtsY@OUV32974.1_Rhodothermaceae_bacterium_TMED105 [Bacteroidetes]:0.3509141692,(bacFtsY@WP_100314957.1_Thermoflavifilum_aggregans [Bacteroidetes]:0.2388370779,bacFtsY@OUU18192.1_Crocinitomicaceae_bacterium_TMED45 [Bacteroidetes]:0.3046203501)58:0.0626307735)65:0.0669294826,((bacFtsY@OUV76101.1_Flavobacteriales_bacterium_TMED123 [Bacteroidetes]:0.2075631733,bacFtsY@PSR05731.1_Bacteroidetes_bacterium_SW_10_40_5 [Bacteroidetes]:0.2777384864)86:0.0790218452,(bacFtsY@WP_103327589.1_Bacteroidetes_endosymbiont_of_Geopemphigus_sp [Bacteroidetes]:0.1968777344,bacFtsY@WP_114910387.1_Cardinium_endosymbiont_of_Sogatella_furcifera [Bacteroidetes]:0.4242961907)84:0.1137221900)54:0.0351697503)46:0.0550035525)99:0.1075894580)95:0.0958151716,bacFtsY@OGC84905.1_Zixibacteria_bacterium_RBG_16_43_9 [Zixibacteria]:0.4272498549)73:0.0728680842)45:0.0344046954,(((bacFtsY@OLB22504.1_Nitrospirae_bacterium_13_2_20CM_2_63_8 [Nitrospirae]:0.5059166229,bacFtsY@EKD41878.1_uncultured_bacterium [unassigned]:0.5733554272)67:0.0590215917,bacFtsY@OYZ20489.1_Bdellovibrio_sp_28-41-41 [Deltaproteobacteria]:0.6573500530)77:0.0734666152,(((bacFtsY@RCK76206.1_Anaerolineae_bacterium [Chloroflexi]:0.3149136717,bacFtsY@KUK71287.1_Anaerolineae_bacterium_49_20 [Chloroflexi]:0.2699474347)100:0.2279678943,bacFtsY@OQY47814.1_Anaerolineaceae_bacterium_4572_78 [Chloroflexi]:0.4918260237)89:0.1056463840,(((((((mtFtsY@NA_Naegleria_gruberi [Heterolobosea]:0.0122254336,(mtFtsY@NA_Naegleria_fowleri [Heterolobosea]:0.0134348549,mtFtsY@NA_Naegleria_lovaniensis [Heterolobosea]:0.0138915253)100:0.0925220511)100:0.3807093318,mtFtsY@CAMNT_0005204377-extended_Percolomonas_cosmopolitus_AE [Heterolobosea]:1.3788724513)62:0.1163389262,mtFtsY@NA_Neovahlkampfia_damariscottae [Heterolobosea]:0.5719335644)79:0.1004274087,(mtFtsY@CAMNT_0005246107_Percolomonas_cosmopolitus_WS [Heterolobosea]:0.3883546087,mtFtsY@NA_partial_Percolomonas_ex_Nitzchia_Cheng_2013 [Heterolobosea]:0.6007876263)100:0.3602267956)90:0.1239026561,(mtFtsY@NA_Heterolobosea_BB2 [Heterolobosea]:0.2123543934,mtFtsY@NA_Pharyngomonas_kirbyi [Heterolobosea]:0.3887707219)92:0.1371876047)94:0.1743195526,((mtFtsY@NA_Ancoracysta-related_Colp-4b [Ancoracysta]:0.7977461607,mtFtsY@NA_partial_Goniomonas_pacifica [Cryptophyta]:1.2286982839)59:0.0804762774,(mtFtsY@NA_N-terminus_Ancoracysta_twista [Ancoracysta]:0.7029442836,mtFtsY@QUTJ01025227.1_Goniomonas_avonlea [Cryptophyta]:0.3603656465)79:0.3030849442)91:0.1764836766)64:0.1001463340,mtFtsY@NA_1_Hemimastix_kukwesjijk [Hemimastigophora]:0.6482327401)89:0.1432641219)82:0.0833518483)46:0.0387658906)54:0.0235161316)78:0.0744714325)60:0.0395320143,(bacFtsY@WP_041017695.1_Criblamydia_sequanensis [Chlamydiae]:0.4914906484,bacFtsY@PCI95412.1_Candidatus_Aerophobetes_bacterium [Aerophobetes]:0.4267023600)97:0.2173557818)76:0.0699998694,((((((((((((eukSRa@MMETSP0308_Transcript_27849_m29213_Gloeochaete_wittrockiana [Archaeplastida]:0.2054394637,(((((eukSRa@ONL92607.1_Zea_mays [Archaeplastida]:0.0664843074,(((eukSRa@XP_021634617.1_Manihot_esculenta [Archaeplastida]:0.0125216621,(eukSRa@XP_022750268.1_Durio_zibethinus [Archaeplastida]:0.0135649468,eukSRa@XP_021970653.1_Helianthus_annuus [Archaeplastida]:0.0570625175)50:0.0043252530)53:0.0062607165,eukSRa@XP_003527179.1_Glycine_max [Archaeplastida]:0.0323494763)51:0.0039981507,eukSRa@XP_020873914.1_Arabidopsis_lyrata_subsp_lyrata [Archaeplastida]:0.0544438959)93:0.0254964295)94:0.0365036949,eukSRa@XP_024403926.1_Physcomitrella_patens [Archaeplastida]:0.1557531643)73:0.0375815906,eukSRa@GBG74478.1_Chara_braunii [Archaeplastida]:0.0741041016)89:0.0442099369,eukSRa@CAMPEP_0191492328_Pyramimonas-parkeae-CCMP726 [Archaeplastida]:0.2842830169)53:0.0310320542,((eukSRa@XP_005848975.1_Chlorella_variabilis [Archaeplastida]:0.1343033788,((eukSRa@XP_002949444.1_Volvox_carteri_nagariensis [Archaeplastida]:0.0282809654,eukSRa@KXZ55716.1_Gonium_pectorale [Archaeplastida]:0.0278530444)67:0.0182211057,eukSRa@XP_001692081.1_Chlamydomonas_reinhardtii [Archaeplastida]:0.0277209677)100:0.2221445950)97:0.0567824831,eukSRa@OUS44851.1_Ostreococcus_tauri [Archaeplastida]:0.5864196772)75:0.0381353761)96:0.1015284068)85:0.0650908700,((((eukSRa@NA_Hemimastix_kukwesjijk [Hemimastigophora]:0.2732633194,(eukSRa@GGUN01044729.1_Goniomonas_avonlea [Cryptophyta]:0.1539716543,eukSRa@CAMNT_0031806435_Goniomonas_pacifica [Cryptophyta]:0.1475140623)100:0.2412854025)47:0.0440608141,eukSRa@GFYU01005410.1_Ancoracysta_twista [Ancoracysta]:0.2637692685)56:0.0309971169,(eukSRa@NP_001171313.1_Homo_sapiens [Opisthokonta]:0.2805199832,eukSRa@KOO32508.1_Chrysochromulina_sp_CCMP291 [Haptophyta]:0.3638107226)73:0.1139751663)49:0.0303541210,(eukSRa@GEZU01029986.1_GEZU01002276.1_Heterolobosea_BB2 [Heterolobosea]:0.1775675728,eukSRa@GECH01003431.1_Pharyngomonas_kirbyi [Heterolobosea]:0.4164151254)40:0.0485781227)42:0.0234588871)34:0.0183523528,(((eukSRa@XP_002670075.1_Naegleria_gruberi [Heterolobosea]:0.0500103982,eukSRa@NF0122080_p1_Naegleria_fowleri [Heterolobosea]:0.0481055820)100:0.2632687287,(eukSRa@NA_Neovahlkampfia_damariscottae [Heterolobosea]:0.2166126137,(eukSRa@CAMNT_0005228041_Percolomonas_cosmopolitus_strain_WS [Heterolobosea]:0.3540585822,eukSRa@CAMNT_000520411

_Percolomonas_cosmopolitus_AE [Heterolobosea]:0.4941319781)98:0.1426921871)75:0.0497875158)94:0.0869037179,(eukSRa@KYQ94416.1_Tieghemostelium_lacteum [Amoebozoa]:0.1392142222,eukSRa@XP_012757500.1_Acytostelium_subglobosum_LB1 [Amoebozoa]:0.1165293509)100:0.2317357279)71:0.0688165677)52:0.0248168440,(((((eukSRa@RLN51388.1_Phytophthora_kernoviae [Stramenopiles]:0.0200848295,eukSRa@XP_002906671.1_Phytophthora_infestans_T30-4 [Stramenopiles]:0.0195621886)99:0.0566461461,eukSRa@GAX97406.1_Pythium_insidiosum [Stramenopiles]:0.0951861537)100:0.1718018329,(eukSRa@CBJ30645.1_Ectocarpus_siliculosus [Stramenopiles]:0.2885499926,eukSRa@XP_005853460.1_Nannochloropsis_gaditana_CCMP526 [Stramenopiles]:0.2186110070)90:0.0966768441)100:0.1190204467,eukSRa@XP_012894318.1_Blastocystis_hominis [Stramenopiles]:0.3830129158)94:0.0882420341,(((eukSRa@CEL66130.1_Neospora_caninum [Alveolata]:0.0396859232,eukSRa@ESS33331.1_Toxoplasma_gondii [Alveolata]:0.0161540744)100:0.2683974888,(((eukSRa@POM83525.1_Cryptosporidium_meleagridis [Alveolata]:0.0142645217,eukSRa@XP_668286.1_Cryptosporidium_hominis [Alveolata]:0.0139005171)100:0.3680679335,eukSRa@GBE59094.1_Babesia_ovata [Alveolata]:0.4772779175)65:0.0491539619,eukSRa@SBT01585.1_Plasmodium_malariae [Alveolata]:0.3969531571)79:0.0591444276)97:0.1093051256,(eukSRa@CAMPEP_0170537274_Litonotus_pictus_Strain_P1 [Alveolata]:0.4126573630,eukSRa@XP_004029895.1_Ichthyophthirius_multifiliis [Alveolata]:0.4281621875)97:0.1093321866)86:0.0680663534)57:0.0356522735)66:0.0418365922,eukSRa@ANM86232.1_Stygiella_incarcerata [Jakobida]:0.3724595704)87:0.0627597400,((eukSRa@XP_010703301.1_Leishmania_panamensis [Euglenozoa]:0.0908297676,eukSRa@EPY25999.1_Angomonas_deanei [Euglenozoa]:0.0880637762)78:0.0461310071,eukSRa@EKF29134.1_Trypanosoma_cruzi_marinkellei [Euglenozoa]:0.0892823005)100:0.4585744886)90:0.1749686037,eukSRa@AAD11975.1_Giardia_intestinalis [Metamonada]:0.9384990234)100:0.5265999817,(archFtsY@OIO41360.1_Candidatus_Pacearchaeota_archaeon_CG1_02_31_27 [DPANN]:0.3379812169,archFtsY@PIZ51778.1_Candidatus_Woesearchaeota_archaeon_CG_4_10_14_0_2_um_filter_33_13 [DPANN]:0.4422200807)100:0.2590064636)65:0.0762588315,(archFtsY@WP_048122758.1_Methanosarcina_vacuolata [Euryarchaeota]:0.3040174059,(((archFtsY@WP_004045232.1_Haloferax_volcanii [Euryarchaeota]:0.0957090986,archFtsY@WP_006182963.1_Natrinema_pellirubrum [Euryarchaeota]:0.2257137934)35:0.0632347814,archFtsY@WP_114604860.1_Haloplanus_sp._CBA1112 [Euryarchaeota]:0.1400988715)34:0.0487050502,(archFtsY@WP_021073086.1_Haloarchaeon_3A1_DGR [Euryarchaeota]:0.0421331574,archFtsY@WP_050034148.1_Halorubrum_halophilum [Euryarchaeota]:0.0898993490)94:0.0430846328)100:0.4415359879)100:0.2166796826)46:0.0194484635,(archFtsY@RLI30149.1_Candidatus_Bathyarchaeota_archaeon [TACK]:0.3896151347,archFtsY@RMF91339.1_Euryarchaeota_archaeon [Euryarchaeota]:0.4518924195)75:0.0837782084)46:0.0480225702,(archFtsY@RLI57948.1_Candidatus_Thorarchaeota_archaeon [Asgard]:0.4105613635,archFtsY@OYT54953.1_Candidatus_Altiarchaeales_archaeon_ex4484_2 [DPANN]:0.4033779999)50:0.1036089838)35:0.0435443380,(archFtsY@WP_014122151.1_Thermococcus_sp._AM4 [Euryarchaeota]:0.1151003246,archFtsY@WP_011012907.1_Pyrococcus_furiosus [Euryarchaeota]:0.0846861194)100:0.2458961688)100:0.8491535241)92:0.1039232847,((((ptFtsY@CEM32712.1_Vitrella_brassicaformis_CCMP3155 [Alveolata]:0.3502343567,(((ptFtsY@EWM29383.1_Nannochloropsis_gaditana [Stramenopiles]:0.3382592117,ptFtsY@CBJ31918.1_Ectocarpus_siliculosus [Stramenopiles]:0.3185052328)74:0.0604906774,ptFtsY@XP_002296627.1_Thalassiosira_pseudonana_CCMP1335 [Stramenopiles]:0.5445994422)60:0.0428109845,ptFtsY@XP_009040860.1_Aureococcus_anophagefferens [Stramenopiles]:0.3167677401)61:0.0604846755)92:0.1052489291,(ptFtsY@XP_005714040.1_Chondrus_crispus [Archaeplastida]:0.1197007538,ptFtsY@PXF44704.1_Gracilariopsis_chorda [Archaeplastida]:0.1139598081)100:0.2841281626)60:0.0701296019,ptFtsY@XP_005705405.1_Galdieria_sulphuraria [Archaeplastida]:0.9032182726)68:0.0745342001,ptFtsY@XP_005537370.1_Cyanidioschyzon_merolae_strain_10D [Archaeplastida]:0.5727368070)99:0.1730909964)78:0.0859780988);

**Phylogenetic tree from Fig. S4 (IQ-tree, LG4X model 1000 ultrafast bootstraps with bnni, 154 OTUs)**

(ptFtsY@MMETSP0308_Transcript_18196_m19230_Gloeochaete_wittrockiana [Archaeplastida]:0.4189242408,((((ptFtsY@PRW61060.1_Chlorella_sorokiniana_[Archaeplastida]:0.1572184618,(ptFtsY@XP_005651149.1_Coccomyxa_subellipsoidea [Archaeplastida]:0.2333842198,ptFtsY@XP_001697752.1_Chlamydomonas_reinhardtii [Archaeplastida]:0.3924127686)75:0.0715013729)90:0.0566044297,ptFtsY@XP_011399720.1_Auxenochlorella_protothecoides [Archaeplastida]:0.3364761821)93:0.0747605031,ptFtsY@XP_003080532.1_Ostreococcus_tauri [Archaeplastida]:0.4647231215)82:0.0760346168,(((((ptFtsY@KHF98418.1_Cell_division_FtsY_chloroplastic-like_protein_Gossypium_arboreum [Archaeplastida]:0.0236020561,ptFtsY@XP_022757233.1_Durio_zibethinus [Archaeplastida]:0.0251176423)90:0.0198258852,ptFtsY@XP_021817084.1_Prunus_avium [Archaeplastida]:0.0614534442)64:0.0101718529,ptFtsY@XP_023879170.1_Quercus_suber [Archaeplastida]:0.0214214998)84:0.0193616608,ptFtsY@XP_010055217.1_Eucalyptus_grandis [Archaeplastida]:0.0590721579)71:0.0176167433,ptFtsY@NP_566056.1_Arabidopsis_thaliana [Archaeplastida]:0.0562691795)100:0.2857140642)100:0.2123074362,(((((bacFtsY@RPG16414.1_FtsY_Phycisphaera_sp_TMED9 [Planctomycetes]:0.4287862431,bacFtsY@RMH26650.1_Planctomycetes_bacterium [Planctomycetes]:0.3058655937)99:0.2240784243,bacFtsY@WP_088252003.1_Fimbriiglobus_ruber [Planctomycetes]:0.3874286992)75:0.1065482157,(bacFtsY@WP_041017695.1_Criblamydia_sequanensis [Chlamydiae]:0.5067648899,bacFtsY@PCI95412.1_Candidatus_Aerophobetes_bacterium [Aerophobetes]:0.4547388085)100:0.2048946788)77:0.0765987317,(((((((((((((bacFtsY@WP_107510123.1_Staphylococcus_fleurettii [Firmicutes]:0.1544800930,bacFtsY@AVK83142.1_Lysinibacillus_sp_B2A1 [Firmicutes]:0.1570756706)94:0.0846096003,((bacFtsY@WP_009554695.1_Lactobacillus_saerimneri [Firmicutes]:0.2318221145,bacFtsY@WP_009491663.1_Catellicoccus_marimammalium [Firmicutes]:0.1852121233)44:0.0276193763,((bacFtsY@WP_054646204.1_Lactobacillus_lindneri [Firmicutes]:0.0548362836,bacFtsY@WP_056961480.1_Lactobacillus_florum [Firmicutes]:0.1443008325)98:0.0820209775,bacFtsY@WP_103423367.1_Lactobacillus_sanfranciscensis [Firmicutes]:0.1258457438)100:0.1849883827)100:0.1749926725)83:0.0778462270,(bacFtsY@WP_069327418.1_Paenibacillus_sp_TI45-13ar [Firmicutes]:0.1938992150,(bacFtsY@WP_091834879.1_Marininema_halotolerans [Firmicutes]:0.1693382978,bacFtsY@WP_028778316.1_Shimazuella_kribbensis [Firmicutes]:0.1610095775)100:0.1127587157)65:0.0495415063)90:0.0730796549,(bacFtsY@WP_026974763.1_Alicyclobacillus_contaminans [Firmicutes]:0.4295501867,bacFtsY@PTQ57904.1_Candidatus_Carbobacillus_altaicus [Firmicutes]:0.3628843011)91:0.1140733159)97:0.0830601719,bacFtsY@WP_073092242.1_Thermosyntropha_lipolytica [Firmicutes]:0.3696118813)36:0.0281507058,(bacFtsY@KUO52399.1_Desulfitibacter_sp_BRH_c19 [Firmicutes]:0.4303878669,((bacFtsY@WP_072972532.1_Tissierella_praeacuta [Firmicutes]:0.2589956309,bacFtsY@WP_054252106.1_Neofamilia_massiliensis [Firmicutes]:0.3697255094)100:0.1757314279,(bacFtsY@CDA51269.1_Clostridium_sp_CAG-138 [Firmicutes]:0.4568950037,(((bacFtsY_RCL55122.1_signal_recognition_particle-docking_protein_FtsY_Synechococcus_sp._MED-G71:0.1055648327,(bacFtsY_WP_038650415.1_signal_recognition_particle-docking_protein_FtsY_Prochlorococcus_sp._MIT_0801:0.2147611454,chrFtsY@AUG32399.1_Paulinella_longichromatophora [Rhizaria]:0.1515316527)98:0.0913582501)100:0.2680073445,(bacFtsY_WP_111893930.1_signal_recognition_particle-docking_protein_FtsY_Arthrospira_sp._O9.13F:0.0708282569,bacFtsY@WP_015110998.1_Nostoc_sp [Cyanobacteria]:0.1242931767)99:0.1495465039)56:0.0703663068,bacFtsY_WP_081705397.1_signal_recognition_particle-docking_protein_FtsY_Gloeobacter_kilaueensis:0.2056919983)100:0.5007904420)80:0.1296628201)34:0.0000022634)33:0.0590264494)45:0.0413061002,bacFtsY@EEG77220.1_Dethiobacter_alkaliphilus_AHT_1 [Firmicutes]:0.3153145543)67:0.0625138145,(((bacFtsY@WP_038038136.1_Thermorudis_peleae [Chloroflexi]:0.4230745136,bacFtsY@OQY47814.1_Anaerolineaceae_bacterium_4572_78 [Chloroflexi]:0.5170802256)52:0.0750640440,(bacFtsY@RCK76206.1_Anaerolineae_bacterium [Chloroflexi]:0.3670255630,bacFtsY@KUK71287.1_Anaerolineae_bacterium_49_20 [Chloroflexi]:0.2894827293)100:0.2778600199)92:0.1055895730,bacFtsY@WP_009108227.1_Desulfovibrio_sp_U5L [Deltaproteobacteria]:0.5767447700)44:0.0536632722)16:0.0142125653,(bacFtsY@AEG15826.1_Desulfofundulus_kuznetsovii_DSM_6115 [Firmicutes]:0.2460773827,bacFtsY@WP_075860076.1_Carboxydothermus_pertinax [Firmicutes]:0.3203622462)70:0.1136672357)36:0.0410191917,bacFtsY@OGP30931.1_Deltaproteobacteria_bacterium_GWC2_42_11 [Deltaproteobacteria]:0.4840407172)43:0.0441720180,((((((((bacFtsY@OJX59420.1_Candidatus_Kapabacteria_thiocyanatum [Bacteroidetes]:0.1591480714,bacFtsY@PKL79980.1_Ignavibacteriae_bacterium_HGW-Ignavibacteriae-4 [Ignavibacteriae]:0.2884386046)100:0.1261559011,bacFtsY@KXK57805.1_Chlorobi_bacterium_OLB7 [Chlorobi]:0.2166440314)97:0.1044986204,bacFtsY@PLX30570.1_Ignavibacteria_bacterium [Ignavibacteriae]:0.1903830216)72:0.0344468961,(bacFtsY@WP_092350764.1_Candidatus_Chrysopegis_kryptomonas [Kryptonia]:0.1900692969,((bacFtsY@OGU83728.1_Ignavibacteria_bacterium_RBG_16_35_7 [Ignavibacteriae]:0.4608311076,bacFtsY@PKL82841.1_Ignavibacteriae_bacterium_HGW-Ignavibacteriae-3 [Ignavibacteriae]:0.4709785174)92:0.1252123879,bacFtsY@OQY74580.1_Ignavibacteriales_bacterium_UTCHB3 [Ignavibacteriae]:0.3185418867)100:0.2783155800)87:0.0831315344)84:0.0609405322,bacFtsY@OGU26318.1_Ignavibacteria_bacterium_GWA2_54_16 [Ignavibacteriae]:0.4095866734)60:0.0282948098,(bacFtsY@PSQ63899.1_Bacteroidetes_bacterium_QH_1_61_8 [Bacteroidetes]:0.3357808964,(((bacFtsY@OUV32974.1_Rhodothermaceae_bacterium_TMED105 [Bacteroidetes]:0.3741136288,bacFtsY@OUU18192.1_Crocinitomicaceae_bacterium_TMED45 [Bacteroidetes]:0.3488128912)47:0.0448160895,bacFtsY@WP_100314957.1_Thermoflavifilum_aggregans [Bacteroidetes]:0.2803602891)73:0.0634292741,((bacFtsY@OUV76101.1_Flavobacteriales_bacterium_TMED123 [Bacteroidetes]:0.2210467675,bacFtsY@PSR05731.1_Bacteroidetes_bacterium_SW_10_40_5 [Bacteroidetes]:0.3206147860)93:0.0680304207,(bacFtsY@WP_103327589.1_Bacteroidetes_endosymbiont_of_Geopemphigus_sp [Bacteroidetes]:0.2076733741,bacFtsY@WP_114910387.1_Cardinium_endosymbiont_of_Sogatella_furcifera [Bacteroidetes]:0.4345346128)81:0.0955922917)70:0.0353211004)76:0.0775473653)95:0.0858111801)98:0.1186342670,bacFtsY@OGC84905.1_Zixibacteria_bacterium_RBG_16_43_9 [Zixibacteria]:0.4214350505)67:0.0590380445,(((((bacFtsY@WP_083764053.1_Syntrophobacter_fumaroxidans [Deltaproteobacteria]:0.3491616097,bacFtsY@PIP45503.1_Deltaproteobacteria_bacterium_CG23_combo_of_CG06-09_8_20_14_all_51_20 [Deltaproteobacteria]:0.3071968984)63:0.0622504868,((bacFtsY@PIP06279.1_Syntrophobacteraceae_bacterium_CG23_combo_of_CG06-09_8_20_14_all_50_8 [Deltaproteobacteria]:0.1881438714,bacFtsY@ABC77884.1_Syntrophus_aciditrophicus_SB [Deltaproteobacteria]:0.2601648824)100:0.2438734265,bacFtsY@PIE60181.1_Desulfobulbus_propionicus [Deltaproteobacteria]:0.3356046033)69:0.0747040419)80:0.0446374171,(bacFtsY@WP_011985037.1_Anaeromyxobacter_sp_Fw109-5 [Deltaproteobacteria]:0.3713606503,(bacFtsY@PID38167.1_Proteobacteria_bacterium [Proterobacteria]:0.4436160142,bacFtsY@KPK53060.1_Myxococcales_bacterium_SG8_38_1 [Deltaproteobacteria]:0.5489529663)61:0.1024458180)89:0.1083171162)74:0.0654057044,((((((bacFtsY@WP_121469822.1_Edaphobacter_dinghuensis [Acidobacteria]:0.2007924273,(bacFtsY@WP_074656074.1_Terriglobus_roseus [Acidobacteria]:0.0170118230,bacFtsY@WP_081490725.1_Terriglobus_roseus [Acidobacteria]:0.0096063584)100:0.1673657868)100:0.3233548129,(bacFtsY@WP_031499580.1_Bryobacter_aggregatus [Acidobacteria]:0.1902360179,bacFtsY@WP_020721417.1_Acidobacteriaceae_bacterium_KBS_96 [Acidobacteria]:0.1720434340)98:0.1005761858)63:0.0650443060,bacFtsY@ANM28936.1_Acidobacteria_bacterium_Mor1 [Acidobacteria]:0.6342392281)76:0.0811325202,bacFtsY@PYS67814.1_Acidobacteria_bacterium [Acidobacteria]:0.3364499476)63:0.0667376184,bacFtsY@PIE91415.1_Acidobacteria_bacterium [Acidobacteria]:0.4849518612)89:0.1110180594,((((((((bacFtsY@KGA01103.1_Cobetia_amphilecti [Gammaproteobacteria]:0.0000027184,bacFtsY@WP_107335894.1_Halomonas_sp_SF2003 [Verrucomicrobia]:0.0000010099)100:0.0909341757,bacFtsY@WP_097790462.1_Halomonas_beimenensis [Verrucomicrobia]:0.1074708968)100:0.1091026384,(bacFtsY@WP_091826228.1_Marinobacterium_georgiense [Gammaproteobacteria]:0.1240877498,(bacFtsY@ARM82543.1_Marinobacter_salarius [Gammaproteobacteria]:0.0362903529,bacFtsY@KXS51830.1_Marinobacter_sp_T13-3 [Gammaproteobacteria]:0.0321211714)100:0.1165815381)97:0.0776240015)63:0.0422297452,(((bacFtsY@WP_094041167.1_Zobellella_denitrificans [Gammaproteobacteria]:0.0925017130,bacFtsY@WP_091986549.1_Pseudoalteromonas_denitrificans [Gammaproteobacteria]:0.1595666355)94:0.0534652497,bacFtsY@PPI88525.1_Pantoea_sp_SoEO [Gammaproteobacteria]:0.4853926118)65:0.0341004626,bacFtsY@WP_113743312.1_Anaerobiospirillum_thomasii [Gammaproteobacteria]:0.3657721353)82:0.0635367869)54:0.0282556776,bacFtsY@WP_116686709.1_contaminant of Flavobacteriaceae_bacterium Hp12 genome [Gammaproteobacteria]:0.2339406183)65:0.0579292716,bacFtsY@WP_086487265.1_Thioflexothrix_psekupsii [Gammaproteobacteria]:0.2375550287)82:0.0364653219,(bacFtsY@WP_095208401.1_Luteimonas_sp_JM171 [Gammaproteobacteria]:0.3693688318,bacFtsY@OUX68934.1_Oceanospirillales_bacterium_TMED91 [Gammaproteobacteria]:0.4517278517)55:0.1001375577)95:0.0876237129,((((bacFtsY@GBD43248.1_Bacterium_HR40 [unassigned]:0.4498122423,((bacFtsY@EPY01636.1_Phaeospirillum_fulvum_MGU-K5 [Alphaproteobacteria]:0.1805931824,(bacFtsY@OFX10022.1_Alphaproteobacteria_bacterium_RIFOXYD12_FULL_60_8 [Alphaproteobacteria]:0.1504466615,bacFtsY@WP_092615803.1_Roseospirillum_parvum [Alphaproteobacteria]:0.2714794200)68:0.0512925850)91:0.0951129721,(bacFtsY@OUU28495.1_Candidatus_Endolissoclinum_sp_TMED37 [Alphaproteobacteria]:0.3251751777,bacFtsY@AIL12880.1_Candidatus_Paracaedimonas_acanthamoebae [Alphaproteobacteria]:0.4417876201)60:0.1091725067)56:0.0572282362)23:0.0132734582,(bacFtsY@KRS17267.1_Roseovarius_indicus [Alphaproteobacteria]:0.0850423406,bacFtsY@WP_025048799.1_Sulfitobacter_mediterraneus [Alphaproteobacteria]:0.0773992925)100:0.3289419246)85:0.0339057554,((((((bacFtsY@WP_108880431.1_Anderseniella_sp_Alg231-50 [Alphaproteobacteria]:0.2402854383,bacFtsY@WP_109793879.1_Rhizobiales_bacterium [Alphaproteobacteria]:0.2797599482)88:0.0865837651,(((bacFtsY@SKC16052.1_Bosea_thiooxidans [Alphaproteobacteria]:0.1940273087,((bacFtsY@WP_011996071.1_Xanthobacter_autotrophicus [Alphaproteobacteria]:0.0656889867,bacFtsY@WP_024277252.1_Xanthobacter_sp_126 [Alphaproteobacteria]:0.0456019697)100:0.1260812945,bacFtsY@WP_013168132.1_Starkeya_novella [Alphaproteobacteria]:0.1400668821)98:0.0668323925)85:0.0431019400,bacFtsY@WP_115516251.1_Pseudolabrys_sp_GY_H [Alphaproteobacteria]:0.2799203394)81:0.0314554129,bacFtsY@WP_088520031.1_Rhodoblastus_acidophilus [Alphaproteobacteria]:0.1658747354)92:0.0474441057)79:0.0345020565,bacFtsY@WP_099557887.1_Hartmannibacter_diazotrophicus [Alphaproteobacteria]:0.1419417898)68:0.0314595381,bacFtsY@WP_029041189.1_Cucumibacter_marinus [Alphaproteobacteria]:0.2216668196)64:0.0329167568,((bacFtsY@WP_113333951.1_Rhizobiales_bacterium [Alphaproteobacteria]:0.1962713702,bacFtsY@RCL01761.1_Candidatus_Tokpelaia_sp_JSC085 [Alphaproteobacteria]:0.3059662320)91:0.1060285354,bacFtsY@PPD07903.1_Hyphomicrobium_sp [Alphaproteobacteria]:0.2345507475)45:0.0192871644)82:0.0876902403,(bacFtsY@ACT57804.1_Hirschia_baltica_ATCC_49814 [Alphaproteobacteria]:0.0000020266,bacFtsY@WP_083773102.1_Hirschia_baltica [Alphaproteobacteria]:0.0000026523)100:0.4484896691)86:0.0496957452)100:0.2332278080,bacFtsY@SME87908.1_Pseudobacteriovorax_antillogorgiicola [Deltaproteobacteria]:0.5914569834)87:0.1433488161)77:0.0696509033)53:0.0502148779)42:0.0388179626,bacFtsY@WP_012175138.1_Desulfococcus_oleovorans [Deltaproteobacteria]:0.4923451513)41:0.0303800946)29:0.0220000299)23:0.0306239382,(((bacFtsY@OLB22504.1_Nitrospirae_bacterium_13_2_20CM_2_63_8 [Nitrospirae]:0.5375646976,bacFtsY@EKD41878.1_uncultured_bacterium [unassigned]:0.6460513288)78:0.0715418333,bacFtsY@OYZ20489.1_Bdellovibrio_sp_28-41-41 [Deltaproteobacteria]:0.7381874785)43:0.0719186941,((((((mtFtsY@NA_Naegleria_gruberi [Heterolobosea]:0.0000025807,(mtFtsY@NA_Naegleria_fowleri [Heterolobosea]:0.0125309395,mtFtsY@NA_Naegleria_lovaniensis [Heterolobosea]:0.0124052932)100:0.0821210460)100:0.4888770346,(mtFtsY@NA_Neovahlkampfia_damariscottae [Heterolobosea]:0.4424753842,mtFtsY@CAMNT_0005204377-extended_Percolomonas_cosmopolitus_AE [Heterolobosea]:1.4819986805)73:0.1502440806)77:0.1185482554,(mtFtsY@CAMNT_0005246107_Percolomonas_cosmopolitus_WS [Heterolobosea]:0.3203112354,mtFtsY@NA_partial_Percolomonas_ex_Nitzchia_Cheng_2013 [Heterolobosea]:0.5399421457)99:0.3740590692)81:0.0987020018,(mtFtsY@NA_Heterolobosea_BB2 [Heterolobosea]:0.1964674751,mtFtsY@NA_Pharyngomonas_kirbyi [Heterolobosea]:0.4244090200)79:0.1375174332)82:0.1540179891,(((mtFtsY@NA_Ancoracysta-related_Colp-4b [Ancoracysta]:0.8079687260,mtFtsY@NA_partial_Goniomonas_pacifica [Cryptophyta]:1.2982853208)47:0.0891831229,mtFtsY@QUTJ01025227.1_Goniomonas_avonlea [Cryptophyta]:0.7205086698)55:0.0471637996,mtFtsY@NA_N-terminus_Ancoracysta_twista [Ancoracysta]:0.8080893866)83:0.1826363868)73:0.1473191362,mtFtsY@NA_1_Hemimastix_kukwesjijk [Hemimastigophora]:0.6320074183)87:0.2170024498)46:0.0445262406)39:0.0762237733,(((((bacFtsY@WP_056203513.1_Pelomonas_sp_Root1237 [Betaproteobacteria]:0.0791803930,bacFtsY@PZP35629.1_Roseateles_depolymerans [Betaproteobacteria]:0.0460797235)59:0.0224126670,bacFtsY@WP_089417744.1_Vitreoscilla_filiformis [Betaproteobacteria]:0.1271280813)50:0.0275072265,bacFtsY@WP_066336977.1_Azohydromonas_lata [Betaproteobacteria]:0.0983149877)58:0.0347860136,bacFtsY@OWQ45088.1_Mitsuaria_noduli [Betaproteobacteria]:0.0554858877)61:0.0383645748,bacFtsY@WP_124447410.1_Paucibacter_sp_KBW04 [Betaproteobacteria]:0.0307516612)100:0.5662579624)40:0.0127976368)100:0.2689747404,((((ptFtsY@CEM32712.1_Vitrella_brassicaformis_CCMP3155 [Alveolata]:0.3801685427,(((ptFtsY@EWM29383.1_Nannochloropsis_gaditana [Stramenopiles]:0.2803886502,ptFtsY@CBJ31918.1_Ectocarpus_siliculosus [Stramenopiles]:0.3818830554)83:0.0870196673,ptFtsY@XP_002296627.1_Thalassiosira_pseudonana_CCMP1335 [Stramenopiles]:0.5884891499)58:0.0566639455,ptFtsY@XP_009040860.1_Aureococcus_anophagefferens [Stramenopiles]:0.3648962013)40:0.0439836758)98:0.1448236555,(ptFtsY@XP_005714040.1_Chondrus_crispus [Archaeplastida]:0.1348322667,ptFtsY@PXF44704.1_Gracilariopsis_chorda [Archaeplastida]:0.1124312384)100:0.2690669491)69:0.0881217733,ptFtsY@XP_005537370.1_Cyanidioschyzon_merolae_strain_10D [Archaeplastida]:0.5763045746)58:0.0695131403,ptFtsY@XP_005705405.1_Galdieria_sulphuraria [Archaeplastida]:0.8390439556)100:0.1831631991)79:0.0698517812);

**Phylogenetic tree from Fig. 3A (RAxML, LG4X model, 500 rapid bootstraps, 452 OTUs)**

(((bacFtsY@RCL55122.1_Synechococcus_sp_MED-G71 [Cyanobacteria]:0.04263486517141837351,(bacFtsY@WP_038650415.1_Prochlorococcus_sp_MIT_080 [Cyanobacteria]:0.17140798288713679498,chrFtsY@AUG32399.1_Paulinella_longichromatophora [Rhizaria]:0.09409592413084590101)86:0.07898744571436895623)98:0.12268572508804875820,(bacFtsY@WP_086487265.1_Thioflexothrix_psekupsii [Gammaproteobacteria]:0.06811421638168735360,((bacFtsY@OUX68934.1_Oceanospirillales_bacterium_TMED91 [Gammaproteobacteria]:0.34225138688834294554,((bacFtsY@PID38167.1_Proteobacteria_bacterium [Proterobacteria]:0.33109143898966253428,((bacFtsY@KPK53060.1_Myxococcales_bacterium_SG8_38_1 [Deltaproteobacteria]:0.46952053869645354878,(((mtFtsY@NA_N-terminus_Ancoracysta_twista [Ancoracysta]:0.32418879974436276559,mtFtsY@NA_Ancoracysta-related_Colp-4b [Ancoracysta]:0.57491559607402387133)47:0.00000100000050002909,(mtFtsY@NA_partial_Goniomonas_pacifica [Cryptophyta]:0.80578930223142553047,mtFtsY@QUTJ01025227.1_Goniomonas_avonlea [Cryptophyta]:0.43335646925511994709)55:0.16347041947182777699)38:0.21707419078247999766,(mtFtsY@NA_1_Hemimastix_kukwesjijk [Hemimastigophora]:0.32045646240139136207,((mtFtsY@NA_Heterolobosea_BB2 [Heterolobosea]:0.14042695484554615359,mtFtsY@NA_Pharyngomonas_kirbyi [Heterolobosea]:0.28829955295409553928)81:0.16752970823572849168,((mtFtsY@NA_partial_Percolomonas_ex_Nitzchia_Cheng_2013 [Heterolobosea]:0.42526841975815465879,mtFtsY@CAMNT_0005246107_Percolomonas_cosmopolitus_WS [Heterolobosea]:0.27174689675243030029)97:0.25589314081519642929,(mtFtsY@NA_Neovahlkampfia_damariscottae [Heterolobosea]:0.51751957405013104818,(mtFtsY@NA_Naegleria_gruberi [Heterolobosea]:0.00000100000050002909,(mtFtsY@NA_Naegleria_lovaniensis [Heterolobosea]:0.01134809706214358338,mtFtsY@NA_Naegleria_fowleri [Heterolobosea]:0.00000100000050002909)88:0.06055078257784039103)100:0.24333320542648145812)66:0.14575481834888995292)42:0.08911083960393942349)57:0.14024819070583033342)42:0.07258852992727329867)20:0.11363896968387597608)0:0.01292654054417361961,(((bacFtsY@OUV32974.1_Rhodothermaceae_bacterium_TMED105 [Bacteroidetes]:0.26411317001309125851,(((bacFtsY@WP_103327589.1_Bacteroidetes_endosymbiont_of_Geopemphigus_sp [Bacteroidetes]:0.19214531157934933159,bacFtsY@WP_114910387.1_Cardinium_endosymbiont_of_Sogatella_furcifera [Bacteroidetes]:0.26209091044195670372)67:0.10669710691814592030,(bacFtsY@PSQ63899.1_Bacteroidetes_bacterium_QH_1_61_8 [Bacteroidetes]:0.22753395174299184678,bacFtsY@PSR05731.1_Bacteroidetes_bacterium_SW_10_40_5 [Bacteroidetes]:0.22258001749148076387)50:0.07439349890898241946)26:0.04143107275759518732,(bacFtsY@OUU18192.1_Crocinitomicaceae_bacterium_TMED45 [Bacteroidetes]:0.25970186427057023737,bacFtsY@WP_100314957.1_Thermoflavifilum_aggregans [Bacteroidetes]:0.15828140536447743436)37:0.03965023398672645749)24:0.05471062580655644547)51:0.08657425990662107051,((bacFtsY@OGC84905.1_Zixibacteria_bacterium_RBG_16_43_9 [Zixibacteria]:0.27847092887324204069,((((bacFtsY@ANM28936.1_Acidobacteria_bacterium_Mor1 [Acidobacteria]:0.49761626916885781657,bacFtsY@PYS67814.1_Acidobacteria_bacterium [Acidobacteria]:0.28606531453921518748)5:0.01935627899880847796,(bacFtsY@WP_031499580.1_Bryobacter_aggregatus [Acidobacteria]:0.18697063643033734470,(bacFtsY@WP_121469822.1_Edaphobacter_dinghuensis [Acidobacteria]:0.20385651797729972823,bacFtsY@WP_081490725.1_Terriglobus_roseus [Acidobacteria]:0.07896442941025620665)97:0.35283629577074121508)33:0.04331479375362677631)28:0.08812784877713669696,bacFtsY@PIE91415.1_Acidobacteria_bacterium [Acidobacteria]:0.29951552005659054512)19:0.12287243715261726706,((((bacFtsY@OJX59420.1_Candidatus_Kapabacteria_thiocyanatum [Bacteroidetes]:0.10462953141685596425,bacFtsY@PKL79980.1_Ignavibacteriae_bacterium_HGW-Ignavibacteriae-4 [Ignavibacteriae]:0.17599783568601540251)81:0.08578545731474773728,bacFtsY@KXK57805.1_Chlorobi_bacterium_OLB7 [Chlorobi]:0.15776188980521096972)57:0.05059483725546346650,(bacFtsY@PLX30570.1_Ignavibacteria_bacterium [Ignavibacteriae]:0.16370176555340792968,((bacFtsY@OQY74580.1_Ignavibacteriales_bacterium_UTCHB3 [Ignavibacteriae]:0.20251780501748919971,(bacFtsY@OGU83728.1_Ignavibacteria_bacterium_RBG_16_35_7 [Ignavibacteriae]:0.38635222703536709066,bacFtsY@PKL82841.1_Ignavibacteriae_bacterium_HGW-Ignavibacteriae-3 [Ignavibacteriae]:0.36543678823109931875)71:0.09352846354509095073)80:0.16615387543597978648,bacFtsY@WP_092350764.1_Candidatus_Chrysopegis_kryptomonas [Kryptonia]:0.09967426105101286071)59:0.06606490006516910207)7:0.00647044541457858502)39:0.05214131332593485713,bacFtsY@OGU26318.1_Ignavibacteria_bacterium_GWA2_54_16 [Ignavibacteriae]:0.30495847696047573283)22:0.03782831327406493277)0:0.03852275125189668498)0:0.03227514162757740696,(bacFtsY@WP_012175138.1_Desulfococcus_oleovorans [Deltaproteobacteria]:0.31436906075085313539,(((bacFtsY@EKD41878.1_uncultured_bacterium [unassigned]:0.51026436018768572822,bacFtsY@OLB22504.1_Nitrospirae_bacterium_13_2_20CM_2_63_8 [Nitrospirae]:0.41491068391529845050)36:0.15277736212832271345,(bacFtsY@KUO52399.1_Desulfitibacter_sp_BRH_c19 [Firmicutes]:0.32844716782176414105,((bacFtsY@OYZ20489.1_Bdellovibrio_sp_28-41-41 [Deltaproteobacteria]:0.51179791163959242883,(((bacFtsY@WP_089417744.1_Vitreoscilla_filiformis [Betaproteobacteria]:0.07839646382627736554,((bacFtsY@WP_056203513.1_Pelomonas_sp_Root1237 [Betaproteobacteria]:0.03220150005257011588,bacFtsY@PZP35629.1_Roseateles_depolymerans [Betaproteobacteria]:0.02284141013550828464)58:0.03505667911070813741,bacFtsY@WP_066336977.1_Azohydromonas_lata [Betaproteobacteria]:0.05703556367097853408)20:0.01744266685624739494)23:0.02252516961487701849,bacFtsY@OWQ45088.1_Mitsuaria_noduli [Betaproteobacteria]:0.03156183062219246327)36:0.04622354457294085284,bacFtsY@WP_124447410.1_Paucibacter_sp_KBW04 [Betaproteobacteria]:0.00494055547134936764)100:0.40009256620787725378)10:0.10305590109071455229,(((((ptFtsY@XP_005714040.1_Chondrus_crispus [Archaeplastida]:0.05866919828584851032,ptFtsY@PXF44704.1_Gracilariopsis_chorda [Archaeplastida]:0.10050631348727555314)100:0.23912094372761238947,(ptFtsY@XP_002296627.1_Thalassiosira_pseudonana_CCMP1335 [Stramenopiles]:0.36629314553573816271,(ptFtsY@CEM32712.1_Vitrella_brassicaformis_CCMP3155 [Alveolata]:0.25608854177680839825,(ptFtsY@XP_009040860.1_Aureococcus_anophagefferens [Stramenopiles]:0.20646556451217570882,(ptFtsY@EWM29383.1_Nannochloropsis_gaditana [Stramenopiles]:0.23645044076702684266,ptFtsY@CBJ31918.1_Ectocarpus_siliculosus [Stramenopiles]:0.27573131062521483159)43:0.06995009884909103581)26:0.02305067896036571556)38:0.04467604819947210842)58:0.07105732899875104314)48:0.08310380001053742360,(ptFtsY@XP_005537370.1_Cyanidioschyzon_merolae_strain_10D [Archaeplastida]:0.46015363119785873680,ptFtsY@XP_005705405.1_Galdieria_sulphuraria [Archaeplastida]:0.58832408919934553460)38:0.06290880159903869395)96:0.26349719441925173458,(((ptFtsY@NP_566056.1_Arabidopsis_thaliana [Archaeplastida]:0.04396746204880945530,(ptFtsY@XP_021817084.1_Prunus_avium [Archaeplastida]:0.04002132290189777675,(ptFtsY@XP_023879170.1_Quercus_suber [Archaeplastida]:0.00000100000050002909,(ptFtsY@KHF98418.1_Cell_division_FtsY_chloroplastic-like_protein_Gossypium_arboreum [Archaeplastida]:0.01204228097311002207,ptFtsY@XP_010055217.1_Eucalyptus_grandis [Archaeplastida]:0.05637605135955908037)47:0.01537956333572873709)46:0.01115829123887283891)61:0.00804134495486424385)100:0.18741779151372350842,(ptFtsY@XP_003080532.1_Signal-recognition_particle_receptor_FtsY_Ostreococcus_tauri:0.31438458480414077645,(ptFtsY@XP_011399720.1_Signal_recognition_particle_receptor_FtsY_Auxenochlorella_protothecoides:0.25002084732210383411,((ptFtsY@XP_001697752.1_chloroplast_SRP_receptor_Chlamydomonas_reinhardtii:0.28314702053155671457,ptFtsY@XP_005651149.1_cell_division_transporter_substrate-binding_protein_FtsY_Coccomyxa_subellipsoidea_C-169:0.13078301341018222503)60:0.08785325511325917103,ptFtsY@PRW61060.1_cell_division_chloroplastic_isoform_A_Chlorella_sorokiniana:0.09809291483240920040)25:0.01852106179479416984)43:0.04844575025033458909)67:0.05316383387497650853)98:0.13368105857051279517,ptFtsY@MMETSP0308_Transcript_18196_m19230_Gloeochaete_wittrockiana [Archaeplastida]:0.39491323053800858611)66:0.02603110302371232712)74:0.13857674338796038827,bacFtsY@WP_011985037.1_Anaeromyxobacter_sp_Fw109-5 [Deltaproteobacteria]:0.26866744874956766109)8:0.01759545867685960094)0:0.04103084023862176716)0:0.01413494078515769789)0:0.03357812785325499372,((((bacFtsY@RPG16414.1_FtsY_Phycisphaera_sp_TMED9 [Planctomycetes]:0.29554223659786665879,bacFtsY@RMH26650.1_Planctomycetes_bacterium [Planctomycetes]:0.31902971933401425009)89:0.17684620572195144939,bacFtsY@WP_088252003.1_Fimbriiglobus_ruber [Planctomycetes]:0.36092615062152305638)39:0.12201028136956204584,(bacFtsY@WP_041017695.1_Criblamydia_sequanensis [Chlamydiae]:0.40658894428342556848,bacFtsY@PCI95412.1_Candidatus_Aerophobetes_bacterium [Aerophobetes]:0.35736625078761657948)54:0.15188988517053109151)4:0.08107777582522310289,(((bacFtsY@OGP30931.1_Deltaproteobacteria_bacterium_GWC2_42_11 [Deltaproteobacteria]:0.31979604079027135199,((bacFtsY@SME87908.1_Pseudobacteriovorax_antillogorgiicola [Deltaproteobacteria]:0.37776533000953393948,(bacFtsY@GBD43248.1_Bacterium_HR40 [unassigned]:0.29964108864811772603,((bacFtsY@KRS17267.1_Roseovarius_indicus [Alphaproteobacteria]:0.05104498607744316352,bacFtsY@WP_025048799.1_Sulfitobacter_mediterraneus [Alphaproteobacteria]:0.03282511242012874086)99:0.16636431148203620345,((((bacFtsY@EPY01636.1_Phaeospirillum_fulvum_MGU-K5 [Alphaproteobacteria]:0.12305826458634555598,bacFtsY@OFX10022.1_Alphaproteobacteria_bacterium_RIFOXYD12_FULL_60_8 [Alphaproteobacteria]:0.12611500165725486600)39:0.05494180046429288783,bacFtsY@WP_092615803.1_Roseospirillum_parvum [Alphaproteobacteria]:0.18515241010126312626)22:0.03069003526350056951,(bacFtsY@OUU28495.1_Candidatus_Endolissoclinum_sp_TMED37 [Alphaproteobacteria]:0.21816505920775594585,bacFtsY@AIL12880.1_Candidatus_Paracaedimonas_acanthamoebae [Alphaproteobacteria]:0.36927246743464264833)31:0.08430005966178930055)24:0.06492244796665724849,((bacFtsY@WP_109793879.1_Rhizobiales_bacterium [Alphaproteobacteria]:0.16066994291202810197,bacFtsY@WP_108880431.1_Anderseniella_sp_Alg231-50 [Alphaproteobacteria]:0.24151091367107227481)43:0.04936303190467814656,(bacFtsY@WP_029041189.1_Cucumibacter_marinus [Alphaproteobacteria]:0.12769780297548627424,((bacFtsY@PPD07903.1_Hyphomicrobium_sp [Alphaproteobacteria]:0.19594055040142149826,(bacFtsY@WP_113333951.1_Rhizobiales_bacterium [Alphaproteobacteria]:0.11249389726224999497,bacFtsY@RCL01761.1_Candidatus_Tokpelaia_sp_JSC085 [Alphaproteobacteria]:0.24486304936836755797)91:0.09565499875719155054)29:0.04292392841621522209,(bacFtsY@WP_099557887.1_Hartmannibacter_diazotrophicus [Alphaproteobacteria]:0.08755315326098610240,((bacFtsY@WP_115516251.1_Pseudolabrys_sp_GY_H [Alphaproteobacteria]:0.19159096006829601233,((bacFtsY@WP_083773102.1_Hirschia_baltica [Alphaproteobacteria]:0.00000100000050002909,bacFtsY@ACT57804.1_Hirschia_baltica_ATCC_49814 [Alphaproteobacteria]:0.00000100000050002909)100:0.38161689575341789515,bacFtsY@WP_088520031.1_Rhodoblastus_acidophilus [Alphaproteobacteria]:0.13022855704521435483)14:0.02561087018297894882)14:0.03962062381791325166,(bacFtsY@SKC16052.1_Bosea_thiooxidans [Alphaproteobacteria]:0.14559311375229391783,(bacFtsY@WP_024277252.1_Xanthobacter_sp_126 [Alphaproteobacteria]:0.08962720244317010088,bacFtsY@WP_013168132.1_Starkeya_novella [Alphaproteobacteria]:0.10835734278160180644)70:0.04897476256426281510)49:0.05384869785585522095)9:0.02911624764726782402)3:0.00754502616316303498)2:0.01838539954242956326)14:0.05967643822040763490)38:0.09739022641976614880)16:0.04504376991787998469)39:0.04556591704641626411)93:0.17471422818299861546)55:0.11069453898289191573,(bacFtsY@WP_075860076.1_Carboxydothermus_pertinax [Firmicutes]:0.24780319643678538899,bacFtsY@WP_009108227.1_Desulfovibrio_sp_U5L [Deltaproteobacteria]:0.42074125310027310487)14:0.02747803281965699451)4:0.04039134557179545337)2:0.05819656748259561391,(((((archFtsY@RLI30149.1_Candidatus_Bathyarchaeota_archaeon [TACK]:0.31763141476315415179,((archFtsY@WP_048122758.1_Methanosarcina_vacuolata [Euryarchaeota]:0.19770005509653576503,(archFtsY@WP_006182963.1_Natrinema_pellirubrum [Euryarchaeota]:0.13707146052618413012,(archFtsY@WP_004045232.1_Haloferax_volcanii [Euryarchaeota]:0.05623428345172967052,(archFtsY@WP_114604860.1_Haloplanus_sp._CBA1112 [Euryarchaeota]:0.09561207135188749817,(archFtsY@WP_050034148.1_Halorubrum_halophilum [Euryarchaeota]:0.07896122926351653581,archFtsY@WP_021073086.1_Haloarchaeon_3A1_DGR [Euryarchaeota]:0.05088246823026306570)84:0.06529907064454634757)60:0.05668077592425409261)44:0.02556071618334555817)100:0.27751517095797267887)92:0.11438754330042900931,((eukSRa@AAD11975.1_Giardia_intestinalis [Metamonada]:0.53645018550682321834,(eukSRa@XP_012894318.1_Blastocystis_hominis [Stramenopiles]:0.17160250163424350944,((((eukSRa@CBJ30645.1_Ectocarpus_siliculosus [Stramenopiles]:0.12486967397470805086,((eukSRa@GAX97406.1_Pythium_insidiosum [Stramenopiles]:0.06425774491872357497,(eukSRa@XP_002906671.1_Phytophthora_infestans_T30-4 [Stramenopiles]:0.01454374263427925251,eukSRa@RLN51388.1_Phytophthora_kernoviae [Stramenopiles]:0.01243460263705451797)93:0.04916706311595871182)100:0.11850046667001892797,eukSRa@XP_005853460.1_Nannochloropsis_gaditana_CCMP526 [Stramenopiles]:0.16578572257847931648)35:0.04182011901051108094)83:0.12437704559270625071,(((((eukSRa@CAMNT_0031806435_Goniomonas_pacifica [Cryptophyta]:0.03607241765601880423,eukSRa@GGUN01044729.1_Goniomonas_avonlea [Cryptophyta]:0.06372483508880792680)99:0.17510282037798249877,eukSRa@GFYU01005410.1_Ancoracysta_twista [Ancoracysta]:0.13653724900931279995)32:0.06210034201999171249,((eukSRa@MMETSP0308_Transcript_27849_m29213_Gloeochaete_wittrockiana [Archaeplastida]:0.14457194735027881216,((eukSRa@GBG74478.1_Chara_braunii [Archaeplastida]:0.04750814978301481223,((eukSRa@ONL92607.1_Zea_mays [Archaeplastida]:0.02370097842697516458,(((eukSRa@XP_003527179.1_Glycine_max [Archaeplastida]:0.00000100000050002909,eukSRa@XP_021634617.1_Manihot_esculenta [Archaeplastida]:0.00000100000050002909)81:0.00000100000050002909,eukSRa@XP_022750268.1_Durio_zibethinus [Archaeplastida]:0.01096176443905574199)84:0.00619516396114932078,eukSRa@XP_020873914.1_Arabidopsis_lyrata_subsp_lyrata [Archaeplastida]:0.02725641171104279184)49:0.01005879792921367602)63:0.02215808132823621907,eukSRa@XP_024403926.1_Physcomitrella_patens [Archaeplastida]:0.09850414370196168556)61:0.03325584915943288755)50:0.02324939379273662649,(eukSRa@CAMPEP_0191492328_Pyramimonas-parkeae-CCMP726 [Archaeplastida]:0.16457080125973932994,(eukSRa@OUS44851.1_Ostreococcus_tauri [Archaeplastida]:0.35588937198980463705,(eukSRa@XP_005848975.1_Chlorella_variabilis [Archaeplastida]:0.05208925859501376682,(eukSRa@XP_001692081.1_Chlamydomonas_reinhardtii [Archaeplastida]:0.01444232647900376386,(eukSRa@KXZ55716.1_Gonium_pectorale [Archaeplastida]:0.01616183207225192070,eukSRa@XP_002949444.1_Volvox_carteri_nagariensis [Archaeplastida]:0.01554510338511344222)62:0.01760183715900136975)100:0.09429168053527325899)97:0.06030863473279646919)32:0.02279451158172456776)36:0.01447960709321584501)80:0.09414969411034157520)25:0.04472887340827488845,(((((eukSRa@ESS33331.1_Toxoplasma_gondii [Alveolata]:0.01156794119327487352,eukSRa@CEL66130.1_Neospora_caninum [Alveolata]:0.00551741762908708293)100:0.18082660345129855650,eukSRa@SBT01585.1_Plasmodium_malariae [Alveolata]:0.19090652382133835308)31:0.05332570521429592231,(eukSRa@CAMPEP_0170537274_Litonotus_pictus_Strain_P1 [Alveolata]:0.23358165004248795937,eukSRa@XP_004029895.1_Ichthyophthirius_multifiliis [Alveolata]:0.24700709891415453812)60:0.02524970849309871382)23:0.07026999274576357923,eukSRa@GECH01003431.1_Pharyngomonas_kirbyi [Heterolobosea]:0.19194176906523693904)8:0.05912097403316846772,(((eukSRa@KYQ94416.1_Tieghemostelium_lacteum [Amoebozoa]:0.10379389676632486128,eukSRa@XP_012757500.1_Acytostelium_subglobosum_LB1 [Amoebozoa]:0.09283358290340835983)98:0.16464651741175626021,eukSRa@NA_Neovahlkampfia_damariscottae [Heterolobosea]:0.17358870438623180688)39:0.09292784026650664775,(((eukSRa@XP_002670075.1_Naegleria_gruberi [Heterolobosea]:0.00000100000050002909,eukSRa@NF0122080_p1_Naegleria_fowleri [Heterolobosea]:0.03899027888195923802)99:0.17125402052496052629,

eukSRa@CAMNT_0005204115_Percolomonas_cosmopolitus_AE [Heterolobosea]:0.33599928497340958433,eukSRa@CAMNT_0005228041_Percolomonas_cosmopolitus_strain_WS [Heterolobosea]:0.14918647703571097374)80:0.12605822453002957229)37:0.07565227452132275432,eukSRa@GEZU01029986.1_GEZU01002276.1_Heterolobosea_BB2 [Heterolobosea]:0.10933189930497275166)12:0.03936822121270103919)4:0.01862372388796743050)0:0.01979554909017557443)0:0.03542506543601822711)0:0.00000100000050002909,((eukSRa@NP_001171313.1_Homo_sapiens [Opisthokonta]:0.23285373141962809052,eukSRa@NA_Hemimastix_kukwesjijk [Hemimastigophora]:0.19345148319166083350)16:0.02064992955571083935,(eukSRa@XP_010703301.1_Leishmania_panamensis [Euglenozoa]:0.04255628731224945366,(eukSRa@EPY25999.1_Angomonas_deanei [Euglenozoa]:0.04032852728198314113,eukSRa@EKF29134.1_Trypanosoma_cruzi_marinkellei [Euglenozoa]:0.04375464740106810996)57:0.02662991531344680723)100:0.31912689053829662589)8:0.05168626475330646147)0:0.03948894376735143097,eukSRa@ANM86232.1_Stygiella_incarcerata [Jakobida]:0.24164186786944838459)2:0.03340886700668640324)3:0.02784285882015649985,(eukSRa@GBE59094.1_Babesia_ovata [Alveolata]:0.22383781864367882641,eukSRa@POM83525.1_Cryptosporidium_meleagridis [Alveolata]:0.22171258165116206063)58:0.09424240752226535622)6:0.03649216668826568699,eukSRa@KOO32508.1_Chrysochromulina_sp_CCMP291 [Haptophyta]:0.25537590571839463571)13:0.10154072701249822197)26:0.17532813495866222953)100:0.50486972887063963356,(archFtsY@OIO41360.1_Candidatus_Pacearchaeota_archaeon_CG1_02_31_27 [DPANN]:0.32232875807826139036,archFtsY@PIZ51778.1_Candidatus_Woesearchaeota_archaeon_CG_4_10_14_0_2_um_filter_33_13 [DPANN]:0.24898456623260353804)99:0.20032971669981203267)38:0.07663208592004207287)21:0.02564273622413983100)8:0.03802723179377167501,(((archFtsY@RMF91339.1_Euryarchaeota_archaeon [Euryarchaeota]:0.33615422315860027380,archFtsY@OYT54953.1_Candidatus_Altiarchaeales_archaeon_ex4484_2 [DPANN]:0.26335384867620259541)57:0.13269660839263006102,(archFtsY@WP_014122151.1_Thermococcus_sp._AM4 [Euryarchaeota]:0.09686399721282208664,archFtsY@WP_011012907.1_Pyrococcus_furiosus [Euryarchaeota]:0.07731076320221705322)100:0.23166931609458243924)25:0.04711567121556967108,archFtsY@RLI57948.1_Candidatus_Thorarchaeota_archaeon [Asgard]:0.32840647457122879027)11:0.04454649636352905895)100:0.39100253841464288618,(((archSRP54@RLF74017.1_Thermoplasmata_archaeon [Euryarchaeota]:0.29700004017571779436,((archSRP54@WP_079234317.1_Halolamina_sp_CBA1230 [Euryarchaeota]:0.07879366304199909854,((archSRP54@Q977V2_Haloferax_volcanii [Euryarchaeota]:0.06807387956321148126,(archSRP54@WP_049990021.1_Halopiger_salifodinae [Euryarchaeota]:0.00000100000050002909,(archSRP54@WP_005578715.1_Natronobacterium_gregoryi [Euryarchaeota]:0.05391018938979972447,archSRP54@WP_066299325.1_Haloterrigena_mahii [Euryarchaeota]:0.01232412192357745448)56:0.01779565227605101052)100:0.09570554072963294301)24:0.01875367432015901184,archSRP54@WP_092813433.1_Halopenitus_malekzadehii [Euryarchaeota]:0.07763586018275986378)38:0.01748379393938735704)100:0.33149863065208717838,(archSRP54@WP_014405995.1_Methanocella_conradii [Euryarchaeota]:0.13204634353595828822,(archSRP54@WP_011449961.1_Methanospirillum_hungatei [Euryarchaeota]:0.32659231124436849392,(archSRP54@WP_095645771.1_Methanosarcina_spelaei [Euryarchaeota]:0.11120686145049811511,archSRP54@KXS43658.1_Methanolobus_sp_T82-4 [Euryarchaeota]:0.11958854642553087833)65:0.02863580439531157595)38:0.02692213937454882022)37:0.02587294740906661422)98:0.14836616974079677722)94:0.10465532051930272195,((eukSRP54@CAMNT_0031816087_Goniomonas_pacifica [Cryptophyta]:0.41021857343526269002,(((((eukSRP54@NA_Neovahlkampfia_damariscottae [Heterolobosea]:0.19018008089508570135,(eukSRP54@GFYU01001334.1_Ancoracysta_twista [Ancoracysta]:0.18839141093436206909,((eukSRP54@XP_024367976.1_Physcomitrella_patens [Archaeplastida]:0.05236329924456763207,(eukSRP54@PON48078.1_Parasponia_andersonii [Archaeplastida]:0.00649665373254630987,(eukSRP54@XP_006427317.1_Citrus_clementina [Archaeplastida]:0.00371493354094261331,(eukSRP54@XP_008454178.1_Cucumis_melo [Archaeplastida]:0.02897959917497722540,eukSRP54@XP_022724150.1_Durio_zibethinus [Archaeplastida]:0.00370661103882474275)53:0.00711379251808870808)83:0.01512308135825037730)99:0.06719355419598517509)99:0.13809170193692560380,((eukSRP54@MMETSP0308_Transcript_29267_m30521_Gloeochaete_wittrockiana [Archaeplastida]:0.20785630158450196792,(eukSRP54@GGUN01039927.1_Goniomonas_avonlea [Cryptophyta]:0.05021158167558255336,eukSRP54@CAMNT_0031843689_Goniomonas_pacifica [Cryptophyta]:0.05460800314706931724)98:0.05052696429759022390)43:0.04193425942801531220,(eukSRP54@XP_004367866.1_Acanthamoeba_castellanii_strain_Neff [Amoebozoa]:0.17472090059389044914,((eukSRP54@EPZ32142.1_Rozella_allomycis_CSF55 [Opisthokonta]:0.18747737918709492244,(eukSRP54@XP_016608410.1_Spizellomyces_punctatus_DAOM_BR117 [Opisthokonta]:0.10255972721635046863,(eukSRP54@RDX56745.1_Polyporus_brumalis [Opisthokonta]:0.02431304235649427853,(eukSRP54@KXN86887.1_Leucoagaricus_sp_SymC_cos [Opisthokonta]:0.01888689913652537647,(eukSRP54@PBK96926.1_Armillaria_gallica [Opisthokonta]:0.00362253284935066871,eukSRP54@KNZ71807.1_Termitomyces_sp_J132 [Opisthokonta]:0.02326374612586490712)76:0.01265655177111051945)84:0.02590362020445981933)100:0.28167588718306169060)79:0.05132275836086947624)75:0.05306099357782104869,((eukSRP54@XP_023304254.1_Lucilia_cuprina [Opisthokonta]:0.01062877608872998987,(eukSRP54@XP_002068627.1_Drosophila_willistoni [Opisthokonta]:0.00704873408072543076,(eukSRP54@SPP87160.1_Drosophila_guanche [Opisthokonta]:0.00000100000050002909,eukSRP54@XP_017138354.1_Drosophila_miranda [Opisthokonta]:0.00000100000050002909)98:0.00705715383500964827)79:0.01434250748860917482)75:0.01435809797876302051,(eukSRP54@XP_021695011.1_Aedes_aegypti [Opisthokonta]:0.00872769193039253134,eukSRP54@ETN65735.1_Anopheles_darlingi [Opisthokonta]:0.00830963225864364899)94:0.03043302276811541904)100:0.17541158477104615288)72:0.05236740047566004058)31:0.04663417420454951667)4:0.01366652124424261663)3:0.02264938214671363692)7:0.04261179085690550844)12:0.04887777891777624756,((eukSRP54@GEZU01011656.1_Heterolobosea_BB2 [Heterolobosea]:0.15078759762967550162,eukSRP54@GECH01013817.1_Pharyngomonas_kirbyi [Heterolobosea]:0.21592369108199915839)57:0.05721902836492131356,((eukSRP54@ACER01000537.1_Naegleri_gruberi [Heterolobosea]:0.44441247997682842286,(eukSRP54@NF0102970_p1_Naegleria_fowleri [Heterolobosea]:0.02969814969184125103,eukSRP54@XP_002671296.1-corrected_Naegleria_gruberi [Heterolobosea]:0.05896605344994468312)97:0.09057916152139507127)93:0.10290638390908891930,eukSRP54@ANM86233.1_Stygiella_incarcerata [Jakobida]:0.28571496480010544206)38:0.03684485391496580831)22:0.02910517965772266985)3:0.02927825135145321889,eukSRP54@NA_Spironema_sp [Hemimastigophora]:0.26779146727203118061)12:0.03561807425551011957,(eukSRP54@XP_004039207.1_Ichthyophthirius_multifiliis [Alveolata]:0.11033774993840499146,eukSRP54@XP_001018396.1_Tetrahymena_thermophila_SB210 [Alveolata]:0.08747250072233149487)100:0.15516194645860412193)37:0.05255146589363646897,((eukSRP54@RAW42240.1_Phytophthora_cactorum [Stramenopiles]:0.00356657522054031279,eukSRP54@XP_008893053.1_Phytophthora_parasitica_INRA-310 [Stramenopiles]:0.00000100000050002909)100:0.16385276514322155705,((eukSRP54@OEU17714.1_Fragilariopsis_cylindrus_CCMP1102 [Stramenopiles]:0.09281006273399769468,eukSRP54@XP_002185775.1_Phaeodactylum_tricornutum_CCAP1055-1 [Stramenopiles]:0.13149427372812197423)92:0.06177173003581122590,eukSRP54@CBN75372.1_Ectocarpus_siliculosus [Stramenopiles]:0.15981136292152034462)89:0.06275148468348516750)83:0.10926155623117575488)49:0.06310539502839204729)100:0.19110699046752527286,((((archSRP54@WP_110270916.1_Acidianus_brierleyi [TACK]:0.35790759018269835412,archSRP54@RLF14065.1_Thermoprotei_archaeon [TACK]:0.23617266249900548125)60:0.07743460104572341685,archSRP54@RLE51417.1_Candidatus_Verstraetearchaeota_archaeon [TACK]:0.15471142876244473041)56:0.06238057548063159763,archSRP54@OLS17826.1_Candidatus_Odinarchaeota_archaeon LCB4 [Asgard]:0.29938443047838586564)66:0.07628418937161640656,(archSRP54@WP_014734678.1_Pyrococcus_sp_ST04 [Euryarchaeota]:0.04985424368950414076,(archSRP54@WP_087037458.1_Thermococcus_litoralis [Euryarchaeota]:0.04587411458766715855,(archSRP54@WP_062370762.1_Thermococcus_guaymasensis [Euryarchaeota]:0.05594909966886334185,archSRP54@WP_088864111.1_Thermococcus_barossii [Euryarchaeota]:0.04691323117577526902)91:0.04383556455209344088)72:0.02657798605662087602)100:0.12906581615315271261)56:0.05783725334492664794)44:0.03779173982510634910)100:0.31261939939555655199,(((bacFfh@WP_013275867.1_Thermosediminibacter_oceani [Firmicutes]:0.14699683541869404402,(((((bacFfh@CDC00866.1_Firmicutes_bacterium_CAG_41 [Firmicutes]:0.22182314066321726087,bacFfh@WP_072469189.1_Urinacoccus_massiliensis [Firmicutes]:0.22450113652581812906)20:0.04533342779177430837,(((bacFfh@WP_074910001.1_Proteiniclasticum_ruminis [Firmicutes]:0.19743862651005120168,bacFfh@WP_008908195.1_Caloramator_australicus [Firmicutes]:0.12236331657211245438)66:0.06353619403984271663,bacFfh@WP_054875147.1_Oxobacter_pfennigii [Firmicutes]:0.15335806737250545773)36:0.04558973300375124271,(bacFfh@WP_058486324.1_Defluviitalea_phaphyphila [Firmicutes]:0.14089154239839488114,((bacFfh@WP_022786100.1_Clostridiales_bacterium_NK3B98 [Firmicutes]:0.27059507488145784215,bacFfh@WP_117520369.1_Ruminococcus_sp_AF43-11 [Firmicutes]:0.16738778832856099354)40:0.06823097544728064234,(bacFfh@PKL00150.1_Tenericutes_bacterium_HGW-Tenericutes-1 [Tenericutes]:0.30589368820482837341,(bacFfh@WP_015076211.1_Carnobacterium_maltaromaticum [Firmicutes]:0.12424463359295298548,(bacFfh@WP_086120482.1_Lactobacillus_reuteri [Firmicutes]:0.18142487159975964617,bacFfh@WP_050441216.1_Streptococcus_pneumoniae [Firmicutes]:0.23834515735551414783)65:0.05481922485694606245)77:0.05994632084843134340)60:0.06110056210313361685)10:0.05399384641018865200)5:0.02639690561461674814)1:0.02629559910026958020)2:0.02952466479447947781,(bacFfh@PWM50529.1_Clostridiales_bacterium [Firmicutes]:0.17209596706130300681,bacFfh@KJS18981.1_Clostridiaceae_bacterium_BRH_c20a [Firmicutes]:0.13383346780543714516)60:0.06351467661194822034)1:0.03495037571717306973,(((bacFfh@WP_116552983.1_Pueribacillus_theae [Firmicutes]:0.08308362733566890257,bacFfh@WP_109984449.1_Gracilibacillus_dipsosauri [Firmicutes]:0.14096637935676198383)73:0.09943788886319096521,bacFfh@WP_007505027.1_Caldalkalibacillus_thermarum [Firmicutes]:0.07029771610473303789)34:0.04106388252092040697,(bacFfh@WP_089967246.1_Lihuaxuella_thermophila [Firmicutes]:0.15622033743320026811,((bacFfh@WP_015891890.1_Brevibacillus_brevis [Firmicutes]:0.09840365093986234990,bacFfh@WP_120461224.1_Paenibacillus_aceti [Firmicutes]:0.12931079077322729654)54:0.02517030222854750587,bacFfh@WP_038091674.1_Tumebacillus_flagellatus [Firmicutes]:0.11667609991306443495)17:0.01554515869473600299)8:0.03171945790149032635)17:0.03452719188904673775)1:0.02270782588465581828,(bacFfh@WP_018702437.1_Anaeromusa_acidaminophila [Firmicutes]:0.11560249818762781404,(bacFfh@WP_046498292.1_Syntrophomonas_zehnderi [Firmicutes]:0.15244547276929074076,bacFfh@WP_106004935.1_Moorella_humiferrea [Firmicutes]:0.14075575291196570915)44:0.04238420054578798807)9:0.02640661621088844641)2:0.03638837520605559678)1:0.02804089064076679960,(bacFfh@WP_109430892.1_Acidibacillus_sulfuroxidans [Firmicutes]:0.25969984367016168969,(bacFfh@OPL10543.1_Firmicutes_bacterium_ML8_F2 [Firmicutes]:0.27273581471766106255,bacFfh@WP_072905435.1_Anaerobranca_californiensis [Firmicutes]:0.15707871933015407628)20:0.03495394016746494054)8:0.03207866779304491267)2:0.01892310460513659323,(((bacFfh@EFH87465.1_Ktedonobacter_racemifer_DSM_44963 [Chloroflexi]:0.38291976244948555674,bacFfh@WP_054491650.1_Ardenticatena_maritima [Chloroflexi]:0.15449931199365984913)31:0.08722890962649905433,(bacFfh@ACX52518.1_Ammonifex_degensii_KC4 [Firmicutes]:0.12374300708843714858,bacFfh@OIP71264.1_Candidatus_Atribacteria_bacterium_CG2_30_33_13 [Atribacteria]:0.58697602692935624269)10:0.02865423747644562855)5:0.05104199777764257401,(((((bacFfh@PIQ24328.1_Candidatus_Blackallbacteria [unassigned]:0.20677927092991760238,bacFfh@PCJ61345.1_Planctomycetes_bacterium [Planctomycetes]:0.28976381622400887439)64:0.10900411402255967552,(bacFfh@OJX56912.1_Candidatus_Kapabacteria_thiocyanatum [Bacteroidetes]:0.23250323051376711736,(bacFfh@WP_011429947.1_Synechococcus_sp [Cyanobacteria]:0.07225807039276499477,(((bacFfh@WP_081914739.1_Leptolyngbya_sp [Cyanobacteria]:0.06713883175054292940,(((bacFfh@WP_009785362.1_Lyngbya_sp_PCC_8106 [Cyanobacteria]:0.08101071354908918887,(bacFfh@A8YEA3_Microcystis_aeruginosa_PCC_7806 [Cyanobacteria]:0.06244565594748065218,bacFfh@KFF41240.1_Candidatus_Atelocyanobacterium_thalassa_isolate_SIO64986 [Cyanobacteria]:0.16991843191697914017)81:0.06655543872977891240)36:0.02263022749014274512,bacFfh@PSP17129.1_Cyanobacteria_bacterium_QS_8_64_29 [Cyanobacteria]:0.19589999487775672238)18:0.02567203530825180879,(bacFfh@WP_011612100.1_Trichodesmium_erythraeum [Cyanobacteria]:0.08593602053407400909,bacFfh@0A2P8WK82_Filamentous_cyanobacterium_CCP5 [Cyanobacteria]:0.07259019884456810456)22:0.02626989735412526769)3:0.00587672410841668259)12:0.02897352490272952358,(bacFfh@WP_015124913.1_Synechococcus_sp_PCC_6312 [Cyanobacteria]:0.08859838103230012474,bacFfh@RMH67925.1_Cyanobacteria_bacterium_J003 [Cyanobacteria]:0.05282897302498633374)43:0.02694569785335186596)25:0.06116880801735457424,((((((((ptFfh@XP_002964409.2_Selaginella_moellendorffii [Archaeplastida]:0.14197825477607795008,(((ptFfh@XP_023898971.1_Quercus_suber [Archaeplastida]:0.02326600966969035247,ptFfh@XP_007204322.1_Prunus_persica [Archaeplastida]:0.02563424245686470385)39:0.01652603294730071656,(ptFfh@XP_016738451.1_Gossypium_hirsutum [Archaeplastida]:0.01407574996329031582,(ptFfh@XP_024442952.1_Populus_trichocarpa [Archaeplastida]:0.04915103984854416602,ptFfh@NP_196014.1_Arabidopsis_thaliana [Archaeplastida]:0.02902885812909651586)59:0.01420962483811747008)41:0.00322736240866677414)100:0.10908959506718947374,ptFfh@ABR16458.1_Picea_sitchensis [Archaeplastida]:0.04972389774594573875)95:0.07648484934775240385)82:0.05738177832053546062,(ptFfh@XP_024383502.1_Physcomitrella_patens [Archaeplastida]:0.00000100000050002909,ptFfh@A9RGM4_Physcomitrella_patens_subsp_patens [Archaeplastida]:0.00000100000050002909)100:0.06396883293581351326)79:0.06727533831369969519,ptFfh@GAQ87100.1_Klebsormidium_nitens [Archaeplastida]:0.16560038096614468350)82:0.06343897421030195161,((ptFfh@A0A061R4M7_Tetraselmis_sp_GSL018 [Archaeplastida]:0.22850865770645603647,ptFfh@GAX85777.1_Chlamydomonas_eustigma [Archaeplastida]:0.18622414872409420727)39:0.03719548375469090146,(ptFfh@I0YJE8_Coccomyxa_subellipsoidea_strain_C-169 [Archaeplastida]:0.12701963113786507442,(ptFfh@A0A2P6TNJ4_Chlorella_sorokiniana [Archaeplastida]:0.07209949817047731080,ptFfh@XP_011401759.1_Auxenochlorella_protothecoides [Archaeplastida]:0.23245175139440549250)84:0.08832678917250892658)30:0.01863988157271342580)41:0.04970516496613769375)71:0.10122920028557480521,ptFfh@MMETSP0308_Transcript_33397_m34971_Gloeochaete_wittrockiana [Archaeplastida]:0.40720803137964428631)51:0.06287650537601374712,((ptFfh@CBN76263.1_Ectocarpus_siliculosus [Stramenopiles]:0.29580897748919171564,(ptFfh@OEU13328.1_Fragilariopsis_cylindrus_CCMP1102 [Stramenopiles]:0.17989779603526373508,(ptFfh@XP_002287312.1_Thalassiosira_pseudonana_CCMP1335 [Stramenopiles]:0.15183071179371879222,(ptFfh@GAX28389.1_Fistulifera_solaris [Stramenopiles]:0.11203559606995742992,ptFfh@XP_002179613.1_Phaeodactylum_tricornutum_CCAP_1055-1 [Stramenopiles]:0.10581710991523843313)93:0.08115966385575341946)86:0.06108825768837369830)99:0.12330656076171002400)60:0.06876624500506436921,(ptFfh@PXF44869.1_Gracilariopsis_chorda [Archaeplastida]:0.11363809051483327039,(ptFfh@GFZU01021206.1_Laurencia_pacifica [Archaeplastida]:0.10800430547295424011,ptFfh@XP_005717012.1_Chondrus_crispus [Archaeplastida]:0.07577694033066392776)57:0.01978849784278602364)97:0.14536376631192993170)98:0.10372383409608075533)35:0.09763175854797150055,((bacFfh@WP_063414566.1_Prochlorococcus_marinus [Cyanobacteria]:0.04228274555847461730,(bacFfh@WP_011618707.1_Synechococcus_sp._CC9311 [Cyanobacteria]:0.02677128805829212185,(bacFfh@WP_087068640.1_Cyanobium_sp_NIES-98

[Cyanobacteria]:0.02558581729242207295,bacFfh@WP_106220726.1_Aphanothece_minutissima [Cyanobacteria]:0.06191596059128601226)71:0.04509169497064501042)33:0.02129472310047410624)59:0.04078358017689645565,(chrFfh@YP_002048787.1_Paulinella_chromatophora [Rhizaria]:0.05785071749220533494,((chrFfh@AXY63015.1_Paulinella_micropora [Rhizaria]:0.00000100000050002909,chrFfh@AQX44624.1_Paulinella_micropora [Rhizaria]:0.01060534255313738509)85:0.02660656585533703600,chrFfh@AUG32274.1_Paulinella_longichromatophora [Rhizaria]:0.03196294034554091751)89:0.04531548127815807764)80:0.05303017104198515935)100:0.19005296274606409135)6:0.03495012366839699708,bacFfh@WP_023172070.1_Gloeobacter_kilaueensis [Cyanobacteria]:0.17115778053351254906)1:0.01623244571181597581)42:0.08187956683800667879)97:0.18392844686971521195)35:0.05095293616508594220)19:0.04931691676739148600,(bacFfh@WP_006928829.1_Caldithrix_abyssi [Calditrichaeota]:0.20380482556394052818,bacFfh@OPX24629.1_Candidatus_Latescibacteria_bacterium_4484_107 [Bacteroidetes]:0.24024889072434493786)58:0.10305086562681951834)7:0.04170359233719587305,((bacFfh@WP_006980240.1_Chthoniobacter_flavus [Verrucomicrobia]:0.19893532678681824510,((bacFfh@CDD93459.1_Akkermansia_sp_CAG_344 [Verrucomicrobia]:0.01800080466698905993,bacFfh@WP_102742496.1_Akkermansia_muciniphila [Verrucomicrobia]:0.00473765237820629199)100:0.14779299719953173375,(bacFfh@WP_035603039.1_Haloferula_sp_BvORR071 [Verrucomicrobia]:0.19366547651257917662,bacFfh@WP_105044656.1_Rubritalea_profundi [Verrucomicrobia]:0.14360566236056546141)60:0.04088407404732458811)84:0.12526506770335121832)100:0.22919762071895383171,bacFfh@WP_049675438.1_Desulfocarbo_indianensis [Deltaproteobacteria]:0.24114632519749165662)18:0.07650363893236396262)3:0.05529917210212176304,((((((((mtFfh@NA_Pharyngomonas_kirbyi [Heterolobosea]:0.33882451633207893993,(mtFfh@NA_Percolomonas_ex_Nitzchia_Cheng_2013 [Heterolobosea]:0.37750466052254977312,mtFfh@NA_Percolomonas_cosmopolitus_strain_WS [Heterolobosea]:0.23624223078376205276)100:0.45192183566788896032)47:0.09093316209029012054,((mtFfh@NA_Neovahlkampfia_damariscottae [Heterolobosea]:0.33427431228088594350,(mtFfh@NA_Naegleri_gruberi [Heterolobosea]:0.09329493374496483638,(mtFfh@NA_Naegleria_lovaniensis [Heterolobosea]:0.01308622787623464970,mtFfh@NA_Naegleria_fowleri [Heterolobosea]:0.01314685964526706406)75:0.03438281922618015296)100:0.28315785643757523937)76:0.08437248746303629976,mtFfh@NA_Heterolobosea_BB2 [Heterolobosea]:0.20310998485122777613)59:0.06222604268425086654)64:0.11800624196776453301,((mtFfh@NA_Percolomonas_cosmopolitus_strain_AE [Heterolobosea]:0.71862620049222547536,(mtFfh@NA_Goniomonas_avonlea [Cryptophyta]:0.22897501026689434656,mtFfh@NA_Goniomonas_pacifica [Cryptophyta]:0.56161287352701971809)89:0.21379725244210803781)51:0.08272283292497691065,((mtFfh@NA_Ancoracysta_twista [Ancoracysta]:0.20356767705493256093,mtFfh@NA_Ancoracysta-related_Colp-4b [Ancoracysta]:0.26542691864768341858)100:0.25361299846408968950,(mtFfh@NA_Hemimastix_kukwesjijk [Hemimastigophora]:0.24636660707286728300,mtFfh@NA_Spironema_sp [Hemimastigophora]:0.46246460097021213631)100:0.40964069623539578480)42:0.06660984421685213486)26:0.07152843767586909707)85:0.13912344992274910949,((bacFfh@PDH20339.1_Pelagibacterales_bacterium_MED-G40 [Alphaproteobacteria]:0.40010365461075420024,(bacFfh@PLX30515.1_Alphaproteobacteria_bacterium [Alphaproteobacteria]:0.23722924860382310630,bacFfh@OUW71296.1_Rickettsiales_bacterium_TMED211 [Alphaproteobacteria]:0.35999727707903628193)15:0.03430646419883354864)22:0.05792847717337933078,bacFfh@PPR79452.1_Alphaproteobacteria_bacterium_MarineAlpha2_Bin1 [Alphaproteobacteria]:0.26397340453053469433)7:0.06303067286535189717)2:0.05258321663032208132,((bacFfh@WP_027134478.1_Geminicoccus_roseus [Alphaproteobacteria]:0.20763016763948546894,bacFfh@WP_088559973.1_Arboriscoccus_pini [Alphaproteobacteria]:0.16381255577807318780)68:0.07467975413132235674,bacFfh@PZP86081.1_Azospirillum_brasilense [Alphaproteobacteria]:0.23299344006014421904)13:0.04437645966586772905)0:0.01866414884066464747,(((((bacFfh@WP_043360684.1_Belnapia_sp_F-4-1 [Alphaproteobacteria]:0.12945114253150932782,bacFfh@WP_034336672.1_Commensalibacter_sp_MX01 [Alphaproteobacteria]:0.23956472131487463462)70:0.08759093078236934726,(bacFfh@WP_014746762.1_Tistrella_mobilis [Alphaproteobacteria]:0.17093707691686776950,bacFfh@WP_038035438.1_Thermopetrobacter_sp_TC1 [Alphaproteobacteria]:0.19843562296131539435)3:0.00846111064611472681)1:0.02262908259898584185,((((((bacFfh@WP_012973141.1_Azospirillum_lipoferum [Alphaproteobacteria]:0.05600244748095792313,bacFfh@WP_094454746.1_Niveispirillum_lacus [Alphaproteobacteria]:0.17056078523072903952)34:0.02568255773219487240,bacFfh@WP_028466142.1_Nisaea_denitrificans [Alphaproteobacteria]:0.15085042521179942154)21:0.05492576267419825575,((bacFfh@OUX71121.1_Rhodospirillaceae_bacterium_TMED140 [Alphaproteobacteria]:0.18835850639560014486,((bacFfh@OIN86659.1_Alphaproteobacteria_bacterium_CG1_02_46_17 [Alphaproteobacteria]:0.14119859327792488868,bacFfh@PZQ45682.1_Micavibrio_aeruginosavorus [Alphaproteobacteria]:0.10819713782305898087)86:0.06050389824376407877,(bacFfh@WP_015467793.1_Micavibrio_aeruginosavorus [Alphaproteobacteria]:0.14072386056377020336,bacFfh@PCJ00252.1_Alphaproteobacteria_bacterium [Alphaproteobacteria]:0.20629560092839310803)50:0.02956315159386975130)84:0.04864706086011637981)26:0.03562073886011758034,bacFfh@WP_092823393.1_Rhodospirillales_bacterium_URHD0017 [Alphaproteobacteria]:0.21127776938258846506)8:0.01322219039012935936)1:0.02454509969283892590,((bacFfh@OUT52366.1_Rhodospirillaceae_bacterium_TMED8 [Alphaproteobacteria]:0.22169670966752269559,bacFfh@WP_069956818.1_Magnetovibrio_blakemorei [Alphaproteobacteria]:0.13379703813996254858)51:0.08158886177688741415,(bacFfh@PHY00942.1_Rhodospirillaceae_bacterium [Alphaproteobacteria]:0.26757674163255540378,((((bacFfh@WP_092613925.1_Roseospirillum_parvum [Alphaproteobacteria]:0.21054707059766786248,bacFfh@WP_019645789.1_Novispirillum_itersonii [Alphaproteobacteria]:0.08381708163014395252)18:0.03296168878145595837,(((bacFfh@WP_028877797.1_Terasakiella_pusilla [Alphaproteobacteria]:0.14963702601507100631,bacFfh@WP_073953247.1_Thalassospira_sp_TSL5-1 [Alphaproteobacteria]:0.15970871946050371748)33:0.04973928942204348252,((bacFfh@CDB39986.1_Azospirillum_sp_CAG_260 [Alphaproteobacteria]:0.03225035730291445080,bacFfh@CDB53938.1_Azospirillum_sp_CAG_239 [Alphaproteobacteria]:0.04293926407234533588)100:0.21643442832165829537,bacFfh@OFX07559.1_Alphaproteobacteria_bacterium_RIFOXYD12_FULL_60_8 [Alphaproteobacteria]:0.12601218015500439962)64:0.06379987869433020875)3:0.01791003638360557509,(bacFfh@WP_068493492.1_Magnetospirillum_marisnigri [Alphaproteobacteria]:0.03534659450678075132,(bacFfh@WP_002727862.1_Phaeospirillum_molischianum [Alphaproteobacteria]:0.04738041580354581533,(bacFfh@WP_068497751.1_Magnetospirillum_moscoviense [Alphaproteobacteria]:0.01287266082249012566,(bacFfh@OJX70273.1_Magnetospirillum_sp_64-120 [Alphaproteobacteria]:0.03856675391267830322,bacFfh@WP_024081694.1_Magnetospirillum_gryphiswaldense [Alphaproteobacteria]:0.02073897701568194751)87:0.02829720870309408026)100:0.08100732772571886398)50:0.02044224631060035172)98:0.10981296665390559431)1:0.02636691238035656845)6:0.02604576866441076638,bacFfh@WP_041795188.1_Pararhodospirillum_photometricum [Alphaproteobacteria]:0.16327272791356070569)7:0.03104378470036792126,bacFfh@CCZ21287.1_Acetobacter_sp_CAG_977 [Alphaproteobacteria]:0.16312066725564494662)7:0.02601403467200257003)13:0.01648149326748685542)3:0.04147314186070859970)0:0.01745571701073935292,(((((bacFfh@WP_109920731.1_Zavarzinia_compransoris [Alphaproteobacteria]:0.20618075570383623263,((((bacFfh@RCL83452.1_PS1_clade_bacterium [Alphaproteobacteria]:0.24540465931108629283,bacFfh@OQW59094.1_Proteobacteria_bacterium_HN_bin10 [Proterobacteria]:0.27592554892302934011)45:0.06630237368934069342,bacFfh@OJT95039.1_Alphaproteobacteria_bacterium_65-7 [Alphaproteobacteria]:0.18518529326771715482)19:0.04613476442093481650,(bacFfh@RCL81099.1_SAR116_cluster_bacterium [Alphaproteobacteria]:0.27175531608272535111,bacFfh@WP_116392064.1_Parvularcula_sp_SM1705 [Alphaproteobacteria]:0.23042627928358375144)15:0.05312087987461389105)4:0.04812009819031005942,(bacFfh@PKQ09217.1_Alphaproteobacteria_bacterium_HGW-Alphaproteobacteria-12 [Alphaproteobacteria]:0.15147801352477696657,((bacFfh@OUU83527.1_Hyphomicrobiaceae_bacterium_TMED74 [Alphaproteobacteria]:0.12380719729924347106,bacFfh@WP_099557780.1_Hartmannibacter_diazotrophicus [Alphaproteobacteria]:0.15067025431615249653)62:0.06196219227984622185,(bacFfh@WP_013420482.1_Rhodomicrobium_vannielii [Alphaproteobacteria]:0.11395953631121094607,((bacFfh@WP_018634118.1_Neomegalonema_perideroedes [Alphaproteobacteria]:0.20934698229818696213,(bacFfh@WP_111197444.1_Rhizobiales_bacterium_KCTC_52945 [Alphaproteobacteria]:0.10737057046345858946,(bacFfh@WP_085770433.1_Methylocystis_bryophila [Alphaproteobacteria]:0.27494682310862728070,bacFfh@GBE42717.1_Bacterium_BMS3Bbin10 [unassigned]:0.14546953387070804542)26:0.03814801140909301891)13:0.02656649458925550850)2:0.00591942215457636096,(bacFfh@PCI85686.1_Rhizobiales_bacterium [Alphaproteobacteria]:0.00000100000050002909,bacFfh@PCJ00993.1_OCS116_cluster_bacterium [Alphaproteobacteria]:0.00367421550479839340)100:0.18738268359328619228)2:0.02637303482066291616)2:0.02999884575528860564)6:0.02898082320202577736)6:0.03169019732031102404)2:0.02266763153896266328)0:0.01377650528058287256,((bacFfh@PCI43326.1_Alphaproteobacteria_bacterium [Alphaproteobacteria]:0.18795533679639306324,(bacFfh@WP_072596811.1_Sphingomonas_sp_JJ-A5 [Alphaproteobacteria]:0.09975470800596648868,(bacFfh@WP_011240910.1_Zymomonas_mobilis [Alphaproteobacteria]:0.17682782594339313542,((bacFfh@WP_014076705.1_Sphingobium_sp_SYK-6 [Alphaproteobacteria]:0.08782474140724426392,(bacFfh@WP_010335520.1_Sphingobium_yanoikuyae [Alphaproteobacteria]:0.02789049269700132408,bacFfh@SCW52489.1_Sphingobium_faniae [Alphaproteobacteria]:0.04005293189996907144)54:0.01574081269638625408)50:0.02103334522096160855,(bacFfh@WP_089215287.1_Sphingopyxis_indica [Alphaproteobacteria]:0.10549883148259642240,(bacFfh@WP_116091360.1_Sphingomonas_crusticola [Alphaproteobacteria]:0.09407050449864755304,bacFfh@WP_022691338.1_Sphingomonas-like_bacterium_B12 [Alphaproteobacteria]:0.09775344932621954364)37:0.02766105796554951812)25:0.02603626625454374260)38:0.03590522408378814950)74:0.05130755943124549329)97:0.10231180447779297804)13:0.04354069051688550657,(bacFfh@WP_025896931.1_Sneathiella_glossodoripedis [Alphaproteobacteria]:0.03461835074760195169,bacFfh@OUR76855.1_Alphaproteobacteria_bacterium_46_93_T64 [Alphaproteobacteria]:0.05740017733294774244)99:0.12051983543604949389)4:0.02907829105279070153)2:0.01752319736837087971,bacFfh@WP_115937331.1_Aestuariispira_insulae [Alphaproteobacteria]:0.14985001225699040583)1:0.00507798970510726488,bacFfh@PPR12333.1_Alphaproteobacteria_bacterium_MarineAlpha11_Bin1 [Alphaproteobacteria]:0.21389539283960989913)1:0.01134686876777322301,((bacFfh@WP_027287498.1_Rhodovibrio_salinarum [Alphaproteobacteria]:0.19639448825838753954,bacFfh@WP_046506348.1_Kiloniella_litopenaei [Alphaproteobacteria]:0.10011435580491591379)25:0.03872914830080945864,bacFfh@WP_119283627.1_Rhodospirillaceae_bacterium_SYSU_D60006 [Alphaproteobacteria]:0.12387440383336939531)23:0.05331249504404061285)0:0.00970792534454255861)0:0.02151117734437034759,bacFfh@PCJ58386.1_Rhodospirillaceae_bacterium [Alphaproteobacteria]:0.25875151531489870571)0:0.02844069246313604693)0:0.03317969706804795721,(bacFfh@OHC73580.1_Rhodospirillales_bacterium_RIFCSPLOWO2_02_FULL_58_16 [Alphaproteobacteria]:0.17239009643030109298,bacFfh@PPR36214.1_Alphaproteobacteria_bacterium_MarineAlpha9_Bin6 [Alphaproteobacteria]:0.23104562373614867532)35:0.06176076213616577687)0:0.02841357071791045588,((bacFfh@PIR38902.1_Alphaproteobacteria_bacterium_CG11_big_fil_rev_8_21_14_0_20_39_49 [Alphaproteobacteria]:0.16031617429735009472,bacFfh@KKB96089.1_Arcanobacter_lacustris [Alphaproteobacteria]:0.22754111300882595703)28:0.06621152165090854469,((bacFfh@WP_032113236.1_Candidatus_Paracaedibacter_symbiosus [Alphaproteobacteria]:0.18717723187869303447,((bacFfh@WP_065432712.1_Ehrlichia_ruminantium [Alphaproteobacteria]:0.19975542559727529901,bacFfh@WP_025264366.1_Wolbachia_endosymbiont_of_Onchocerca_volvulus [Alphaproteobacteria]:0.12939759442003095913)100:0.21983436054855617914,(bacFfh@OUX67412.1_Rhizobiales_bacterium_TMED227 [Alphaproteobacteria]:0.04303266798651422115,bacFfh@OUT75042.1_Rhizobiales_bacterium_TMED25 [Alphaproteobacteria]:0.11959903930620272017)100:0.19348124048209108805)24:0.06991002894726491834)4:0.03382365993994516234,(bacFfh@OYZ36327.1_Alphaproteobacteria_bacterium_16-39-46 [Alphaproteobacteria]:0.22158019036624360121,((bacFfh@OJV16027.1_Alphaproteobacteria_bacterium_33-17 [Alphaproteobacteria]:0.36997660750391536677,bacFfh@WP_085783826.1_Candidatus_Nucleicultrix_amoebiphila [Alphaproteobacteria]:0.11386254160558421933)70:0.06514301022720975221,bacFfh@OJX13986.1_Caedibacter_sp_37-49 [Alphaproteobacteria]:0.17780990275644995013)51:0.04171222403921961974)33:0.03648527354439141251)5:0.03574078992331825716)1:0.03055269355284195854)0:0.02625009864615362662)9:0.04368451164093042904,(bacFfh@PPR14985.1_Alphaproteobacteria_bacterium_MarineAlpha9_Bin3 [Alphaproteobacteria]:0.33290916104182932678,bacFfh@PPR20700.1_Alphaproteobacteria_bacterium_MarineAlpha10_Bin2 [Alphaproteobacteria]:0.10371282553306103436)45:0.06536166520614600239)92:0.10987710408698658782,(bacFfh@WP_025769383.1_Thioalkalivibrio_sp_HK1 [Gammaproteobacteria]:0.20666936703624977367,(bacFfh@WP_091713482.1_Methylophaga_sulfidovorans [Gammaproteobacteria]:0.12619914584678673664,((bacFfh@WP_024496304.1_Candidatus_Schmidhempelia_bombi [Gammaproteobacteria]:0.12014805320526407428,(bacFfh@WP_033187470.1_Pseudoalteromonas_sp_PLSV [Gammaproteobacteria]:0.10476975303411852070,(bacFfh@WP_078744295.1_Oceanospirillum_multiglobuliferum [Gammaproteobacteria]:0.07457057958885469906,bacFfh@WP_039914025.1_Cellvibrio_mixtus [Gammaproteobacteria]:0.12859626359216450031)78:0.03291750891200839746)89:0.06639172456088884844)37:0.01904033303888389914,(bacFfh@WP_114137085.1_Klebsiella_pneumoniae [Gammaproteobacteria]:0.04811421428760594837,bacFfh@RJL31521.1_Pectobacterium_polaris [Gammaproteobacteria]:0.05358382888384116000)98:0.05625975611169781176)54:0.04924788814958993427)52:0.03980173482860725753)100:0.19079031339396176903)84:0.11853225823527083205)0:0.01654441232374660037)2:0.01741969997855478264)93:0.26903100754355857571)99:0.38573626489612355961)91:0.24593197202852856709,((bacFtsY@KUK71287.1_Anaerolineae_bacterium_49_20 [Chloroflexi]:0.47846043026404105891,bacFtsY@OQY47814.1_Anaerolineaceae_bacterium_4572_78 [Chloroflexi]:0.33319450950979223602)27:0.11753777330038658877,bacFtsY@WP_038038136.1_Thermorudis_peleae [Chloroflexi]:0.33243720803673698638)8:0.03451402562537073115)1:0.05656477953362820665,bacFtsY@AEG15826.1_Desulfofundulus_kuznetsovii_DSM_6115 [Firmicutes]:0.18786433243235900470)0:0.03933217561776082560)0:0.01757626841727279524,(bacFtsY@EEG77220.1_Dethiobacter_alkaliphilus_AHT_1 [Firmicutes]:0.24332689430162129929,(((bacFtsY@WP_054252106.1_Neofamilia_massiliensis [Firmicutes]:0.36776242536611541967,bacFtsY@WP_072972532.1_Tissierella_praeacuta [Firmicutes]:0.20487923089500489904)66:0.11934368339404301251,((bacFtsY@WP_026974763.1_Alicyclobacillus_contaminans [Firmicutes]:0.32831654353237077482,(bacFtsY@PTQ57904.1_Candidatus_Carbobacillus_altaicus [Firmicutes]:0.25388880577617273238,(((bacFtsY@WP_009554695.1_Lactobacillus_saerimneri [Firmicutes]:0.09126004203642500145,(bacFtsY@WP_056961480.1_Lactobacillus_florum [Firmicutes]:0.13640055879871265665,bacFtsY@WP_103423367.1_Lactobacillus_sanfranciscensis [Firmicutes]:0.08913826055600369835)100:0.19983532130173226049)87:0.13080304301491207930,(bacFtsY@WP_107510123.1_Staphylococcus_fleurettii [Firmicutes]:0.08921227221646828298,bacFtsY@AVK83142.1_Lysinibacillus_sp_B2A1 [Firmicutes]:0.10527844871060137999)67:0.06183490551169087968)52:0.08880874406016639466,(bacFtsY@WP_069327418.1_Paenibacillus_sp_TI45-13ar [Firmicutes]:0.10532981789216089297,(bacFtsY@WP_028778316.1_Shimazuella_kribbensis [Firmicutes]:0.08852800472870961390,bacFtsY@WP_091834879.1_Marininema_halotolerans [Firmicutes]:0.15997154470596100517)49:0.058389053208776

5078)28:0.05144527855060283122)17:0.03008643339253752602)46:0.05614584295136421099)40:0.04972865722813743505,bacFtsY@WP_073092242.1_Thermosyntropha_lipolytica [Firmicutes]:0.31903808703297337201)18:0.02375734282776547496)7:0.01184317749455244921,bacFtsY@CDA51269.1_Clostridium_sp_CAG-138 [Firmicutes]:0.34036260110128740974)4:0.02850055824181088951)7:0.06800632304249062954)0:0.04447970036699913643)0:0.01372131600196954335)0:0.04648982325008212818)0:0.01862178119181839842)0:0.04654184816193523283)0:0.02961699306670354317,(bacFtsY@WP_083764053.1_Syntrophobacter_fumaroxidans [Deltaproteobacteria]:0.28458316040389125545,(bacFtsY@PIE60181.1_Desulfobulbus_propionicus [Deltaproteobacteria]:0.26520599187063864655,(bacFtsY@ABC77884.1_Syntrophus_aciditrophicus_SB [Deltaproteobacteria]:0.16648924783266449978,bacFtsY@PIP06279.1_Syntrophobacteraceae_bacterium_CG23_combo_of_CG06-09_8_20_14_all_50_8 [Deltaproteobacteria]:0.13045829300652997396)95:0.13723863866720753535)19:0.05507318345495423179)8:0.07056649280446034012)0:0.06542318014875926624)4:0.01839156724971097862)25:0.06814976838599268172,bacFtsY@WP_095208401.1_Luteimonas_sp_JM171 [Gammaproteobacteria]:0.24331214188095301454)19:0.05269498030597871696)14:0.07809783809216648554,(bacFtsY@WP_091826228.1_Marinobacterium_georgiense [Gammaproteobacteria]:0.07062140132469293952,(bacFtsY@ARM82543.1_Marinobacter_salarius [Gammaproteobacteria]:0.12912082103516983600,((bacFtsY@PPI88525.1_Pantoea_sp_SoEO [Gammaproteobacteria]:0.41222171778477995074,((bacFtsY@WP_091986549.1_Pseudoalteromonas_denitrificans [Gammaproteobacteria]:0.12606462253379591143,bacFtsY@WP_094041167.1_Zobellella_denitrificans [Gammaproteobacteria]:0.05818797033052287154)67:0.02347378511152585154,bacFtsY@WP_113743312.1_Anaerobiospirillum_thomasii [Gammaproteobacteria]:0.29048817234278057065)57:0.04601198573357339477)68:0.08827703973200025367,bacFtsY@WP_097790462.1_Halomonas_beimenensis [Verrucomicrobia]:0.13501697965166001292)17:0.04593815398705444752)18:0.04203870294276031150)47:0.09593968154830914696)26:0.08730576119708441385)100:0.37142896432942745788)42:0.08117686617735560273,(bacFtsY@WP_015110998.1_Nostoc_sp [Cyanobacteria]:0.10092725407747389632,bacFtsY@WP_111893930.1_Arthrospira_sp_O9_13F [Cyanobacteria]:0.05024691122047121594)97:0.11570128898074151513,bacFtsY@WP_081705397.1_Gloeobacter_kilaueensis [Cyanobacteria]:0.24785925847716364090);

**Phylogenetic tree from Fig. 3B (RAxML, LG4X model, 500 rapid bootstraps, 295 OTUs)**

((bacFfh@WP_091713482.1_Methylophaga_sulfidovorans [Gammaproteobacteria]:0.17922513106593246368,(((bacFfh@WP_078744295.1_Oceanospirillum_multiglobuliferum [Gammaproteobacteria]:0.12892649098739630831,bacFfh@WP_039914025.1_Cellvibrio_mixtus [Gammaproteobacteria]:0.16859631746489905924)95:0.05330008093810168629,bacFfh@WP_033187470.1_Pseudoalteromonas_sp_PLSV [Gammaproteobacteria]:0.13250023277380218456)98:0.08315313860041032012,((bacFfh@WP_114137085.1_Klebsiella_pneumoniae [Gammaproteobacteria]:0.04555244369838055973,bacFfh@RJL31521.1_Pectobacterium_polaris [Gammaproteobacteria]:0.05850078013118158171)96:0.05105733175956912034,bacFfh@WP_024496304.1_Candidatus_Schmidhempelia_bombi [Gammaproteobacteria]:0.13717649904418863227)86:0.05348727816981926203)91:0.08000924747682494620)76:0.05403695035438659672,(((((((bacFfh@PCJ61345.1_Planctomycetes_bacterium [Planctomycetes]:0.47052999025845371195,bacFfh@PIQ24328.1_Candidatus_Blackallbacteria [unassigned]:0.26007972562565290220)54:0.07665260546776622719,(((((chrFfh@YP_002048787.1_Paulinella_chromatophora [Rhizaria]:0.11270534784572269760,(chrFfh@AUG32274.1_Paulinella_longichromatophora [Rhizaria]:0.04793587744131515976,(chrFfh@AXY63015.1_Paulinella_micropora [Rhizaria]:0.00355564390484014795,chrFfh@AQX44624.1_Paulinella_micropora [Rhizaria]:0.01280514128179189789)99:0.03539095147410035308)99:0.04879821170990305407)100:0.09724609843209201798,((bacFfh@WP_063414566.1_Prochlorococcus_marinus [Cyanobacteria]:0.06575070570724747543,bacFfh@WP_011618707.1_Synechococcus_sp._CC9311 [Cyanobacteria]:0.04153824967708924509)45:0.01789933540335870640,(bacFfh@WP_087068640.1_Cyanobium_sp_NIES-981 [Cyanobacteria]:0.05523090698405996868,bacFfh@WP_106220726.1_Aphanothece_minutissima [Cyanobacteria]:0.06564455802918797711)74:0.03298344797162849895)33:0.02782869649074910107)100:0.23138582115934552830,(bacFfh@WP_011429947.1_Synechococcus_sp [Cyanobacteria]:0.19594517150593301902,(bacFfh@WP_023172070.1_Gloeobacter_kilaueensis [Cyanobacteria]:0.21146181043833189861,((bacFfh@RMH67925.1_Cyanobacteria_bacterium_J003 [Cyanobacteria]:0.07528075722717009199,bacFfh@WP_015124913.1_Synechococcus_sp_PCC_6312 [Cyanobacteria]:0.09332077756735934759)92:0.05031526303591263294,((bacFfh@0A2P8WK82_Filamentous_cyanobacterium_CCP5 [Cyanobacteria]:0.12799927431294630398,((bacFfh@WP_009785362.1_Lyngbya_sp_PCC_8106 [Cyanobacteria]:0.11319627479886967825,bacFfh@WP_011612100.1_Trichodesmium_erythraeum [Cyanobacteria]:0.10159766575488606011)46:0.03880204265322507312,bacFfh@WP_081914739.1_Leptolyngbya_sp [Cyanobacteria]:0.08574642818186355153)23:0.02087896221470693717)14:0.01435167021716924479,((bacFfh@KFF41240.1_Candidatus_Atelocyanobacterium_thalassa_isolate_SIO64986 [Cyanobacteria]:0.21448364781651960143,bacFfh@A8YEA3_Microcystis_aeruginosa_PCC_7806 [Cyanobacteria]:0.09302022881184365821)94:0.06236338668340365987,bacFfh@PSP17129.1_Cyanobacteria_bacterium_QS_8_64_29 [Cyanobacteria]:0.23663715616752312165)19:0.02854154423581927585)37:0.02983953494908855517)94:0.07939025833564002099)36:0.03408469701270914337)35:0.02492479078843496884)78:0.07431681380247964763,((((ptFfh@I0YJE8_Coccomyxa_subellipsoidea_strain_C-169 [Archaeplastida]:0.16385434441534948991,(ptFfh@XP_011401759.1_Auxenochlorella_protothecoides [Archaeplastida]:0.30325974018306500746,ptFfh@A0A2P6TNJ4_Chlorella_sorokiniana [Archaeplastida]:0.13956736530313607059)75:0.08812119186613527611)63:0.04823456331005745307,(ptFfh@GAX85777.1_Chlamydomonas_eustigma [Archaeplastida]:0.30678614480393739949,ptFfh@A0A061R4M7_Tetraselmis_sp_GSL018 [Archaeplastida]:0.27872942062325578583)51:0.04645655430748124592)95:0.07423818279189413338,(((ptFfh@XP_024383502.1_Physcomitrella_patens [Archaeplastida]:0.00000100000050002909,ptFfh@A9RGM4_Physcomitrella_patens_subsp_patens [Archaeplastida]:0.00000100000050002909)100:0.07939503849837227512,((ptFfh@ABR16458.1_Picea_sitchensis [Archaeplastida]:0.11205949301019860198,((ptFfh@XP_021316972.1_Sorghum_bicolor [Archaeplastida]:0.03084370977618336104,ptFfh@XP_003576310.1_Brachypodium_distachyon [Archaeplastida]:0.04588693485873251987)100:0.10895905367368166616,(ptFfh@OAY71139.1_Ananas_comosus [Archaeplastida]:0.03758278693949963778,(((ptFfh@XP_016738451.1_Gossypium_hirsutum [Archaeplastida]:0.03563522483374905597,(ptFfh@XP_024442952.1_Populus_trichocarpa [Archaeplastida]:0.03500735273849546469,(ptFfh@NP_196014.1_Arabidopsis_thaliana [Archaeplastida]:0.04596073912068596862,(ptFfh@XP_021977940.1_Helianthus_annuus [Archaeplastida]:0.03032431343378165153,(ptFfh@XP_016448785.1_Nicotiana_tabacum [Archaeplastida]:0.03143713491166948149,ptFfh@XP_006341101.1_Solanum_tuberosum [Archaeplastida]:0.01873540187953964473)66:0.01457032754799508951)98:0.03057936486900334969)36:0.01474688363072188153)21:0.01163323697053106169)5:0.00470855027090263598,(((ptFfh@XP_008391835.1_Malus_domestica [Archaeplastida]:0.02488960182657326548,ptFfh@XP_007204322.1_Prunus_persica [Archaeplastida]:0.01953418961490453742)86:0.02339493792271949216,ptFfh@XP_023898971.1_Quercus_suber [Archaeplastida]:0.02873539073560034060)23:0.01030348760157002649,(ptFfh@XP_024038087.1_Citrus_clementina [Archaeplastida]:0.03828291278093431949,(ptFfh@PON78013.1_Parasponia_andersonii [Archaeplastida]:0.02014239851847496668,((ptFfh@ABN06080.1_Medicago_truncatula [Archaeplastida]:0.05816270056741890127,(ptFfh@XP_017418457.1_Vigna_angularis [Archaeplastida]:0.01747603553003690066,ptFfh@XP_003521470.1_Glycine_max [Archaeplastida]:0.00815210366162099839)95:0.01151615008968668273)34:0.00977701182695485393,(ptFfh@CCH47177.1_Lupinus_angustifolius [Archaeplastida]:0.05726906757382471680,ptFfh@XP_020980415.1_Arachis_duranensis [Archaeplastida]:0.01410127281656808548)55:0.01460173716620487110)46:0.01872071772841675530)7:0.00558696228296207045)10:0.00680026190408112435)2:0.00681864101762648389)25:0.00834963978263714851,(ptFfh@XP_002530328.1_Ricinus_communis [Archaeplastida]:0.01751063844649174833,ptFfh@XP_021621164.1_Manihot_esculenta [Archaeplastida]:0.02393501768605797719)50:0.00487118680347522082)77:0.04523165010215403348)49:0.02239412801728781627)100:0.08304946265069000633)99:0.09020309128855230618,ptFfh@XP_002964409.2_Selaginella_moellendorffii [Archaeplastida]:0.20081445549450602228)70:0.05037919507626879845)99:0.14181899802427916368,ptFfh@GAQ87100.1_Klebsormidium_nitens [Archaeplastida]:0.23481351402065619993)98:0.07350779411390867546)100:0.15385033119697863468,((ptFfh@PXF44869.1_Gracilariopsis_chorda [Archaeplastida]:0.12769513410064772807,(ptFfh@XP_005717012.1_Chondrus_crispus [Archaeplastida]:0.11656039890759813116,ptFfh@GFZU01021206.1_Laurencia_pacifica [Archaeplastida]:0.18498952508046986876)46:0.03035028345107371531)100:0.25479370346395424640,((ptFfh@OEU13328.1_Fragilariopsis_cylindrus_CCMP1102 [Stramenopiles]:0.19855332416564139275,((ptFfh@GAX28389.1_Fistulifera_solaris [Stramenopiles]:0.09921325115765142200,ptFfh@XP_002179613.1_Phaeodactylum_tricornutum_CCAP_1055-1 [Stramenopiles]:0.13453634335359704322)100:0.10193329514607887831,ptFfh@XP_002287312.1_Thalassiosira_pseudonana_CCMP1335 [Stramenopiles]:0.15591381715435015387)91:0.08129093842076369480)100:0.16741085820794060490,ptFfh@CBN76263.1_Ectocarpus_siliculosus [Stramenopiles]:0.41479299049795109733)75:0.08681787178125874305)99:0.11318866551372917806)72:0.05124235108994834587)100:0.19648836447786996451,bacFfh@OJX56912.1_Candidatus_Kapabacteria_thiocyanatum [Bacteroidetes]:0.36256941367715123103)34:0.05134331850999175606)34:0.05390356188075951654,((((archSRP54@PIV68086.1_Euryarchaeota_archaeon_CG01_land_8_20_14_3_00_38_12 [Euryarchaeota]:0.27601936206772748061,archSRP54@RLF74017.1_Thermoplasmata_archaeon [Euryarchaeota]:0.17145078441364899891)100:0.15269123875054546602,(((archSRP54@WP_079234317.1_Halolamina_sp_CBA1230 [Euryarchaeota]:0.09782956816929117005,(archSRP54@Q977V2_Haloferax_volcanii [Euryarchaeota]:0.07837183738349322304,((archSRP54@WP_066299325.1_Haloterrigena_mahii [Euryarchaeota]:0.00401039090220253486,((archSRP54@WP_006826848.1_Natrialba_taiwanensis [Euryarchaeota]:0.02916196575984508071,archSRP54@WP_049990021.1_Halopiger_salifodinae [Euryarchaeota]:0.01564088636411202493)58:0.00996583308625343355,archSRP54@WP_005578715.1_Natronobacterium_gregoryi [Euryarchaeota]:0.04638619779677047306)77:0.01879717824421110045)100:0.11379621333443080966,archSRP54@WP_092813433.1_Halopenitus_malekzadehii [Euryarchaeota]:0.07954313589675252039)36:0.02826596768955424999)22:0.02025548889826458282)100:0.35980231036812793111,archSRP54@WP_011449961.1_Methanospirillum_hungatei [Euryarchaeota]:0.34355945683162869564)81:0.08625658084109220913,((archSRP54@KXS43658.1_Methanolobus_sp_T82-4 [Euryarchaeota]:0.13208433929143986729,archSRP54@WP_095645771.1_Methanosarcina_spelaei [Euryarchaeota]:0.12922814540254096149)98:0.08048423347211895729,archSRP54@WP_014405995.1_Methanocella_conradii [Euryarchaeota]:0.20121095585641740611)39:0.04235691593017004708)98:0.13774373473035861348)99:0.13060935637848886115,(((eukSRP54@GECH01013817.1_Pharyngomonas_kirbyi [Heterolobosea]:0.32513443472552455704,(((eukSRP54@XP_024367976.1_Physcomitrella_patens [Archaeplastida]:0.06446491000621192013,(eukSRP54@PON48078.1_Parasponia_andersonii [Archaeplastida]:0.00902851548980189086,((eukSRP54@XP_006465307.1_Citrus_sinensis [Archaeplastida]:0.00452529241646812148,eukSRP54@XP_006427317.1_Citrus_clementina [Archaeplastida]:0.00000100000050002909)96:0.01780996543064825369,((eukSRP54@XP_008454178.1_Cucumis_melo [Archaeplastida]:0.02516633059463302055,(eukSRP54@XP_022724150.1_Durio_zibethinus [Archaeplastida]:0.00451138675269901514,eukSRP54@XP_012455795.1_Gossypium_raimondii [Archaeplastida]:0.00225117584058394469)71:0.00000100000050002909)39:0.00494234879935627914,eukSRP54@XP_021636295.1_Hevea_brasiliensis [Archaeplastida]:0.00638522411920048553)43:0.00702849486000275536)54:0.01250136272381819832)100:0.07098119889445633768)100:0.18566413348342999945,(((eukSRP54@XP_004367866.1_Acanthamoeba_castellanii_strain_Neff [Amoebozoa]:0.20279718200608268242,((eukSRP54@EPZ32142.1_Rozella_allomycis_CSF55 [Opisthokonta]:0.25556350525625098902,((eukSRP54@RDX56745.1_Polyporus_brumalis [Opisthokonta]:0.01940354503155234181,(((eukSRP54@KXN86887.1_Leucoagaricus_sp_SymC_cos [Opisthokonta]:0.03660793204318028848,(eukSRP54@PBK96926.1_Armillaria_gallica [Opisthokonta]:0.02236546403623084628,(eukSRP54@XP_001833227.1_Coprinopsis_cinerea_okayama [Opisthokonta]:0.06255436973129997746,eukSRP54@KDQ33155.1_Pleurotus_ostreatus_PC15 [Opisthokonta]:0.03041931190985273578)20:0.00862756418531321692)3:0.00319535941809201678)7:0.00302291568804484261,eukSRP54@KIK08302.1_Laccaria_amethystina_LaAM-08-1 [Opisthokonta]:0.02402675176615491973)16:0.01125328797832216877,eukSRP54@KNZ71807.1_Termitomyces_sp_J132 [Opisthokonta]:0.02050993738839832284)70:0.04930327473089960583)100:0.37641792731066353594,eukSRP54@XP_016608410.1_Spizellomyces_punctatus_DAOM_BR117 [Opisthokonta]:0.12241038947984794749)55:0.04903477415252657790)94:0.07684545963118714906,((eukSRP54@XP_023304254.1_Lucilia_cuprina [Opisthokonta]:0.00695708509674898990,((eukSRP54@XP_017138354.1_Drosophila_miranda [Opisthokonta]:0.00000100000050002909,eukSRP54@SPP87160.1_Drosophila_guanche [Opisthokonta]:0.00224920576477209829)86:0.01049607546042273173,eukSRP54@XP_002068627.1_Drosophila_willistoni [Opisthokonta]:0.00785419109510388652)99:0.02060822253215624858)97:0.02951808408835871617,(eukSRP54@ETN65735.1_Anopheles_darlingi [Opisthokonta]:0.01259786951402378646,eukSRP54@XP_021695011.1_Aedes_aegypti [Opisthokonta]:0.00794620146695331452)100:0.03630693131493448189)100:0.21786529537308010895)98:0.10268708982050818446)46:0.05259720949386657851,(eukSRP54@CAMNT_0031843689_Goniomonas_pacifica [Cryptophyta]:0.05582137716114156378,eukSRP54@GGUN01039927.1_Goniomonas_avonlea [Cryptophyta]:0.05723632301499093078)96:0.12663579543314976150)27:0.03943197763556244295,((((eukSRP54@XP_001018396.1_Tetrahymena_thermophila_SB210 [Alveolata]:0.13502846505575244462,eukSRP54@XP_004039207.1_Ichthyophthirius_multifiliis [Alveolata]:0.13072605543945506335)100:0.22239358691308572236,eukSRP54@GFYU01001334.1_Ancoracysta_twista [Ancoracysta]:0.20653242327258178324)65:0.04656355502281271652,((eukSRP54@RAW42240.1_Phytophthora_cactorum [Stramenopiles]:0.00910159017148943345,eukSRP54@XP_008893053.1_Phytophthora_parasitica_INRA-310 [Stramenopiles]:0.00000100000050002909)100:0.19846008360917100077,((eukSRP54@XP_002185775.1_Phaeodactylum_tricornutum_CCAP1055-1 [Stramenopiles]:0.15392683185722644268,eukSRP54@OEU17714.1_Fragilariopsis_cylindrus_CCMP1102 [Stramenopiles]:0.13097879626678532450)98:0.11667399559774058182,eukSRP54@CBN75372.1_Ectocarpus_siliculosus [Stramenopiles]:0.26003703907517861094)93:0.09092049683754083178)97:0.18493307886203017021)59:0.05643116395185220763,eukSRP54@NA_Spironema_sp [Hemimastigophora]:0.38765814988504682104)14:0.02678288891887467202)9:0.03255996973430940605)32:0.05940614440475135150,(eukSRP54@ANM86233.1_Stygiella_incarcerata [Jakobida]:0.34516663142688230881,((eukSRP54@GEZU01011656.1_Heterolobosea_BB2 [Heterolobosea]:0.19556382485758341816,eukSRP54@CAMNT_0005238523_Percolomonas_cosmopolitus_WS [Heterolobosea]:0.40657125221265599802)40:0.06575812254089344333,(eukSRP54@NA_Neovahlkampfia_damariscottae [Heterolobosea]:0.20192949337119939224,((eukSRP54@XP_002671296.1-corrected_Naegleria_gruberi [Heterolobosea]:0.05995234263047006379,eukSRP54@NF0102970_p1_Naegleria_fowleri [Heterolobosea]:0.05428335295132997312)99:0.10004729562719112679,eukSRP54@ACER01000537.1_Naegleri_gruberi [Heterolobosea]:0.49594000214286698691)100:0.15199059553513330489)79:0.08029699785930299871)15:0.02805371388281186623)7:0.00745540825375757676)27:0.07079876128132528423)53:0.06436392189870158986,eukSRP54@CAMNT_0031816087_Goniomonas_pacifica [Cryptophyta]:0.60145722787401612042)100:0.30195315490894575339,((((archSRP54@RLE51417.1_Candidatus_Verstraetearchaeota_archaeon [TACK]:0.10018207371081869206,archSRP54@RLE50907.1_Candidatus_Verstraetearchaeota_archaeon [TACK]:0.10436754178956880734)99:0.14915962832787563186,(archSRP54@OLS17826.1_Candidatus_Odinarchaeota_archaeon LCB4 [Asgard]:0.34682818260046749392,archSRP54@RLF14065.1_Thermoprotei_archaeon [TACK]:0.39144709716376463460)21:0.04088165370811717297)54:0.06845175470394572470,archSRP54@WP_110270916.1_Acidianus_brierleyi [TACK]:0.57228381154062313296)62:0.08602000679758477208,(archSRP54@WP_014734678.1_Pyrococcus_sp_ST04 [Euryarchaeota]:0.05483906998742614503,((archSRP54@WP_062370762.1_Thermococcus_guaymasensis [Euryarchaeota]:0.06857239002974301056,(archSRP54@WP_088864111.1_Thermococcus_barossii [Euryarchaeota]:0.06676396531057256223,archSRP54@WP_050002783.1_Thermococcus_eurythermalis [Euryarchaeota]:0.04866089584661124207)56:0.01464542136354716508)76:0.03894337496832581819,archSRP54@WP_087037458.1_Thermococcus_litoralis [Euryarchaeota]:0.07275006958869381646)89:0.05260453550517290439)100:0.23795970256408419119)78:0.08868565501009789687)68:0.05733156596977071612)100:0.59919849333091879107,(bacFfh@WP_006980240.1_Chthoniobacter_flavus [Verrucomicrobia]:0.27104944550472576115,(((bacFfh@WP_018970803.1_Rubritalea_marina [Verrucomicrobia]:0.06861601282297974391,bacFfh@WP_105044656.1_Rubritalea_profundi [Verrucomicrobia]:0.05093071422970910783)100:0.13300157121480149502,(bacFfh@WP_038136700.1_Verrucomicrobia_bacterium_SCGC_AAA168-F10 [Verrucomicrobia]:0.22185407208363891463,bacFfh@WP_035603039.1_Haloferula_sp_BvORR071 [Verrucomicrobia]:0.16543267860364330168)62:0.03272145395988324684)98:0.07200781290533743439,(bacFfh@CDD93459.1_Akkermansia_sp_CAG_344 [Verrucomicrobia]:0.01324260253017189165,bacFfh@WP_102742496.1_Akkermansia_muciniphila [Verrucomicrobia]:0.01046251143658831657)100:0.18445166829553985566)98:0.11816271234982209104)100:0.25387824614560361436)28:0.03367945072728110306)6:0.02949456527960540517,(bacFfh@WP_006928829.1_Caldithrix_abyssi [Calditrichaeota]:0.28716006447264719181,bacFfh@OPX24629.1_Candidatus_Latescibacteria_bacterium_4484_107 [Bacteroidetes]:0.30661881574676469464)76:0.12660377237279524243)9:0.03570460669389161068,(((bacFfh@ACX52518.1_Ammonifex_degensii_KC4 [Firmicutes]:0.22448517822486682705,(bacFfh@WP_054491650.1_Ardenticatena_maritima [Chloroflexi]:0.20895079665418289649,bacFfh@EFH87465.1_Ktedonobacter_racemifer_DSM_44963 [Chloroflexi]:0.45628386365230289012)55:0.09351802282559713853)22:0.05801890014681115870,((bacFfh@WP_109430892.1_Acidibacillus_sulfuroxidans [Firmicutes]:0.33237783287778077668,(b

cFfh@WP_072905435.1_Anaerobranca_californiensis [Firmicutes]:0.21622856347834545909,bacFfh@OPL10543.1_Firmicutes_bacterium_ML8_F2 [Firmicutes]:0.39823561833473652971)24:0.02915659568942711999)15:0.04448983146445374681,(((((bacFfh@WP_022786100.1_Clostridiales_bacterium_NK3B98 [Firmicutes]:0.30986032706575133977,bacFfh@WP_117520369.1_Ruminococcus_sp_AF43-11 [Firmicutes]:0.23765656218432265612)63:0.09571025188665382988,((bacFfh@WP_058486324.1_Defluviitalea_phaphyphila [Firmicutes]:0.21389629682550304057,bacFfh@WP_072469189.1_Urinacoccus_massiliensis [Firmicutes]:0.25907118140055379607)21:0.02039181207661447046,((bacFfh@WP_013275867.1_Thermosediminibacter_oceani [Firmicutes]:0.23445338079901859030,bacFfh@CDC00866.1_Firmicutes_bacterium_CAG_41 [Firmicutes]:0.22741694980550128391)3:0.03830971997767798620,(bacFfh@WP_074910001.1_Proteiniclasticum_ruminis [Firmicutes]:0.31557499133907213062,(bacFfh@WP_054875147.1_Oxobacter_pfennigii [Firmicutes]:0.19954810517622539878,bacFfh@WP_008908195.1_Caloramator_australicus [Firmicutes]:0.14970941127363923684)42:0.04044225414372967092)28:0.04477356722681762741)6:0.02766823453807934485)5:0.02941468969917205092)7:0.02583134254612083971,(bacFfh@PWM50529.1_Clostridiales_bacterium [Firmicutes]:0.21918480044049776234,bacFfh@KJS18981.1_Clostridiaceae_bacterium_BRH_c20a [Firmicutes]:0.15992628612541989752)93:0.09161603329861035816)13:0.05107690926990305497,((bacFfh@WP_038091674.1_Tumebacillus_flagellatus [Firmicutes]:0.13715178585706244707,(bacFfh@WP_015891890.1_Brevibacillus_brevis [Firmicutes]:0.12259555229515908814,bacFfh@WP_120461224.1_Paenibacillus_aceti [Firmicutes]:0.18855540588932589996)79:0.04291933532316644095)56:0.03676565145440735538,((bacFfh@WP_007505027.1_Caldalkalibacillus_thermarum [Firmicutes]:0.09014142415508810680,(bacFfh@WP_116552983.1_Pueribacillus_theae [Firmicutes]:0.09740059773603690352,(bacFfh@WP_109984449.1_Gracilibacillus_dipsosauri [Firmicutes]:0.11959150408535357268,(bacFfh@PKL00150.1_Tenericutes_bacterium_HGW-Tenericutes-1 [Tenericutes]:0.41916817082372453962,(bacFfh@WP_015076211.1_Carnobacterium_maltaromaticum [Firmicutes]:0.12683274106437772422,(bacFfh@WP_050441216.1_Streptococcus_pneumoniae [Firmicutes]:0.29457548562195834396,bacFfh@WP_086120482.1_Lactobacillus_reuteri [Firmicutes]:0.23541359739288042485)88:0.09499525035844319509)98:0.11371015702311447482)53:0.08268293591646443119)40:0.05985645950224335876)50:0.08232518803174114497)40:0.04365343568587519429,bacFfh@WP_089967246.1_Lihuaxuella_thermophila [Firmicutes]:0.17455691814111864990)17:0.03295442615824342725)31:0.06583979580024702793)2:0.02667819594205961264,((bacFfh@WP_046498292.1_Syntrophomonas_zehnderi [Firmicutes]:0.21552832762356066709,(bacFfh@WP_106004935.1_Moorella_humiferrea [Firmicutes]:0.04496227255198410877,bacFfh@WP_062283840.1_Moorella_mulderi [Firmicutes]:0.04492278556513797094)100:0.14805271343129991490)34:0.02933509196476345726,bacFfh@WP_018702437.1_Anaeromusa_acidaminophila [Firmicutes]:0.21492690231733452277)27:0.02485613143047987056)4:0.02834004818966886011)15:0.07074858523552295109)3:0.01974360784778210476,bacFfh@OIP71264.1_Candidatus_Atribacteria_bacterium_CG2_30_33_13 [Atribacteria]:0.76891034643900457723)13:0.02321414143424079674)8:0.02836379412034684133,bacFfh@WP_049675438.1_Desulfocarbo_indianensis [Deltaproteobacteria]:0.36844634864642622496)97:0.10384222070033163887,(((((bacFfh@PZP86081.1_Azospirillum_brasilense [Alphaproteobacteria]:0.33149573191941661410,(bacFfh@WP_027134478.1_Geminicoccus_roseus [Alphaproteobacteria]:0.21847218144390179173,(bacFfh@WP_088559973.1_Arboriscoccus_pini [Alphaproteobacteria]:0.00000100000050002909,bacFfh@WP_088559973.1_Arboricoccus_pini [Alphaproteobacteria]:0.00000100000050002909)100:0.19620130324190601656)85:0.08000476287691558885)46:0.08442954691468888828,((bacFfh@WP_032113236.1_Candidatus_Paracaedibacter_symbiosus [Alphaproteobacteria]:0.24518769809336152243,((bacFfh@OJX13986.1_Caedibacter_sp_37-49 [Alphaproteobacteria]:0.20187962197147057974,bacFfh@WP_085783826.1_Candidatus_Nucleicultrix_amoebiphila [Alphaproteobacteria]:0.18433339663861775248)73:0.04968137692259173843,bacFfh@OYZ36327.1_Alphaproteobacteria_bacterium_16-39-46 [Alphaproteobacteria]:0.25564334702189595028)51:0.04531218527995112960)29:0.02753134592448096968,(((bacFfh@PIR38902.1_Alphaproteobacteria_bacterium_CG11_big_fil_rev_8_21_14_0_20_39_49 [Alphaproteobacteria]:0.20773835202761120611,bacFfh@KKB96089.1_Arcanobacter_lacustris [Alphaproteobacteria]:0.26237791452271397308)43:0.05367739495097856306,(bacFfh@PLX30515.1_Alphaproteobacteria_bacterium [Alphaproteobacteria]:0.33812105500490186039,bacFfh@OUW71296.1_Rickettsiales_bacterium_TMED211 [Alphaproteobacteria]:0.47517545264136906713)13:0.04990670474059752393)6:0.02828545684513921604,((bacFfh@OUT75042.1_Rhizobiales_bacterium_TMED25 [Alphaproteobacteria]:0.08470304931306524288,(bacFfh@OUX67412.1_Rhizobiales_bacterium_TMED227 [Alphaproteobacteria]:0.07968464761136118124,bacFfh@OUT82378.1_Rhizobiales_bacterium_TMED28 [Alphaproteobacteria]:0.08605511804312154789)57:0.02256742974707008284)100:0.26679821116508573020,(bacFfh@OJV16027.1_Alphaproteobacteria_bacterium_33-17 [Alphaproteobacteria]:0.42128179789233560770,(bacFfh@WP_065432712.1_Ehrlichia_ruminantium [Alphaproteobacteria]:0.29794961347252874040,bacFfh@WP_025264366.1_Wolbachia_endosymbiont_of_Onchocerca_volvulus [Alphaproteobacteria]:0.18473443275726775248)100:0.24493085206650938579)22:0.05759710045894100849)7:0.04630212556712862138)5:0.04340668599383821352)14:0.06041293453998693164)2:0.04572026039144409804,((bacFfh@PPR36214.1_Alphaproteobacteria_bacterium_MarineAlpha9_Bin6 [Alphaproteobacteria]:0.32732736602708351059,(bacFfh@PPR20700.1_Alphaproteobacteria_bacterium_MarineAlpha10_Bin2 [Alphaproteobacteria]:0.03704403198618418103,bacFfh@PPR25390.1_Alphaproteobacteria_bacterium_MarineAlpha10_Bin1 [Alphaproteobacteria]:0.05536392055439806614)100:0.17019568484924185725)26:0.06237888825681214833,((((((bacFfh@WP_119283627.1_Rhodospirillaceae_bacterium_SYSU_D60006 [Alphaproteobacteria]:0.20002150106619961956,((bacFfh@WP_068791160.1_unknown alphaproteobacterium [Alphaproteobacteria]:0.13888514196734047523,bacFfh@WP_027287498.1_Rhodovibrio_salinarum [Alphaproteobacteria]:0.24675209811166379237)75:0.06154874658780324498,bacFfh@WP_046506348.1_Kiloniella_litopenaei [Alphaproteobacteria]:0.18061008437852058006)33:0.01930271547132699980)51:0.06308811577487272171,((((bacFfh@WP_073953247.1_Thalassospira_sp_TSL5-1 [Alphaproteobacteria]:0.18898733936019676549,bacFfh@OFX07559.1_Alphaproteobacteria_bacterium_RIFOXYD12_FULL_60_8 [Alphaproteobacteria]:0.21476733496942135293)50:0.05009601966173665016,(((((bacFfh@CDB53938.1_Azospirillum_sp_CAG_239 [Alphaproteobacteria]:0.05723233798081989177,(bacFfh@OLA79528.1_Azospirillum_sp_47_25 [Alphaproteobacteria]:0.00000100000050002909,bacFfh@CDB39986.1_Azospirillum_sp_CAG_260 [Alphaproteobacteria]:0.00485858335986054553)100:0.07458309303526050593)100:0.24671422433849254929,bacFfh@PHY00942.1_Rhodospirillaceae_bacterium [Alphaproteobacteria]:0.28313681841387045024)36:0.06501148139344527233,(bacFfh@WP_092613925.1_Roseospirillum_parvum [Alphaproteobacteria]:0.19458724154503515003,bacFfh@WP_019645789.1_Novispirillum_itersonii [Alphaproteobacteria]:0.13709609522595003761)32:0.03146999612859090190)10:0.01320288189351879159,(bacFfh@CCZ21287.1_Acetobacter_sp_CAG_977 [Alphaproteobacteria]:0.22162773697006174500,bacFfh@WP_041795188.1_Pararhodospirillum_photometricum [Alphaproteobacteria]:0.21568514374016037327)11:0.01323313641933793888)10:0.03486253695833344685,((bacFfh@WP_043360684.1_Belnapia_sp_F-4-1 [Alphaproteobacteria]:0.14652409820271486418,((bacFfh@WP_008853607.1_Commensalibacter_intestini [Alphaproteobacteria]:0.00000100000050002909,bacFfh@WP_086632043.1_Commensalibacter_intestini [Alphaproteobacteria]:0.00000100000050002909)100:0.00982867680491733044,bacFfh@WP_034336672.1_Commensalibacter_sp_MX01 [Alphaproteobacteria]:0.00869500274160522717)100:0.27336485842278490654)95:0.12602284187745224631,((((bacFfh@OJX70273.1_Magnetospirillum_sp_64-120 [Alphaproteobacteria]:0.03655935475219353192,bacFfh@WP_024081694.1_Magnetospirillum_gryphiswaldense [Alphaproteobacteria]:0.03006411780915559800)82:0.01730431531510967860,bacFfh@WP_068497751.1_Magnetospirillum_moscoviense [Alphaproteobacteria]:0.04317022798691682456)99:0.04935064288523026421,((bacFfh@WP_002727862.1_Phaeospirillum_molischianum [Alphaproteobacteria]:0.00480154024659963305,bacFfh@WP_074764996.1_Phaeospirillum_fulvum [Alphaproteobacteria]:0.00722773598730662957)100:0.07044848442845376424,(bacFfh@WP_011386413.1_Magnetospirillum_magneticum [Alphaproteobacteria]:0.04286429439238273464,bacFfh@WP_068493492.1_Magnetospirillum_marisnigri [Alphaproteobacteria]:0.03140157182693332355)75:0.02090645425949244976)86:0.03725782115116062221)100:0.12354047195963945016,(bacFfh@WP_028877797.1_Terasakiella_pusilla [Alphaproteobacteria]:0.04249586681860685611,bacFfh@WP_069189220.1_Terasakiella_sp_PR1 [Alphaproteobacteria]:0.06298700596674158825)100:0.19055133653884936962)14:0.02061514319557533961)1:0.02103413460939811130)1:0.02289244485262091275)9:0.04481657390277641456,(bacFfh@WP_069956818.1_Magnetovibrio_blakemorei [Alphaproteobacteria]:0.19705235244208724188,(bacFfh@OUT52366.1_Rhodospirillaceae_bacterium_TMED8 [Alphaproteobacteria]:0.30046202708093405764,bacFfh@OHC73580.1_Rhodospirillales_bacterium_RIFCSPLOWO2_02_FULL_58_16 [Alphaproteobacteria]:0.19403869082569533133)38:0.05159828827791246991)66:0.05667156774995202317)3:0.02732972587288113112,((bacFfh@WP_028466142.1_Nisaea_denitrificans [Alphaproteobacteria]:0.09212230091560585921,bacFfh@OUU28491.1_Candidatus_Endolissoclinum_sp_TMED37 [Alphaproteobacteria]:0.23763747423795317237)99:0.14355094516255401116,((bacFfh@OUX71121.1_Rhodospirillaceae_bacterium_TMED140 [Alphaproteobacteria]:0.23199933799668856493,((bacFfh@WP_015467793.1_Micavibrio_aeruginosavorus [Alphaproteobacteria]:0.13893902330058899297,bacFfh@PCJ00252.1_Alphaproteobacteria_bacterium [Alphaproteobacteria]:0.22727365977140664977)85:0.05182570051634433073,(bacFfh@OIN86659.1_Alphaproteobacteria_bacterium_CG1_02_46_17 [Alphaproteobacteria]:0.20431577926718369476,bacFfh@PZQ45682.1_Micavibrio_aeruginosavorus [Alphaproteobacteria]:0.14080107393982072006)58:0.04572533300413781504)98:0.10272045606493299119)31:0.03852009251750257435,((bacFfh@WP_012973141.1_Azospirillum_lipoferum [Alphaproteobacteria]:0.10164258290871171220,bacFfh@WP_094454746.1_Niveispirillum_lacus [Alphaproteobacteria]:0.18572691198352850317)76:0.07900086382768529980,(bacFfh@WP_092823393.1_Rhodospirillales_bacterium_URHD0017 [Alphaproteobacteria]:0.25342384826583741475,bacFfh@WP_108794692.1_Rhodospirillaceae_bacterium_Spongia-Bin9 [Alphaproteobacteria]:0.19227699965932179560)31:0.04520670166217715530)8:0.02988604283806478232)1:0.01672053710028452433)0:0.02060520776834480136)0:0.01342763802320352112)1:0.01874628615426642692,(((bacFfh@PDH20339.1_Pelagibacterales_bacterium_MED-G40 [Alphaproteobacteria]:0.45067057728212489565,bacFfh@PPR79452.1_Alphaproteobacteria_bacterium_MarineAlpha2_Bin1 [Alphaproteobacteria]:0.23572228630084660073)48:0.13727076353406450493,bacFfh@PCI43326.1_Alphaproteobacteria_bacterium [Alphaproteobacteria]:0.12944446242380133749)15:0.12021804663762730714,((((bacFfh@RCL83452.1_PS1_clade_bacterium [Alphaproteobacteria]:0.29455407488919088665,bacFfh@PKQ09217.1_Alphaproteobacteria_bacterium_HGW-Alphaproteobacteria-12 [Alphaproteobacteria]:0.16244450380290217928)30:0.06473591792653837773,((((bacFfh@WP_085770433.1_Methylocystis_bryophila [Alphaproteobacteria]:0.30208035714325970345,(bacFfh@WP_111197444.1_Rhizobiales_bacterium_KCTC_52945 [Alphaproteobacteria]:0.17994406182758168611,bacFfh@WP_038035438.1_Thermopetrobacter_sp_TC1 [Alphaproteobacteria]:0.24427568051475853639)58:0.03522600930757727872)27:0.02849259340596987586,bacFfh@GBE42717.1_Bacterium_BMS3Bbin10 [unassigned]:0.21451209024058034980)10:0.02077333255924478095,(bacFfh@WP_013420482.1_Rhodomicrobium_vannielii [Alphaproteobacteria]:0.14867182820519056485,(bacFfh@OUU83527.1_Hyphomicrobiaceae_bacterium_TMED74 [Alphaproteobacteria]:0.13492575507462356565,bacFfh@WP_099557780.1_Hartmannibacter_diazotrophicus [Alphaproteobacteria]:0.17327014437354035237)76:0.05186394684880499262)13:0.01806099762625910238)21:0.02951181069007501392,(bacFfh@PCJ00993.1_OCS116_cluster_bacterium [Alphaproteobacteria]:0.00968572529423322899,bacFfh@PCI85686.1_Rhizobiales_bacterium [Alphaproteobacteria]:0.00714102321430802080)100:0.23289422736335529951)20:0.01466933707376095151)25:0.03352872650123174536,((bacFfh@WP_116392064.1_Parvularcula_sp_SM1705 [Alphaproteobacteria]:0.24622244417176000764,bacFfh@RCL81099.1_SAR116_cluster_bacterium [Alphaproteobacteria]:0.34199020114103206858)19:0.04364063847224702170,((bacFfh@OJT95039.1_Alphaproteobacteria_bacterium_65-7 [Alphaproteobacteria]:0.19137954986298064131,bacFfh@OQW59094.1_Proteobacteria_bacterium_HN_bin10 [Proterobacteria]:0.32404501005514224632)48:0.07236168753169663903,bacFfh@WP_018634118.1_Neomegalonema_perideroedes [Alphaproteobacteria]:0.23892060663817668575)13:0.03859875295811711349)12:0.04125148822261531789)37:0.04486574791224788583,((bacFfh@OUR76855.1_Alphaproteobacteria_bacterium_46_93_T64 [Alphaproteobacteria]:0.06199638972488966809,bacFfh@WP_025896931.1_Sneathiella_glossodoripedis [Alphaproteobacteria]:0.03704600446370887035)100:0.19342058857260610583,(bacFfh@WP_109920731.1_Zavarzinia_compransoris [Alphaproteobacteria]:0.18547339537000681609,bacFfh@ANK81659.1_Rhizobiales_bacterium_NRL2 [Alphaproteobacteria]:0.19076631822253362070)44:0.02623040995856400562)39:0.03359560767239030965)5:0.00996896498075337638)2:0.03002092876328434426)0:0.02279681435644664855,(bacFfh@PCJ58386.1_Rhodospirillaceae_bacterium [Alphaproteobacteria]:0.35912856596034542500,((bacFfh@WP_072596811.1_Sphingomonas_sp_JJ-A5 [Alphaproteobacteria]:0.12788205667025284717,((bacFfh@WP_011240910.1_Zymomonas_mobilis [Alphaproteobacteria]:0.02670301988576014723,bacFfh@WP_013933516.1_Zymomonas_mobilis [Alphaproteobacteria]:0.03860045641481286982)100:0.14217410527730289793,((bacFfh@WP_116091360.1_Sphingomonas_crusticola [Alphaproteobacteria]:0.11292944795681811310,bacFfh@WP_119532804.1_Sphingomonas_sp_DAC4 [Alphaproteobacteria]:0.18964294089385017039)51:0.04013497927023538386,(bacFfh@WP_022691338.1_Sphingomonas-like_bacterium_B12 [Alphaproteobacteria]:0.09664635728139421567,((bacFfh@WP_089215287.1_Sphingopyxis_indica [Alphaproteobacteria]:0.03167822643739844068,bacFfh@WP_076073722.1_Sphingopyxis_granuli [Alphaproteobacteria]:0.03546514424309748365)100:0.08564950413771868498,(bacFfh@SCW52489.1_Sphingobium_faniae [Alphaproteobacteria]:0.03432270793410992227,(bacFfh@WP_010335520.1_Sphingobium_yanoikuyae [Alphaproteobacteria]:0.02060576861801377904,bacFfh@WP_014076705.1_Sphingobium_sp_SYK-6 [Alphaproteobacteria]:0.09497634043060994291)59:0.03362729176351508115)95:0.05638714911176914008)71:0.04563707567668908588)31:0.01999589949210514583)56:0.03374903178936680681)52:0.03798505528899840528)100:0.16005087492953654071,bacFfh@WP_115937331.1_Aestuariispira_insulae [Alphaproteobacteria]:0.17926396266419791781)8:0.01698752786792692357)3:0.02011332096427535104)0:0.02523503623512924829,bacFfh@PPR12333.1_Alphaproteobacteria_bacterium_MarineAlpha11_Bin1 [Alphaproteobacteria]:0.30207434254402099061)1:0.01668541327631642771,bacFfh@WP_014746762.1_Tistrella_mobilis [Alphaproteobacteria]:0.25483217876479485886)1:0.02330152920533613142)4:0.03068502063323715057)17:0.03868327809092856479,bacFfh@PPR14985.1_Alphaproteobacteria_bacterium_MarineAlpha9_Bin3 [Alphaproteobacteria]:0.40670460468201691251)44:0.04599943545608580625,(((mtFfh@NA_Percolomonas_cosmopolitus_strain_AE [Heterolobosea]:1.15768691420321601093,(((((mtFfh@NA_Naegleria_lovaniensis [Heterolobosea]:0.01655319347304744920,mtFfh@NA_Naegleria_fowleri [Heterolobosea]:0.01525629688206948074)95:0.04388881845265728970,mtFfh@NA_Naegleri_gruberi [Heterolobosea]:0.10357460134114643280)100:0.43408905990924029350,mtFfh@NA_Neovahlkampfia_damariscottae [Heterolobosea]:0.49842347508102180509)55:0.07308546077734406898,mtFfh@NA_Heterolobosea_BB2 [Heterolobosea]:0.37451103905423105411)59:0.08135992909456273015,(mtFfh@NA_Pharyngomonas_kirbyi [Heterolobosea]:0.45638231596096673348,(mtFfh@NA_Percolomonas_cosmopolitus_strain_WS [Heterolobosea]:0.39870339200331567087,mtFfh@NA_Percolomonas_ex_Nitzchia_Cheng_2013 [Heterolobosea]:0.62

21459849991090341)100:0.66572616195639910952)55:0.08523873983118319952)66:0.13048380004322601700)66:0.05672478714744148831,((mtFfh@NA_Goniomonas_pacifica [Cryptophyta]:0.69132747899764079857,mtFfh@NA_Goniomonas_avonlea [Cryptophyta]:0.38737904879198153951)97:0.27982678333116739466,(mtFfh@NA_Spironema_sp [Hemimastigophora]:0.66908471111622747074,mtFfh@NA_Hemimastix_kukwesjijk [Hemimastigophora]:0.43869100939017174889)100:0.60229153722551953543)25:0.05981027611510440073)30:0.06494532764856528195,(mtFfh@NA_Ancoracysta_twista [Ancoracysta]:0.30515097510354205479,mtFfh@NA_Ancoracysta-related_Colp-4b [Ancoracysta]:0.42908560878312873577)100:0.37712151572907054442)99:0.21363546542722572363)99:0.11378651170111450663)100:0.20833638441315102674,bacFfh@WP_025769383.1_Thioalkalivibrio_sp_HK1 [Gammaproteobacteria]:0.30435084378756194212);

**Phylogenetic tree from Fig. 3C (RAxML, LG4X model, 500 rapid bootstraps, 217 OTUs)**

((ptFtsY@XP_005705405.1_Galdieria_sulphuraria [Archaeplastida]:0.94454101606672691283,(((((ptFtsY@EWM29383.1_Nannochloropsis_gaditana [Stramenopiles]:0.35185280970563936886,ptFtsY@CBJ31918.1_Ectocarpus_siliculosus [Stramenopiles]:0.32525754718476523886)63:0.06103827972487478604,ptFtsY@XP_002296627.1_Thalassiosira_pseudonana_CCMP1335 [Stramenopiles]:0.56962149621896152052)43:0.04445472532189077841,ptFtsY@XP_009040860.1_Aureococcus_anophagefferens [Stramenopiles]:0.33378751794260330676)46:0.06100176135577733277,ptFtsY@CEM32712.1_Vitrella_brassicaformis_CCMP3155 [Alveolata]:0.36545741600075892785)83:0.10734321555864553832,(ptFtsY@XP_005714040.1_Chondrus_crispus [Archaeplastida]:0.11943855754644448763,ptFtsY@PXF44704.1_Gracilariopsis_chorda [Archaeplastida]:0.12305488782994263020)100:0.29788921299927173525)51:0.07428081796730477693)48:0.07157287706767480329,((((bacFtsY@PCI95412.1_Candidatus_Aerophobetes_bacterium [Aerophobetes]:0.45550150281155515897,bacFtsY@WP_041017695.1_Criblamydia_sequanensis [Chlamydiae]:0.49116420989959336252)82:0.23043844623169593144,(((bacFtsY@WP_088252003.1_Fimbriiglobus_ruber [Planctomycetes]:0.40466935859714758816,(bacFtsY@RPG16414.1_FtsY_Phycisphaera_sp_TMED9 [Planctomycetes]:0.44129095958295344593,bacFtsY@RMH26650.1_Planctomycetes_bacterium [Planctomycetes]:0.34649243139129071167)97:0.23506899585663279528)50:0.14765393420740022257,(bacFtsY@OYZ20489.1_Bdellovibrio_sp_28-41-41 [Deltaproteobacteria]:0.67659422783721223649,(bacFtsY@WP_124447410.1_Paucibacter_sp_KBW04 [Betaproteobacteria]:0.02570527839931696298,(bacFtsY@OWQ45088.1_Mitsuaria_noduli [Betaproteobacteria]:0.05139179503372987012,((bacFtsY@WP_066336977.1_Azohydromonas_lata [Betaproteobacteria]:0.09214350196310926167,bacFtsY@WP_089417744.1_Vitreoscilla_filiformis [Betaproteobacteria]:0.12615344460798397219)44:0.03593693011943028398,(bacFtsY@PZP35629.1_Roseateles_depolymerans [Betaproteobacteria]:0.03499232774299040200,bacFtsY@WP_056203513.1_Pelomonas_sp_Root1237 [Betaproteobacteria]:0.06547132682673947879)38:0.03619830957602756388)20:0.04449025212753128511)14:0.03803086770392006405)100:0.48530190397422467985)28:0.09201515381943431438)5:0.02340814280761376737,(bacFtsY@OGP30931.1_Deltaproteobacteria_bacterium_GWC2_42_11 [Deltaproteobacteria]:0.42985569897281206142,(((((((((bacFtsY@WP_121469822.1_Edaphobacter_dinghuensis [Acidobacteria]:0.21016269400560375069,(bacFtsY@WP_081490725.1_Terriglobus_roseus [Acidobacteria]:0.01852391296327955683,bacFtsY@WP_074656074.1_Terriglobus_roseus [Acidobacteria]:0.01171052418657935086)100:0.15013932940522539683)100:0.34338984408657863279,(bacFtsY@WP_031499580.1_Bryobacter_aggregatus [Acidobacteria]:0.22271632936485746823,bacFtsY@WP_020721417.1_Acidobacteriaceae_bacterium_KBS_96 [Acidobacteria]:0.11919603795033360816)89:0.12850276014029793359)46:0.06343955841124027106,bacFtsY@ANM28936.1_Acidobacteria_bacterium_Mor1 [Acidobacteria]:0.56377545282479279987)24:0.08188979374149057966,(bacFtsY@PYS67814.1_Acidobacteria_bacterium [Acidobacteria]:0.29133511660751376660,bacFtsY@PIE91415.1_Acidobacteria_bacterium [Acidobacteria]:0.49768682256514462647)50:0.06138409091273514667)29:0.12987624657794472061,((mtFtsY@NA_1_Hemimastix_kukwesjijk [Hemimastigophora]:0.70351329674334028841,(((mtFtsY@NA_partial_Percolomonas_ex_Nitzchia_Cheng_2013 [Heterolobosea]:0.64385141645968180235,mtFtsY@CAMNT_0005246107_Percolomonas_cosmopolitus_WS [Heterolobosea]:0.38696898042101385284)94:0.34988327813776004538,(mtFtsY@NA_Neovahlkampfia_damariscottae [Heterolobosea]:0.58650749373735211467,(mtFtsY@CAMNT_0005204377-extended_Percolomonas_cosmopolitus_AE [Heterolobosea]:1.47128219660476622366,((mtFtsY@NA_Naegleria_fowleri [Heterolobosea]:0.01393889288339679802,mtFtsY@NA_Naegleria_lovaniensis [Heterolobosea]:0.01438125863653981484)96:0.08817048459033056951,mtFtsY@NA_Naegleria_gruberi [Heterolobosea]:0.02068523507410521822)100:0.39417320825776736015)22:0.13401047396600843098)37:0.11683465085877559053)36:0.11546299260937223363,(mtFtsY@NA_Pharyngomonas_kirbyi [Heterolobosea]:0.40637039503548061381,mtFtsY@NA_Heterolobosea_BB2 [Heterolobosea]:0.21009281217899025695)85:0.15839489211687965420)35:0.11745782858756378952)18:0.15597768553819593706,((mtFtsY@NA_partial_Goniomonas_pacifica [Cryptophyta]:1.19080926550012367038,mtFtsY@NA_Ancoracysta-related_Colp-4b [Ancoracysta]:0.85304346074263659450)17:0.07723292728660105522,(mtFtsY@NA_N-terminus_Ancoracysta_twista [Ancoracysta]:0.72681957307310285366,mtFtsY@QUTJ01025227.1_Goniomonas_avonlea [Cryptophyta]:0.41598660469412362950)38:0.28963940625120104810)31:0.14814347919417508725)10:0.17329776507055760781)1:0.05485590892112999356,(((((((bacFtsY@ARM82543.1_Marinobacter_salarius [Gammaproteobacteria]:0.04606886893609869749,bacFtsY@KXS51830.1_Marinobacter_sp_T13-3 [Gammaproteobacteria]:0.02678593970076582897)98:0.13151941655091234362,bacFtsY@WP_091826228.1_Marinobacterium_georgiense [Gammaproteobacteria]:0.11277920873927094192)73:0.10092894122633762344,(bacFtsY@WP_097790462.1_Halomonas_beimenensis [Verrucomicrobia]:0.09908364026190706608,(bacFtsY@WP_107335894.1_Halomonas_sp_SF2003 [Verrucomicrobia]:0.00000100000050002909,bacFtsY@KGA01103.1_Cobetia_amphilecti [Gammaproteobacteria]:0.00000100000050002909)100:0.10610302868015232536)96:0.07801392847853989843)39:0.04981949643288323404,(bacFtsY@WP_086487265.1_Thioflexothrix_psekupsii [Gammaproteobacteria]:0.22053431388860822437,bacFtsY@WP_116686709.1_contaminant of Flavobacteriaceae_bacterium Hp12 genome [Gammaproteobacteria]:0.22962968567648578411)50:0.05987211557422363745)26:0.04267024380760728319,(bacFtsY@PPI88525.1_Pantoea_sp_SoEO [Gammaproteobacteria]:0.42418750337851929277,(bacFtsY@WP_094041167.1_Zobellella_denitrificans [Gammaproteobacteria]:0.10107964858384040074,bacFtsY@WP_091986549.1_Pseudoalteromonas_denitrificans [Gammaproteobacteria]:0.16807652493225677670)93:0.06441193085471176583)51:0.05742136413128024613)18:0.05939780482399936029,(bacFtsY@WP_113743312.1_Anaerobiospirillum_thomasii [Gammaproteobacteria]:0.36562186514807348825,(bacFtsY@WP_095208401.1_Luteimonas_sp_JM171 [Gammaproteobacteria]:0.37655097377087848320,bacFtsY@OUX68934.1_Oceanospirillales_bacterium_TMED91 [Gammaproteobacteria]:0.41396219256656791030)37:0.05893309926509016777)11:0.07653006328218002474)80:0.10188658052099602846,((((bacFtsY@AIL12880.1_Candidatus_Paracaedimonas_acanthamoebae [Alphaproteobacteria]:0.46348709375050012316,(bacFtsY@KRS17267.1_Roseovarius_indicus [Alphaproteobacteria]:0.07140591532964667143,bacFtsY@WP_025048799.1_Sulfitobacter_mediterraneus [Alphaproteobacteria]:0.05858257785139305007)100:0.23609510737601774322)38:0.06836263001508312287,(bacFtsY@GBD43248.1_Bacterium_HR40 [unassigned]:0.39453092158732283590,(bacFtsY@OUU28495.1_Candidatus_Endolissoclinum_sp_TMED37 [Alphaproteobacteria]:0.37531817566623754123,(bacFtsY@EPY01636.1_Phaeospirillum_fulvum_MGU-K5 [Alphaproteobacteria]:0.18801287433028154861,(bacFtsY@OFX10022.1_Alphaproteobacteria_bacterium_RIFOXYD12_FULL_60_8 [Alphaproteobacteria]:0.15857326062643675724,bacFtsY@WP_092615803.1_Roseospirillum_parvum [Alphaproteobacteria]:0.24526829108377418143)57:0.05422298802788644290)58:0.06727840885371025315)34:0.07793675194071349177)30:0.02580965317112756188)55:0.03666016555994341952,(((bacFtsY@WP_113333951.1_Rhizobiales_bacterium [Alphaproteobacteria]:0.15183763671556493868,bacFtsY@RCL01761.1_Candidatus_Tokpelaia_sp_JSC085 [Alphaproteobacteria]:0.33306383831726305012)71:0.11367253093961629529,((bacFtsY@WP_109793879.1_Rhizobiales_bacterium [Alphaproteobacteria]:0.27109892674904573218,bacFtsY@WP_108880431.1_Anderseniella_sp_Alg231-50 [Alphaproteobacteria]:0.22939869655293590456)45:0.06341644717035492540,((bacFtsY@WP_029041189.1_Cucumibacter_marinus [Alphaproteobacteria]:0.17411122528749567451,bacFtsY@PPD07903.1_Hyphomicrobium_sp [Alphaproteobacteria]:0.26593889498316169995)9:0.02661156542513962511,((bacFtsY@WP_088520031.1_Rhodoblastus_acidophilus [Alphaproteobacteria]:0.18817304167072460008,(bacFtsY@WP_115516251.1_Pseudolabrys_sp_GY_H [Alphaproteobacteria]:0.26991294556783385072,(bacFtsY@SKC16052.1_Bosea_thiooxidans [Alphaproteobacteria]:0.16342940723027576944,(bacFtsY@WP_013168132.1_Starkeya_novella [Alphaproteobacteria]:0.12629672339212622001,(bacFtsY@WP_024277252.1_Xanthobacter_sp_126 [Alphaproteobacteria]:0.04311002295703093151,bacFtsY@WP_011996071.1_Xanthobacter_autotrophicus [Alphaproteobacteria]:0.07564246860693527030)99:0.13157203342559267711)88:0.08943491770078408620)71:0.05146984826762828619)32:0.02940947462776777668)40:0.05223613706871090695,bacFtsY@WP_099557887.1_Hartmannibacter_diazotrophicus [Alphaproteobacteria]:0.14937236422124333668)11:0.01644011162069824311)17:0.05766220995304473934)5:0.01116637696143028304)36:0.09909281974889994071,(bacFtsY@WP_083773102.1_Hirschia_baltica [Alphaproteobacteria]:0.00000100000050002909,bacFtsY@ACT57804.1_Hirschia_baltica_ATCC_49814 [Alphaproteobacteria]:0.00000100000050002909)100:0.41919222248739596015)34:0.04435503228515318835)64:0.20513539385458984254,bacFtsY@SME87908.1_Pseudobacteriovorax_antillogorgiicola [Deltaproteobacteria]:0.53309887147371526339)43:0.15693487181202739333)3:0.04202083736492340932)0:0.04112288590184313974,(((bacFtsY@KPK53060.1_Myxococcales_bacterium_SG8_38_1 [Deltaproteobacteria]:0.56809138098257538907,bacFtsY@PID38167.1_Proteobacteria_bacterium [Proterobacteria]:0.37443545459589405722)49:0.11658781207422708182,bacFtsY@WP_011985037.1_Anaeromyxobacter_sp_Fw109-5 [Deltaproteobacteria]:0.35637103356927329889)15:0.11593079592082629337,(bacFtsY@WP_083764053.1_Syntrophobacter_fumaroxidans [Deltaproteobacteria]:0.37813486808594382049,((bacFtsY@PIP45503.1_Deltaproteobacteria_bacterium_CG23_combo_of_CG06-09_8_20_14_all_51_20 [Deltaproteobacteria]:0.32387486604128468137,bacFtsY@PIE60181.1_Desulfobulbus_propionicus [Deltaproteobacteria]:0.33120386274306423680)40:0.04692121123561185381,(bacFtsY@ABC77884.1_Syntrophus_aciditrophicus_SB [Deltaproteobacteria]:0.26072023178720254988,bacFtsY@PIP06279.1_Syntrophobacteraceae_bacterium_CG23_combo_of_CG06-09_8_20_14_all_50_8 [Deltaproteobacteria]:0.16356873989032924555)100:0.29281572947027689757)12:0.02713510822111351792)30:0.06545836170242090579)1:0.07342571626221817971)0:0.06588706420051601564,(bacFtsY@WP_012175138.1_Desulfococcus_oleovorans [Deltaproteobacteria]:0.45216582472399602377,(bacFtsY@OGC84905.1_Zixibacteria_bacterium_RBG_16_43_9 [Zixibacteria]:0.43141271611618609017,((bacFtsY@PSQ63899.1_Bacteroidetes_bacterium_QH_1_61_8 [Bacteroidetes]:0.33148187505071241565,(((bacFtsY@WP_114910387.1_Cardinium_endosymbiont_of_Sogatella_furcifera [Bacteroidetes]:0.43791829674639709546,bacFtsY@WP_103327589.1_Bacteroidetes_endosymbiont_of_Geopemphigus_sp [Bacteroidetes]:0.20548115767538358001)67:0.11647693427423926049,(bacFtsY@OUV76101.1_Flavobacteriales_bacterium_TMED123 [Bacteroidetes]:0.21824302013837246217,bacFtsY@PSR05731.1_Bacteroidetes_bacterium_SW_10_40_5 [Bacteroidetes]:0.28512019701732682631)77:0.08081942305475009469)39:0.03751072416120516256,((bacFtsY@OUU18192.1_Crocinitomicaceae_bacterium_TMED45 [Bacteroidetes]:0.31678877760107337913,bacFtsY@WP_100314957.1_Thermoflavifilum_aggregans [Bacteroidetes]:0.24661396850979580564)43:0.06542966661852409049,bacFtsY@OUV32974.1_Rhodothermaceae_bacterium_TMED105 [Bacteroidetes]:0.36514295120014755858)41:0.06860068091145635205)31:0.05940989202016508375)84:0.10905510552005796832,(((bacFtsY@KXK57805.1_Chlorobi_bacterium_OLB7 [Chlorobi]:0.22376339838774819690,(bacFtsY@OJX59420.1_Candidatus_Kapabacteria_thiocyanatum [Bacteroidetes]:0.16291028746771266111,bacFtsY@PKL79980.1_Ignavibacteriae_bacterium_HGW-Ignavibacteriae-4 [Ignavibacteriae]:0.27487560504274977102)87:0.11335708941332471589)77:0.13263129680229426843,(bacFtsY@OGU26318.1_Ignavibacteria_bacterium_GWA2_54_16 [Ignavibacteriae]:0.37230721323264803768,bacFtsY@PLX30570.1_Ignavibacteria_bacterium [Ignavibacteriae]:0.17082050309138668842)43:0.06042834436447503538)24:0.02677106171235033721,(bacFtsY@WP_092350764.1_Candidatus_Chrysopegis_kryptomonas [Kryptonia]:0.20635329145637021364,(bacFtsY@OQY74580.1_Ignavibacteriales_bacterium_UTCHB3 [Ignavibacteriae]:0.30364493569564765529,(bacFtsY@PKL82841.1_Ignavibacteriae_bacterium_HGW-Ignavibacteriae-3 [Ignavibacteriae]:0.44607202067719442518,bacFtsY@OGU83728.1_Ignavibacteria_bacterium_RBG_16_35_7 [Ignavibacteriae]:0.43518240024359916562)87:0.11731220074143715315)95:0.21401401656097304715)65:0.08664048036818343401)35:0.04246016865338544249)59:0.10104020998176012347)17:0.06756945656298547764)1:0.01665977784155703301)0:0.03905101608834647686,((((((bacFtsY@WP_073092242.1_Thermosyntropha_lipolytica [Firmicutes]:0.41004396512248841855,((((bacFtsY@WP_009554695.1_Lactobacillus_saerimneri [Firmicutes]:0.21366369940362406332,((bacFtsY@WP_103423367.1_Lactobacillus_sanfranciscensis [Firmicutes]:0.09542589146415360324,(bacFtsY@WP_056961480.1_Lactobacillus_florum [Firmicutes]:0.14270922100550553768,bacFtsY@WP_054646204.1_Lactobacillus_lindneri [Firmicutes]:0.06090046767037739228)87:0.07450792633965533374)100:0.20065296623760808803,bacFtsY@WP_009491663.1_Catellicoccus_marimammalium [Firmicutes]:0.18319963700944755236)49:0.03505178260150942221)100:0.17294486994344990261,(bacFtsY@WP_107510123.1_Staphylococcus_fleurettii [Firmicutes]:0.14859370835603516459,bacFtsY@AVK83142.1_Lysinibacillus_sp_B2A1 [Firmicutes]:0.15611705266078795828)84:0.08633486228927086947)76:0.08811389984970537104,(bacFtsY@WP_069327418.1_Paenibacillus_sp_TI45-13ar [Firmicutes]:0.16812180335501539230,(bacFtsY@WP_091834879.1_Marininema_halotolerans [Firmicutes]:0.17872043965696807200,bacFtsY@WP_028778316.1_Shimazuella_kribbensis [Firmicutes]:0.14839724987936192546)94:0.11183554952123123116)44:0.06312019674441764205)79:0.07162924527203394509,(bacFtsY@WP_026974763.1_Alicyclobacillus_contaminans [Firmicutes]:0.40779737368274887599,bacFtsY@PTQ57904.1_Candidatus_Carbobacillus_altaicus [Firmicutes]:0.35520900551231820996)73:0.10412161146297836678)65:0.04977836635061722720)15:0.03823392154775578228,(((bacFtsY@WP_081705397.1_Gloeobacter_kilaueensis [Cyanobacteria]:0.20919383977489405813,((bacFtsY@RCL55122.1_Synechococcus_sp_MED-G71 [Cyanobacteria]:0.11755062367536540935,(chrFtsY@AUG32399.1_Paulinella_longichromatophora [Rhizaria]:0.14995409299878054155,bacFtsY@WP_038650415.1_Prochlorococcus_sp_MIT_080 [Cyanobacteria]:0.22088613520821637204)98:0.10983139416108436692)100:0.25718456358704966380,(bacFtsY@WP_015110998.1_Nostoc_sp [Cyanobacteria]:0.10841202911667280340,bacFtsY@WP_111893930.1_Arthrospira_sp_O9_13F [Cyanobacteria]:0.08251981340498412698)98:0.14498169199356214509)59:0.08254502466498678248)100:0.41400682381119230380,bacFtsY@CDA51269.1_Clostridium_sp_CAG-138 [Firmicutes]:0.42306287865167607753)44:0.14615769784768337103,(bacFtsY@WP_054252106.1_Neofamilia_massiliensis [Firmicutes]:0.34322849760927581775,bacFtsY@WP_072972532.1_Tissierella_praeacuta [Firmicutes]:0.26535724273324673428)95:0.16059934404018910703)11:0.01514577603663419363)12:0.06037093270417581703,bacFtsY@KUO52399.1_Desulfitibacter_sp_BRH_c19 [Firmicutes]:0.38160036406290309685)6:0.05839559348570529801,bacFtsY@EEG77220.1_Dethiobacter_alkaliphilus_AHT_1 [Firmicutes]:0.27758432602545157764)5:0.03831490829622746092,(((bacFtsY@RCK76206.1_Anaerolineae_bacterium [Chloroflexi]:0.31254554864277894755,bacFtsY@KUK71287.1_Anaerolineae_bacterium_49_20 [Chloroflexi]:0.28876272887133180856)99:0.30172893963136676598,(bacFtsY@OQY47814.1_Anaerolineaceae_bacterium_4572_78 [Chloroflexi]:0.52190818148833262313,bacFtsY@WP_038038136.1_Thermorudis_peleae [Chloroflexi]:0.39870141113660589882)28:0.08860077965688523127)19:0.09917791449792706659,(bacFtsY@OLB22504.1_Nitrospirae_bacterium_13_2_20CM_2_63_8 [Nitrospirae]:0.51631177535753869368,bacFtsY@EKD41878.1_uncultured_bacterium [unassigned]:0.59032538534302070321)37:0.13333734410580289076)0:0.02473844672456647353)0:0.05542595739780690339,(bacFtsY@WP_009108227.1_Desulfovibrio_sp_U5L [Deltaproteobacteria]:0.56194778381643883947,(bacFtsY@AEG15826.1_Desulfofundulus_kuznetsovii_DSM_6115 [Firmicutes]:0.22741220436818790351,bacFtsY@WP_075860076.1_Carboxydothermus_pertinax [Firmicutes]:0.34183998833065454281)25:0.09174218304648419642)6:0.04426618045385726646)0:0.03589530485371571905)0:0.02528845202846503881)2:0.06185089096486070387)6:0.03898944393862244923)24:0.07274499072815526768,((((((((eukSRa@EKF29134.1_Trypanosoma_cruzi_marinkellei [Euglenozoa]:0.09299602525954617227,(eukSRa@EPY25999.1_Angomonas_deane

[Euglenozoa]:0.09172558904079242126,eukSRa@XP_010703301.1_Leishmania_panamensis [Euglenozoa]:0.09375266623392851384)65:0.04762770848149868591)100:0.48434448164819293714,((((eukSRa@XP_012894318.1_Blastocystis_hominis [Stramenopiles]:0.39426114236808135205,(((eukSRa@RLN51388.1_Phytophthora_kernoviae [Stramenopiles]:0.02084188953593871241,eukSRa@XP_002906671.1_Phytophthora_infestans_T30-4 [Stramenopiles]:0.02031773677313440662)99:0.05356982368442808617,eukSRa@GAX97406.1_Pythium_insidiosum [Stramenopiles]:0.10351189572280160289)100:0.17765873310298166410,(eukSRa@XP_005853460.1_Nannochloropsis_gaditana_CCMP526 [Stramenopiles]:0.22923829281003108016,eukSRa@CBJ30645.1_Ectocarpus_siliculosus [Stramenopiles]:0.29736070765883926281)94:0.10288885599604367937)97:0.12598004903553342015)63:0.08902721631093250820,((eukSRa@CAMPEP_0170537274_Litonotus_pictus_Strain_P1 [Alveolata]:0.42595792468529075636,eukSRa@XP_004029895.1_Ichthyophthirius_multifiliis [Alveolata]:0.44342042437049278281)83:0.11340238529118855382,((eukSRa@SBT01585.1_Plasmodium_malariae [Alveolata]:0.40986875821545909471,(eukSRa@GBE59094.1_Babesia_ovata [Alveolata]:0.49397831471640712975,(eukSRa@POM83525.1_Cryptosporidium_meleagridis [Alveolata]:0.01503372537306659970,eukSRa@XP_668286.1_Cryptosporidium_hominis [Alveolata]:0.01418766598348906310)100:0.38566264979904057997)41:0.05069487611675848465)54:0.06256140159961308400,(eukSRa@ESS33331.1_Toxoplasma_gondii [Alveolata]:0.01664929579964725612,eukSRa@CEL66130.1_Neospora_caninum [Alveolata]:0.04128564006831145217)100:0.27601645273177199957)81:0.11606037720902037669)55:0.07061698584650207000)25:0.03555558195160885004,((((eukSRa@GECH01003431.1_Pharyngomonas_kirbyi [Heterolobosea]:0.41157248825653341973,(eukSRa@NP_001171313.1_Homo_sapiens [Opisthokonta]:0.28721201351874142693,eukSRa@KOO32508.1_Chrysochromulina_sp_CCMP291 [Haptophyta]:0.37367266471808197048)22:0.08778797410969847725)3:0.04990085885502749952,(eukSRa@GFYU01005410.1_Ancoracysta_twista [Ancoracysta]:0.27022185877620802374,((eukSRa@CAMNT_0031806435_Goniomonas_pacifica [Cryptophyta]:0.14773569616506837532,eukSRa@GGUN01044729.1_Goniomonas_avonlea [Cryptophyta]:0.16394311973940423055)100:0.25480967862377917887,eukSRa@NA_Hemimastix_kukwesjijk [Hemimastigophora]:0.27798573007806626212)15:0.04519165433974599994)15:0.04681053050707519730)4:0.03046280352160890201,(eukSRa@GEZU01029986.1_GEZU01002276.1_Heterolobosea_BB2 [Heterolobosea]:0.20137811417271914149,(((eukSRa@XP_002670075.1_Naegleria_gruberi [Heterolobosea]:0.05136915072293381929,eukSRa@NF0122080_p1_Naegleria_fowleri [Heterolobosea]:0.05044206233537416179)100:0.27725373654931007028,((eukSRa@CAMNT_0005228041_Percolomonas_cosmopolitus_strain_WS [Heterolobosea]:0.37258947485451315851,eukSRa@CAMNT_0005204115_Percolomonas_cosmopolitus_AE [Heterolobosea]:0.50127013996633562609)76:0.16223877543516024291,eukSRa@NA_Neovahlkampfia_damariscottae [Heterolobosea]:0.20583245607049308057)42:0.06050198945678431778)49:0.07516602275356455698,(eukSRa@KYQ94416.1_Tieghemostelium_lacteum [Amoebozoa]:0.14532190497842109589,eukSRa@XP_012757500.1_Acytostelium_subglobosum_LB1 [Amoebozoa]:0.12176511541659526539)100:0.24089892135207616186)21:0.06414654627111153473)6:0.04354349704746730138)2:0.02312307977064168019,(eukSRa@MMETSP0308_Transcript_27849_m29213_Gloeochaete_wittrockiana [Archaeplastida]:0.21235521724078360029,((eukSRa@OUS44851.1_Ostreococcus_tauri [Archaeplastida]:0.60851279708902805687,(eukSRa@XP_005848975.1_Chlorella_variabilis [Archaeplastida]:0.13834593576113191071,(eukSRa@XP_001692081.1_Chlamydomonas_reinhardtii [Archaeplastida]:0.02867056796855534107,(eukSRa@KXZ55716.1_Gonium_pectorale [Archaeplastida]:0.02893827685451482237,eukSRa@XP_002949444.1_Volvox_carteri_nagariensis [Archaeplastida]:0.02928394537538089176)48:0.01897709693547283694)100:0.23160290927887836054)92:0.05742318091163960136)52:0.03932675159504057244,((eukSRa@GBG74478.1_Chara_braunii [Archaeplastida]:0.07673798585181189347,(((eukSRa@XP_020873914.1_Arabidopsis_lyrata_subsp_lyrata [Archaeplastida]:0.05634857988770818754,((eukSRa@XP_021634617.1_Manihot_esculenta [Archaeplastida]:0.01297381922106438483,(eukSRa@XP_021970653.1_Helianthus_annuus [Archaeplastida]:0.05910965685740722508,eukSRa@XP_022750268.1_Durio_zibethinus [Archaeplastida]:0.01407101293709921326)27:0.00448762125997803109)30:0.00656725056615349839,eukSRa@XP_003527179.1_Glycine_max [Archaeplastida]:0.03347743966136553645)38:0.00413404239948628790)73:0.02643652952705826883,eukSRa@ONL92607.1_Zea_mays [Archaeplastida]:0.06886433160186154756)84:0.03733804032864350420,eukSRa@XP_024403926.1_Physcomitrella_patens [Archaeplastida]:0.16197868948921670396)69:0.03870611919893053587)83:0.05183026473155807889,eukSRa@CAMPEP_0191492328_Pyramimonas-parkeae-CCMP726 [Archaeplastida]:0.28940851797650768429)39:0.03140613981310237668)83:0.10546391809573364695)38:0.06284875236218327721)3:0.03556519676808456154)4:0.04240387617653667424,eukSRa@ANM86232.1_Stygiella_incarcerata [Jakobida]:0.38850202801947891773)11:0.05885314184142799443)23:0.18498853181200175522,eukSRa@AAD11975.1_Giardia_intestinalis [Metamonada]:0.97571064092698622794)100:0.54803872556384070069,(archFtsY@OIO41360.1_Candidatus_Pacearchaeota_archaeon_CG1_02_31_27 [DPANN]:0.35147441110888327787,archFtsY@PIZ51778.1_Candidatus_Woesearchaeota_archaeon_CG_4_10_14_0_2_um_filter_33_13 [DPANN]:0.45655916925368644765)100:0.26015601943021171572)43:0.07852762617211615781,(archFtsY@WP_048122758.1_Methanosarcina_vacuolata [Euryarchaeota]:0.31651765434489570472,(archFtsY@WP_006182963.1_Natrinema_pellirubrum [Euryarchaeota]:0.16089279407930334731,(archFtsY@WP_004045232.1_Haloferax_volcanii [Euryarchaeota]:0.10203084746895703383,((archFtsY@WP_021073086.1_Haloarchaeon_3A1_DGR [Euryarchaeota]:0.04401273756436686890,archFtsY@WP_050034148.1_Halorubrum_halophilum [Euryarchaeota]:0.09179044040620275291)85:0.08872815451221903460,archFtsY@WP_114604860.1_Haloplanus_sp._CBA1112 [Euryarchaeota]:0.14501386852046457832)49:0.06474724940356484104)50:0.07710479800648348259)100:0.43545488380221075708)99:0.21569146788122653402)20:0.02014530566659287689,(archFtsY@RMF91339.1_Euryarchaeota_archaeon [Euryarchaeota]:0.46932309090042717559,archFtsY@RLI30149.1_Candidatus_Bathyarchaeota_archaeon [TACK]:0.40890081447513837443)31:0.08721835601676580785)11:0.04917807458063272463,(archFtsY@RLI57948.1_Candidatus_Thorarchaeota_archaeon [Asgard]:0.43014404046435128492,archFtsY@OYT54953.1_Candidatus_Altiarchaeales_archaeon_ex4484_2 [DPANN]:0.41802390401539141207)21:0.10989977819276153248)17:0.04725474795562389918,(archFtsY@WP_014122151.1_Thermococcus_sp._AM4 [Euryarchaeota]:0.12109428377467866544,archFtsY@WP_011012907.1_Pyrococcus_furiosus [Euryarchaeota]:0.08681045716815478930)100:0.25084184100743872614)100:0.88977649929717017585)97:0.11685171900582451421,(ptFtsY@MMETSP0308_Transcript_18196_m19230_Gloeochaete_wittrockiana [Archaeplastida]:0.47474315727414151400,(((((ptFtsY@XP_001697752.1_chloroplast_SRP_receptor_Chlamydomonas_reinhardtii:0.41693940019799430319,ptFtsY@XP_005651149.1_cell_division_transporter_substrate-binding_protein_FtsY_Coccomyxa_subellipsoidea_C-169:0.21278179613998093878)54:0.07631652792165066757,ptFtsY@PRW61060.1_cell_division_chloroplastic_isoform_A_Chlorella_sorokiniana:0.15016461530635788479)60:0.07103263354081019998,ptFtsY@XP_011399720.1_Signal_recognition_particle_receptor_FtsY_Auxenochlorella_protothecoides:0.35928732499940796430)63:0.08861293063647421309,ptFtsY@XP_003080532.1_Signal-recognition_particle_receptor_FtsY_Ostreococcus_tauri:0.43544652977661701954)55:0.06811318269524556379,(ptFtsY@NP_566056.1_Arabidopsis_thaliana [Archaeplastida]:0.06878267847562155590,(ptFtsY@XP_010055217.1_Eucalyptus_grandis [Archaeplastida]:0.06042012986964622606,((ptFtsY@XP_023879170.1_Quercus_suber [Archaeplastida]:0.02887089915730963255,(ptFtsY@XP_022757233.1_Durio_zibethinus [Archaeplastida]:0.03442660581774370510,ptFtsY@KHF98418.1_Cell_division_FtsY_chloroplastic-like_protein_Gossypium_arboreum [Archaeplastida]:0.03140871998249148561)82:0.01676644148566954970)28:0.00511376555387653940,ptFtsY@XP_021817084.1_Prunus_avium [Archaeplastida]:0.05595762204731181272)23:0.01766698109250820423)35:0.02838547372882177511)100:0.28424814423400812657)100:0.22070857534507679065)65:0.08333663764161480803)96:0.19116721908797307261,ptFtsY@XP_005537370.1_Cyanidioschyzon_merolae_strain_10D [Archaeplastida]:0.59050232980913797132);

**Phylogenetic tree from Fig. S4 (RAxML, LG4X model, 500 rapid bootstraps, 154 OTUs)**

((bacFtsY@OWQ45088.1_Mitsuaria_noduli [Betaproteobacteria]:0.05708639624182364897,((bacFtsY@WP_089417744.1_Vitreoscilla_filiformis [Betaproteobacteria]:0.13093011116778680014,(bacFtsY@WP_056203513.1_Pelomonas_sp_Root1237 [Betaproteobacteria]:0.08163021216033708449,bacFtsY@PZP35629.1_Roseateles_depolymerans [Betaproteobacteria]:0.04748418772864165804)45:0.02322360584192610308)34:0.02860787970909261721,bacFtsY@WP_066336977.1_Azohydromonas_lata [Betaproteobacteria]:0.10107016103840309185)36:0.03564663282367405706)26:0.04029163845925950310,(bacFtsY@OYZ20489.1_Bdellovibrio_sp_28-41-41 [Deltaproteobacteria]:0.76952488656783701870,((((((((bacFtsY@PIE60181.1_Desulfobulbus_propionicus [Deltaproteobacteria]:0.35511987043114950913,(bacFtsY@ABC77884.1_Syntrophus_aciditrophicus_SB [Deltaproteobacteria]:0.26989804641944203922,bacFtsY@PIP06279.1_Syntrophobacteraceae_bacterium_CG23_combo_of_CG06-09_8_20_14_all_50_8 [Deltaproteobacteria]:0.19266758103665254653)100:0.24522875151897954860)15:0.06887638403993553982,(bacFtsY@WP_083764053.1_Syntrophobacter_fumaroxidans [Deltaproteobacteria]:0.35472575957403312819,bacFtsY@PIP45503.1_Deltaproteobacteria_bacterium_CG23_combo_of_CG06-09_8_20_14_all_51_20 [Deltaproteobacteria]:0.32637068839195432224)30:0.06584933341520447792)20:0.04210510341774632848,(bacFtsY@WP_011985037.1_Anaeromyxobacter_sp_Fw109-5 [Deltaproteobacteria]:0.37781220386240332854,(bacFtsY@PID38167.1_Proteobacteria_bacterium [Proterobacteria]:0.45763478398149320947,bacFtsY@KPK53060.1_Myxococcales_bacterium_SG8_38_1 [Deltaproteobacteria]:0.56699576076870994434)36:0.10795790980359826206)31:0.12253004758838619004)6:0.08022769299708605584,(((((bacFtsY@WP_116686709.1_contaminant of Flavobacteriaceae_bacterium Hp12 genome [Gammaproteobacteria]:0.23630517519335830778,(((bacFtsY@WP_091826228.1_Marinobacterium_georgiense [Gammaproteobacteria]:0.12804338440049237158,(bacFtsY@KXS51830.1_Marinobacter_sp_T13-3 [Gammaproteobacteria]:0.03305609765446096415,bacFtsY@ARM82543.1_Marinobacter_salarius [Gammaproteobacteria]:0.03746036232221933854)98:0.11982078659146662425)88:0.07806332323104714366,(bacFtsY@WP_097790462.1_Halomonas_beimenensis [Verrucomicrobia]:0.11360423159144279748,(bacFtsY@KGA01103.1_Cobetia_amphilecti [Gammaproteobacteria]:0.00000100000050002909,bacFtsY@WP_107335894.1_Halomonas_sp_SF2003 [Verrucomicrobia]:0.00000100000050002909)100:0.09042280918803244361)98:0.11373363279255500891)43:0.04831270782521430301,(bacFtsY@WP_113743312.1_Anaerobiospirillum_thomasii [Gammaproteobacteria]:0.37840016934597037190,((bacFtsY@WP_091986549.1_Pseudoalteromonas_denitrificans [Gammaproteobacteria]:0.16040045595793100963,bacFtsY@WP_094041167.1_Zobellella_denitrificans [Gammaproteobacteria]:0.09930556132116939716)92:0.05264927393674926487,bacFtsY@PPI88525.1_Pantoea_sp_SoEO [Gammaproteobacteria]:0.50195019599367274310)55:0.03463792042219310946)44:0.06316071324775192342)25:0.03653661355246477782)38:0.05974694049258539447,bacFtsY@WP_086487265.1_Thioflexothrix_psekupsii [Gammaproteobacteria]:0.22936458053095426513)44:0.04359822500960410657,(bacFtsY@WP_095208401.1_Luteimonas_sp_JM171 [Gammaproteobacteria]:0.38760591623874846023,bacFtsY@OUX68934.1_Oceanospirillales_bacterium_TMED91 [Gammaproteobacteria]:0.45690463283039040032)39:0.10806859642902602592)62:0.07751608544866121775,(((bacFtsY@WP_025048799.1_Sulfitobacter_mediterraneus [Alphaproteobacteria]:0.07951836059521343036,bacFtsY@KRS17267.1_Roseovarius_indicus [Alphaproteobacteria]:0.08757748414409693982)100:0.34444501244736941414,(bacFtsY@GBD43248.1_Bacterium_HR40 [unassigned]:0.46246053900979333662,((bacFtsY@EPY01636.1_Phaeospirillum_fulvum_MGU-K5 [Alphaproteobacteria]:0.18544878837367312352,(bacFtsY@OFX10022.1_Alphaproteobacteria_bacterium_RIFOXYD12_FULL_60_8 [Alphaproteobacteria]:0.15429603664632662863,bacFtsY@WP_092615803.1_Roseospirillum_parvum [Alphaproteobacteria]:0.27994089471735944530)50:0.05564442742093191063)65:0.09644565731827738075,(bacFtsY@AIL12880.1_Candidatus_Paracaedimonas_acanthamoebae [Alphaproteobacteria]:0.45368253847863831973,bacFtsY@OUU28495.1_Candidatus_Endolissoclinum_sp_TMED37 [Alphaproteobacteria]:0.33662226389555777173)51:0.11065015638867820025)30:0.05601203368155282386)7:0.01266392150709278602)53:0.03552079758686484123,((bacFtsY@WP_083773102.1_Hirschia_baltica [Alphaproteobacteria]:0.00000100000050002909,bacFtsY@ACT57804.1_Hirschia_baltica_ATCC_49814 [Alphaproteobacteria]:0.00000100000050002909)100:0.46613014046606393581,((bacFtsY@WP_029041189.1_Cucumibacter_marinus [Alphaproteobacteria]:0.22642562136614083346,(bacFtsY@WP_099557887.1_Hartmannibacter_diazotrophicus [Alphaproteobacteria]:0.14321204760837757952,((bacFtsY@WP_108880431.1_Anderseniella_sp_Alg231-50 [Alphaproteobacteria]:0.24647639660134540440,bacFtsY@WP_109793879.1_Rhizobiales_bacterium [Alphaproteobacteria]:0.28816367380127350106)66:0.08966527085283422516,(bacFtsY@WP_088520031.1_Rhodoblastus_acidophilus [Alphaproteobacteria]:0.17096764480922679863,(bacFtsY@WP_115516251.1_Pseudolabrys_sp_GY_H [Alphaproteobacteria]:0.28865957619668070055,(bacFtsY@SKC16052.1_Bosea_thiooxidans [Alphaproteobacteria]:0.19961628813095566581,(bacFtsY@WP_013168132.1_Starkeya_novella [Alphaproteobacteria]:0.14415254646594702348,(bacFtsY@WP_024277252.1_Xanthobacter_sp_126 [Alphaproteobacteria]:0.04699074777149300081,bacFtsY@WP_011996071.1_Xanthobacter_autotrophicus [Alphaproteobacteria]:0.06768085177765212346)100:0.12981217964863375758)88:0.06902264737470251155)64:0.04376691878510823031)57:0.03354036260261110403)61:0.04967202886355905761)30:0.03642036664374374150)27:0.03362267791668048511)32:0.03135932474980927115,(bacFtsY@PPD07903.1_Hyphomicrobium_sp [Alphaproteobacteria]:0.24149079527978314919,(bacFtsY@WP_113333951.1_Rhizobiales_bacterium [Alphaproteobacteria]:0.20227666405808467087,bacFtsY@RCL01761.1_Candidatus_Tokpelaia_sp_JSC085 [Alphaproteobacteria]:0.31436837838837833337)77:0.11114966818098157009)24:0.02025716535710788552)38:0.08633166229284126858)54:0.05282342686100503781)85:0.37842972493062454475)41:0.08099750440745086266,((bacFtsY@PIE91415.1_Acidobacteria_bacterium [Acidobacteria]:0.50574280045029562647,(bacFtsY@PYS67814.1_Acidobacteria_bacterium [Acidobacteria]:0.35694540407492536538,(((bacFtsY@WP_121469822.1_Edaphobacter_dinghuensis [Acidobacteria]:0.20386260488237856192,(bacFtsY@WP_081490725.1_Terriglobus_roseus [Acidobacteria]:0.00956131658573000642,bacFtsY@WP_074656074.1_Terriglobus_roseus [Acidobacteria]:0.01788549173100237658)99:0.17561157673975433258)99:0.33231637311316963856,(bacFtsY@WP_020721417.1_Acidobacteriaceae_bacterium_KBS_96 [Acidobacteria]:0.17582693483252725963,bacFtsY@WP_031499580.1_Bryobacter_aggregatus [Acidobacteria]:0.19773392742657208698)90:0.10850937198424161212)49:0.07221292193595539588,bacFtsY@ANM28936.1_Acidobacteria_bacterium_Mor1 [Acidobacteria]:0.65121757458197837565)33:0.07302809148497325931)25:0.06530687950229573102)35:0.08709122807962001167,((mtFtsY@NA_N-terminus_Ancoracysta_twista [Ancoracysta]:0.80812734623425286351,(mtFtsY@QUTJ01025227.1_Goniomonas_avonlea [Cryptophyta]:0.77183656827991609095,(mtFtsY@NA_Ancoracysta-related_Colp-4b [Ancoracysta]:0.84532302543055692912,mtFtsY@NA_partial_Goniomonas_pacifica [Cryptophyta]:1.27849656175284520643)15:0.08144522910802659554)34:0.05615132880681400485)20:0.16035758153416154737,(mtFtsY@NA_1_Hemimastix_kukwesjijk [Hemimastigophora]:0.69267300515711127673,(((mtFtsY@CAMNT_0005246107_Percolomonas_cosmopolitus_WS [Heterolobosea]:0.31991681713993613823,mtFtsY@NA_partial_Percolomonas_ex_Nitzchia_Cheng_2013 [Heterolobosea]:0.56723458584296049256)86:0.38133418203334751295,((mtFtsY@NA_Naegleria_gruberi [Heterolobosea]:0.00015569548488128819,(mtFtsY@NA_Naegleria_lovaniensis [Heterolobosea]:0.01278768308521138243,mtFtsY@NA_Naegleria_fowleri [Heterolobosea]:0.01291361106768191581)99:0.08448750198634523423)100:0.49739553827335319935,(mtFtsY@NA_Neovahlkampfia_damariscottae [Heterolobosea]:0.45760039471519048382,mtFtsY@CAMNT_0005204377-extended_Percolomonas_cosmopolitus_AE [Heterolobosea]:1.54034978654773135887)51:0.15926646606487307456)50:0.12698785236133094956)34:0.09461682408667095034,(mtFtsY@NA_Heterolobosea_BB2 [Heterolobosea]:0.21009699475730839180,mtFtsY@NA_Pharyngomonas_kirbyi [Heterolobosea]:0.42677779004138338692)69:0.15772582671400708909)33:0.15851719976583092397)10:0.09791673390782282271)21:0.23870368840613526151)2:0.04076745645543795871)0:0.04431425470755687157)0:0.06312556343794618885,((bacFtsY@WP_012175138.1_Desulfococcus_oleovorans [Deltaproteobacteria]:0.42168574227722355197,(bacFtsY@EKD41878.1_uncultured_bacterium [unassigned]:0.70411354132751413637,bacFtsY@OLB22504.1_Nitrospirae_bacterium_13_2_20CM_2_63_8 [Nitrospirae]:0.48171793097299625552)39:0.14263680363203368184)3:0.08922657155044269595,(((bacFtsY@PSQ63899.1_Bacteroidetes_bacterium_QH_1_61_8 [Bacteroidetes]:0.34855193896795932940,((bacFtsY@OUV32974.1_Rhodothermaceae_bacterium_TMED105 [Bacteroidetes]:0.40519397068506740611,(bacFtsY@WP_100314957.1_Thermoflavifilum_aggregans [Bacteroidetes]:0.26482711839868128267,bacFtsY@OUU18192.1_Crocinitomicaceae_bacterium_TMED45 [Bacteroidetes]:0.36091926530179935240)54:0.05563659189822461187)37:0.06084389394107479754,((bacFtsY@PSR05731.1_Bacteroidetes_bacterium_SW_10_40_5 [Bacteroidetes]:0.33005527292469150069,bacFtsY@OUV76101.1_Flavobacteriales_bacterium_TMED123 [Bacteroidetes]:0.22887560301969039900)77:0.06912983272609038443,(bacFtsY@WP_103327589.1_Bacteroidetes_endosymbiont_of_Geopemphigus_sp [Bacteroidetes]:0.20968882121671009178,bacFtsY@WP_114910387.1_Cardinium_endosymbiont_of_Sogatella_furcifera [Bacteroidetes]:0.45191146370176871194)62:0.10076280337468943027)45:0.03710281476454517502)46:0.07612984385241670926)86:0.09202165291458334895,((((bacFtsY@OQY74580.1_Ignavibacteriales_bacterium_UTCHB3 [Ignavibacteriae]:0.32892404417497628222,(bacFtsY@PKL82841.1_Ignavibacteriae_bacterium_HGW-Ignavibacteriae-3 [Ignavibacteriae]:0.48470231171274147375,bacFtsY@OGU83728.1_Ignavibacteria_bacterium_RBG_16_35_7 [Ignavibacteriae]:0.47406513245488951513)86:0.12752580873846225407)96:0.28606309489531150936,bacFtsY@WP_092350764.1_Candidatus_Chrysopegis_kryptomonas [Kryptonia]:0.19632666334413786946)55:0.08485161859284966190,(((bacFtsY@PKL79980.1_Ignavibacteriae_bacterium_HGW-Ignavibacteriae-4 [Ignavibacteriae]:0.29512912126081719544,bacFtsY@OJX59420.1_Candidatus_Kapabacteria_thiocyanatum [Bacteroidetes]:0.16548057621585202104)90:0.12898474011053678856,bacFtsY@KXK57805.1_Chlorobi_bacterium_OLB7 [Chlorobi]:0.22417893516171319424)79:0.10743141079274509675,bacFtsY@PLX30570.1_Ignavibacteria_bacterium [Ignavibacteriae]:0.19795169310847393618)46:0.03286542631796068176)43:0.05776983591738142632,bacFtsY@OGU26318.1_Ignavibacteria_bacterium_GWA2_54_16 [Ignavibacteriae]:0.43483267718525769885)26:0.02994905263490432734)68:0.12594795286614318686,bacFtsY@OGC84905.1_Zixibacteria_bacterium_RBG_16_43_9 [Zixibacteria]:0.41859567126203900411)17:0.05883263900694953724)0:0.00760855336525522504)0:0.04124215302684883233,(((bacFtsY@WP_009108227.1_Desulfovibrio_sp_U5L [Deltaproteobacteria]:0.58451226039125048217,((bacFtsY@RCK76206.1_Anaerolineae_bacterium [Chloroflexi]:0.37703092841285090753,bacFtsY@KUK71287.1_Anaerolineae_bacterium_49_20 [Chloroflexi]:0.29909107205398410834)99:0.29035651958664931982,(bacFtsY@OQY47814.1_Anaerolineaceae_bacterium_4572_78 [Chloroflexi]:0.52914861616450059589,bacFtsY@WP_038038136.1_Thermorudis_peleae [Chloroflexi]:0.43908615823831226299)34:0.07734878351865059898)39:0.11506218262870736291)5:0.06831860158332334065,((bacFtsY@AEG15826.1_Desulfofundulus_kuznetsovii_DSM_6115 [Firmicutes]:0.24997639314745093153,bacFtsY@WP_075860076.1_Carboxydothermus_pertinax [Firmicutes]:0.33461144523555857999)36:0.12905204418467391969,(((((((bacFtsY@WP_103423367.1_Lactobacillus_sanfranciscensis [Firmicutes]:0.12996570575681704041,(bacFtsY@WP_056961480.1_Lactobacillus_florum [Firmicutes]:0.14855972580143600448,bacFtsY@WP_054646204.1_Lactobacillus_lindneri [Firmicutes]:0.05670740783799629176)96:0.08416421925951496352)100:0.19134489234987495099,(bacFtsY@WP_009554695.1_Lactobacillus_saerimneri [Firmicutes]:0.23792005069622229607,bacFtsY@WP_009491663.1_Catellicoccus_marimammalium [Firmicutes]:0.19213653775515882050)36:0.02754288086963799578)100:0.17615202048377137656,(bacFtsY@WP_107510123.1_Staphylococcus_fleurettii [Firmicutes]:0.15769000815073014321,bacFtsY@AVK83142.1_Lysinibacillus_sp_B2A1 [Firmicutes]:0.16378101095520675168)86:0.08998994306287748379)85:0.09309944512740507550,((bacFtsY@WP_028778316.1_Shimazuella_kribbensis [Firmicutes]:0.15942479747870469398,bacFtsY@WP_091834879.1_Marininema_halotolerans [Firmicutes]:0.18103314813535464900)96:0.11865390475287845851,bacFtsY@WP_069327418.1_Paenibacillus_sp_TI45-13ar [Firmicutes]:0.19325331592167541150)38:0.04588814640558377261)72:0.06922855494801122278,(bacFtsY@WP_026974763.1_Alicyclobacillus_contaminans [Firmicutes]:0.44505409032468284947,bacFtsY@PTQ57904.1_Candidatus_Carbobacillus_altaicus [Firmicutes]:0.36907397008806602789)67:0.11649995021602049805)76:0.10707372450985046253,(bacFtsY@KUO52399.1_Desulfitibacter_sp_BRH_c19 [Firmicutes]:0.43308401490076381402,((bacFtsY@SME87908.1_Pseudobacteriovorax_antillogorgiicola [Deltaproteobacteria]:0.62147014905587305567,((bacFtsY@WP_081705397.1_Gloeobacter_kilaueensis [Cyanobacteria]:0.20227744012202605872,(((chrFtsY@AUG32399.1_Paulinella_longichromatophora [Rhizaria]:0.15676989497056051381,bacFtsY@WP_038650415.1_Prochlorococcus_sp_MIT_080 [Cyanobacteria]:0.22052907358239828839)98:0.09258813065370327666,bacFtsY@RCL55122.1_Synechococcus_sp_MED-G71 [Cyanobacteria]:0.11020549896323837691)99:0.27913904705307851506,(bacFtsY@WP_015110998.1_Nostoc_sp [Cyanobacteria]:0.12773874103920956902,bacFtsY@WP_111893930.1_Arthrospira_sp_O9_13F [Cyanobacteria]:0.07319063642045618712)98:0.15256064892256387955)37:0.07984808369042246945)100:0.51133724753591358603,bacFtsY@CDA51269.1_Clostridium_sp_CAG-138 [Firmicutes]:0.43587888024197907200)35:0.08912059666562910376)6:0.08392719034250857579,(bacFtsY@WP_054252106.1_Neofamilia_massiliensis [Firmicutes]:0.37909849328795874879,bacFtsY@WP_072972532.1_Tissierella_praeacuta [Firmicutes]:0.26868318718427713909)98:0.18947395640870623446)3:0.00000100000050002909)1:0.03943094142226393622)1:0.06056147922769847952,(bacFtsY@WP_073092242.1_Thermosyntropha_lipolytica [Firmicutes]:0.38386628486997931287,bacFtsY@EEG77220.1_Dethiobacter_alkaliphilus_AHT_1 [Firmicutes]:0.31029682523780116599)6:0.03451463268267868784)1:0.05673060205924698729)0:0.00330067225670574492)0:0.04977305037576921359,bacFtsY@OGP30931.1_Deltaproteobacteria_bacterium_GWC2_42_11 [Deltaproteobacteria]:0.50801197666323105739)0:0.03019471938391331614)2:0.07579912287763608536,((bacFtsY@WP_041017695.1_Criblamydia_sequanensis [Chlamydiae]:0.51840721040137494047,bacFtsY@PCI95412.1_Candidatus_Aerophobetes_bacterium [Aerophobetes]:0.47111802859100382346)85:0.21157535491548967732,(bacFtsY@WP_088252003.1_Fimbriiglobus_ruber [Planctomycetes]:0.40295206488868884342,(bacFtsY@RMH26650.1_Planctomycetes_bacterium [Planctomycetes]:0.31741547807957043448,bacFtsY@RPG16414.1_FtsY_Phycisphaera_sp_TMED9 [Planctomycetes]:0.43891782014087959984)98:0.22723819724756402771)68:0.11404025665574360482)23:0.08683391417700907222)6:0.03724983081780555011,(((ptFtsY@XP_005537370.1_Cyanidioschyzon_merolae_strain_10D [Archaeplastida]:0.59901425687155718247,((ptFtsY@CEM32712.1_Vitrella_brassicaformis_CCMP3155 [Alveolata]:0.38929474061957958231,((ptFtsY@XP_002296627.1_Thalassiosira_pseudonana_CCMP1335 [Stramenopiles]:0.60559892235355117229,(ptFtsY@EWM29383.1_Nannochloropsis_gaditana [Stramenopiles]:0.28830762628018880012,ptFtsY@CBJ31918.1_Ectocarpus_siliculosus [Stramenopiles]:0.39351702105322039493)71:0.08953076738015333658)39:0.05804162814563721545,ptFtsY@XP_009040860.1_Aureococcus_anophagefferens [Stramenopiles]:0.37643868827678239608)29:0.04627357298808008196)93:0.14624560840455905208,(ptFtsY@XP_005714040.1_Chondrus_crispus [Archaeplastida]:0.13887994776891218751,ptFtsY@PXF44704.1_Gracilariopsis_chorda [Archaeplastida]:0.11603271390800515284)100:0.27859762466902604494)53:0.08947385499658838670)43:0.07138517091474254339,ptFtsY@XP_005705405.1_Galdieria_sulphuraria [Archaeplastida]:0.86143672714851093897)98:0.18852826960128607481,(ptFtsY@MMETSP0308_Transcript_18196_m19230_Gloeochaete_wittrockiana [Archaeplastida]:0.42137126857549833669,((ptFtsY@NP_566056.1_Arabidopsis_thaliana [Archaeplastida]:0.058499

8906248857567,(ptFtsY@XP_010055217.1_Eucalyptus_grandis [Archaeplastida]:0.06085219544283056170,(ptFtsY@XP_023879170.1_Quercus_suber [Archaeplastida]:0.02211527050819737245,((ptFtsY@KHF98418.1_Cell_division_FtsY_chloroplastic-like_protein_Gossypium_arboreum [Archaeplastida]:0.02431443142612418548,ptFtsY@XP_022757233.1_Durio_zibethinus [Archaeplastida]:0.02588460288511316293)85:0.02043506307922911269,ptFtsY@XP_021817084.1_Prunus_avium [Archaeplastida]:0.06330118520103825142)34:0.01043664912838357210)52:0.01987905358739553222)32:0.01778918395277858180)100:0.29633447234594972874,((ptFtsY@XP_011399720.1_Signal_recognition_particle_receptor_FtsY_Auxenochlorella_protothecoides:0.34516094780178213641,(ptFtsY@PRW61060.1_cell_division_chloroplastic_isoform_A_Chlorella_sorokiniana:0.16182254302938040014,(ptFtsY@XP_005651149.1_cell_division_transporter_substrate-binding_protein_FtsY_Coccomyxa_subellipsoidea_C-169:0.23979627901433012149,ptFtsY@XP_001697752.1_chloroplast_SRP_receptor_Chlamydomonas_reinhardtii:0.40434889094723619474)55:0.07403136225511368351)65:0.05893502995014886919)68:0.07450251308910496584,ptFtsY@XP_003080532.1_Signal-recognition_particle_receptor_FtsY_Ostreococcus_tauri:0.48240110576511296969)75:0.07852415021933305261)100:0.22691623855105788610)80:0.07337277267935518599)97:0.26198134594474342673)29:0.08695497499186390089)99:0.49366972842146933376,bacFtsY@WP_124447410.1_Paucibacter_sp_KBW04 [Betaproteobacteria]:0.03115074568890529097);
