# Supplementary material for: Vestiges of the Bacterial Signal Recognition Particle-Based Protein Targeting in Mitochondria
Source: Mol Biol Evol. 2021 Apr 10;38(8):3170–87. doi: 10.1093/molbev/msab090 (PMC8321541; doi:10.1093/molbev/msab090)
Supplement: msab090_Supplementary_Data [file msab090_supplementary_data.zip › Supplementary_figures.pdf]

(A)

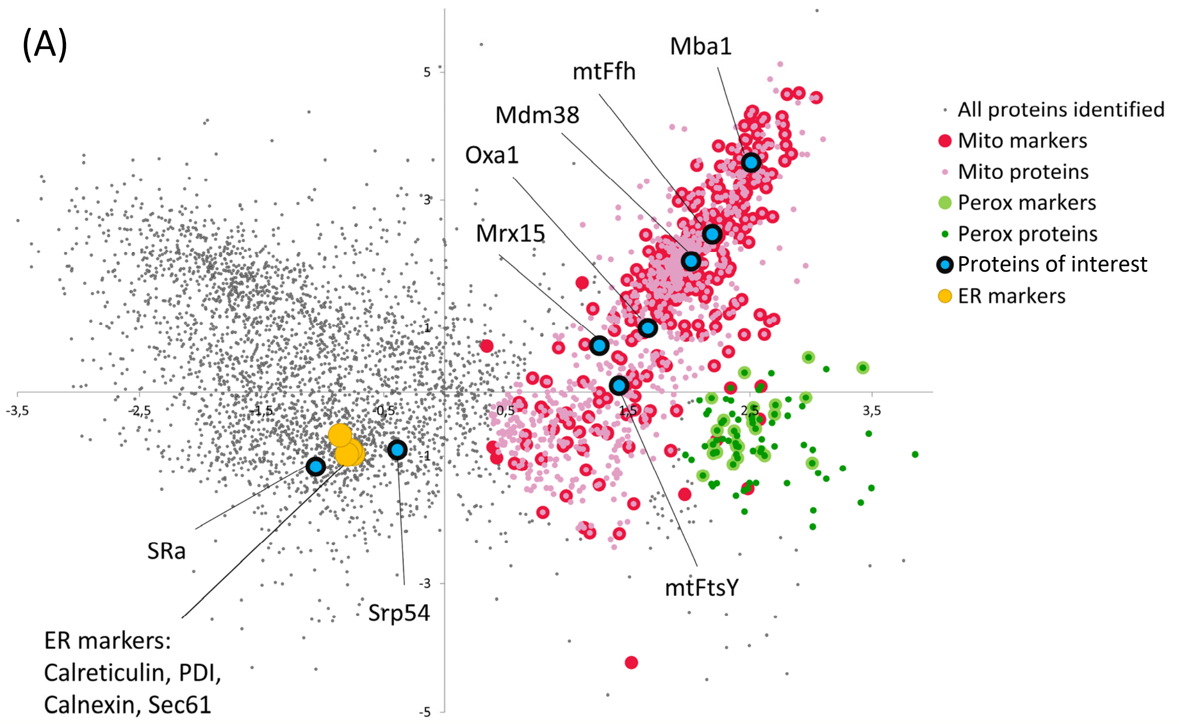

(B)

mtFfh

```
                                                                                                            EDVEEAMQK
MFKSNSRSLLSRFLKTANTHHHCKIVFSSSEVFSPALCHHTSLFSSNNKNVGLQHVLQQYNQQQVRFFHSSPKQYGLFSNLTSNMSDAFTNLFKKKTLTKEDVEEAMQK

  VALLDADVAESVVSQFVK                                                                                 SVIMVTGIQGSGK
                    EATEDAIGIAAVK                                                    LSELLGGGVAPLELIPSTEGK
VRVALLDADVAESVVSQFVKEATEDAIGIAAVKQVESQENKGILSRIAKWVGPNQKAEELPKSATVYLMVMDRLSELLGGGVAPLELIPSTEGKSVIMVTGIQGSGKTT

                                                                 AMEYVGPNGEQFDTVIFDTAGR                     AIVQPNETLLVAD
                                                                                       MHIDEQLMTELEELR
SSAKLALQLKRKENRRVLLVSLDTYRPAAQMQLQTLAQQIQVESLPIIPEQNPIEIAKRAMEYVGPNGEQFDTVIFDTAGRMHIDEQLMTELEELRAIVQPNETLLVAD

SMLGNDAVNIATQFHDR                                             MDDLETFDPQSLAK             AKDAMNLDAAQAQQK       GAYT
                                                                            RILGGGDIMTLAQK
SMLGNDAVNIATQFHDRVGLSGIVLTRMDGTSSGGCAISMKQVVGLSVKYIGIGERMDDLETFDPQSLAKRILGGGDIMTLAQKAKDAMNLDAAQAQQKLVQFSKGAYT

FKDYQDQIQMLK                                                                                  MLKMYEK
  DYQDQIQMLK                    DKLDNMDLSFIDNHEHIISCMSEQEK                          AKVEVTDVNK
FKDYQDQIQMLKKMGSLKNMASYLPEQFIGKFRDKLDNMDLSFIDNHEHIISCMSEQEKLQPLLVESSSARRLDLAKRAKVEVTDVNKMLKMYEKMKSFISKAGSAAMK

DPKKMSEQMMKDPMFFANMFPMKVKKQLIRPPKR
```

mtFtsY

```
                                 KIFNEEDFKLLEK                                       QIENNPTAEENVKPMK                     RL
                                              ALLSSDVGNNTTQLLLTR                     QIENNPTAEENVK                         L
MFNKILGGLSKTSIFSKIGSILGGGATSRKIFNEEDFKLLEKALLSSDVGNNTTQLLLTRMKTQVSEIEKQIENNPTAEENVKPMKNILREEMLKLFQYPMQQQIVKRL

QQNQGTPEGGVSIPLIPLNPK
QQNQGTPEGGVSIPLIPLNPK                                                                            TSGCTLVDLTESEK
QQNQGTPEGGVSIPLIPLNPKSRPTVVQICGVNGSGKTTTIGKLLHKYRESGTVRNIVVAAADTVRAAAPDQLRTWVERTSGCTLVDLTESEKLKKQHTTTPQQVKVKN

       VPAESVVYEAIQQALRKEDVDLVFVDTAGR
       VPAESVVYEAIQQALR
                       KEDVDLVFVDTAGR         ELSLITEMCSK
IRQVSYKVPAESVVYEAIQQALRKEDVDLVFVDTAGRLQNQEASMKELSLITEMCSKSRKGAPDHTWLILDGTIGQNSIQQAKLFQKYVRISGIIVTKLDGSAKGGVIL

AIANELKIPVLYIGLGESVSDLKPFYPEQFVDSILSVASAEEKQTKSEEDDE
```

Supplementary fig. S1. Proteomic identification of mtFfh, mtFtsY, and other mitochondrial proteins of interest in cell fractions of *N. gruberi*. (**A**) PCA analysis of 4,198 proteins proteomically measured in cell fractions differentially enriched in mitochondria. The cluster of mitochondrial proteins was defined based on 376 mitochondrial markers. The boundaries of the cluster of co-purified peroxisomal proteins were established by 26 peroxisomal markers, the position of four selected endoplasmic reticulum (ER) markers is also shown. All *N. gruberi* proteins specifically discussed in the main text as supposedly mitochondrial, including mtFfh and mtFtsY, clearly co-purify with the mitochondrial markers, in contrast to the components of the cytoplasmic/ER SRP system (SRP54 and SRa) following the ER markers. (**B**) Peptides (in red) from *N. gruberi* mtFfh and mtFtsY identified by tandem mass spectrometry (nLC-MS2). The N-terminal regions of the two proteins highlighted in yellow correspond to the leader sequences tested (as fusions with mNeonGreen) in subcellular targeting experiments in the heterologous system of *Trypanosoma brucei*. Further technical details on the proteomic analysis are provided in Horváthová et al. (2021).

(A) *N. gruberi* mtFfh gene: revised sequence and model

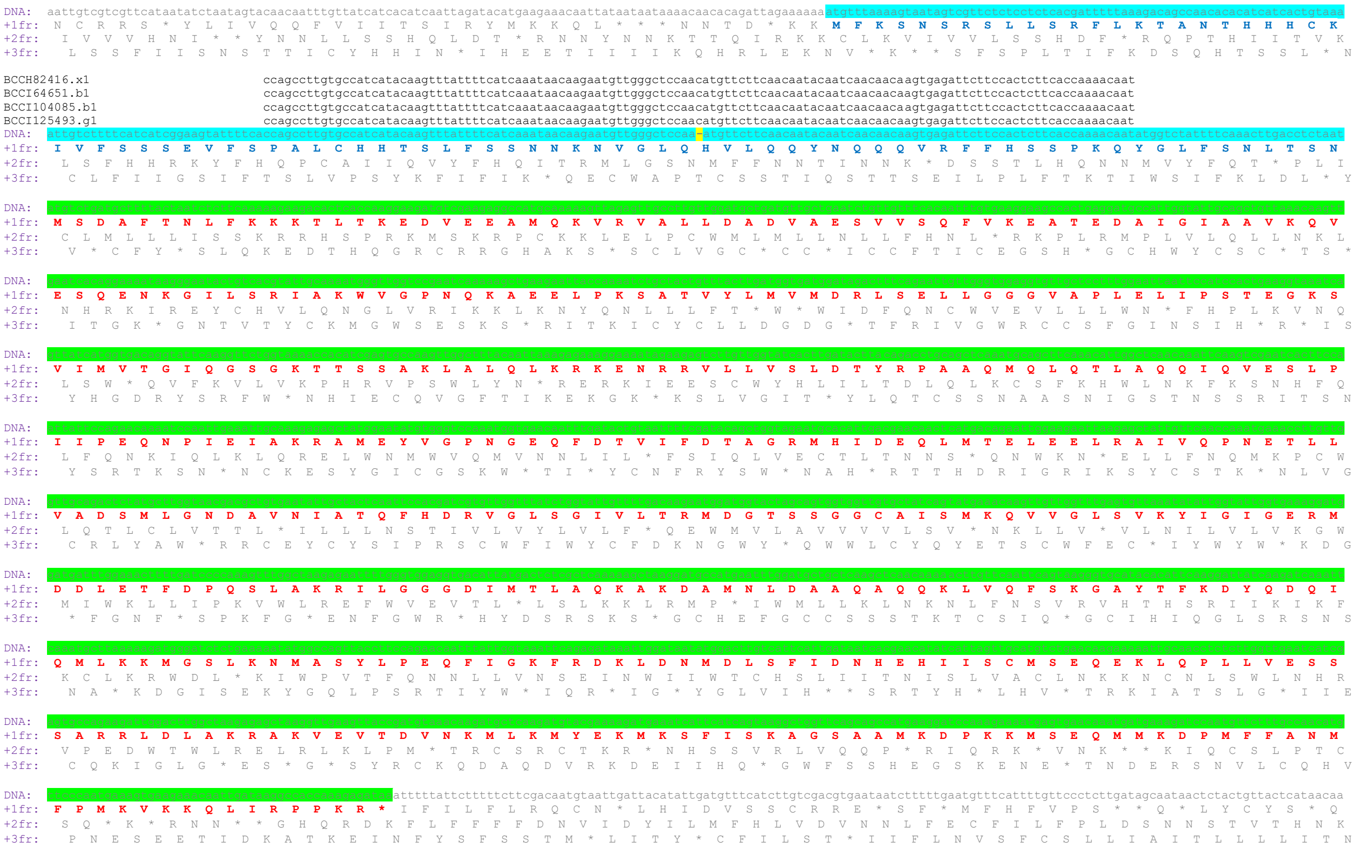

(B) Alignment of mtFfh protein sequences from *Naegleria* spp.

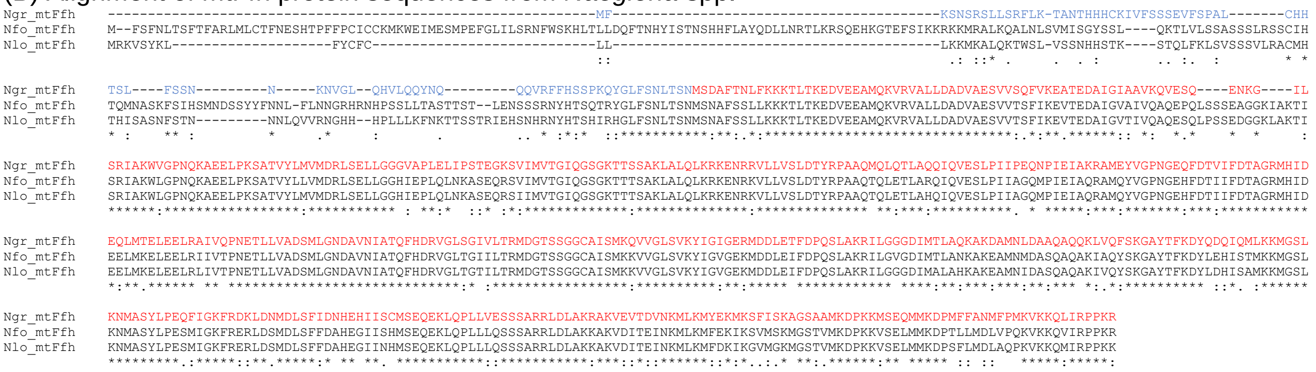

(C) *N. gruberi* mtFtsY gene: revised model

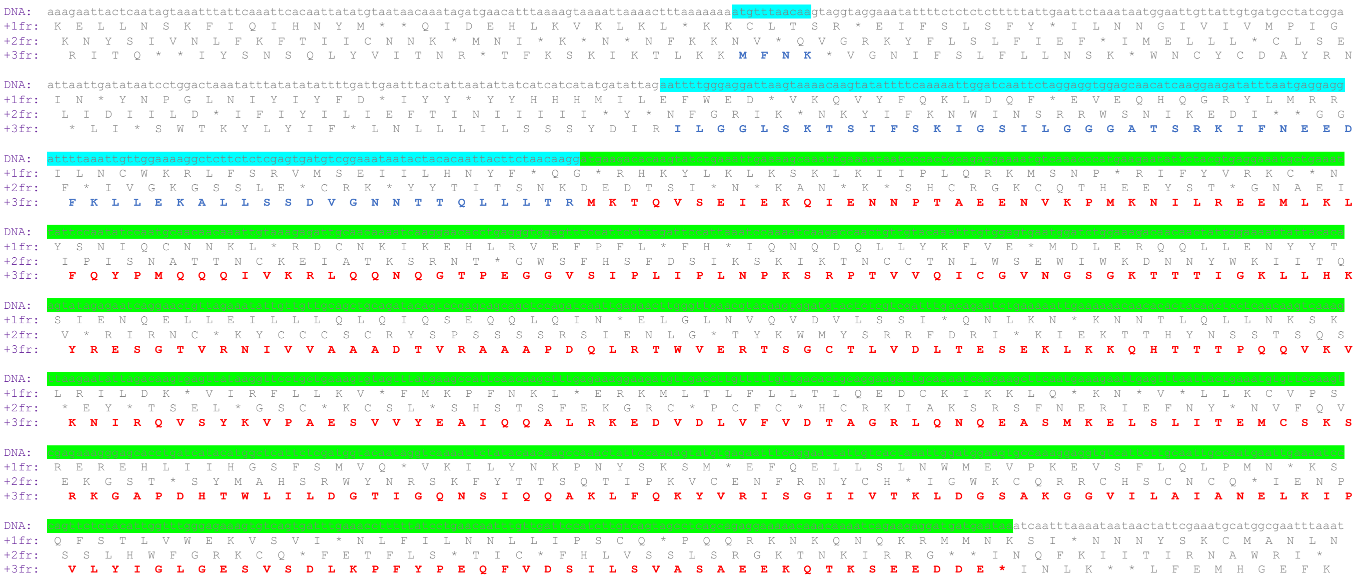

(D) Alignment of mtFtsY protein sequences from *Naegleria* spp.

```
Ngr_mtFtsY    MFNKILGGLSKTS--IFSKIGSILGGGATS-RKIFNEEDFKLLEKALLSSDVGNNTTQLLLTRMKTQVSEIEKQIENNPTAEENVKPMKNILREEMLKLFQYPMQQQIVKRLQQNQGTPEGGVSIPLIPLNPKSRPTVVQICGVNGSGKT
Nfo_mtFtsY    MFSRILGGLSKTSSTIFSKIGSILGGSANAQRKVFNEEDFKLLESALLSSDVGNNTTQLLLQRMKSQVTNIEKELENNPNMTD-VKPMKSILREEMLKLFQNPMQQQIVKRLQQNQGTPEEEISVSLTPLSMKSRPTVVQICGVNGSGKT
Nlo_mtFtsY    MFNRILGGLSKTSSTIFSKIGSILGGSSTGPRKVFNEEDFKLLESALLSSDVGNNTTQLLLQRMKTQVTNIEKELELNPNMTD-IKPMKSILREEMLKLFQNPMQQQIVKRLQQNQGTAEQEISVSLIPISVKSRPTVVQICGVNGSGKT
              **.:*********  ***********.:.. **:**********.**************** ***:**::***::* **.  : :****.*********** ****************.*  :*:.* *:. ******************

Ngr_mtFtsY    TTIGKLLHKYRESGTVRNIVVAAADTVRAAAPDQLRTWVERTSGCTLVDLTESEKLKKQ-HTTTPQQVKVKNIRQVSYKVPAESVVYEAIQQALRKEDVDLVFVDTAGRLQNQEASMKELSLITEMCSKSRKGAPDHTWLILDGTIGQNS
Nfo_mtFtsY    TTIGKLLHKYRQSGTVRHMIVAAADTVRAAAPDQLRSWVERTPDCSIVDLTESEKLKKQSE--THQPVKTKNIRQVSYKVPSESVVYEAIQQGLRKEDVDVVFVDTAGRLQNQEASMKELALINEMCSRSRKGAPDHTWLILDGTIGQNS
Nlo_mtFtsY    TSIGKLLHKYRQSGTVRHMIVAAADTVRAAAPDQLRSWVERTPDCSIVDLTESEKLKKQSESATHQPVKTKNIRQVSYKVPAESVVYEAIQQGLRKEDVDVVFVDTAGRLQNQEASMKELALINEMCSRSRKGAPDHTWLVLDGTIGQNS
              *:*********:*****:::****************:*****..*::************ .  * * **.***********:**********.*******:*******************:**.****:***********:*********

Ngr_mtFtsY    IQQAKLFQKYVRISGIIVTKLDGSAKGGVILAIANELKIPVLYIGLGESVSDLKPFYPEQFVDSILSVASAEEKQTKSEEDDE
Nfo_mtFtsY    IQQAKLFQKYVRISGIIVTKLDGSAKGGVILAIANELKIPVLYIGLGESVQDLRPFYPEQFVDSILSVTEQNNEKQDDDEEE-
Nlo_mtFtsY    IQQAKLFQKYVRISGIIVTKLDGSAKGGVILAIANELKIPVLYIGLGESVQDLRPFYPEQFVDSILSVTEQTKEKPQDEEEE-
              **************************************************.**:**************:.  ::: ..:*::
```

Supplementary fig. S2. Revisions of existing models of *Naegleria gruberi* mtFfh and mtFtsY genes. (**A**) and (**C**) Nucleotide sequences of the respective genes extracted from the current genome assembly; mtFfh gene – GenBank accession number ACER01000115.1 (positions 64001 to 66000, reverse complement sequence), mtFtsY – GenBank accession number ACER01000034.1 (positions 330136 to 331575). Coding sequences as delimited in the existing gene models are highlighted in green, extensions of the coding sequences resulting from model revisions are highlighted in turquoise. Conceptual translation in all three sense frames is shown beneath the sequences; amino acids constituting the currently predicted protein (mtFfh – XP_002680081.1, mtFtsY – XP_002682642.1) are in red, N-terminal extensions resulting from revised gene models are in blue. The sequence of the mtFfh gene is impacted by a one-nucleotide deletion (highlighted in yellow), as is apparent from a comparison with raw Sanger sequencing reads (segments of four exemplar reads aligning to the region containing the assembly error are shown above the gene sequence). (**B**) and (**D**) Multiple alignments of revised (*N. gruberi*) or *de novo* predicted (*Naegleria fowleri* and *Naegleria lovanensis*) sequences of mtFfh and mtFtsY proteins, respectively. The alignments are shown to document that homologous sequences from other *Naegleria* species support the validity of the revisions of the existing *N. gruberi* gene modes. Note that the N-terminus of mtFfh in *Naegleria* spp. is poorly conserved and it is possible that in case of *N. fowleri* and *N. lovanensis* the actual start of the protein is at a methionine residue downstream of that used in the scheme. Still, the alignment clearly supports the extension of the N-terminal region of the *N. gruberi* beyond the existing model.

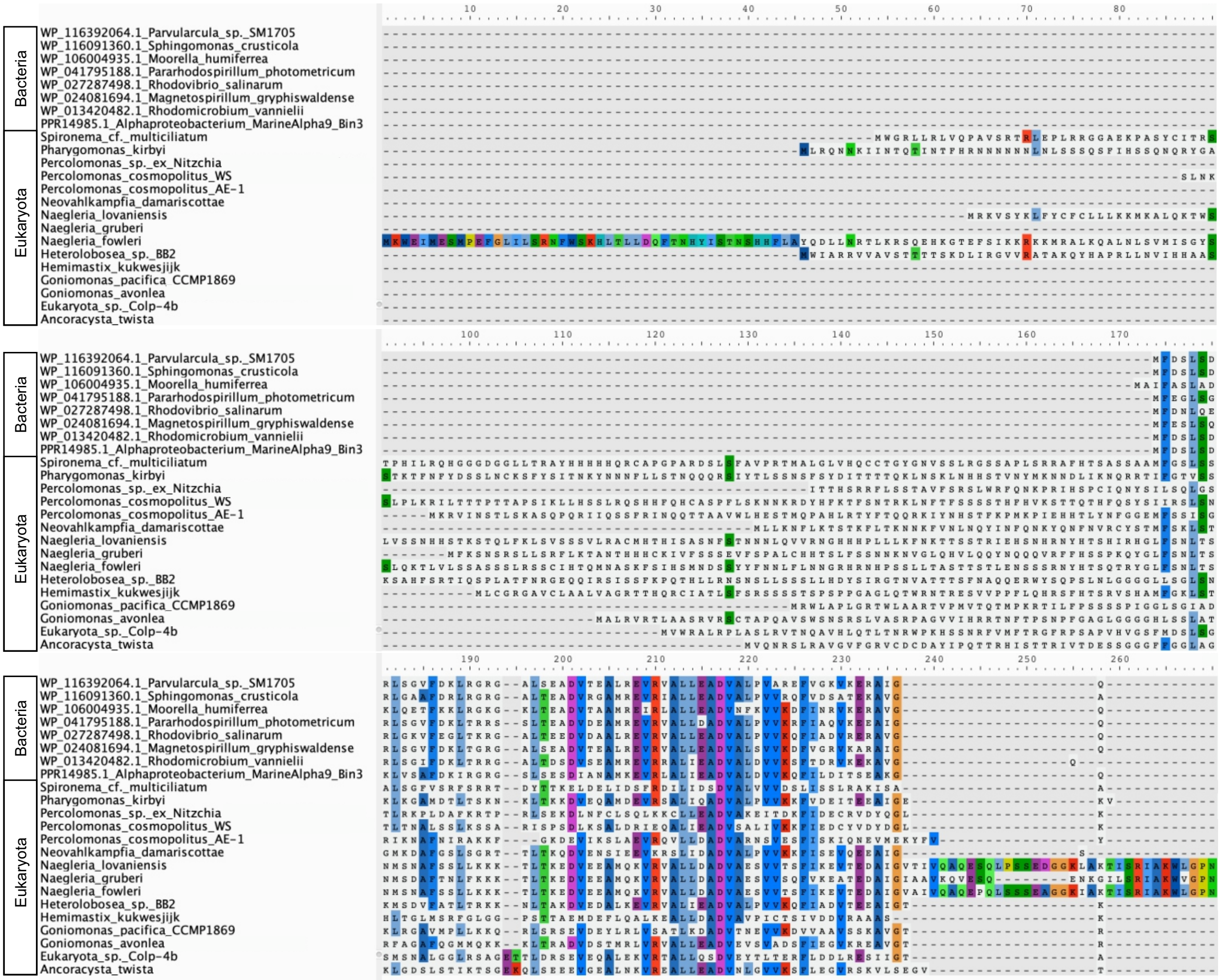

Supplementary fig. S3. Multiple sequence alignment of bacterial and mitochondrial Ffh proteins. All detected mitochondrial homologs possess N-terminal extensions compared to bacterial proteins (positions 1–171). The alignment was constructed in Geneious Prime 2019.2.3 using the Geneious alignment tool with default settings. The full alignment contains 750 positions, only positions 1–270 are shown. Note that the sequences from *Percolomonas* sp. ex *Nitzschia* and *Percolomonas cosmpolitus* WS are truncated and the N-terminal extensions of the respective proteins are presumably longer than shown in the figure.

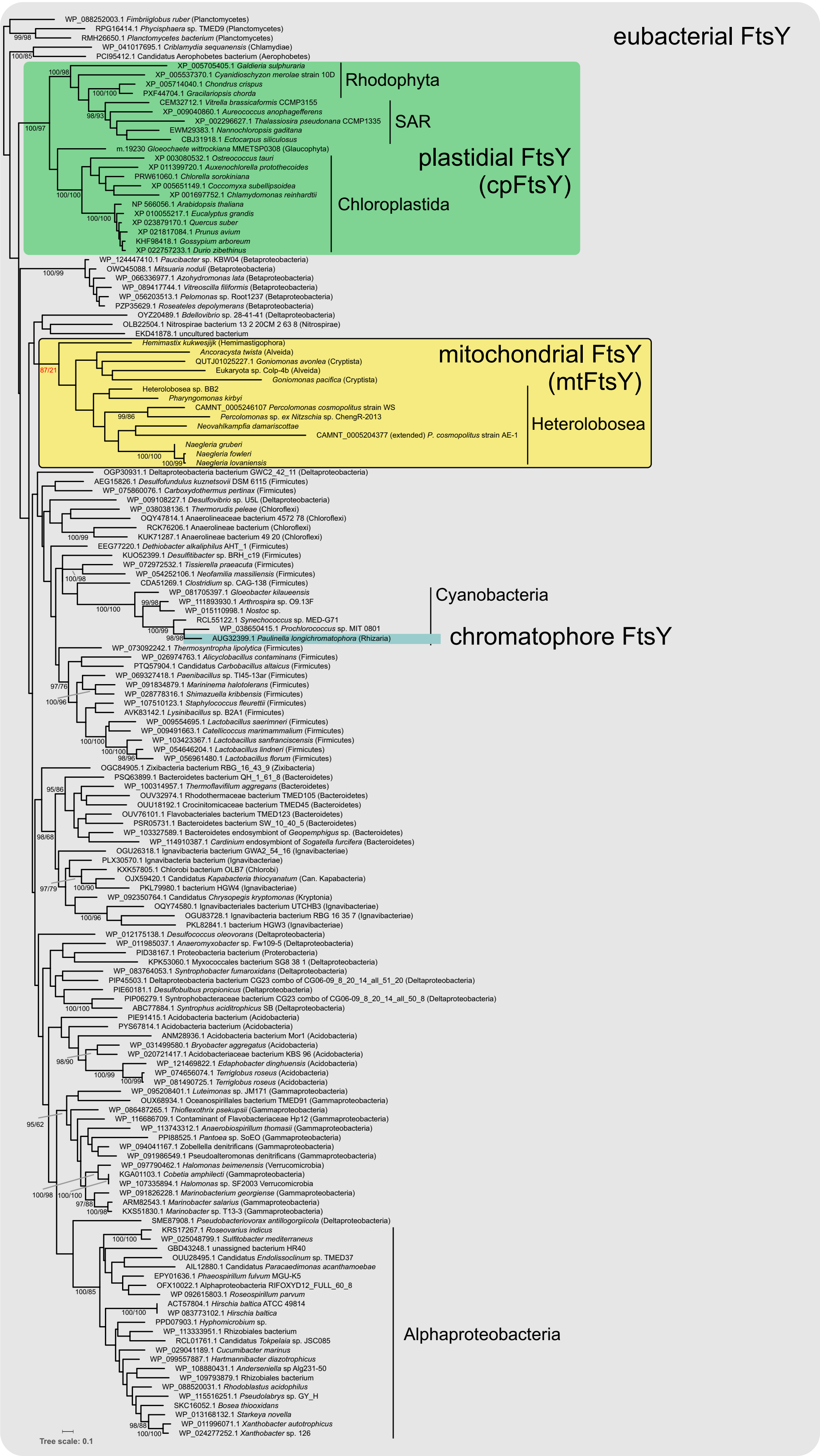

Supplementary fig. S4. Phylogenetic analysis of eubacterial and organellar FtsY proteins. The tree topologies shown correspond to maximum likelihood tree (LG4X substitution model) computed with IQ-TREE based on a multiple alignment of 154 sequences and 287 amino acid positions. Branch support was assessed by ultrafast bootstrapping (N=1000, IQ-TREE) and rapid bootstrapping (N=500, RAxML) using the same model. The full trees from both methods are also provided in the Newick format in supplementary dataset S1, Supplementary Material online.

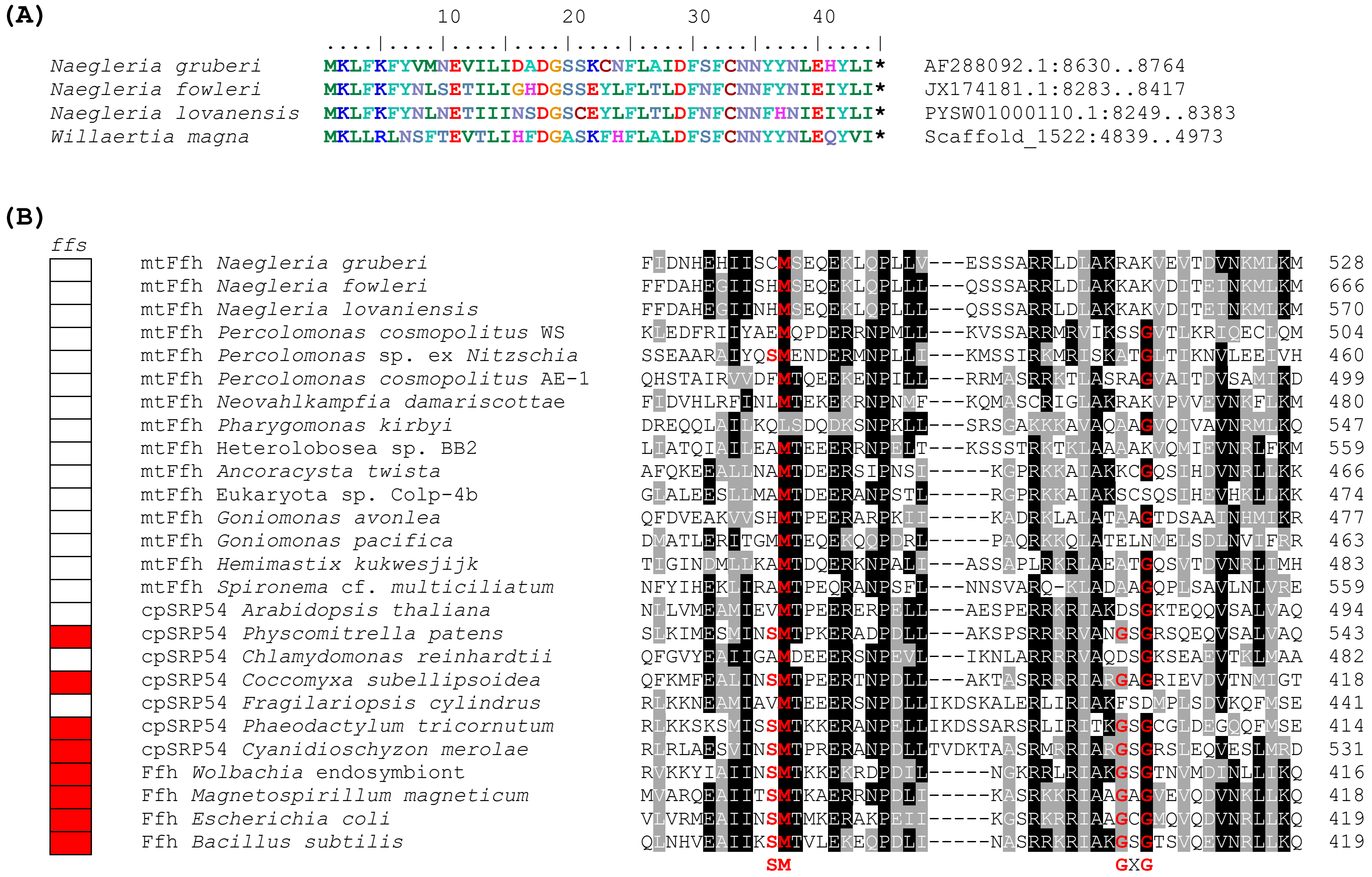

Supplementary fig. S5. No evidence for mitochondrial 4.5S RNA. (**A**) A novel short conserved unannotated ORF found in mitogenomes of certain Heterolobosea when searching for potential missed *ssrA* gene in intergenic regions. Accession numbers of the respective nucleotide sequences and coordinates defining the ORF are given to the right. The mitogenome sequence from *Willaertia magna* was detected in the whole genome assembly reported by Hasni et al. (*Scientific Reports* 2019, 9:18318, doi:10.1038/s41598-019-54580-6). (**B**) Mitochondrial Ffh has substituted the amino acid residues critical for binding 4.5S RNA (the RNA component of the signal recognition particle). Displayed is a segment of a multiple sequence alignment of mtFfh (the protein component of the mitochondrial SRP) and their homologs, including plastidial cpSRP45 and bacterial Ffh. The region shown includes two motifs involved in binding of the 4.5S RNA molecule (the four residues critical for the binding are highlighted in red). The red rectangles on the left indicate the presence of a discernible 4.5S RNA-specifying *ffs* gene in the respective organellar (mtFfh, cpSRP54) or bacterial (Ffh) genome. Note the perfect correlation between the conservation of the 4.5S RNA-binding residues in the protein and the presence of the *ffs* gene.

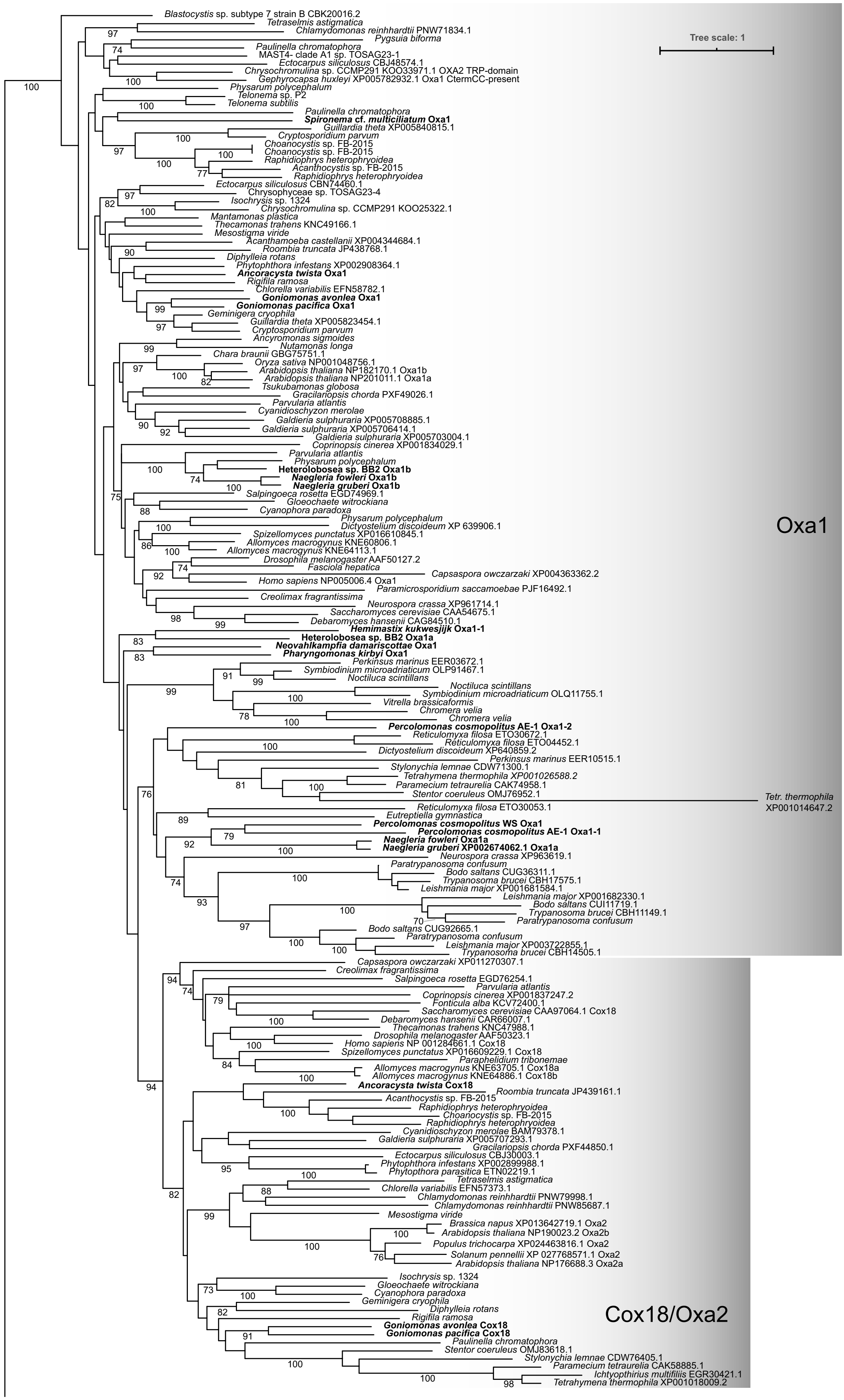
Tree scale: 1
Oxa1
Cox18/Oxa2
Blastocystis sp. subtype 7 strain B CBK20016.2
Tetraselmis astigmatica
Chlamydomonas reinhardtii PNW71834.1
Pygsuia biforma
Paulinella chromatophora
MAST4- clade A1 sp. TOSAG23-1
Ectocarpus siliculosus CBJ48574.1
Chrysochromulina sp. CCMP291 KOO33971.1 OXA2 TRP-domain
Gephyrocapsa huxleyi XP005782932.1 Oxa1 CtermCC-present
Physarum polycephalum
Telonema sp. P2
Telonema subtilis
Paulinella chromatophora
Spironema cf. multiciliatum Oxa1
Guillardia theta XP005840815.1
Cryptosporidium parvum
Choanocystis sp. FB-2015
Choanocystis sp. FB-2015
Raphidiophrys heterophryoidea
Acanthocystis sp. FB-2015
Raphidiophrys heterophryoidea
Ectocarpus siliculosus CBN74460.1
Chrysophyceae sp. TOSAG23-4
Isochrysis sp. 1324
Chrysochromulina sp. CCMP291 KOO25322.1
Mantamonas plastica
Thecamonas trahens KNC49166.1
Mesostigma viride
Acanthamoeba castellanii XP004344684.1
Roombia truncata JP438768.1
Diphylleia rotans
Phytophthora infestans XP002908364.1
Ancoracysta twista Oxa1
Rigifila ramosa
Chlorella variabilis EFN58782.1
Goniomonas avonlea Oxa1
Goniomonas pacifica Oxa1
Geminigera cryophila
Guillardia theta XP005823454.1
Cryptosporidium parvum
Ancyromonas sigmoides
Nutamonas longa
Chara braunii GBG75751.1
Oryza sativa NP001048756.1
Arabidopsis thaliana NP182170.1 Oxa1b
Arabidopsis thaliana NP201011.1 Oxa1a
Tsukubamonas globosa
Gracilariopsis chorda PXF49026.1
Parvularia atlantis
Cyanidioschyzon merolae
Galdieria sulphuraria XP005708885.1
Galdieria sulphuraria XP005706414.1
Galdieria sulphuraria XP005703004.1
Coprinopsis cinerea XP001834029.1
Parvularia atlantis
Physarum polycephalum
Heterolobosea sp. BB2 Oxa1b
Naegleria fowleri Oxa1b
Naegleria gruberi Oxa1b
Salpingoeca rosetta EGD74969.1
Gloeochaete witrockiana
Cyanophora paradoxa
Physarum polycephalum
Dictyostelium discoideum XP 639906.1
Spizellomyces punctatus XP016610845.1
Allomyces macrogynus KNE60806.1
Allomyces macrogynus KNE64113.1
Drosophila melanogaster AAF50127.2
Fasciola hepatica
Capsaspora owczarzaki XP004363362.2
Homo sapiens NP005006.4 Oxa1
Paramicrosporidium saccamoebae PJF16492.1
Creolimax fragrantissima
Neurospora crassa XP961714.1
Saccharomyces cerevisiae CAA54675.1
Debaromyces hansenii CAG84510.1
Hemimastix kukwesjijk Oxa1-1
Heterolobosea sp. BB2 Oxa1a
Neovahlkampfia damariscottae Oxa1
Pharyngomonas kirbyi Oxa1
Perkinsus marinus EER03672.1
Symbiodinium microadriaticum OLP91467.1
Noctiluca scintillans
Noctiluca scintillans
Symbiodinium microadriaticum OLQ11755.1
Vitrella brassicaformis
Chromera velia
Chromera velia
Percolomonas cosmopolitus AE-1 Oxa1-2
Reticulomyxa filosa ETO30672.1
Reticulomyxa filosa ETO04452.1
Dictyostelium discoideum XP640859.2
Perkinsus marinus EER10515.1
Stylonychia lemnae CDW71300.1
Tetrahymena thermophila XP001026588.2
Paramecium tetraurelia CAK74958.1
Stentor coeruleus OMJ76952.1
Tetr. thermophila XP001014647.2
Reticulomyxa filosa ETO30053.1
Eutreptiella gymnastica
Percolomonas cosmopolitus WS Oxa1
Percolomonas cosmopolitus AE-1 Oxa1-1
Naegleria fowleri Oxa1a
Naegleria gruberi XP002674062.1 Oxa1a
Neurospora crassa XP963619.1
Paratrypanosoma confusum
Bodo saltans CUG36311.1
Trypanosoma brucei CBH17575.1
Leishmania major XP001681584.1
Leishmania major XP001682330.1
Bodo saltans CUI11719.1
Trypanosoma brucei CBH11149.1
Paratrypanosoma confusum
Bodo saltans CUG92665.1
Paratrypanosoma confusum
Leishmania major XP003722855.1
Trypanosoma brucei CBH14505.1
Capsaspora owczarzaki XP011270307.1
Creolimax fragrantissima
Salpingoeca rosetta EGD76254.1
Parvularia atlantis
Coprinopsis cinerea XP001837247.2
Fonticula alba KCV72400.1
Saccharomyces cerevisiae CAA97064.1 Cox18
Debaromyces hansenii CAR66007.1
Thecamonas trahens KNC47988.1
Drosophila melanogaster AAF50323.1
Homo sapiens NP 001284661.1 Cox18
Spizellomyces punctatus XP016609229.1 Cox18
Paraphelidium tribonemae
Allomyces macrogynus KNE63705.1 Cox18a
Allomyces macrogynus KNE64886.1 Cox18b
Ancoracysta twista Cox18
Roombia truncata JP439161.1
Acanthocystis sp. FB-2015
Raphidiophrys heterophryoidea
Choanocystis sp. FB-2015
Raphidiophrys heterophryoidea
Cyanidioschyzon merolae BAM79378.1
Galdieria sulphuraria XP005707293.1
Gracilariopsis chorda PXF44850.1
Ectocarpus siliculosus CBJ30003.1
Phytophthora infestans XP002899988.1
Phytopthora parasitica ETN02219.1
Tetraselmis astigmatica
Chlorella variabilis EFN57373.1
Chlamydomonas reinhardtii PNW79998.1
Chlamydomonas reinhardtii PNW85687.1
Mesostigma viride
Brassica napus XP013642719.1 Oxa2
Arabidopsis thaliana NP190023.2 Oxa2b
Populus trichocarpa XP024463816.1 Oxa2
Solanum pennellii XP 027768571.1 Oxa2
Arabidopsis thaliana NP176688.3 Oxa2a
Isochrysis sp. 1324
Gloeochaete witrockiana
Cyanophora paradoxa
Geminigera cryophila
Diphylleia rotans
Rigifila ramosa
Goniomonas avonlea Cox18
Goniomonas pacifica Cox18
Paulinella chromatophora
Stentor coeruleus OMJ83618.1
Stylonychia lemnae CDW76405.1
Paramecium tetraurelia CAK58885.1
Ichtyopthirius multifiliis EGR30421.1
Tetrahymena thermophila XP001018009.2

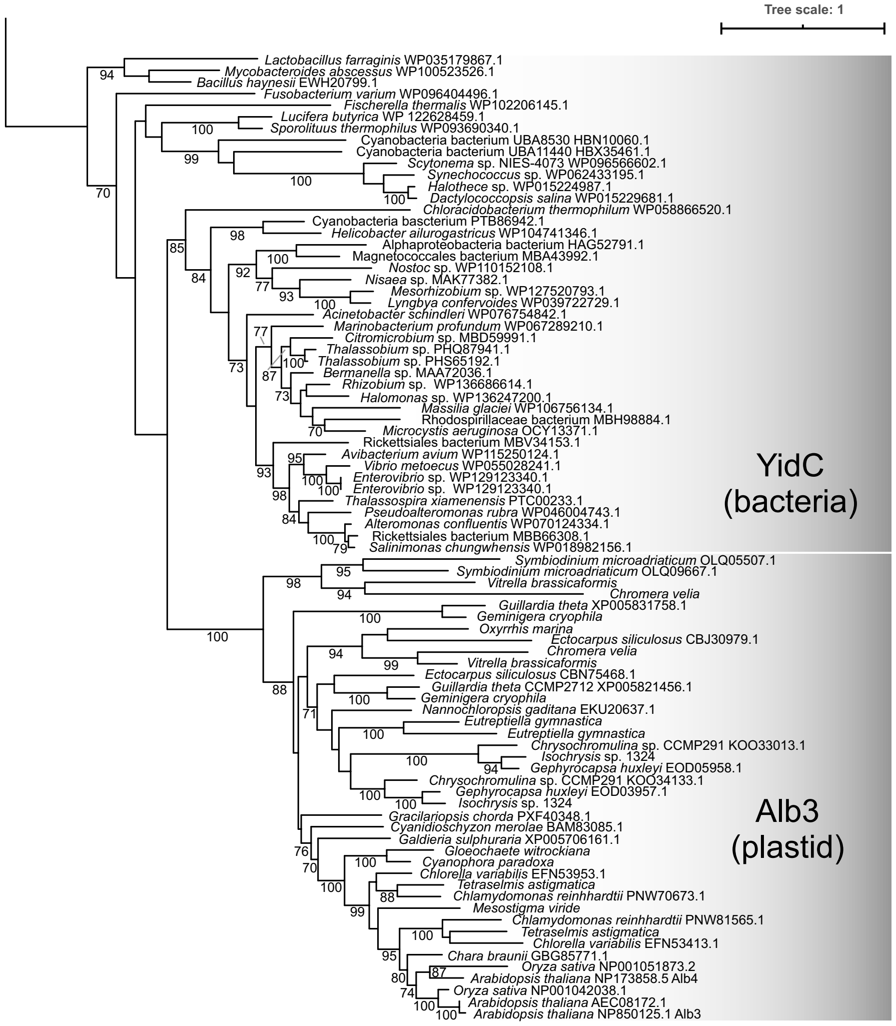

Supplementary fig. S6. Phylogenetic analysis of the YidC/Alb3/Oxa1 family. A selection of bacterial YidC sequences and their eukaryotic homologs, i.e. the plastidial Alb3 and the mitochondrial Oxa1 and Cox18, was aligned using PASTA (Mirab et al. 2015 - doi.org/10.1089/cmb.2014.0156) and trimmed manually to remove poorly conserved regions. The final alignment (222 amino acid positions, 251 sequences) was subjected to tree inference using IQ-TREE multicore version 1.6.12, with the LG+C20+F+G4 substitution model and 1000 ultrafast bootstraps (bnni). Bootstrap values are shown when ≥70%. The tree was arbitrarily rooted between YidC/Alb3 and Oxa1/Cox18 groups and separated into two parts, each displayed on a separate page. The resolution of the tree is inherently limited by the low sequence conservation and the small length of the alignment. As a result, the Oxa1 and Cox18, presumably two separate deeply diverged eukaryotic paralogs, are not clearly separated in the tree (Oxa1 is paraphyletic). The sequences from mtFfh/mtFtsY-carrying species are highlighted in boldface; their sequence IDs are provided in supplementary table S5. Note that the Oxa1-2 sequence from *Hemimastix kukwesjijk* (Hemi2|18767_TR6208_c0_g1_i1) is very short and hence was not included in the analysis.

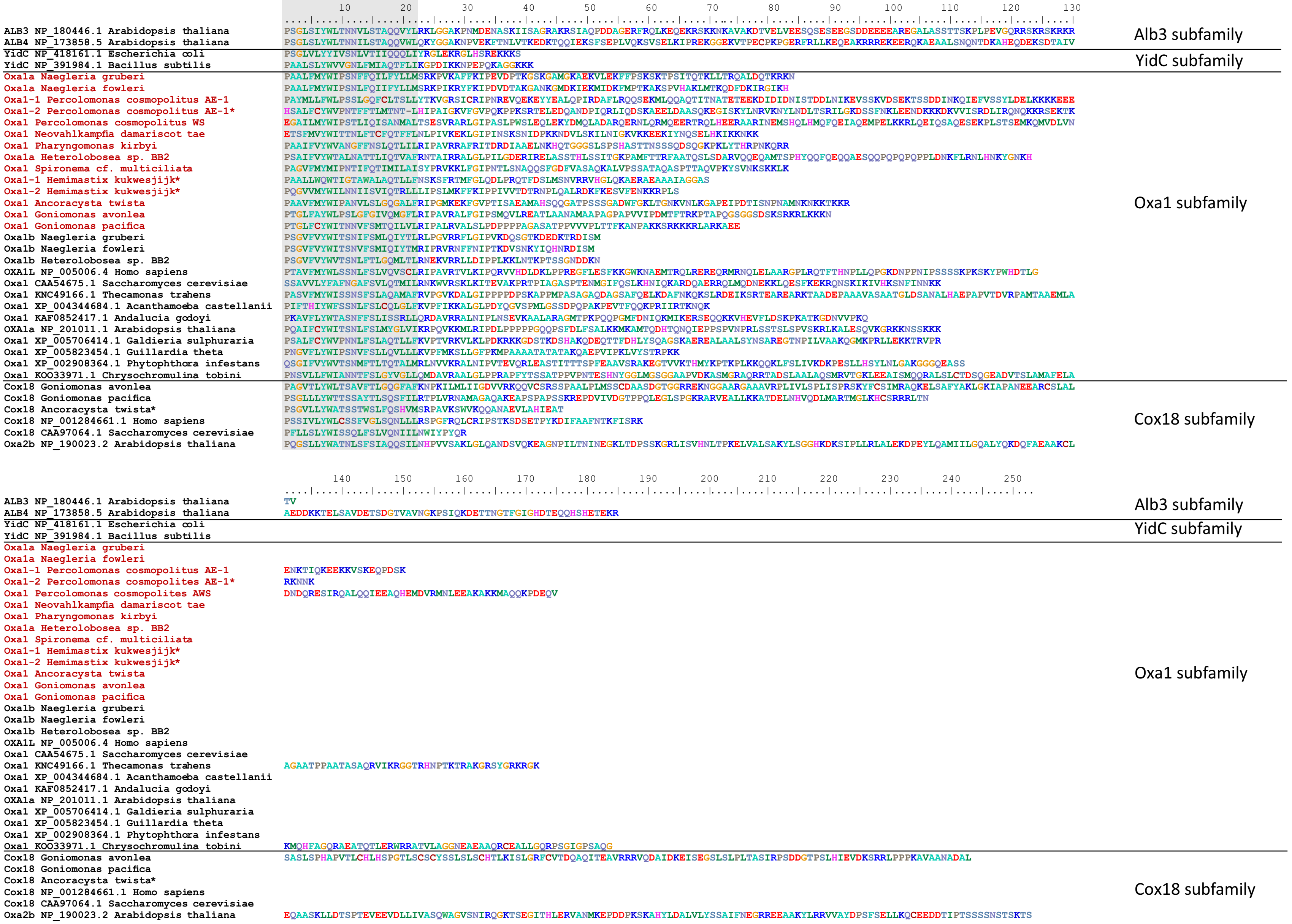

Supplementary fig. S7. Putative ribosome-binding C-terminal extensions in Oxa1 proteins of diverse eukaryotes. The figure shows multiple sequence alignment of the C-terminal segment, including the last transmembrane helix (highlighted in a gray box) and the regions downstream, of selected representatives of the YidC/Alb3/Oxa1 family. Note the presence of C-terminal extensions (poorly conserved in sequence and length) in the eukaryotic Oxa1 proteins, including those from mtFfh/mtFtsY-carrying taxa (highlighted in red) compared to the reference bacterial YidC sequences. The extensions of some sequences (marked with an asterisk) are in fact longer than apparent from the figure, as the available sequences are truncated. Sequence IDs of proteins from the mtFfh/mtFtsY-carrying taxa are provided in supplementary table S5. The classification of the sequences into the Oxa1 and Cox18 subfamilies is based on the phylogenetic analyses presented in supplementary fig. S6.

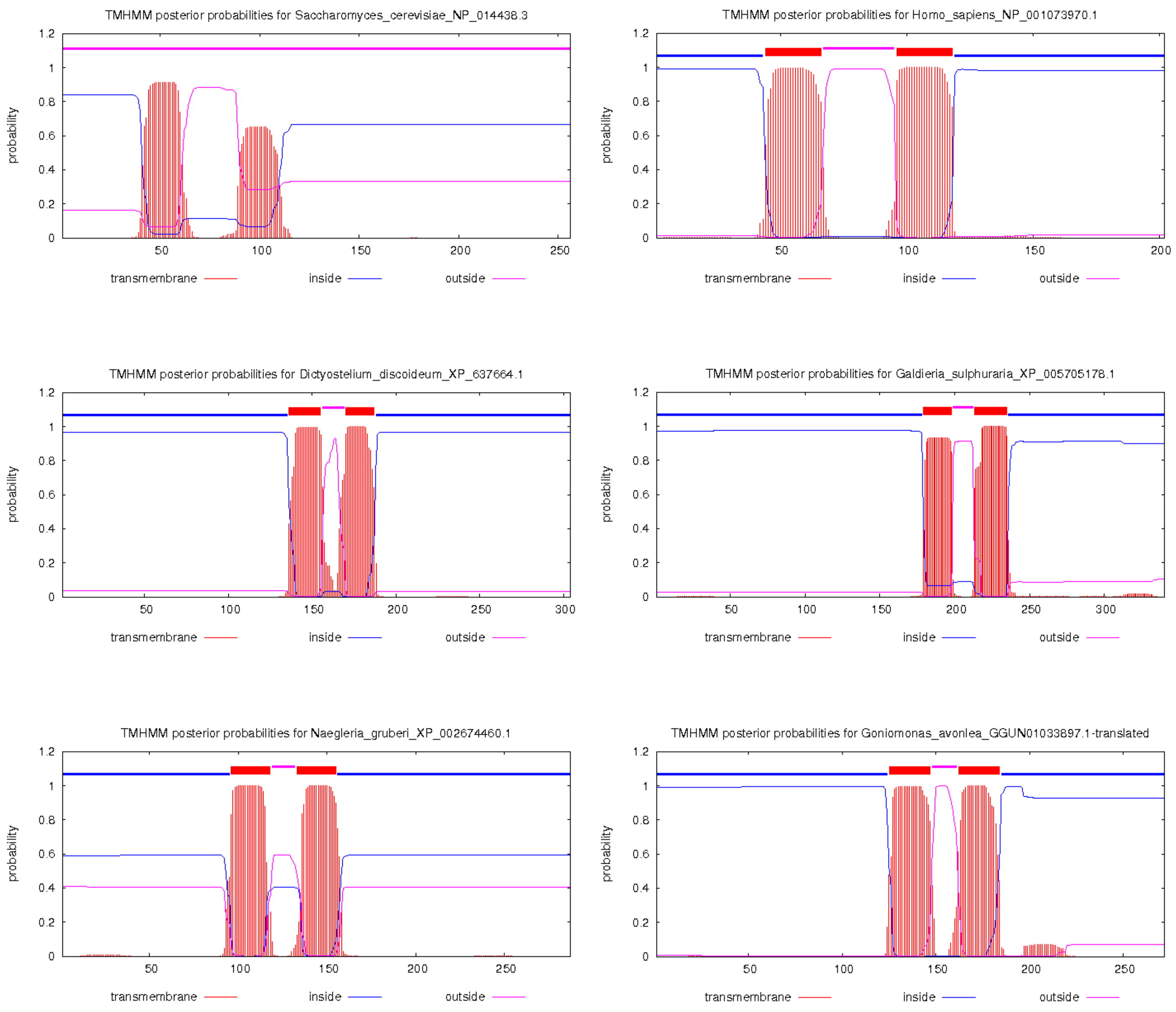

Supplementary fig. S8. Two transmembrane domains are a conserved feature of proteins of the Mrx15/TMEM223 family. Selected representatives of the family (identified with PSI-BLAST of HMMER searches) were evaluated by the TMHMM Server v. 2.0 (http://www.cbs.dtu.dk/services/TMHMM/). Note that despite the below-threshold probability of transmembrane domains in the *S. cerevisiae* Mrx15 protein (top left), their presence has been confirmed experimentally (Möller-Hergt et al. 2018).
